# Selective Copper-Catalyzed Cross-Coupling of Cyclopropyl and Cyclobutyl 1,2-Bis(boronates)

**DOI:** 10.1021/acs.orglett.6c01560

**Published:** 2026-06-03

**Authors:** Marina Velado, Javier Teresa, Roberto Fernández de la Pradilla, Alma Viso, Mariola Tortosa

**Affiliations:** † Organic Chemistry Department, 16722 Universidad Autónoma de Madrid (UAM), 28049 Madrid, Spain; ‡ Instituto de Química Orgánica General (IQOG), CSIC, Juan de la Cierva 3, 28006 Madrid, Spain; § Institute for Advanced Research in Chemical Sciences (IAdChem), Universidad Autónoma de Madrid, Madrid 28049, Spain

## Abstract

Herein we disclose an approach to perform the selective copper-catalyzed cross-coupling of 1,2-diborylcyclopropanes and cyclobutanes with different electrophiles. In the case of 1,2-diborylcyclopropanes, a simple alkoxide base was needed to promote the cross-coupling with either allyl halides, propargyl halides, alkynyl halides, or electrophilic amines, which highlights the special reactivity of these diboron compounds. In the case of 1,2-diborylspirocyclobutanes, a site-selective activation of the less hindered boron was observed. Further functionalization of the remaining boryl moiety provides access to highly functionalized stereodefined cyclopropanes and spirocyclobutanes.

## Graphical abstract

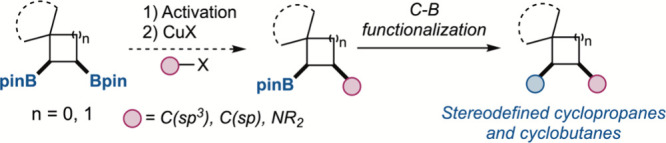

Bis­(boronic esters) have attracted the interest of the synthetic community over the years due to their high versatility.^1^ Among them, 1,2-bis­(boronates) are particularly attractive synthetic intermediates due to the possibility of selectively functionalizing both boryl moieties, allowing rapid and diverse assembly of molecular complexity.^2^ Moreover, 1,2-bis­(boronates) can be readily prepared from alkenes using transition-metal catalysis^3^ or Lewis base activation, including their preparation as enantioenriched compounds.^4^

In this context, the palladium-catalyzed Suzuki-Miyaura cross-coupling of terminal 1,2-bis­(boronates) with sp^2^-electrophiles has been widely explored, allowing for selective functionalization of the less-substituted boryl unit (Figure 1a).^5^ In contrast, the selective functionalization of internal 1,2-bis­(boronates) remains a difficult task, with only a few examples reported with cyclic derivatives.^6^

**Figure 1:**
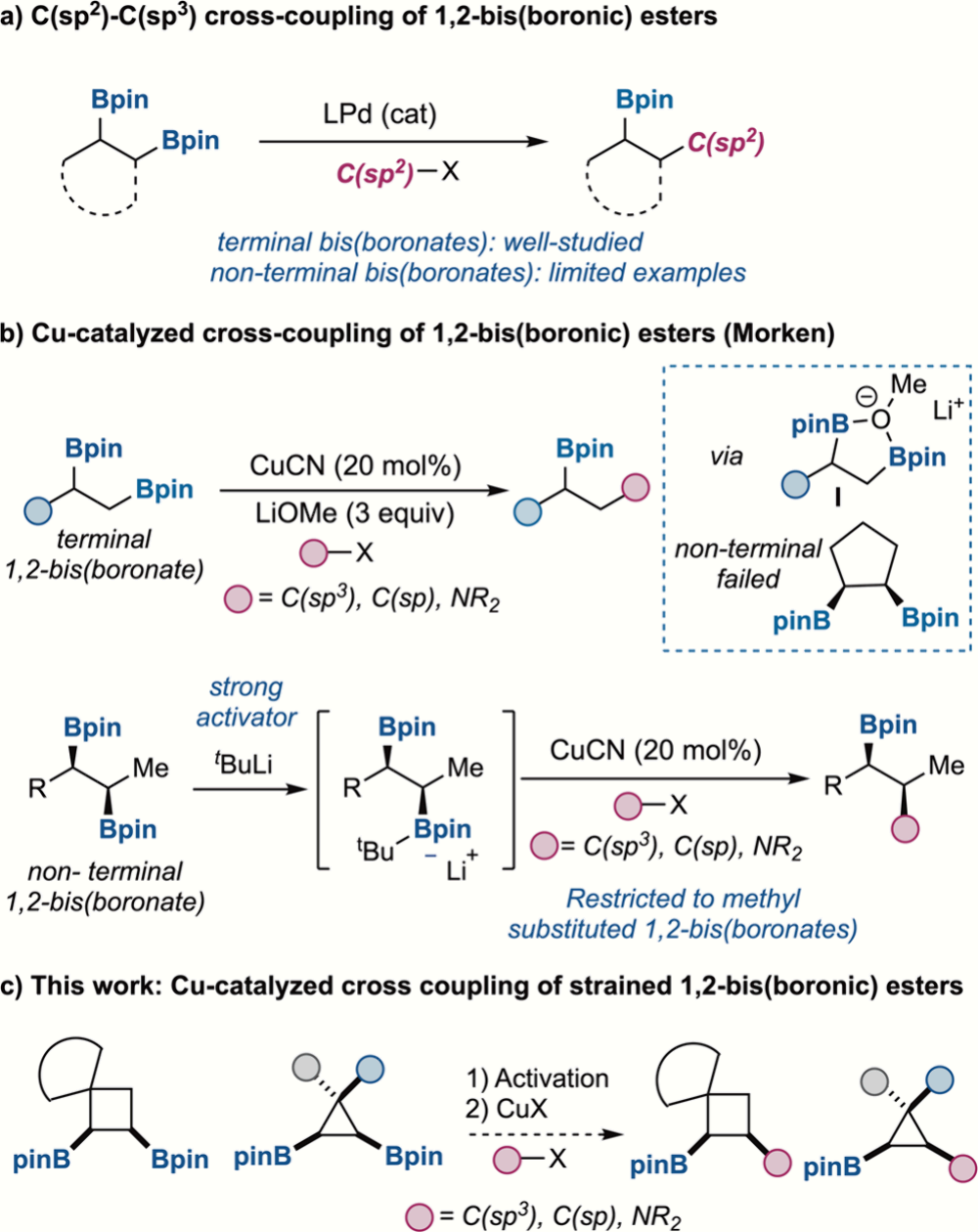
Metal-catalyzed functionalization of 1,2-bis­(boronates).

Searching to expand the range of electrophiles, Morken and co-workers reported in a seminal contribution the copper-catalyzed cross-coupling of terminal 1,2-bis­(boronates) (Figure 1b).^7^ In this study, different sp^3^- and sp-electrophiles as well as electrophilic amines were used in the presence of CuCN (20 mol %) and LiOMe. The formation of 5-membered heterocyclic complex **I** with a single oxygen atom bridging both boron atoms was proposed to have a key role in the transmetalation step. However, internal 1,2-bis­(boronates) were not reactive under these conditions (Figure 1b). Shortly after, the same authors reported the copper-catalyzed cross-coupling of methyl-substituted internal derivatives using this time *^t^* BuLi as an activator (Figure 1b).^8^ The use of other internal bis­(boronates) in copper-catalyzed cross-coupling reactions has remained elusive.

Following our interest in the functionalization of strained 1,2-bis­(boronates),^6b,6c^ we were intrigued about the possibility of using copper catalysis to achieve selective functionalization of one of the two boryl moieties. We reasoned that the increased sp^2^ character of the C–B bond in these compounds could overcome the lack of reactivity observed with nonterminal 1,2-bisboronates, providing an entry to difficult to access cyclopropanes and spirocyclobutanes (Figure 1c).

We started our study with bis­(boryl)­cyclopropane **1a** as a model substrate. We first attempted the cross-coupling under the conditions reported for methyl-substituted 1,2-bis­(boronates).^8^ Using allyl bromide, *^t^* BuLi as activator, and 20 mol % CuCN, extensive protodeborylation byproduct was produced.^9^ Trying to minimize the protodeborylation, we turned our attention to the use of conditions previously reported for more reactive terminal 1,2-bis­(boronates) (Table 1).^7^ When we treated cyclopropane **1a** with an excess of LiOMe (3 equiv), 20 mol % CuCN, and allyl bromide, for 3 h at 60 °C, we were pleased to observe the formation of the allylated cyclopropyl boronate **2a** in almost quantitative yield (entry 1). Compound **2a** was obtained as a single diastereomer showing that the cross-coupling was stereoretentive. The unusually high reactivity under these conditions compared with the unreactive cyclopentane derivative (Figure 1b) might be due to the increased strain imposed by the cyclopropane in intermediate complexes such as **I** (Figure 1b).^6c^ This structural feature would facilitate the reaction by increasing the relaxation of the cyclic structure in **I** upon achieving the transition state for the transmetalation step. These results led us to explore lower catalyst loadings as well as lower temperatures (entries 2 and 3). Interestingly, the product was obtained even at room temperature and using 5 mol % CuCN, although more extended reaction times were needed (24 h).

**Table 1:**
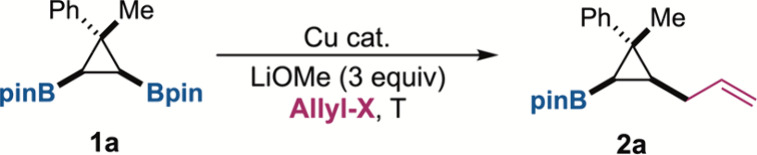
Optimization of the Allylation of 1,2-Bis­(boryl)­cyclopropanes^a^

entry	Cu catalyst	X	*T* (°C)	yield
1	CuCN (20%)	Br	60	98
2	CuCN (10%)	Br	40	98
3	CuCN (5%)	Br	rt	98
4	CuCl (5%)	Br	rt	95
5	CuCl (5%)	I	rt	95
6	CuCl (5%)	OTs	rt	-
7	CuCN (5%)	OTs	rt	99
8	CuCl (5%)	Cl	rt	47

a 0.2 mmol scale. Yields were calculated by ^1^H NMR using an internal standard.

Then, we wanted to explore the use of less toxic copper catalysts. Pleasingly, using 5 mol % CuCl was similarly effective (entry 4). Different leaving groups on the electrophile were also tested (entries 5–8). Both allylic bromides and iodides behaved similarly. However, allylic sulfonate provided the product with outstanding yield only when CuCN was used as the catalyst. Allyl chloride also provided the product, albeit in lower yield, with a mixture of both allylated and protodeborylated products.

Having suitable conditions in hand, we proceeded to examine the scope of allylation with different substrates (Scheme 1). We demonstrated that different 1,2-bis­(boryl)­cyclopropanes could be efficiently allylated using 5 mol % CuCl at room temperature, obtaining the products in good yields (**2a**-**d**). Also, 2-substituted allyl bromides could be used as coupling partners. Bromide (**2e**-**h**), methyl (**2i**), phenyl (**2j**), and bromomethyl (**2k**) in the 2-position of the allyl bromide were well tolerated and provided the products effectively. When a 3-substituted allyl bromide was used, the product was obtained in good yield, albeit with moderate diastereoselectivity (**2l**).

**Scheme 1:**
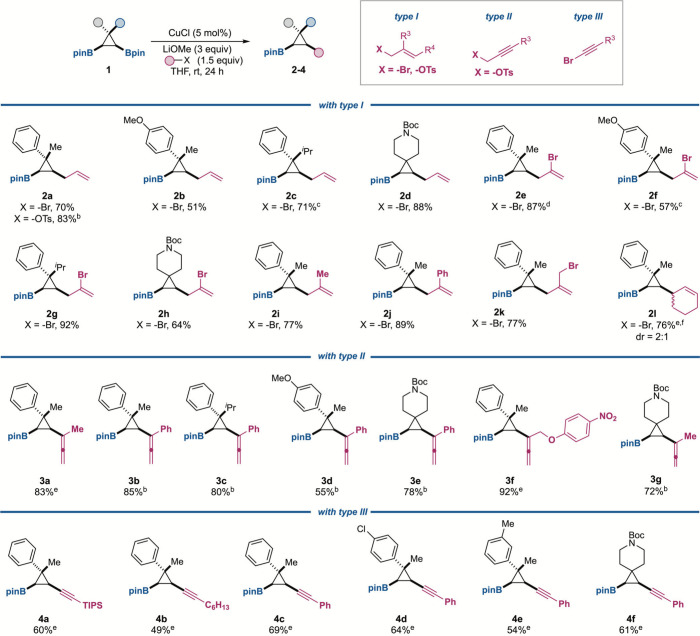
Scope of the Copper-Catalyzed Cross-Coupling of 1,2-Bis­(boryl)­cyclopropanes with Electrophiles^a^

a 0.10–0.80 mmol scale. Isolated yields. >20:1 dr in all cases unless otherwise stated.
b CuCN (5 mol %) was used.
c 48 h of reaction time.
d 1.3 mmol scale.
e CuCN (20 mol %), 60 °C, 2–4 h.
f dr’s determined by ^1^H NMR of the crude mixture.

Interestingly, propargyl tosylates were also effective coupling partners in this transformation. By using slightly modified conditions, different cyclopropyl allenes were prepared in good yields (**3a**-**g**).

Then, we turned our attention to the alkynylation of 1,2-bis­(boryl)­cyclopropanes. Using alkynyl bromides, we found that the reaction was much slower than the allylic counterparts. However, when we used higher catalyst loadings of CuCN (20 mol %) at 60 °C, we were able to obtain synthetically useful yields of the alkynylated cyclopropanes bearing different substitutions (**4a**-**f**).

We then examined electrophilic amines as coupling partners to obtain aminocyclopropyl boronates (Scheme 2). To our delight, after some experimentation (see Table S3, Supporting Information), we found optimal conditions for the amination using CsF and styrene as additives.^10^ Different electrophilic amines could be used as coupling partners, providing aminocyclopropyl boronates in moderate to good yields (**5a**-**f**). Interestingly, the amination of 1,2-bis­(boryl) cyclopropanes did not require the use of *^t^* BuLi as previously observed for methyl-substituted 1,2-bis­(boronates),^8^ showcasing again the effect of the ring strain on the reactivity of these compounds.

**Scheme 2:**
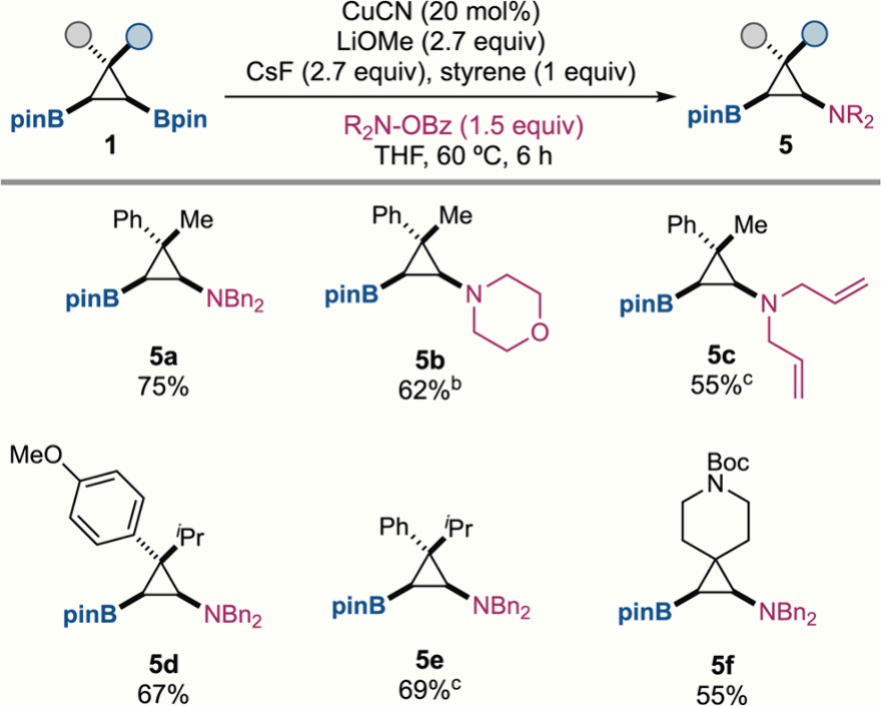
Scope of the Copper-Catalyzed Cross-Coupling with Electrophilic Amines^a^

a 0.20–0.67 mmol scale. Isolated yields. >20:1 dr in all cases unless otherwise stated.
b 2.7 equiv of LiO*^t^* Bu was used instead.
c 24 h of reaction time.

We have previously demonstrated that 1,2-bis­(boryl)­spirocyclobutanes can undergo site-selective transmetalation with a Pd catalyst. Encouraged by the results obtained with 1,2-bis­(boryl)­cyclopropanes, we wondered whether cyclobutanes would react under similar conditions. However, when we tested the copper-catalyzed allylation of 1,2-bis­(boryl)­spirocyclobutanes **6** using the conditions described in Scheme 1, we did not detect the formation of the product. Likely, the difference in strain energy compared to that of cyclopropane is enough to preclude it from an efficient transmetalation.

At this stage, we examined *^t^* BuLi as site-selective activator. To our delight, when we treated these scaffolds with *^t^* BuLi at – 78 °C, followed by allyl bromide in the presence of catalytic amounts of CuCN at 80 °C, we observed the formation of the allylated spirocyclobutyl boronate **7a** in good yield and with full control of the diastereo- and regioselectivity (Scheme 3).

**Scheme 3:**
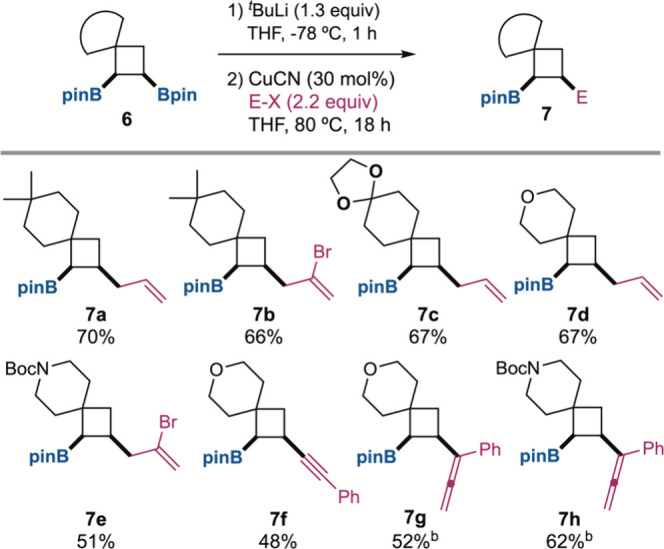
Scope of the Site-Selective Copper-Catalyzed Cross-Coupling of 1,2-Bis­(boryl)­spirocyclobutanes^a^

a 0.15–0.35 mmol scale. Isolated yields. >20:1 dr in all cases unless otherwise stated.
b 5 h of reaction time.

With the optimal conditions in hand, we examined the scope of this site-selective transformation. Pleasingly, we found that various 1,2-bis­(boryl)­spirocyclobutanes (**7a**-**d**) could be efficiently allylated under these conditions, even those bearing a more sensitive Boc-protected amine (**7e**). Also, an alkynyl bromide (**7f**) and propargyl tosylates (**7g**-**h**) were suitable electrophiles for this transformation.

All of the products prepared through selective copper-catalyzed cross-coupling contain an additional boryl moiety that can be engaged in further C–B bond functionalizations (Scheme 4). For example, Matteson homologation of compounds **7g**, **4c**, and **2c** afforded elongated boronates **8–10** in moderate to good yields. The oxidation/esterification protocol of **2a** and **7c** yielded cyclopropanol derivatives **11** and **12** in a good overall yield. Moreover, vinyl allyl cyclopropanes **13** and **14** were prepared by starting from compounds **2a** and **7b** using Zweifel olefination conditions.

**Scheme 4:**
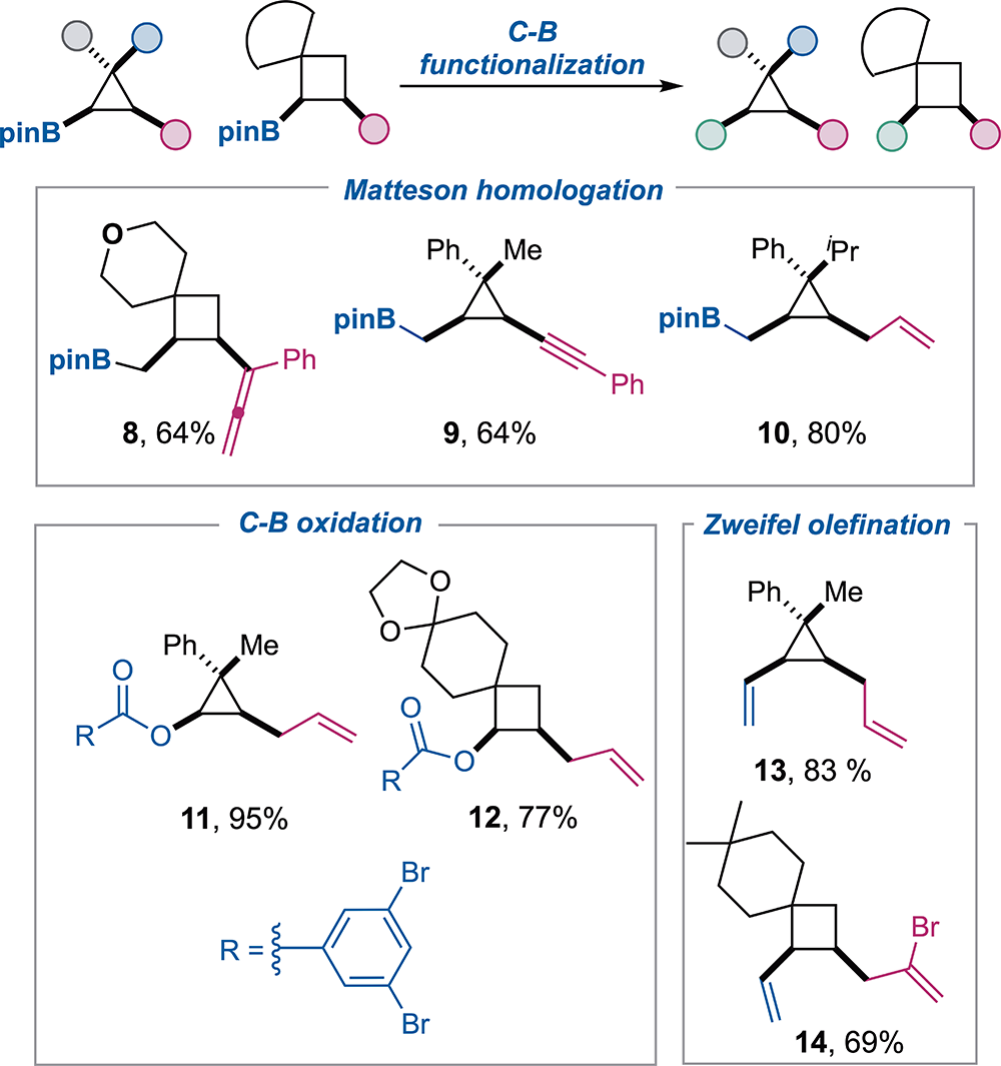
Functionalization of the Remaining Boryl Unit^a^

a Matteson homologation: CH_2_BrCl/*^n^* BuLi, −78 °C to rt, Et_2_O, 3 h. C–B oxidation: 1) H_2_O_2_/NaOH, THF/H_2_O; 2) 3,5-Br_2_–C_6_H_3_–COOH, EDC, DMAP, DCM. Zweifel olefination: CH_2_CHMgBr, I_2_/NaOMe, THF, 0 °C to rt.

In summary, we have demonstrated that 1,2-bis­(boryl)­cyclopropanes can participate in copper-catalyzed cross-coupling reactions with an array of electrophiles using a simple alkoxide base as an activator. On the other hand, we describe the site-selective activation of 1,2-bis­(boryl)­spirocyclobutanes and subsequent copper-catalyzed cross-coupling. This study showcases that substituents other than methyl can be conveniently included in the selective copper-catalyzed cross-coupling of vicinal bis­(boryl) compounds. This methodology allows for access to different substituted cyclopropanes with full control of the diastereoselectivity that otherwise would be challenging to prepare. In addition, we have performed the site-selective activation of 1,2-bis­(boryl)­spirocyclobutanes, expanding the applicability of the copper-catalyzed cross coupling to a library of sp^3^-rich molecules. We believe that this study will be of interest to the synthetic and medicinal chemistry communities.

## Supplementary Material



## Data Availability

The data underlying this study are available in the published article and its Supporting Information.

## Notes

General procedures, product characterization, copies of NMR spectra (PDF)
