# Supplementary material for: Selective Copper-Catalyzed Cross-Coupling of Cyclopropyl and Cyclobutyl 1,2-Bis(boronates)
Source: Org Lett. 2026 Jun 3;28(23):7153–7. doi: 10.1021/acs.orglett.6c01560 (PMC13270634; doi:10.1021/acs.orglett.6c01560)

Selective Copper-Catalyzed Cross-Coupling of Cyclopropyl and Cyclobutyl 1,2-Bis(boronates)

Marina Velado,[b] Javier Teresa,[a] Roberto Fernández de la Pradilla,[b] Alma Viso*[b] and Mariola Tortosa*[a]

[a]Organic Chemistry Department, Universidad Autónoma de Madrid (UAM), 28049 Madrid, Spain.

[b] Instituto de Química Orgánica General (IQOG), CSIC, Juan de la Cierva 3, 28006 Madrid, Spain.

[c]Institute for Advanced Research in Chemical Sciences (IAdChem), Universidad Autónoma de Madrid, Madrid 28049, Spain.

Mariola Tortosa: mariola.tortosa@uam.es

Alma Viso: almaviso@iqog.csic.es

Contents

General Experimental Details 3

Synthesis of Electrophiles 3

General Procedure for the Diboration of Cyclopropenes and Cyclobutenes 6

General Procedure for Boron to Copper Transmetalation of Bisboronates 7

A. Using LiOMe/CuL and carbon-centered electrophiles. 7

B. Using ^{t}BuLi/CuCN and carbon-centered electrophiles. 7

C. Using LiOMe/CuCN and nitrogen-centered electrophiles. 7

Optimization of Boron to Copper Transmetalation of Bisboronates 8

Table S1. Optimization of the allylation of cyclopropyl bisboronates 8

Table S2. Optimization of the propargylation/allenation of cyclopropyl bisboronates 9

Table S3. Optimization of the amination of cyclopropyl bisboronates 10

Table S4. Attempts of asymmetric allylation of cyclopropyl bisboronates 11

Table S5. Optimization of the allylation/amination of cyclobutyl bisboronates 12

Procedure for scale-up of 2e 12

Characterization Data 13

Allylation of cyclopropyl bisboronates 13

Propargylation/alkynylation of cyclopropyl bisboronates 20

Amination of cyclopropyl bisboronates 25

Functionalization of spirocyclobutyl bisboronates 27

General Procedure for Matteson Homologation 30

General Procedure for Oxidation and Benzoylation 31

General Procedure for Zweifel Olefination 32

References 34

Proofs of stereochemistry 35

NMR data 38

Allylation of cyclopropyl bisboronates 38

Propargylation/alkynylation of cyclopropyl bisboronates 64

Amination of cyclopropyl bisboronates 85

Functionalization of spirocyclobutyl bisboronates 95

C-B bond functionalization 107

General Experimental Details

Tetrahydrofuran, toluene and dichloromethane were purified by passing through a Pure Solv™ column drying system from Innovative Technology, Inc. Anhydrous MeOH and DMSO were purchased from VWR and stored over molecular sieves. NMR spectra were acquired on a Bruker Advance 300 MHz spectrometer, Varian INOVA-400, Varian MERCURY-400 or Varian INOVA-500 MHz (^{1}H NMR), at 75, 100 or 125 MHz (^{13}C NMR), at 128.4 (^{11}B NMR) and 376 MHz (^{19}F NMR). Chemical shifts (δ) are reported in ppm relative to residual solvent signals. ^{13}C NMR and ^{19}F spectra were acquired on a broad band decoupled mode. Structural assignments were made with additional information from gCOSY, gHSQC, and gHMBC experiments. The following abbreviations are used to describe peak patterns when appropriate: s (singlet), d (doublet), t (triplet), q (quartet), quint (quintet), m (multiplet), br (broad). Analytical thin layer chromatography (TLC) was performed using pre-coated aluminum-backed plates (Merck Kieselgel 60 F254) and visualized by ultraviolet irradiation and phosphomolybdic acid dip, potassium permanganate dip or cerium ammonium molybdate dip. Flash column chromatography (FC) was performed using silica gel Merck-60 (230-400 mesh) or Florisil (100-200 Mesh). High Resolution Mass Spectrometry (HRMS) were registered in an Agilent 6500 Accurate Mass Q-TOF (ESI/APCI). Melting points were determined on a Koffler block. Commercially available substrates were purchased from Aldrich, BDL Pharmatech, TCI and Fluorochem.

Synthesis of Electrophiles

The electrophiles employed were either commercially available or known compounds and were synthesized using reported procedures.

Synthesis of Allyl 4-methylbenzenesulfonate[1]

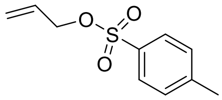

To a suspension of sodium hydride (1.5 equiv, washed with hexane) in Et_2O (5 mL x mmol of alcohol) under an atmosphere of argon was added (10 mmol, 1 equiv) of allyl alcohol at room temperature. Then, the reaction mixture was cooled to 0 ^{o}C and a solution of TsCl (1 equiv) in Et_2O (1 mL x mmol) was added. After the addition was complete the reaction mixture was warmed to 25 ^{o}C and allowed to proceed until disappearance of the starting material. A saturated solution of ammonium chloride was added and the mixture was extracted with Et_2O (x3), dried ($MgSO_4$), filtered, and concentrated under reduced pressure to furnish allyl 4-methylbenzenesulfonate after chromatography (silica gel; EtOAc/Hexane, 10-20%) in 74% yield (1.57 g, 7.4 mmol) as a clear oil. The data for the compound was consistent with reported values.[1] R_f = 0.30 (10% EtOAc/Hexane). ^{1}H NMR (300 MHz, $CDCl_3$) δ 7.79 (d, *J* = 8.3 Hz, 2H), 7.35 (d, *J* = 8.0 Hz, 2H), 5.82 (ddt, *J* = 17.2, 10.3, 5.9 Hz, 1H), 5.35 – 5.22 (m, 2H), 4.53 (dt, *J* = 6.0, 1.3 Hz, 2H), 2.45 (s, 3H).

Preparation of propargylic tosylates. A solution of propargyl alcohol (1 equiv), dry Et_2O (1.5 mL x mmol of alcohol) and TsCl (1.2 equiv) was cooled to 0 ˚C (ice bath). Ground KOH (1.2 equiv) was added to the solution and stirred at 0 ˚C for 1-3 h until disappearance of the starting alcohol. Then, the reaction was quenched by pouring water and the products were extracted with Et_2O (x3). The organic layer was washed with brine, dried over Na_2SO_4 and filtered. The filtrate was concentrated under reduced pressure. The residue was purified by column chromatography (silica gel; EtOAc/Hexane, 10-25%) to afford the corresponding tosylates. The data of the compounds were consistent with reported values.

3-Phenylprop-2-yn-1-yl 4-methylbenzenesulfonate.[2]

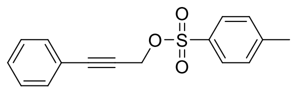

Yield 85% (2.54 mmol, 727 mg). R_f = 0.40 (20% EtOAc/Hexane). ^{1}H NMR (300 MHz, $CDCl_3$) δ 7.86 (d, J = 8.4 Hz, 1H), 7.33 – 7.26 (m, 8H), 4.95 (s, 2H), 2.39 (s, 3H).

But-2-yn-1-yl 4-methylbenzenesulfonate.[3]

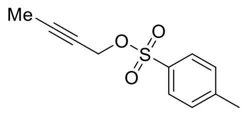

Yield 61% (1.22 mmol, 274 mg). R_f = 0.20 (20% EtOAc/Hexane). ^{1}H NMR (300 MHz, $CDCl_3$) δ 7.81 (d, J = 8.6 Hz, 2H), 7.34 (d, J = 8.0 Hz, 2H), 4.67 (s, 2H), 2.45 (s, 3H), 1.72 (s, 3H).

4-(4-nitrophenoxy)but-2-yn-1-yl 4-methylbenzenesulfonate.

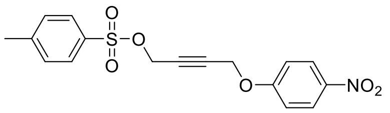

Yield 72% (1 mmol, 361 mg). ^{1}H NMR (400 MHz, $CDCl_3$) δ 8.20 (d, J = 9.2 Hz, 2H), 7.80 (d, J = 8.4 Hz, 2H), 7.41 – 7.29 (m, 2H), 6.96 (d, J = 9.3 Hz, 2H), 4.78 – 4.65 (m, 4H), 2.44 (s, 3H). ^{13}C NMR (101 MHz, $CDCl_3$) δ 162.3, 145.5, 133.1, 130.0, 128.3, 126.0, 115.0, 82.8, 80.6, 57.4, 56.3, 21.8.

Synthesis of alkynyl bromides. To a solution of *N*-bromosuccinimide (1.2 equiv) and alkyne (1 equiv) in acetone (10 mL x mmol of alkyne), silver nitrate (0.1 equiv) was added under an argon atmosphere. The reaction mixture was stirred at room temperature until disappearance of the starting alkyne (18 h) and subsequently evaporated to remove all volatiles. The crude mixture was dissolved in pentane and filtered through silica gel and concentrated to give a colorless liquid. The data of the compounds were consistent with reported values.[4]

(Bromoethynyl)triisopropylsilane.

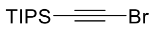

Yield 78% (0.78 mmol, 204 mg). R_f = 0.90 (Pentane). ^{1}H NMR (300 MHz, $CDCl_3$) δ 1.07 (s, 21H).

1-Bromooct-1-yne.

Yield 70% (0.7 mmol, 132 mg). R_f = 0.90 (Pentane). ^{1}H NMR (300 MHz, $CDCl_3$) δ 2.20 (t, *J* = 7.0 Hz, 2H), 1.55 – 1.46 (m, 2H), 1.42 – 1.25 (m, 6H), 0.89 (t, *J* = 7.1 Hz, 3H).

(Bromoethynyl)benzene.

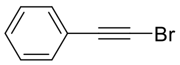

Yield 88% (4.4 mmol, 797 mg). R_f = 0.9 (Pentane). ^{1}H NMR (300 MHz, $CDCl_3$) δ 7.45 –7.43 (m, 2H), 7.35 – 7.31 (m, 3H).

Synthesis of N-(benzoyloxy)amines. A suspension of benzoyl peroxide (2.0 equiv) and Cs_2CO_3 (3.0 equiv) in CH_2Cl_2 (5 mL x mmol of benzoyl peroxide) was stirred for 2 h at room temperature. Then, a solution of amine (1.0 equiv) in CH_2Cl_2 (4 mL x mmol of amine) was added and the mixture was stirred until disappearance of the starting material (14 h). Then water was added to the reaction mixture and it was stirred for 5 min and extracted with CH_2Cl_2. The organic layer was washed with brine, dried over Na_2SO_4 and concentrated to afford a crude product that was purified by column chromatography (silica gel; CH_2Cl_2/Hexane or EtOAc/Hexane) to yield pure *N,N*-(benzoyloxy)amines. Analytical data were in good agreement with data reported in the literature.[5]

***O*-Benzoyl-*N,N*-dibenzylhydroxylamine.**

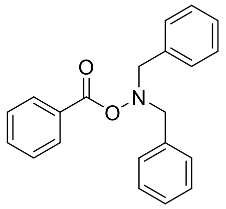

Yield 92% (2.76 mmol, 876 mg). R_f = 0.40 (40% EtOAc/Hexane). ^{1}H NMR (300 MHz, $CDCl_3$) δ 8.04 – 7.97 (m, 6H), 7.61 – 7.54 (m, 3H), 7.47 – 7.29 (m, 6H), 4.28 (d, *J* = 6.4 Hz, 4H).

Morpholino benzoate.

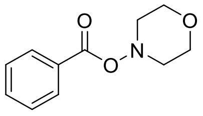

Yield 81% (2.43 mmol, 504 mg). R_f = 0.20 (40% CH_2Cl_2/Hexane). ^{1}H NMR (300 MHz, $CDCl_3$) δ 8.02 (d, *J* = 8.5 Hz, 2H), 7.61 – 7.55 (m, 1H), 7.47 – 7.42 (m, 2H), 4.04 – 3.82 (m, 4H), 3.45 (dm, *J* = 9.4 Hz, 2H), 3.07 –2.98 (m, 2H).

***N,N*-Diallyl-*O*-benzoylhydroxylamine.**

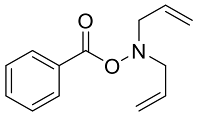

Yield 79% (2.37 mmol, 515 mg). R_f = 0.20 (80% CH_2Cl_2/Hexane). ^{1}H NMR (300 MHz, $CDCl_3$) δ 7.97 (d, *J* = 6.6 Hz, 2H), 7.58 – 7.52 (m, 1H), 7.44 – 7.39 (m, 2H), 6.08-5.95 (m, 2H), 5.29 – 5.16 (m, 4H), 3.68 – 3.64 (d, *J* = 6.6 Hz, 4H).

Synthesis of (3-bromoprop-1-en-2-yl)benzene. To a solution of prop-1-en-2-yl benzene (1.0 equiv) in THF (3.0 mL x mmol), *N*-bromosuccinimide (2.2 equiv) and TsOH·H_2O (0.1 equiv) were added and the solution was refluxed at 100 °C in an oil bath for 4 h. The reaction mixture was cooled to ambient temperature and was diluted with Et_2O. The organic phase was washed with H_2O (×3), dried over anhydrous Na_2SO_4 and concentrated under reduced pressure to obtain a yellow oil. Purification by column chromatography over silica gel using 100% hexanes as eluent afforded the title compound as a colorless oil. Analytical data were in good agreement with data reported in the literature.[6] Yield 48% (1.44 mmol, 284 mg). R_f = 0.80 (Hexane). ^{1}H NMR (300 MHz, $CDCl_3$) δ 7.52 – 7.31 (m, 5H), 5.57 (s, 1H), 5.51 (s, 1H), 4.40 (s, 2H).

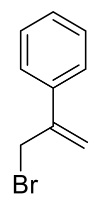

General Procedure for the Diboration of Cyclopropenes and Cyclobutenes

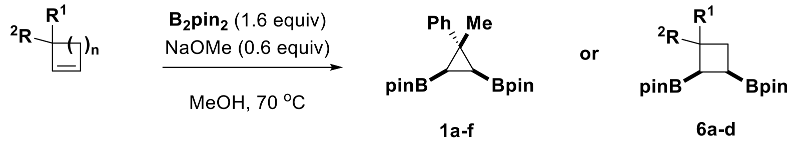

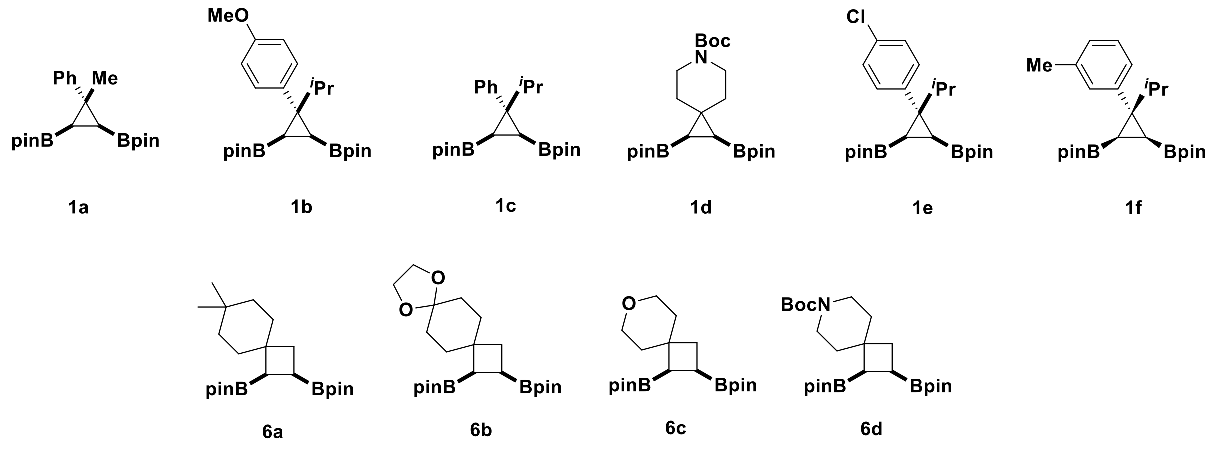

An oven-dried round-bottomed flask was charged with sodium methoxide (0.6 equiv) and bis(pinacolato)diboron (1.6 equiv) (both reagents were previously dried separately under vacuum for 30 min). After being sealed with a septum, the flask was connected to an argon-vacuum line and was evacuated and then backfilled with argon (x3). Anhydrous MeOH was bubbled with argon and added (0.5 mL x 0.2 mmol of cycloalkene). Then, a solution of cyclopropene or cyclobutene (1 equiv) in MeOH (0.5 mL x 0.2 mmol) was added and the resulting mixture was stirred at 70 °C in an oil bath for 16 h. After cooling to room temperature, the solvent was removed, and H_2O was added. Then, EtOAc was added, and the mixture was extracted with EtOAc (x3). The combined organic layers were dried ($MgSO_4$) and concentrated under reduced pressure. The crude mixture was suspended in 2-3 mL of 3:1, MeOH:H_2O and was evaporated under vacuum to remove pinacol byproducts. This procedure was repeated 6-8 times

(approx.) and then the crude product was purified by a rapid flash column chromatography on silica gel, Florisil and/or by crystallization from $MeOH/H_2O$ or MeOH. (CAUTION: bisboronates partially decompose on silica gel especially on the TLC plate to give a new spot that does not elute). Compounds **1a-f**[7] and **6a-d**[8] were previously reported.

General Procedure for Boron to Copper Transmetalation of Bisboronates

A. Using LiOMe/CuL and carbon-centered electrophiles.

In an oven-dried round bottomed flask, CuCN or CuCl (5% - 20 mol% depending on the electrophile), LiOMe (3 equiv), electrophile (1.5 equiv) and bis-boronate (1 equiv) were dissolved in THF (0.5 M). The reaction mixture was stirred at 60 ^oC in an oil bath until full conversion was observed by TLC (2 – 16 h). The reaction mixture was filtered through a pad of silica gel and concentrated under vacuum. The crude product was purified by flash column chromatography on silica gel or Florisil.

B. Using ^{t}BuLi/CuCN and carbon-centered electrophiles.

In an oven-dried round bottomed flask, bis-boronate (1 equiv) was dissolved in THF (3.5 mL x mmol of bis-boronate). The solution was cooled to -78 ^oC (dry ice/acetone) and *tert*-butyllithium (1.3 equiv, 1.7 M in heptane) was added dropwise to the mixture. Then, the reaction mixture was stirred while warming to room temperature for 30 min. After this time, a suspension containing the CuCN (20 mol%) in THF (3.5 mL x mmol of bis-boronate) was added, followed by the addition of the electrophile (2.2 equiv) in THF (3.5 mL x mmol of bis-boronate). The reaction mixture was stirred at 80 ^oC in an oil bath until total disappearance of starting material was observed by TLC (4 – 50 h). Then, MeOH (0.5 mL/mmol) was added while stirring and the mixture was filtered through a pad of silica gel. The crude product was purified by flash column chromatography on silica gel.

C. Using LiOMe/CuCN and nitrogen-centered electrophiles.

In an oven-dried round bottomed flask, CuCN (20 mol%), LiOMe (2.7 equiv), CsF (2.7 equiv), $PhCH=CH_2$ (1 equiv), electrophile (2.7 equiv) and bis-boronate (1 equiv) were dissolved in THF (2 mL x mmol of bis-boronate). The reaction mixture was stirred at 60 ^oC in an oil bath until full conversion was observed by TLC (4 – 6 h). The reaction mixture was filtered through a pad of silica gel and concentrated under vacuum. The crude product was purified by flash column chromatography on Florisil.

Optimization of Boron to Copper Transmetalation of Bisboronates

Table S1. Optimization of the allylation of cyclopropyl bisboronates

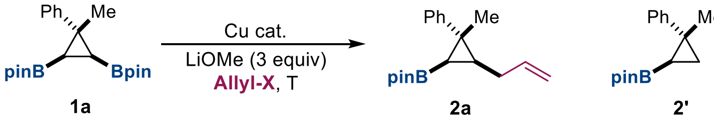

entry	base/T	CuX	additives	E-X/T	t	2a[a] (Yield%)	2'[a] (Yield%)	1a[a]
1	*t*-BuLi 1.3 equiv /-78 °C	CuCN 20 mol%	-	allyl-Br 1.5 equiv / 80 °C	16 h	50	50	-
2	LiOMe 3 equiv / 60 °C	CuCN 20 mol%	-	allyl-Br 1.5 equiv / 60 °C	3 h	98 (92%)[b]	2	-
**3**	LiOMe 3 equiv / 40 °C	CuCN 10 mol%	-	allyl-Br 1.5 equiv / 40 °C	24 h	98	2	-
**4**	**LiOMe 3 equiv / rt**	**CuCN 5 mol%**	**-**	**allyl-Br 1.5 equiv / rt**	**24 h**	**98 (90%)**	**2**	**-**
5	LiOMe 3 equiv / rt	CuCN 5 mol%	CsF 3 equiv $PhCH=CH_2$ 1 equiv	allyl-Br 1.5 equiv / rt	24 h	16	-	84
**6**	**LiOMe 3 equiv / rt**	**CuCl 5 mol%**	**-**	**allyl-Br 1.5 equiv / rt**	**24 h**	**95 (70%)**	**5**	**-**
**7**	**LiOMe 3 equiv / rt**	**CuCl 5 mol%**	**-**	**allyl-I 1.5 equiv / rt**	**24 h**	**95 (80%)**	**5**	**-**
**8**	**LiOMe 3 equiv / rt**	**CuCN 5 mol%**	**-**	**allyl-OTs 1.5 equiv / rt**	**24 h**	**99 (83%)**	**1**	
9	LiOMe 3 equiv / rt	CuCl 5 mol%	-	allyl-OTs 1.5equiv / rt	48 h	-	-	100
10	LiOMe 3 equiv / rt	CuCl 5 mol%		allyl-Cl 1.5equiv / rt	24 h	47 (39%)	53	-

[a] The ratio of compounds was measured in the ^{1}H NMR of the reaction crude.
[b] From 0.53 mmol (205 mg) of starting material.

Table S2. Optimization of the propargylation/allenation of cyclopropyl bisboronates

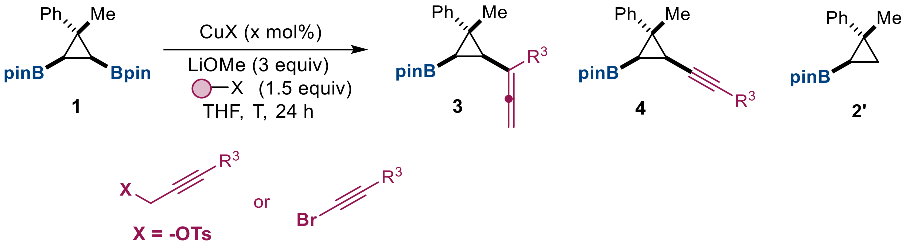

entry	base/T	CuX	E-X	t	3[a] (Yield%)	4[a] (Yield%)	2'[a] (Yield%)
1	LiOMe 3 equiv / 60 °C	CuCN 20 mol%	TIPS-CC-Br 1.5 equiv	4 h		73 (55%)[b]	27
2	LiOMe 3 equiv / 60 °C	CuCN 20 mol%	TIPS-CC-Br 1.5 equiv	4 h		73 (60%)[c]	27
**3**	**LiOMe 3 equiv / 60 °C**	**CuCN 20 mol%**	**C_6H_{13}-CC-Br 1.5 equiv**	**4 h**		**84 (49%)[b]**	**16**
4	LiOMe 3 equiv / rt	CuCl 5 mol%	C_6H_{13}-CC-Br 1.5 equiv	24 h		75	25
5	LiOMe 3 equiv / 60 °C	CuCN 20 mol%	Ph-CC-Br 1.5 equiv	4 h		95 (47%)[b]	5
6[d]	LiOMe 3 equiv / 60 °C	CuCN 20 mol%	Ph-CC-Br 1.5 equiv	4 h		93 (69%)[c]	7
**7**	**LiOMe 3 equiv / rt**	**CuCl 5 mol%**	**Ph-CC-Br 1.5 equiv**	**24 h**		**92 (60%)[c]**	**8**
8	LiOMe 3 equiv / 60 °C	CuCN 20 mol%	Me-CC-CH_2Br 1.5 equiv	4 h	95 (55%)[b]		5
9	LiOMe 3 equiv / rt	CuCl 5 mol%	Me-CC-CH_2Br 1.5 equiv	40 h	85 (39%)[b]		15
10	LiOMe 3 equiv / 60 °C	CuCN 20 mol%	Me-CC-CH_2OTs 1.5 equiv	4 h	98 (81)[c]		2
11	LiOMe 3 equiv / 60 °C	CuCN 20 mol%	Ph-CC-CH_2Cl 1.5 equiv	2 h	25		75
12	LiOMe 3 equiv / 60 °C	CuCN 20 mol%	Ph-CC-CH_2OTs 1.5 equiv	2 h	95 (69%)[c]		5
**13[e]**	**LiOMe 3 equiv / rt**	**CuCN 5 mol%**	**Ph-CC-CH_2OTs 1.5 equiv**	**20 h**	**95 (60%)[b]**		**5**

[a] The ratio of compounds was measured in the ^{1}H NMR of the reaction crude.

[b] Silica gel was used for purification.

[c] Florisil was used for purification.

[d] 0.3 mmol (115 mg) of starting material was used.

[e] 0.81 mmol (310 mg) of starting material was used.

Table S3. Optimization of the amination of cyclopropyl bisboronates

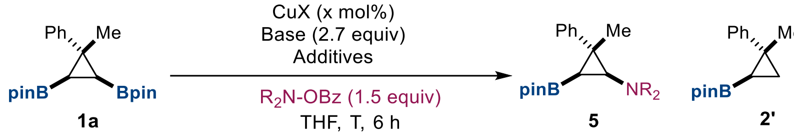

entry	base/T	CuX	additives	E-X/T	t	5[a,b] (Yield%)	2'[a] (Yield%)	1[a]
**1**	LiOMe 2.7 equiv / 60 °C	CuCN 20 mol%	CsF 2.7 equiv $PhCH{=}CH_2$ 1 equiv	Bn_2N-OCOPh 2.7 equiv / 60 °C	4 h	93 (45%)[b]	7	-
**2[c]**	**LiOMe 2.7 equiv / 60 °C**	**CuCN 20 mol%**	**CsF 2.7 equiv $PhCH{=}CH_2$ 1 equiv**	**Bn_2N-OCOPh 2.7 equiv / 60 °C**	**4 h**	**93% (75%)[d]**	**7**	**-**
3	LiOMe 2.7 equiv / 60 °C	CuCN 20 mol%	CsF 2.7 equiv $PhCH{=}CH_2$ 1 equiv	BnNH-OCOPh 2.7 equiv / 60 °C	4 h	-	10	90
4	LiOMe 2.7 equiv / 60 °C	CuCN 20 mol%	CsF 2.7 equiv $PhCH{=}CH_2$ 1 equiv	MeNTs-OCOPh[9] 2.7 equiv/ 60 °C	4 h	-	-	100
**5**	LiOMe 2.7 equiv / 60 °C	CuCN 20 mol%	CsF 2.7 equiv $PhCH{=}CH_2$ 1 equiv	$Ph_2{=}N$-O-CO_2Me[10] 2.7 equiv/ 60 °C	12 h	-	100	-
**6**	LiOMe 2.7 equiv / 60 °C	CuCN 20 mol%	CsF 2.7 equiv $PhCH{=}CH_2$ 1 equiv	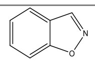 2.7 equiv / 60 °C	12 h	-	-	100
**7**	LiOMe 2.7 equiv / 60 °C	CuCN 20 mol%	CsF 2.7 equiv PhCH=CH2 1 equiv	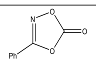 2.7 equiv[11] / 60 °C	48 h	-	74	26
**8**	LiOMe 2.7 equiv / 60 °C	CuCN 20 mol%	CsF 2.7 equiv PhCH=CH2 1 equiv	morfolino-OCOPh 2.7 equiv / 60 °C	24	75	-	25
**9**	**LiO^{t}Bu 2.7 equiv / 60 °C**	**CuCN 20 mol%**	**CsF 2.7 equiv PhCH=CH2 1 equiv**	**morpholino-OCOPh 2.7 equiv / 60 °C**	**7 h**	**98 (62%)[d]**	**-**	**2**

[a] The ratio of compounds was measured in the ^{1}H NMR of the reaction crude.

[b] Silica gel was used for purification.

[c] From 0.40 mmol (154 mg) of starting material.

[d] Florisil was used for purification.

Table S4. Attempts of asymmetric allylation of cyclopropyl bisboronates

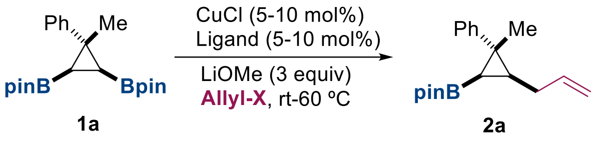

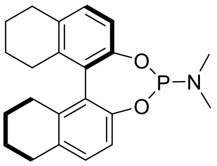

89%
50:50

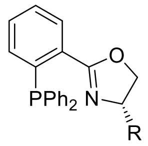

R = ^{t}Bu, 62%, 50:50
R = ^{i}Pr, 73%, 50:50
R = Ph, 38%, 50:50

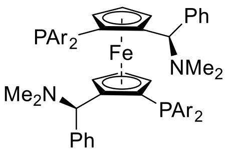

Ar = -3,5-CF_3
78%
50:50

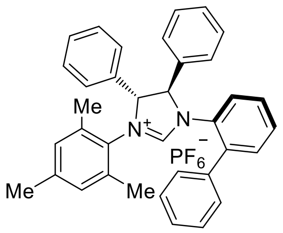

52%
50:50

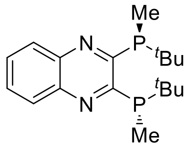

78%
50:50

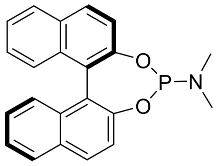

71%
50:50

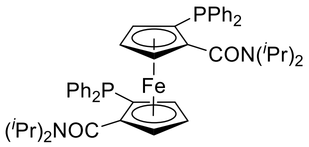

(*R*)-Japhaphos
97%
50:50

Table S5. Optimization of the allylation/amination of cyclobutyl bisboronates

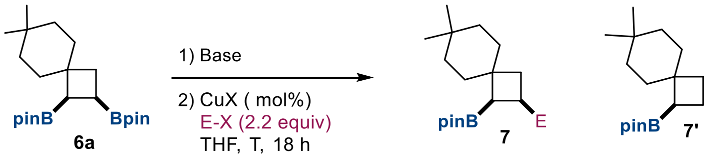

entry	base/T	CuX	additives	E-X/T	t	7 (Yield%)	7’ (Yield%)	6 (yield%)
**1**[a]	LiOMe 3 equiv / 80 ºC	CuCl 20 mol%	-	allyl-Br 2.2 equiv / 80 ºC	28 h	-	-	50%
**2**	^{t}BuLi 1.3 equiv / -78 ºC	CuCN 40 mol%	-	allyl-Br 2.2 equiv / 80 ºC	24 h	55%	10%	10%
**3**	^{t}BuLi 1.3 equiv / -78 ºC	CuCN 20 mol%	CsF 3 equiv PhCH=CH_2 1 equiv	allyl-Br 2.2 equiv / 80 ºC	24 h	-[b]	-[b]	-
**4**	**^{t}BuLi[c] 1.3 equiv / -78 ºC**	**CuCN 30 mol%**	**-**	**allyl-Br 2.2 equiv / 80 ºC**	**16 h**	**100 (70%)**	**-**	**-**
**5**	^{t}BuLi 1.3 equiv / -78 ºC	CuCN 20 mol%	CsF 3 equiv PhCH=CH_2 1 equiv	Bn_2NO-COPh 1.6 equiv / 80 ºC	24 h	-	-[d]	-[d]

[a] Sluggish and complex reaction. Starting material is the major compound in the ^{1}H NMR crude.
[b] Non regioselective mixture of allylation and deborylation products are tentatively present in this complex reaction mixture.
[c] The quality of the ^{t}BuLi solution is crucial for a good yield of **7**.
[d] Non regioselective mixture of deborylation products and starting material are tentatively present in this complex reaction mixture.

Procedure for scale-up of 2e

In an oven-dried round bottomed flask, CuCl (5 mol%, 0.005 mmol, 6.5 mg), LiOMe (3 equiv, 3.9 mmol, 148 mg), and bis-boronate **1a** (1 equiv, 1.3 mmol, 500 mg) and CH_2=C(Br)CH_2Br (1.5 equiv, 1.95 mmol, 0.24 mL) were dissolved in THF (13 mL). The reaction mixture was stirred 24 h at rt until full conversion was observed by TLC. The reaction mixture was filtered through a pad of silica gel and concentrated under vacuum. The crude product was purified by flash column chromatography (10-30% CH_2Cl_2/Hex) on silica gel to yield 87% of pure **2e** (425 mg, 1.13 mmol) as a colorless oil.

Characterization Data

Allylation of cyclopropyl bisboronates

(±)-2-((1*R*,2*S*,3*S*)-3-Allyl-2-methyl-2-phenylcyclopropyl)-4,4,5,5-tetramethyl-1,3,2-dioxaborolane (2a).

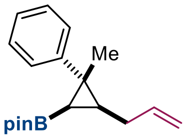

From bisboronate cyclopropane **1a** (77 mg, 0.20 mmol) following the general procedure GP.A (CuCl or CuCN 5 mol%, CH_2=$CHCH_2Br$, rt, 24 h), compound **2a** (42.0 mg, 0.14 mmol) was obtained in 70% yield as a colorless oil, after purification by flash column chromatography (silica gel; CH_2Cl_2/Hexane, 10-40%). Similarly, from **1a** (205 mg, 0.53 mmol) following the general procedure GP.A (CuCN, 20 mol%, CH_2=CH-CH_2Br, 60 °C, 3 h), compound **2a** (146 mg mg, 0.49 mmol) was obtained in 92% yield. From **1a** (77 mg, 0.20 mmol) following the general procedure GP.A (CuCN 5 mol%, CH_2=CH-CH_2I, rt, 24 h), compound **2a** (48 mg mg, 0.16 mmol) was obtained in 80% yield. From **1a** (77 mg, 0.20 mmol) following the general procedure GP.A (CuCN 5 mol%, CH_2=CH-CH_2OTs, rt, 24 h), compound **2a** (50 mg mg, 0.17 mmol) was obtained in 83% yield.

Data for **2a**: R_f 0.6 (10% Et_2O/Hexane). **^{1}H NMR (400 MHz, $CDCl_3$)** δ 7.29 – 7.22 (m, 4H), 7.15 – 7.06 (m, 1H), 5.96 (ddt, *J* = 16.7, 10.2, 6.3 Hz, 1H), 5.11 (m, 1H), 4.99 (m, 1H), 2.45 – 2.41 (m, 2H), 1.47 (s, 3H), 1.47 – 1.40 (m, 1H), 1.25 (s, 6H), 1.24 (s, 6H), 0.50 (d, *J* = 9.9 Hz, 1H). **2D-NOESY (400 MHz, $CDCl_3$)** crosspoint between: 1.47 – 1.40 (m, 1H)/ 0.50 (d, *J* = 9.9 Hz, 1H); 2.54 – 2.29 (m, 2H)/ 1.47 – 1.40 (m, 1H). **^{13}C NMR (101 MHz, $CDCl_3$)** δ 149.9, 139.0, 128.3, 127.4, 125.7, 114.6, 83.0, 31.0, 30.7, 25.1, 25.0, 18.2, 12.9. **^{11}B NMR (128 MHz, $CDCl_3$)** δ 32.6. **HRMS (ES)** *m/z* calcd for $C_{19}H_{28}BO_2$ $[M+H]^+$ 299.2180; found 299.2178.

(±)-2-((1*R*,2*S*,3S)-3-Allyl-2-(4-methoxyphenyl)-2-methylcyclopropyl)-4,4,5,5-tetramethyl-1,3,2-dioxaborolane (2b).

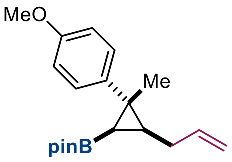

From bisboronate cyclopropane **1b** (92 mg, 0.22 mmol) following the general procedure GP.A (CuCl 5 mol%, CH_2=$CHCH_2Br$, rt, 24 h), compound **2b** (39.0 mg, 0.12 mmol) was obtained in 51% yield as a colorless oil, after purification by flash column chromatography (silica gel; CH_2Cl_2/Hexane, 20-60%).

Data for **2b**: R_f 0.60 (20% Et_2O/Hexane). **^{1}H NMR (400 MHz, $CDCl_3$)** δ 7.21 (d, *J* = 8.7 Hz, 2H), 6.80 (d, *J* = 8.7 Hz, 2H), 6.01 – 5.94 (m, 1H), 5.13 (m, 1H), 5.01 (m, 1H), 3.78 (s, 3H), 2.43 (m, 2H), 1.45 (s, 3H), 1.45 – 1.37 (m, 1H), 1.26 (2s, 6H+6H), 0.46 (d, *J* = 9.8 Hz, 1H). **^{13}C NMR (101 MHz, $CDCl_3$)** δ 157.6, 142.3, 139.0, 128.5, 114.5, 113.6, 82.9, 55.4, 31.0, 30.6, 30.5, 25.1, 25.0, 18.6, 12.6. **^{11}B NMR (128 MHz, $CDCl_3$)** δ 33.0. **HRMS (APCI)** *m/z* calcd for $C_{20}H_{30}BO_3$ $[M+H]^+$ 329.2286; found 329.2281.

(±)-2-((1*R*,2*R*,3*S*)-3-Allyl-2-isopropyl-2-phenylcyclopropyl)-4,4,5,5-tetramethyl-1,3,2-dioxaborolane (2c).

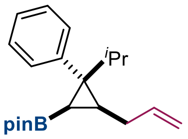

From bisboronate cyclopropane **1c** (82 mg, 0.20 mmol) following the general procedure GP.A [CuCl 5 mol%, CH_2=$CHCH_2Br$, rt, 24 h], compound **2c** (46.3 mg, 0.14 mmol) was obtained in 71% yield as a colorless oil, after purification by flash column chromatography (silica gel; CH_2Cl_2/Hexane, 10-30%).

Data for **2c**: R_f 0.60 (10% Et_2O/Hexane). **^{1}H NMR (400 MHz, $CDCl_3$)** δ 7.28 – 7.17 (m, 5H), 6.01 – 5.93 (m, 1H), 5.15 (m, 1H), 5.01 (m, 1H), 2.62 – 2.42 (m, 1H), 2.50 – 2.41 (m, 1H), 2.17 – 2.10 (m, 1H), 1.47 (td, *J* = 9.4, 5.6 Hz, 1H), 1.28 (s, 6H), 1.26 (s, 6H), 0.85 (d, *J* = 6.8 Hz, 6H), 0.43 (d, *J* = 9.7 Hz, 1H). **^{13}C NMR (101 MHz, $CDCl_3$)** δ 144.9, 139.3, 131.8, 127.2, 126.1, 114.5, 82.9, 42.7, 31.09, 30.13, 27.9, 25.2, 24.9, 21.1, 20.5, 11.6. **^{11}B NMR (128 MHz, $CDCl_3$)** δ 33.1. **HRMS (APCI)** *m/z* calcd for $C_{21}H_{32}BO_2$ $[M+H]^+$ 327.2494; found 327.2507.

(±)-*tert*-Butyl (1*S*,2*R*)-1-allyl-2-(4,4,5,5-tetramethyl-1,3,2-dioxaborolan-2-yl)-6-azaspiro[2.5] octane-6-carboxylate (2d).

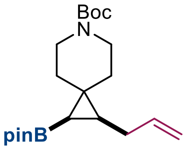

From bisboronate cyclopropane **1d** (93 mg, 0.20 mmol) following the general procedure GP.A (CuCl 5 mol%, CH_2=$CHCH_2Br$, rt, 24 h), compound **2d** (66.4 mg, 0.18 mmol) was obtained in 88% yield as a colorless oil, after purification by flash column chromatography (silica gel; CH_2Cl_2/Hexane, 10-30%).

Data for **2d**: R_f 0.60 (40% Et_2O/Hexane). **^{1}H NMR (400 MHz, $CDCl_3$)** δ 5.89 – 5.82 (m, 1H), 5.06 (m, 1H), 4.95 (m, 1H), 3.62 (m, 2H), 3.22 – 3.16 (m, 1H), 3.10 – 3.05 (m, 1H), 2.27 – 2.24 (m, 2H), 1.68 – 1.50 (m, 3H), 1.44 (s, 9H), 1.20 (s, 6H), 1.19 (s, 6H), 1.19 – 1.12 (m, 1H), 1.03 (dd, *J* = 9.6, 7.4 Hz, 1H), -0.11 (d, *J* = 9.4 Hz, 1H). **^{13}C NMR (101 MHz, $CDCl_3$, HSQC)** δ 155.1, 138.8, 114.4, 82.9, 79.3, 44.2, 39.3, 30.1, 29.6, 28.7, 27.3, 25.1, 24.8, 10.6. **^{11}B NMR (128 MHz, $CDCl_3$)** δ 32.6. **HRMS (ESI)** *m/z* calcd for $C_{21}H_{37}BNO_4$ $[M+H]^+$ 378.2814; found 378.2811.

(±)-2-((1*R*,2*S*,3*S*)-3-(2-Bromoallyl)-2-methyl-2-phenylcyclopropyl)-4,4,5,5-tetramethyl-1,3,2-dioxaborolane (2e).

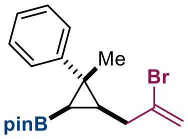

From bisboronate cyclopropane **1a** (77 mg, 0.20 mmol) following the general procedure GP.A [CuCl 5 mol%, CH_2=$C(Br)CH_2Br$, rt, 24 h], compound **2e** (60.0 mg, 0.16 mmol) was obtained in 87% yield as a colorless oil, after purification by flash column chromatography (silica gel; CH_2Cl_2/Hexane, 10-30%).

Data for **2e**: R_f 0.50 (40% CH_2Cl_2/Hexane). **^{1}H NMR (400 MHz, $CDCl_3$)** δ 7.32 – 7.24 (m, 4H), 7.17 – 7.15 (m, 1H), 5.72 (brs, 1H), 5.45 (brs, 1H), 2.86 – 2.84 (m, 2H), 1.64 (dt, *J* = 9.9, 7.1 Hz, 1H), 1.48 (s, 3H), 1.26 (s,

6H), 1.25 (s, 6H), 0.59 (d, *J* = 9.9 Hz, 1H). **^{13}C NMR (101 MHz, $CDCl_3$)** δ 149.2, 134.8, 128.3, 127.4, 125.9, 116.3, 83.2, 38.9, 31.2, 29.8, 25.1, 24.9, 18.3, 12.8. **^{11}B NMR (128 MHz, $CDCl_3$)** δ 32.8. **HRMS (APCI)** *m/z* calcd for $C_{19}H_{27}BBrO_2$ $[M+H]^+$ 377.1285; found 377.1293.

(±)-2-((1*R*,2*S*,3*S*)-3-(2-Bromoallyl)-2-(4-methoxyphenyl)-2-methylcyclopropyl)-4,4,5,5-tetramethyl-1,3,2-dioxaborolane (2f).

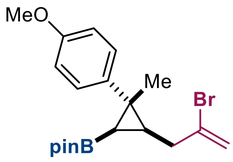

From bisboronate cyclopropane **1b** (83 mg, 0.20 mmol) following the general procedure GP.A [CuCl 5 mol%, $CH_2=C(Br)-CH_2Br$, rt, 24 h], compound **2f** (46.0 mg, 0.11 mmol) was obtained in 57% yield as a colorless oil, after purification by flash column chromatography (silica gel; Et_2O/Hexane, 2-5%).

Data for **2f**: R_f 0.45 (20% Et_2O-Hexane). **^{1}H NMR (400 MHz, $CDCl_3$)** δ 7.24 (d, *J* = 8.8 Hz, 2H), 6.81 (d, *J* = 8.8 Hz, 2H), 5.73 (s, 1H), 5.46 (s, 1H), 3.78 (s, 3H), 2.85 (m, 2H), 1.62 – 1.58 (m, 1H), 1.46 (s, 3H), 1.27 (s, 6H), 1.26 (s, 6H), 0.54 (d, *J* = 9.9 Hz, 1H). **^{13}C NMR (101 MHz, $CDCl_3$)** δ 157.8, 141.7, 134.9, 128.5, 116.3, 113.7, 83.2, 55.4, 38.9, 30.7, 29.73, 25.1, 24.9, 18.7. [note: the carbon attached to boron was not observed due to quadrupole broadening caused by the ^{11}B nucleus]. **^{11}B NMR (128 MHz, $CDCl_3$)** δ 32.8**.** **HRMS (APCI)** *m/z* calcd for $C_{20}H_{29}BBrO_3$ $[M+H]^+$ 407.1391; found 407.1388.

(±)-2-((1*R*,2*R*,3*S*)-3-(2-Bromoallyl)-2-isopropyl-2-phenylcyclopropyl)-4,4,5,5-tetramethyl-1,3,2-dioxaborolane (2g).

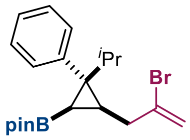

From bisboronate cyclopropane **1c** (82 mg, 0.20 mmol) following the general procedure GP.A [CuCl 5 mol%, $CH_2=C(Br)CH_2Br$, rt, 24 h], compound **2g** (74.5 mg, 0.18 mmol) was obtained in 92% yield as a colorless oil, after purification by flash column chromatography (silica gel; CH_2Cl_2/Hexane, 10-30%).

Data for **2g**: R_f 0.80 (10% Et_2O/Hexane). **^{1}H NMR (400 MHz, $CDCl_3$)** δ 7.28 – 7.19 (m, 5H), 5.75 (brs, 1H), 5.46 (brs, 1H), 3.01 (dd, *J* = 16.6, 4.3 Hz, 1H), 2.89 (dd, *J* = 17.2, 9.8 Hz, 1H), 2.12 (m, 1H), 1.69 (td, *J* = 9.7, 4.6 Hz, 1H), 1.29 (s, 6H), 1.26 (s, 6H), 0.85 (d, *J* = 7.1 Hz, 6H), 0.53 (d, *J* = 9.6 Hz, 1H). **^{13}C NMR (101 MHz, $CDCl_3$)** δ 144.2, 135.0, 131.5, 127.2, 126.2, 115.9, 83.0, 42.7, 37.8, 30.0, 27.9, 25.1, 24.7, 21.0, 20.3, 11.8. **^{11}B NMR (128 MHz, $CDCl_3$)** δ 32.7. **HRMS (APCI)** *m/z* calcd for $C_{21}H_{31}BBrO_2$ $[M+H]^+$ 405.1599; found 405.1611.

(±)-*tert*-Butyl (1*S*,2*R*)-1-(2-bromoallyl)-2-(4,4,5,5-tetramethyl-1,3,2-dioxaborolan-2-yl)-6-azaspiro [2.5]octane-6-carboxylate (2h).

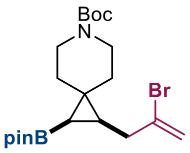

From bisboronate cyclopropane **1d** (93 mg, 0.20 mmol) following the general procedure GP.A [CuCl 5 mol%, CH_2=C(Br)CH_2Br, rt, 24 h], compound **2h** (58.4 mg, 0.13 mmol) was obtained in 64% yield as a colorless oil, after purification by flash column chromatography (silica gel; Et_2O/CH_2Cl_2, 1-5%).

Data for **2h**: R_f 0.55 (10% Et_2O/Hexane). **^{1}H NMR (400 MHz, $CDCl_3$)** δ 5.68 (s, 1H), 5.42 (s, 1H), 3.67 – 3.63 (m, 2H), 3.20 – 3.14 (m, 1H), 3.07 – 3.01 (m, 1H), 2.69 – 2.65 (m, 2H), 1.69 – 1.59 (m, 3H), 1.46 (s, 9H), 1.25-1.19 (m, 13H), 1.18 – 1.09 (m, 1H), –0.00 (d, J = 9.4 Hz, 1H). **^{13}C NMR (101 MHz, $CDCl_3$, HSQC)** δ 155.1, 134.7, 116.1, 83.2, 79.4, 44.0, 43.7, 39.1, 38.0, 28.94, 28.89, 28.6, 27.4, 25.2, 24.8, 10.7. **^{11}B NMR (128 MHz, $CDCl_3$)** δ 31.9. **HRMS (ESI)** *m/z* calcd for $C_{21}H_{36}BBrNO_4$ $[M+H]^+$ 458.1901; found 458.1898.

(±)-4,4,5,5-Tetramethyl-2-((1*R*,2*S*,3*S*)-2-methyl-3-(2-methylallyl)-2-phenylcyclopropyl)-1,3,2-dioxaborolane (2i).

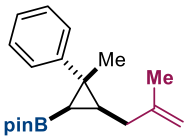

From bisboronate cyclopropane **1a** (77 mg, 0.20 mmol) following the general procedure GP.A [CuCl 5 mol%, CH_2=C(Me)CH_2Br, rt, 24 h], compound **2i** (48.0 mg, 0.15 mmol) was obtained in 77% yield as a colorless oil, after purification by flash column chromatography (silica gel; CH_2Cl_2/Hexane, 10-30%).

Data for **2i**: R_f 0.60 (10% Et_2O/Hexane). **^{1}H NMR (400 MHz, $CDCl_3$)** δ 7.29 – 7.22 (m, 4H), 7.15 – 7.11 (m, 1H), 4.82 (brs, 1H), 4.74 (brs, 1H), 2.39-2.36 (m, 2H), 1.79 (s, 3H), 1.54 – 1.49 (m, 1H), 1.47 (s, 3H), 1.24 (s, 6H), 1.23 (s, 6H), 0.54 (d, J = 10.3 Hz, 1H). **^{13}C NMR (101 MHz, $CDCl_3$)** δ 149.9, 146.5, 128.3, 127.2, 125.6, 109.9, 83.0, 34.5, 30.8, 30.2, 25.2, 24.9, 23.2, 18.1, 13.2. **^{11}B NMR (128 MHz, $CDCl_3$)** δ 32.9. **HRMS (ES)** *m/z* calcd for $C_{20}H_{30}BO_2$ $[M+H]^+$ 313.2337; found 313.2329.

(±)-4,4,5,5-Tetramethyl-2-((1*R*,2*S*,3*S*)-2-methyl-2-phenyl-3-(2-phenylallyl)cyclopropyl)-1,3,2-dioxaborolane (2j).

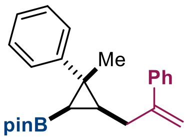

From bisboronate cyclopropane **1a** (77 mg, 0.20 mmol) following the general procedure GP.A [CuCl 5 mol%, CH_2=C(Ph)CH_2Br, rt, 24 h], compound **2j** (66.0 mg, 0.18 mmol) was obtained in 89% yield as a colorless oil, after purification by flash column chromatography (silica gel; CH_2Cl_2/Hexane, 10-30%).

Data for **2j**: R_f 0.50 (10% Et_2O/Hexane). **^{1}H NMR (400 MHz, $CDCl_3$)** δ 7.50 – 7.47 (m, 2H), 7.38 – 7.14 (m, 8H), 5.34 (brs, 1H), 5.25 (brs, 1H), 2.89 (m, 2H), 1.65 – 1.59 (m, 1H), 1.53 (s, 3H), 1.25 (s, 12H), 0.60 (d, J = 9.9 Hz, 1H). **^{13}C NMR (101 MHz, $CDCl_3$)** δ 149.8, 149.1, 142.4, 128.3, 128.2, 127.4, 127.4, 126.3, 125.7, 112.2, 83.1, 32.1, 31.3, 29.9, 25.2, 24.9, 18.3. [note: the carbon attached to boron was not observed due

to quadrupole broadening caused by the ^{11}B nucleus]. **^{11}B NMR (128 MHz, CDCl$_3$)** δ 33.2. **HRMS (ES)** *m/z* calcd for $C_{25}H_{32}BO_2$ $[M+H]^+$ 375.2494; found 375.2488.

(±)-2-((1*R*,2*S*,3*S*)-3-(2-(Bromomethyl)allyl)-2-methyl-2-phenylcyclopropyl)-4,4,5,5-tetramethyl-1,3,2-dioxaborolane (2k).

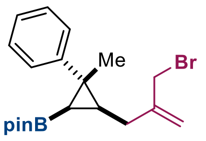

From bisboronate cyclopropane **1a** (77 mg, 0.20 mmol) following the general procedure GP.A [CuCl 5 mol%, 2.7 equiv of $CH_2=C(CH_2Br)_2$, rt, 24 h], compound **2k** (60.0 mg, 0.15 mmol) was obtained in 77% yield as a colorless oil, after purification by flash column chromatography (silica gel; CH_2Cl_2/Hexane, 10-30%). Alternatively, from bisboronate cyclopropane **1a** (38 mg, 0.10 mmol) following the general procedure GP.A [CuCN 20 mol%, 1.5 equiv $CH_2=C(CH_2Br)_2$, 60 °C, 3 h], after purification by flash column chromatography (silica gel; CH_2Cl_2/Hexane, 10-50%) compound **2k** (26.0 mg, 0.07 mmol) was obtained in 67% yield as a colorless oil and **2k'** as a mixture of diastereoisomers (8 mg, 0.014 mmol, 14% yield).

Data for **2k**: R_f 0.50 (50% CH_2Cl_2/Hexane). **^{1}H NMR (400 MHz, CDCl$_3$, COSY)** δ 7.29 – 7.22 (m, 4H), 7.16 – 7.13 (m, 1H), 5.20 (brs, 1H), 5.13 (brs, 1H), 4.03 (brs, 2H), 2.66 (dd, *J* = 16.8, 6.6 Hz, 1H), 2.54 (dd, *J* = 16.8, 7.7 Hz, 1H), 1.55 – 1.51 (m, 1H), 1.49 (s, 3H), 1.25 (s, 6H), 1.23 (s, 6H), 0.58 (d, *J* = 9.9 Hz, 1H). **2D-NOESY (400 MHz, CDCl$_3$)** crosspoint between: 0.58 (d, *J* = 9.9 Hz, 1H) / 1.51 (m, 1H); 1.49 (s, 3H) / 2.66 (dd, *J* = 16.8, 6.6 Hz, 1H); 1.49 (s, 3H) / 2.54 (dd, *J* = 16.8, 7.7 Hz, 1H). **^{13}C NMR (101 MHz, CDCl$_3$, HSQC, HMBC)** δ 149.5, 146.1, 128.3, 127.1, 125.8, 115.1, 83.1, 37.6, 30.8, 30.5, 29.5, 25.2, 24.9, 18.0, 13.1. **^{11}B NMR (128 MHz, CDCl$_3$)** δ 32.7. **HRMS (ES)** *m/z* calcd for $C_{20}H_{29}BBrO_2$ $[M+H]^+$ 391.1442; found 391.1411.

(±)-2,2'-((1*R*,1'*R*,2*S*,2'*S*,3*S*,3'*S*)-(2-Methylenepropane-1,3-diyl)bis(3-methyl-3-phenyl cyclopropane-2,1-diyl))bis(4,4,5,5-tetramethyl-1,3,2-dioxaborolane) (2k)'.

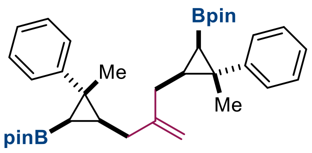

Data for **2k'** as a single diastereomer from an enriched sample. R_f 0.50 (70% CH_2Cl_2/Hexane). **^{1}H NMR (400 MHz, CDCl$_3$)** δ 7.38 – 6.96 (m, 10H), 4.93 (brs, 2H), 2.48 (m, 4H), 1.66 – 1.59 (m, 2H), 1.50 (s, 6H), 1.23 (s, 24H), 0.56 (d, *J* = 9.4 Hz, 2H). **^{13}C NMR (101 MHz, CDCl$_3$)** δ 150.6, 149.9, 128.2, 127.2, 125.6, 109.1, 83.0, 33.7, 31.0, 30.4, 25.2, 24.9, 18.0, 13.3. **^{11}B NMR (128 MHz, CDCl$_3$)** δ 32.0. **HRMS (APCI)** *m/z* calcd for $C_{36}H_{51}B_2O_4$ $[M+H]^+$ 569.3980; found 569.3997.

(±)-2-((1*R*,2*S*,3*R*)-3-((*S*)-Cyclohexane-2-en-1-yl)-2-methyl-2-phenylcyclopropyl)-4,4,5,5-tetramethyl-1,3,2-dioxaborolane (2l).

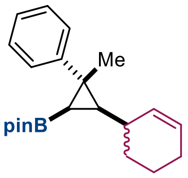

From bisboronate cyclopropane **1a** (38 mg, 0.10 mmol) following the general procedure GP.A [CuCN 20 mol%, 3-bromocyclohex-1-ene, 60 °C, 4 h], compound **2l** (dr 67:33, 25.7 mg, 0.08 mmol) was obtained in 76% yield as a colorless oil, after purification by flash column chromatography (silica gel; CH_2Cl_2/Hexane, 10-20%).

Data for **2l** from an enriched sample of the major isomer: R_f 0.70 (10% Et_2O/Hexane). **^{1}H NMR (400 MHz, CDCl$_3$, COSY)** δ 7.29 – 7.22 (m, 4H), 7.15 – 7.12 (m, 1H), 5.76 (m, 2H), 2.39 – 2.31 (m, 1H), 2.03 – 1.99 (m, 2H), 1.88 – 1.72 (m, 2H), 1.61 – 1.53 (m, 4H), 1.42 (m, 1H), 1.26 – 1.21 (m, 13 H), 0.44 (d, J = 9.8 Hz, 1H**).** **2D-NOESY (400 MHz, CDCl$_3$)** crosspoint between: 1.26-1.21 (m, 1 H) / 0.44 (d, J = 9.8 Hz, 1H); 1.53 (s, 3H) / 5.76 (s, 2H); 0.44 (d, J = 9.8 Hz, 1H) / 7.29 – 7.13 (m, 6H**).** **^{13}C NMR (101 MHz, CDCl$_3$, HSQC)** δ 150.2, 132.00, 128.2, 127.8, 127.3, 125.7, 82.9, 37.3, 32.9, 31.8, 29.5, 25.5, 25.1, 24.9, 21.4, 18.9. [note: the carbon attached to boron was not observed due to quadrupole broadening caused by the ^{11}B nucleus]. **^{11}B NMR (128 MHz, CDCl$_3$)** δ 33.3. **HRMS (APCI)** *m/z* calcd for $C_{22}H_{32}BO_2$ $[M+H]^+$ 339.2494; found 339.2493.

Additional examples

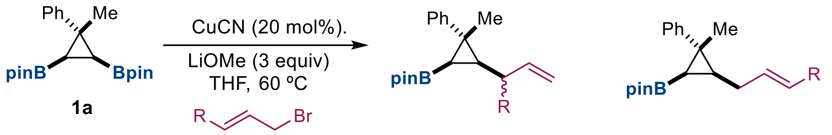

R = -Ph **2m** 80%	80 (79:21 dr)	20
R = -Me **2n** 84%	70 (65:35 dr)	30
R = -COOMe **2o** 83%	74 (50:50 dr)	26

(±)-4,4,5,5-Tetramethyl-2-((1*R*,2*S*,3*R*)-2-methyl-2-phenyl-3-((*S*)-1-phenylallyl)cyclopropyl)-1,3,2-dioxaborolane (2m)

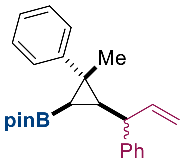

From bisboronate cyclopropane **1a** (38 mg, 0.10 mmol) following the general procedure GP.A [CuCN 20 mol%, cinnamyl bromide, 60 °C, 4 h], an 80:20 mixture of isomers [SN2':SN2] (29.9 mg, 0.08 mmol, 80% combined yield). Compound **2m** (dr 79:21) was obtained as a colorless oil, after purification by flash column chromatography (silica gel; CH_2Cl_2/Hexane, 10-20%).

Data for **2m**: R_f 0.65 (10% CH_2Cl_2/Hexane). **^{1}H NMR (400 MHz, CDCl$_3$)** δ 7.35 – 7.05 (m, 10H), 6.14 – 6.01 (m, 1H), 5.17 – 5.03 (m, 2H), 3.75 – 3.70 (m, 1H), 1.77 (dd, J = 11.1, 9.9 Hz, 1H), 1.60 (s, 3H major), 1.42 (s, 3H minor), 1.29 (s, 6H minor), 1.27 (s, 6H minor), 1.11 (s, 6H, major), 1.03 (s, 6H, major), 0.68 (d, J = 10.8 Hz, 1H, minor), 0.61 (d, J = 9.9 Hz, 1H, major). **^{13}C NMR (101 MHz, CDCl$_3$, HSQC)** δ 149.7, 144.6 (minor),

144.3, 142.9, 142.5 (minor), 128.5 (minor), 128.4, 128.3, 128.2, 127.6 (minor), 127.5, 126.3 (minor), 126.1, 125.83, 125.75 (minor), 114.1, 114.0 (minor), 83.16 (minor), 83.05, 46.3 (minor), 46.2, 36.0 (minor), 35.8, 32.5, 32.1 (minor), 25.3 (minor), 25.27, 24.9 (minor), 24.4, 19.0 (minor), 18.3 (minor), 12.5. **^{11}B NMR (128 MHz, $CDCl_3$)** δ 32.9. **HRMS (ESI)** *m/z* calcd for $C_{25}H_{35}BNO_2$ $[M+NH_4]^+$ 392.2760; found 392.2757.

(±)-2-((1*R*,2*S*,3*R*)-3-((*S*)-But-3-en-2-yl)-2-methyl-2-phenylcyclopropyl)-4,4,5,5-tetramethyl-1,3,2-dioxaborolane (2n)

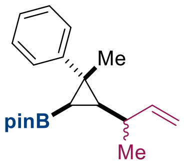

From bisboronate cyclopropane **1a** (38 mg, 0.10 mmol) following the general procedure GP.A [CuCN 20 mol%, crotyl bromide, 60 °C, 4 h], a 70:30 mixture of isomers [S_N2':S_N2] (26.2 mg, 0.08 mmol, 84% combined yield). Compound **2n** (dr 65:35) was obtained as a colorless oil, after purification by flash column chromatography (silica gel; CH_2Cl_2/Hexane, 10-20%).

Data for **2n**: R_f 0.60 (10% Et_2O/Hexane). **^{1}H NMR (300 MHz, $CDCl_3$)** δ 7.28 – 7.09 (m, 5H), 5.99 (ddd, *J* = 17.3, 10.2, 6.5 Hz, 1H), 5.07 (m, 1H), 4.99 (m, 1H), 2.53 – 2.45 (m, 1H), 1.49 (s, 3H), 1.27 – 1.19 (m, 13H), 1.10 (d, *J* = 6.7 Hz, 3H), 0.48 (d, *J* = 9.6 Hz, 1H). **^{13}C NMR (101 MHz, $CDCl_3$)** δ 149.9, 144.4, 128.2, 127.4, 125.7, 112.4, 83.0, 38.1, 34.9, 31.9, 25.1, 25.0, 20.1, 18.5. [note: the carbon attached to boron was not observed due to quadrupole broadening caused by the ^{11}B nucleus]. **^{11}B NMR (128 MHz, $CDCl_3$)** δ 33.3. **HRMS (APCI)** *m/z* calcd for $C_{20}H_{30}BO_2$ $[M+H]^+$ 313.2337; found 313.2337.

(±)-Methyl (*S*)-2-((1*R*,2*S*,3*R*)-2-methyl-2-phenyl-3-(4,4,5,5-tetramethyl-1,3,2-dioxaborolan-2-yl)cyclopropyl)but-3-enoate (2o)

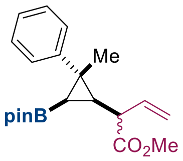

From bisboronate cyclopropane **1a** (20 mg, 0.05 mmol) following the general procedure GP.A [CuCN 20 mol%, methyl 4-bromocrotonate, 60 °C, 4 h], a 74:26 mixture of isomers [S_N2':S_N2] (14.8 mg, 0.04 mmol, 83% combined yield). Compound **2o** (dr 50:50) was obtained as a colorless oil, after purification by flash column chromatography (silica gel; CH_2Cl_2/Hexane, 10-20%).

Data for **2o**: R_f 0.70 (10% Et_2O-Hexane). **^{1}H NMR (500 MHz, $CDCl_3$)** δ 7.39 – 7.37 (m, 2H), 7.26 (m, 2H), 7.17 (m, 1H), 5.98 (ddd, *J* = 17.3, 10.3, 7.0 Hz, 1H), 5.28 (m, 1H), 5.16 (m, 1H), 3.79 (s, 3H), 3.53 – 3.49 (m, 1H), 1.76 (dd, *J* = 11.0, 9.8 Hz, 1H), 1.45 (s, 3H), 1.26 (s, 6H), 1.24 (s, 6H), 0.57 (d, *J* = 9.8 Hz, 1H). **2D-NOESY (400 MHz, $CDCl_3$)** crosspoint between: 0.57 (d, *J* = 9.8 Hz, 1H) / 1.76 (dd, *J* = 11.0, 9.8 Hz, 1H); 3.53 – 3.49 (m, 1H) / 1.45 (s, 3H). **^{13}C NMR (126 MHz, $CDCl_3$)** δ 174.8, 149.4, 136.2, 128.3, 128.2, 126.1, 116.9, 83.3, 52.1, 46.9, 32.2, 31.6, 25.3, 24.8, 19.2. [note: the carbon attached to boron was not observed due to

quadrupole broadening caused by the ^{11}B nucleus]. **^{11}B NMR (128 MHz, $CDCl_3$)** δ 33.2. **HRMS (ESI)** *m/z* calcd for $C_{21}H_{30}BO_4$ $[M+H]^+$ 357.2235; found 357.2240.

Propargylation/alkynylation of cyclopropyl bisboronates

(±)-2-((1*R*,2*S*,3*R*)-3-(Buta-2,3-dien-2-yl)-2-methyl-2-phenylcyclopropyl)-4,4,5,5-tetramethyl-1,3,2-dioxaborolane (3a).

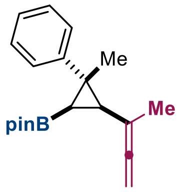

From bisboronate cyclopropane **1a** (38 mg, 0.10 mmol) following the general procedure GP.A [CuCN 20 mol%, but-2-yn-1-yl 4-methylbenzenesulfonate , 60 °C, 2 h], compound **3a** (25.1 mg, 0.08 mmol) was obtained in 83% yield as a colorless oil, after purification by flash column chromatography (silica gel; CH_2Cl_2/Hexane, 10-20%).

Data for **3a**: R_f 0.70 (10% Et_2O/Hexane). **^1H NMR (400 MHz, $CDCl_3$)** δ 7.29 – 7.26 (m, 4H), 7.26 – 7.14 (m, 1H), 4.70 – 4.67 (m, 2H), 1.98 – 1.93 (m, 1H), 1.83 (apt, *J* = 3.0 Hz, 3H), 1.53 (s, 3H), 1.28 (s, 6H), 1.26 (s, 6H), 0.63 (d, *J* = 10.2 Hz, 1H). **2D-NOESY (400 MHz, $CDCl_3$)** crosspoint between: 0.63 (d, *J* = 10.2 Hz, 1H) / 1.98 – 1.93 (m, 1H); 0.63 (d, *J* = 10.2 Hz, 1H) / 1.53 (s, 3H). **^{13}C NMR (101 MHz, $CDCl_3$, HSQC)** δ 208.8, 149.3, 128.3, 127.4, 125.8, 96.5, 83.0, 75.5, 34.3, 32.2, 25.6, 24.7, 20.2, 19.5. [note: the carbon attached to boron was not observed due to quadrupole broadening caused by the ^{11}B nucleus]. **^{11}B NMR (128 MHz, $CDCl_3$)** δ 32.9. **HRMS (ESI)** *m/z* calcd for $C_{20}H_{28}BO_2$ $[M+H]^+$ 311.2181; found 311.2179.

(±)-4,4,5,5-Tetramethyl-2-((1*R*,2*S*,3*S*)-2-methyl-2-phenyl-3-(1-phenylpropa-1,2-dien-1-yl)cyclopropyl)-1,3,2-dioxaborolane (3b).

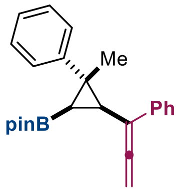

From bisboronate cyclopropane **1a** (310 mg, 0.81 mmol) following the general procedure GP.A [CuCN 5 mol%, 3-phenylprop-2-yn-1-yl 4-methylbenzenesulfonate (1.05 equiv), rt, 20 h], compound **3b** (256.3 mg, 0.69 mmol) was obtained in 85% yield as a colorless oil, after purification by flash column chromatography (silica gel; CH_2Cl_2/Hexane, 10-30%).

Data for **3b**: R_f 0.80 (10% Et_2O/Hexane). **^1H NMR (400 MHz, $CDCl_3$)** δ 7.47 – 7.29 (m, 8H), 7.19-7.15 (m, 2H), 5.17 (m, 2H), 2.49 – 2.44 (m, 1H), 1.54 (s, 3H), 1.07 (s, 6H), 1.04 (s, 6H), 0.89 (d, *J* = 10.2 Hz, 1H). **^{13}C NMR (101 MHz, $CDCl_3$)** δ 211.3, 148.8, 137.6, 128.4, 128.3, 126.9, 126.4, 126.3, 125.9, 103.0, 83.0, 79.3, 32.6, 31.5, 25.4, 24.4, 19.0. [note: the carbon attached to boron was not observed due to quadrupole broadening caused by the ^{11}B nucleus]. **^{11}B NMR (128 MHz, $CDCl_3$)** δ 32.9. **HRMS (APCI)** *m/z* calcd for $C_{25}H_{30}BO_2$ $[M+H]^+$ 373.2338; found 373.2336.

(±)-2-((1*R*,2*R*,3*S*)-2-Isopropyl-2-phenyl-3-(1-phenylpropa-1,2-dien-1-yl)cyclopropyl)-4,4,5,5-tetramethyl-1,3,2-dioxaborolane (3c).

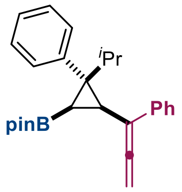

From bisboronate cyclopropane **1c** (82 mg, 0.2 mmol) following the general procedure GP.A (CuCN 5 mol%, 3-phenylprop-2-yn-1-yl 4-methylbenzenesulfonate, rt, 20 h), compound **3c** (64.0 mg, 0.16 mmol) was obtained in 80% yield as a colorless oil, after purification by flash column chromatography (silica gel; CH_2Cl_2/Hexane, 10-30%).

Data for **3c**: R_f 0.60 (10% Et_2O/Hexane). **^{1}H NMR (400 MHz, $CDCl_3$)** δ 7.51 (d, *J* = 7.5 Hz, 2H), 7.36 – 7.14 (m, 8H), 5.21 (m, 1H), 5.14 (m, 1H), 2.49 (dt, *J* = 10.1, 4.4 Hz, 1H), 2.41 – 2.38 (m, 1H), 0.98 (s, 6H), 0.97 (s, 6H), 0.89-0.86 (m, 4H), 0.80 (d, *J* = 6.7 Hz, 3H). **^{13}C NMR (101 MHz, $CDCl_3$)** δ 210.1, 144.8, 138.7, 131.6, 128.2, 127.3, 126.4, 126.36, 126.34, 102.0, 82.8, 79.0, 45.3, 31.9, 27.8, 25.3, 24.4, 22.2, 21.9, 14.3. **^{11}B NMR (128 MHz, $CDCl_3$)** δ 33.3. **HRMS (APCI)** *m/z* calcd for $C_{27}H_{34}BO_2$ $[M+H]^+$ 401.2651; found 401.2647.

(±)-2-((1*R*,2*S*,3*S*)-2-(4-Methoxyphenyl)-2-methyl-3-(1-phenylpropa-1,2-dien-1-yl)cyclopropyl)-4,4,5,5-tetramethyl-1,3,2-dioxaborolane (3d).

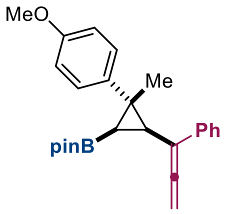

From bisboronate cyclopropane **1b** (80 mg, 0.19 mmol) following the general procedure GP.A (CuCN 5 mol%, 3-phenylprop-2-yn-1-yl 4-methylbenzenesulfonate**,** rt, 20 h), compound **3d** (42.7 mg, 0.11 mmol) was obtained in 55% yield as a colorless oil, after purification by flash column chromatography (silica gel; CH_2Cl_2/Hexane, 10-30%).

Data for **3d**: R_f 0.60 (20% Et_2O/Hexane). **^{1}H NMR (400 MHz, $CDCl_3$)** δ 7.48 (m, 2H), 7.31 – 7.26 (m, 4H), 7.18 – 7.14 (m, 1H), 6.86 (d, *J* = 8.7 Hz, 2H), 5.17 (m, 2H), 3.81 (s, 3H), 2.44 – 2.39 (m, 1H), 1.52 (s, 3H), 1.07 (s, 6H), 1.04 (s, 6H), 0.84 (d, *J* = 10.1 Hz, 1H). **^{13}C NMR (101 MHz, $CDCl_3$)** δ 211.2, 157.7, 141.0, 137.5, 128.1, 127.9, 126.3, 126.1, 113.6, 102.9, 82.8, 79.1, 55.3, 31.7, 31.2, 25.3, 24.3, 19.2. [note: the carbon attached to boron was not observed due to quadrupole broadening caused by the ^{11}B nucleus]. **^{11}B NMR (128 MHz, $CDCl_3$)** δ 33.7**. HRMS (APCI)** *m/z* calcd for $C_{26}H_{32}BO_3$ $[M+H]^+$ 403.2444; found 403.2423.

(±)-*tert*-Butyl (1*S*,2*R*)-1-(1-phenylpropa-1,2-dien-1-yl)-2-(4,4,5,5-tetramethyl-1,3,2-dioxaborolan-2-yl)-6-azaspiro[2.5]octane-6-carboxylate (3e).

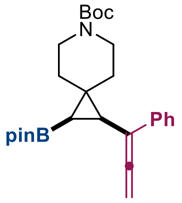

From bisboronate cyclopropane **1d** (93 mg, 0.2 mmol) following the general procedure GP.A (CuCN 5 mol%, 3-phenylprop-2-yn-1-yl 4-methylbenzenesulfonate**,** rt, 20 h), compound **3e** (70.4 mg, 0.16 mmol) was obtained in 78% yield as a colorless oil, after purification by flash column chromatography (silica gel; Et_2O/CH_2Cl_2, 1-4%).

Data for **3e**: R_f 0.50 (40% Et_2O/Hexane). **^{1}H NMR (400 MHz, $CDCl_3$)** δ 7.45 (d, *J* = 7.3 Hz, 2H), 7.30 – 7.26 (m, 2H), 7.17 – 7.13 (m, 1H), 5.09 (m, 2H), 3.90 (brs, 2H), 3.05 (t, *J* = 11.9 Hz, 1H), 2.83 (t, *J* = 11.9 Hz, 1H), 2.03 – 1.74 (m, 4H), 1.46 (s, 9H), 1.09 (m, 1H), 0.99 (s, 12H), 0.27 (d, *J* = 9.7 Hz, 1H). **2D-NOESY (400 MHz,**

$CDCl_3$) crosspoint between: 0.27 (d, *J* = 9.7 Hz, 1H) / 2.03 (dt, *J* = 10.5, 3.9 Hz, 1H); 2.03 (dt, *J* = 10.5, 3.9 Hz, 1H) / 5.09 (d, *J* = 4.4 Hz, 2H); 3.90 (brs, 2H) / 3.05 (t, *J* = 11.9 Hz, 1H); 3.90 (brs, 2H) / 2.83 (t, *J* = 11.9 Hz, 1H). **^{13}C NMR (101 MHz, $CDCl_3$, HSQC)** δ 210.7, 155.1, 137.5, 128.33, 128.28, 126.5, 126.1, 102.1, 82. 9, 79.3, 43.9, 39.1, 30.6, 29.9, 28.6, 28.0, 25.4, 24.3, 11.8. **^{11}B NMR (128 MHz, $CDCl_3$)** δ 33.3. **HRMS (APCI)** *m/z* calcd for $C_{27}H_{39}BNO_4$ $[M+H]^+$ 452.2972; found 452.2974.

4,4,5,5-tetramethyl-2-((1R,2S,3S)-2-methyl-3-(1-(4-nitrophenoxy)buta-2,3-dien-2-yl)-2-phenylcyclopropyl)-1,3,2-dioxaborolane, (3f).

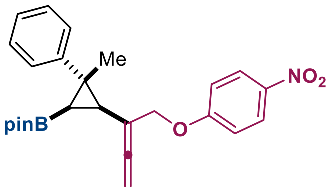

From cyclopropyl bisboronate **1a** (192 mg, 0.5 mmol) following the general procedure GP.A [CuCN 20 mol%, 4-(4-nitrophenoxy)but-2-yn-1-yl 4-methylbenzenesulfonate , 60 °C, 4 h], compound **3f** (206. mg, 0.46 mmol) was obtained in 92% yield as a colorless oil, after purification by flash column chromatography (silica gel; Et_2O/Hexane, 5-15%).

Data for **3f**: R_f 0.30 (10% Et_2O/Hexane). **^{1}H NMR (400 MHz, $CDCl_3$)** δ 8.15 (d, *J* = 9.2 Hz, 2H), 7.29 – 7.19 (m, 4H), 7.13 (t, *J* = 6.2 Hz, 1H), 6.99 (d, *J* = 9.3 Hz, 2H), 4.87 (brs, 2H), 4.74 (s, 2H), 2.03 – 1.99 (m, 1H), 1.49 (s, 3H), 1.25 (s, 6H), 1.24 (s, 6H), 0.67 (d, *J* = 10.1 Hz, 1H). **^{13}C NMR (101 MHz, $CDCl_3$)** δ 209.2, 164.0, 148.6, 141.6, 128.4, 127.5, 125.9, 115.1, 97.4, 83.3, 78.6, 70.4, 32.5, 29.5, 25.5, 24.7, 20.2, 13.1. **^{11}B NMR (128 MHz, $CDCl_3$)** δ 32.2. **HRMS (APCI)** *m/z* calcd for $C_{26}H_{31}BNO_5$ $[M+H]^+$ 448.2294; found 448.2292.

(±)-*tert*-Butyl (1*S*,2*R*)-1-(1-methylpropa-1,2-dien-1-yl)-2-(4,4,5,5-tetramethyl-1,3,2-dioxaborolan-2-yl)-6-azaspiro[2.5]octane-6-carboxylate, (3g).

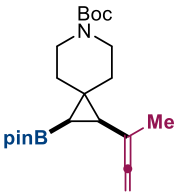

From cyclopropyl bis(boronate) **1d** (46 mg, 0.1 mmol) following the general procedure GP.A (CuCN 10 mol%, but-2-yn-1-yl 4-methylbenzenesulfonate, rt, 20 h), compound **3g** (28 mg, 0.07 mmol) was obtained in 72% yield as a colorless oil, after purification by flash column chromatography (silica gel; Et_2O/CH_2Cl_2, 0.5-2%).

Data for **3g**: R_f 0.50 (40% Et_2O/Hexane). **^{1}H NMR (400 MHz, $CDCl_3$)** δ 4.59 (m, 2H), 3.84 – 3.66 (m, 2H), 3.11 (ddd, *J* = 13.1, 9.9, 3.3 Hz, 1H), 3.05 – 2.97 (m, 1H), 1.81 – 1.76 (m, 2H), 1.72 (t, *J* = 2.6 Hz, 3H), 1.69 – 1.63 (m, 1H), 1.52 – 1.46 (m, 1H), 1.46 (s, 9H), 1.23 (s, 6H), 1.21 (s, 6H), 1.10-1.04 (m, 1H), 0.04 (d, *J* = 9.9 Hz, 1H). **^{13}C NMR (101 MHz, $CDCl_3$)** δ 208.1, 155.1, 95.9, 83.0, 79.3, 75.2, 44.0, 39.2, 33.0, 30.1, 28.7, 28.0, 25.5, 24.7, 20.4, 11.8. **^{11}B NMR (128 MHz, $CDCl_3$)** δ 33.3. **HRMS (ESI)** *m/z* calcd for $C_{22}H_{36}BNNaO_4$ $[M+Na]^+$ 412.2634; found 412.2632.

(±)-Triisopropyl(((1*S*,2*S*,3*R*)-2-methyl-2-phenyl-3-(4,4,5,5-tetramethyl-1,3,2-dioxaborolan-2-yl)cyclopropyl)ethynyl)silane (4a).

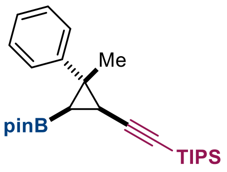

From bisboronate cyclopropane **1a** (38 mg, 0.1 mmol) following the general procedure GP.A (CuCN 20 mol%, (bromoethynyl)triisopropylsilane, 60 °C, 4 h). The crude reaction mixture contained compound **4a** and a 27% of monodeborylated compound. After purification by flash column chromatography (silica gel; CH_2Cl_2/Hexane, 5-10%) compound **4a** (21 mg, 0.05 mmol) was obtained in 60% yield as a colorless oil.

Data for **4a**: R_f 0.70 (10% Et_2O/Hexane). **^{1}H NMR (400 MHz, $CDCl_3$)** δ 7.31 – 7.15 (m, 5H), 2.02 (d, *J* = 9.9 Hz, 1H), 1.62 (s, 3H), 1.27 (s, 12H), 1.18 – 0.94 (m, 21H), 0.77 (d, *J* = 9.9 Hz, 1H). **^{13}C NMR (101 MHz, $CDCl_3$)** δ 147.4, 128.4, 127.3, 126.3, 107.0, 83.4, 80.9, 32.4, 25.2, 24.8, 21.1, 20.4, 18.9, 11.6. **^{11}B NMR (128 MHz, $CDCl_3$)** δ 32.7. **HRMS (APCI)** *m/z* calcd for $C_{27}H_{44}BO_2Si$ $[M+H]^+$ 439.3203; found 439.3225.

(±)-4,4,5,5-Tetramethyl-2-((1*R*,2*S*,3*R*)-2-methyl-3-(oct-1-yn-1-yl)-2-phenylcyclopropyl)-1,3,2-dioxaborolane (4b).

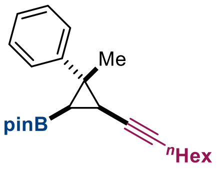

From bisboronate cyclopropane **1a** (38 mg, 0.1 mmol) following the general procedure GP.A (CuCN 20 mol%, 1-bromooct-1-yne, 60 °C, 4 h), compound **4b** (15.8 mg, 0.04 mmol) was obtained in 49% yield as a colorless oil, after purification by flash column chromatography (silica gel; CH_2Cl_2/Hexane, 5-20%).

Data for **4b**: R_f 0.30 (40% CH_2Cl_2/Hexane). **^{1}H NMR (300 MHz, $CDCl_3$)** δ 7.30 – 7.21 (m, 4H), 7.16 (t, *J* = 6.7 Hz, 1H), 2.20 (m, 2H), 1.91 (d, *J* = 9.9 Hz, 1H), 1.59 (s, 3H), 1.52 – 1.28 (m, 8H), 1.28 (s, 12H), 0.89 (t, *J* = 6.7 Hz, 3H), 0.72 (d, *J* = 10.0 Hz, 1H). **^{13}C NMR (101 MHz, $CDCl_3$)** δ 147.8, 128.3, 127.6, 126.2, 83.2, 80.8, 78.9, 32.0, 31.6, 29.3, 28.8, 25.1, 25.0, 22.7, 20.5, 19.9, 19.2, 14.2. **^{11}B NMR (128 MHz, $CDCl_3$)** δ 32.7. **HRMS (APCI)** *m/z* calcd for $C_{24}H_{36}BO_2$ $[M+H]^+$ 367.2807; found 367.2810.

(±)-4,4,5,5-Tetramethyl-2-((1*R*,2*S*,3*R*)-2-methyl-2-phenyl-3-(phenylethynyl)cyclopropyl)-1,3,2-dioxaborolane (4c).

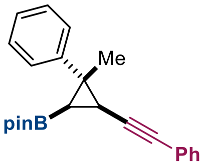

From bisboronate cyclopropane **1a** (114 mg, 0.3 mmol) following the general procedure GP.A (CuCN 20 mol%, (bromoethynyl)benzene, 60 °C, 4 h), compound **4c** (74.2 mg, 0.21 mmol) was obtained in 69% yield as a colorless oil, after purification by flash column chromatography (Florisil; Et_2O/Hexane, 2-3%). Alternatively, using a 5% of CuCN at rt and after 18 h a 60% of **4c** is obtained.

Data for **4c**: R_f 0.60 (10% Et_2O/Hexane). **^{1}H NMR (300 MHz, $CDCl_3$)** δ 7.46 – 7.16 (m, 10H), 2.14 (d, *J* = 9.9 Hz, 1H), 1.70 (s, 3H), 1.28 (s, 12H), 0.92 (d, *J* = 9.9 Hz, 1H). **2D-NOESY (400 MHz, $CDCl_3$)** crosspoint between: 0.92 (d, *J* = 9.9 Hz, 1H) / 2.14 (d, *J* = 9.9 Hz, 1H). **^{13}C NMR (101 MHz, $CDCl_3$)** δ 147.4, 131.6, 128.4, 128.3, 127.6, 127.5, 126.4, 124.6, 89.8, 83.4, 80.8, 33.1, 25.1, 25.1, 20.6, 20.3, 16.4. **^{11}B NMR (128 MHz, $CDCl_3$)** δ 32.1. **HRMS (APCI)** *m/z* calcd for $C_{24}H_{28}BO_2$ $[M+H]^+$ 359.2181; found 359.2174.

(±)-2-((1*R*,2*S*,3*R*)-2-(4-Chlorophenyl)-2-methyl-3-(phenylethynyl)cyclopropyl)-4,4,5,5-tetramethyl-1,3,2-dioxaborolane (4d).

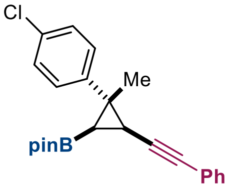

From bisboronate cyclopropane **1e** (42 mg, 0.1 mmol) following the general procedure GP.A (CuCN 20 mol%, (bromoethynyl)benzene, 60 °C, 4 h), compound **4d** (25.1 mg, 0.06 mmol) was obtained in 64% yield as a colorless oil, after purification by flash column chromatography (Florisil; Et_2O/Hexane, 1-5%).

Data for **4d**: R_f 0.60 (10% Et_2O/Hexane). **^{1}H NMR (300 MHz, $CDCl_3$)** δ 7.44 – 7.42 (m, 2H), 7.29 – 7.26 (m, 7H), 2.10 (d, *J* = 10.0 Hz, 1H), 1.67 (s, 3H), 1.28 (s, 12H), 0.87 (d, *J* = 9.9 Hz, 1H). **^{13}C NMR (101 MHz, $CDCl_3$)** δ 146.0, 132.1, 131.6, 129.1, 128.5, 128.3, 127.6, 124.4, 89.3, 83.5, 81.0, 32.6, 25.13, 25.10, 20.6, 20.3, 16.6. **^{11}B NMR (128 MHz, $CDCl_3$)** δ 32.7. **HRMS (APCI)** *m/z* calcd for $C_{24}H_{27}BClO_2$ $[M+H]^+$ 393.1791; found 393.1790.

(±)-4,4,5,5-Tetramethyl-2-((1*R*,2*S*,3*R*)-2-methyl-3-(phenylethynyl)-2-(*m*-tolyl)cyclopropyl)-1,3,2-dioxaborolane (4e).

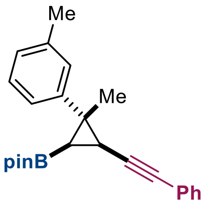

From bisboronate cyclopropane **1f** (40 mg, 0.1 mmol) following the general procedure GP.A (CuCN 20 mol%, (bromoethynyl)benzene, 60 °C, 4 h), compound **4e** (20.1 mg, 0.05 mmol) was obtained in 54% yield as a colorless oil, after purification by flash column chromatography (Florisil; Et_2O/Hexane, 1-5%).

Data for **4e**: R_f 0.60 (10% Et_2O/Hexane). **^{1}H NMR (400 MHz, $CDCl_3$)** δ 7.44 (dd, *J* = 7.7, 2.0 Hz, 2H), 7.29 – 7.26 (m, 3H), 7.18 – 7.16 (m, 3H), 7.02 (dd, *J* = 5.2, 3.4 Hz, 1H), 2.34 (s, 3H), 2.12 (d, *J* = 9.9 Hz, 1H), 1.68 (s, 3H), 1.29 (s, 12H), 0.90 (d, *J* = 9.9 Hz, 1H). **^{13}C NMR (101 MHz, $CDCl_3$)** δ 147.4, 138.1, 131.6, 128.5, 128.4, 128.3, 127.4, 127.2, 124.8, 124.6, 89.9, 83.4, 80.7, 33.3, 25.13, 25.10, 21.5, 20.8, 20.2, 16.7. **^{11}B NMR (128 MHz, $CDCl_3$)** δ 32.6. **HRMS (APCI)** *m/z* calcd for $C_{25}H_{30}BO_2$ $[M+H]^+$ 373.2338; found 373.2340.

(±)-*tert*-Butyl (1*S*,2*R*)-1-(phenylethynyl)-2-(4,4,5,5-tetramethyl-1,3,2-dioxaborolan-2-yl)-6-azaspiro [2.5]octane-6-carboxylate (4f).

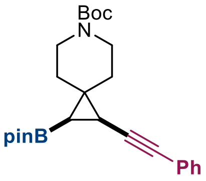

From bisboronate cyclopropane **1d** (46 mg, 0.1 mmol) following the general procedure GP.A (CuCN 20 mol%, (bromoethynyl)benzene, 60 °C, 4 h), compound **4f** (26.7 mg, 0.06 mmol) was obtained in 61% yield as a colorless oil, after purification by flash column chromatography (Florisil; Et_2O/Hexane, 1-5%).

Data for **4f**: R_f 0.70 (10% Et_2O/Hexane). **^{1}H NMR (400 MHz, $CDCl_3$)** δ 7.38 – 7.35 (m, 2H), 7.26 – 7.24 (m, 3H), 3.58 – 3.54 (m, 2H), 3.46 – 3.38 (m, 2H), 1.88 (m, 2H), 1.73 (d, *J* = 9.8 Hz, 1H), 1.46 (s, 9H), 1.32 – 1.23 (m, 2H), 1.23 (s, 12H), 0.33 (d, *J* = 9.5 Hz, 1H). **^{13}C NMR (101 MHz, $CDCl_3$)** δ 155.1, 131.6, 128.3, 127.5, 124.4, 89.3, 83.3, 80.1, 79.4, 44.2, 43.4, 37.6, 30.5, 28.6, 25.1, 25.0, 18.2, 15.5. **^{11}B NMR (128 MHz, $CDCl_3$)** δ 32.2. **HRMS (APCI)** *m/z* calcd for $C_{21}H_{29}BNO_2$ $[M-Boc+2H]^+$ 338.2290; found 338.2290.

Amination of cyclopropyl bisboronates

(±)-(1*S*,2*R*,3*S*)-*N*,*N*-Dibenzyl-2-methyl-2-phenyl-3-(4,4,5,5-tetramethyl-1,3,2-dioxaborolan-2-yl)cyclopropan-1-amine (5a).

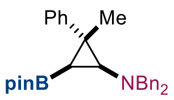

From bisboronate cyclopropane **1a** (154 mg, 0.4 mmol) following the general procedure GP.C (CuCN 20 mol%, *O*-benzoyl-*N*,*N*-dibenzylhydroxylamine, 60 °C, 6 h), compound **5a** (136.0 mg, 0.3 mmol) was obtained in 75% yield as a colorless oil, after purification by flash column chromatography (Florisil; Et_2O/Hexane, 1-50%).

Data for **5a**: R_f 0.50 (10% Et_2O/Hexane). **^{1}H NMR (400 MHz, $CDCl_3$)** δ 7.35 – 7.13 (m, 15H), 3.77 (d, *J* = 13.9 Hz, 2H), 3.68 (d, *J* = 14.1 Hz, 2H), 2.50 (d, *J* = 8.2 Hz, 1H), 1.62 (s, 3H), 1.28 (s, 12H), 0.72 (d, *J* = 8.2 Hz, 1H). **^{13}C NMR (101 MHz, $CDCl_3$)** δ 148.2, 137.8, 129.9, 128.2, 128.1, 127.1, 126.9, 125.7, 83.0, 56.2, 54.0, 34.7, 25.2, 24.9, 17.7. [note: the carbon attached to boron was not observed due to quadrupole broadening caused by the ^{11}B nucleus]. **^{11}B NMR (128 MHz, $CDCl_3$)** δ 33.4. **HRMS (ESI)** *m/z* calcd for $C_{30}H_{37}BNO_2$ $[M+H]^+$ 454.2917; found 454.2908.

(±)-4-((1*S*,2*R*,3*S*)-2-Methyl-2-phenyl-3-(4,4,5,5-tetramethyl-1,3,2-dioxaborolan-2-yl)cyclopropyl) morpholine (5b).

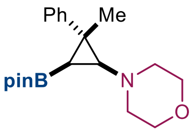

From bisboronate cyclopropane **1a** (38 mg, 0.1 mmol) following the general procedure GP.C (CuCN 20 mol%, morpholino benzoate, 60 °C, 6 h), compound **5b** (20.6 mg, 0.06 mmol) was obtained in 62% yield as a colorless oil, after purification by flash column chromatography (Florisil; Et_2O/Hexane, 5-20%). The data of the compound was consistent with reported values.[12]

Data for **5b**: R_f 0.20 (10% Et_2O/Hexane). **^{1}H NMR (300 MHz, $CDCl_3$)** δ 7.28 – 7.26 (m, 4H), 7.19 – 7.17 (m, 1H), 3.73 (m, 4H), 2.72 – 2.65 (m, 2H), 2.60 – 2.55 (m, 2H), 2.15 (d, *J* = 8.3 Hz, 1H), 1.59 (s, 3H), 1.27 (s, 12H), 0.50 (d, *J* = 8.3 Hz, 1H). **^{13}C NMR (101 MHz, $CDCl_3$)** δ 148.4, 128.3, 127.4, 125.9, 82.9, 67.2, 55.4, 53.9, 32.3, 25.4, 24.9, 18.3. [note: the carbon attached to boron was not observed due to quadrupole broadening caused by the ^{11}B nucleus]. **^{11}B NMR (128 MHz, $CDCl_3$)** δ 32.9.

(±)-(1*S*,2*R*,3*S*)-*N*,*N*-Diallyl-2-methyl-2-phenyl-3-(4,4,5,5-tetramethyl-1,3,2-dioxaborolan-2-yl) cyclopropan-1-amine (5c).

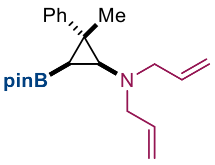

From bisboronate cyclopropane **1a** (76 mg, 0.2 mmol) following the general procedure GP.C (CuCN 20 mol%, *N*,*N*-diallyl-*O*-benzoylhydroxylamine, 60 °C, 24 h), compound **5c** (38.9 mg, 0.11 mmol) was obtained in 55% yield as a colorless oil, after purification by flash column chromatography (Florisil; Et_2O/Hexane, 2-5%). The data of the compound was consistent with reported values.[12]

Data for **5c**: R_f 0.40 (10% Et_2O/Hexane). **^{1}H NMR (300 MHz, $CDCl_3$)** δ 7.27 – 7.26 (m, 4H), 7.17 – 7.15 (m, 1H), 6.03 – 5.89 (m, 2H), 5.21 – 5. 11 (m, 4H), 3.38 – 3.21 (m, 4H), 2.51 (d, *J* = 8.3 Hz, 1H), 1.60 (s, 3H), 1.27 (s, 12H), 0.53 (d, *J* = 8.3 Hz, 1H). **^{13}C NMR (101 MHz, $CDCl_3$)** δ 148.7, 135.5, 128.3, 127.6, 125.8, 117.3, 82.9, 56.2, 53.8, 34.2, 25.1, 25.0, 18.5. [note: the carbon attached to boron was not observed due to quadrupole broadening caused by the ^{11}B nucleus]. **^{11}B NMR (128 MHz, $CDCl_3$)** δ 33.0.

(±)-(1*S*,2*R*,3*S*)-*N*,*N*-Dibenzyl-2-(4-methoxyphenyl)-2-methyl-3-(4,4,5,5-tetramethyl-1,3,2-dioxaborolan-2-yl)cyclopropan-1-amine (5d).

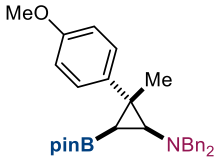

From bisboronate cyclopropane **1b** (42 mg, 0.1 mmol) following the general procedure GP.C (CuCN 20 mol%, *O*-benzoyl-*N*,*N*-dibenzylhydroxylamine, 60 °C, 6 h), compound **5d** (32.4 mg, 0.07 mmol) was obtained in 67% yield as a colorless oil, after purification by flash column chromatography (Florisil; Et_2O/Hexane, 1-5%).

Data for **5d**: R_f 0.50 (20% Et_2O/Hexane). **^{1}H NMR (400 MHz, $CDCl_3$)** δ 7.34 – 7.24 (m, 10H), 7.12 (d, *J* = 8.7 Hz, 2H), 6.79 (d, *J* = 8.7 Hz, 2H), 3.78 – 3.74 (m, 5H), 3.67 (d, *J* = 14.1 Hz, 2H), 2.44 (d, *J* = 8.2 Hz, 1H), 1.59 (s, 3H), 1.27 (s, 12H), 0.65 (d, *J* = 8.2 Hz, 1H). **^{13}C NMR (101 MHz, $CDCl_3$)** δ 157.7, 140.5, 137.9, 129.9, 128.2, 128.1, 126.9, 113.6, 83.0, 56.2, 55.4, 54.1, 34.2, 25.2, 25.0, 18.1, 16.3. **^{11}B NMR (128 MHz, $CDCl_3$)** δ 33.4. **HRMS (APCI)** *m/z* calcd for $C_{31}H_{39}BNO_3$ $[M+H]^+$ 484.3023; found 484.3029.

(±)-(1*S*,2*S*,3*S*)-*N*,*N*-Dibenzyl-2-isopropyl-2-phenyl-3-(4,4,5,5-tetramethyl-1,3,2-dioxaborolan-2-yl) cyclopropan-1-amine (5e).

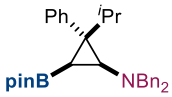

From bisboronate cyclopropane **1c** (277 mg, 0.672 mmol) following the general procedure GP.C (CuCN 20 mol%, *O*-benzoyl-*N*,*N*-dibenzylhydroxylamine, 60 °C, 24 h), compound **5e** (223.3 mg, 0.46 mmol) was obtained in 69% yield as a colorless oil, after purification by flash column chromatography (Florisil; Et_2O/Hexane, 1-10%).

Data for **5e**: R_f 0.50 (10% Et_2O/Hexane). **^{1}H NMR (400 MHz, $CDCl_3$)** δ 7.33 – 7.17 (m, 15H), 3.91 (d, *J* = 14.6 Hz, 2H), 3.73 (d, *J* = 14.6 Hz, 2H), 2.90 – 2.87 (m, 1H), 2.56 (d, *J* = 8.4 Hz, 1H), 1.32 (s, 12H), 1.00 (d, *J* = 6.8 Hz, 3H), 0.79 (d, *J* = 6.8 Hz, 3H), 0.71 (d, *J* = 8.4 Hz, 1H). **^{13}C NMR (101 MHz, $CDCl_3$)** δ 143.8, 137.5, 131.7, 129.7, 128.2, 127.4, 126.9, 126.2, 83.1, 77.2, 56.1, 55.7, 48.5, 26.5, 25.4, 25.0, 22.8, 21.5, 15.3. **^{11}B NMR (128 MHz, $CDCl_3$)** δ 32.4. **HRMS (APCI)** *m/z* calcd for $C_{32}H_{41}BNO_2$ $[M+H]^+$ 482.3231; found 482.3238.

(±)-*tert*-Butyl (1S,2S)-1-(dibenzylamino)-2-(4,4,5,5-tetramethyl-1,3,2-dioxaborolan-2-yl)-6-azaspiro [2.5]octane-6-carboxylate (5f).

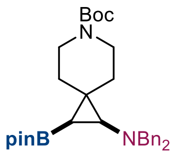

From bisboronate cyclopropane **1d** (46 mg, 0.1 mmol) following the general procedure GP.C (CuCN 20 mol%, *O*-benzoyl-*N*,*N*-dibenzylhydroxylamine, 60 °C, 5 h), compound **5f** (29.3 mg, 0.06 mmol) was obtained in 55% yield as a colorless oil, after purification by flash column chromatography (silica gel; Et_2O/Hexane, 5-40%).

Data for **5f**: R_f 0.60 (40% Et_2O/Hexane). **^{1}H NMR (400 MHz, $CDCl_3$, COSY)** δ 7.32 – 7.22 (m, 10H), 4.40 – 3.86 (m, 2H), 3.68 (d, *J* = 14.1 Hz, 2H), 3.47 (d, *J* = 14.0 Hz, 2H), 2.88 – 2.73 (m, 2H), 2.17 -2.11 (m, 2H), 1.80 (td, *J* = 12.5, 4.2 Hz, 1H), 1.45 (s, 9H), 1.24-1.21 (m, 1H), 1.21 (s, 6H), 1.18 (s, 6H), 0.79 (d, *J* = 13.2 Hz, 1H), 0.02 (d, *J* = 8.3 Hz, 1H). **2D-NOESY (400 MHz, $CDCl_3$)** crosspoint between: 0.02 (d, *J* = 8.3 Hz, 1H) / 2.19 -2.10 (m, 2H). **^{13}C NMR (101 MHz, $CDCl_3$, HSQC)** δ 155.1, 137.8, 129.9, 128.1, 127.0, 83.0, 79.3, 56.6, 53.5, 43.6, 37.6, 33.4, 28.6, 25.5, 25.3, 24.6, 24.5, 13.1. **^{11}B NMR (128 MHz, $CDCl_3$)** δ 33.3. **HRMS (APCI)** *m/z* calcd for $C_{32}H_{46}BN_2O_4$ $[M+H]^+$ 533.3551; found 533.3548.

Functionalization of spirocyclobutyl bisboronates

(±)-2-((1*R*,2*R*)-2-Allyl-7,7-dimethylspiro[3.5]nonan-1-yl)-4,4,5,5-tetramethyl-1,3,2-dioxaborolane (7a).

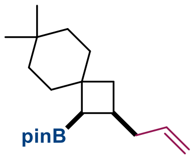

From bisboronate cyclobutane **6a** (122 mg, 0.3 mmol) following the general procedure GP.B (CuCN 30 mol%, CH_2=$CHCH_2Br$, 24 h), compound **7a** (66.8 mg, 0.21 mmol) was obtained in 70% yield as a colorless oil, after purification by flash column chromatography (silica gel; CH_2Cl_2/Hexane, 1-20%).

Data for **7a**: R_f 0.80 (10% Et_2O/Hexane). **^{1}H NMR (400 MHz, $CDCl_3$, COSY, NOESY)** δ 5.71 (m, 1H), 4.98-4.88 (m, 2H), 2.43-2.18 (m, 3H), 1.87 (ddd, *J* = 11.1, 8.3, 3.0 Hz, 1H) 1.64 – 1.41 (m, 6H), 1.25 (s, 6H), 1.24 (s, 6H), 1.22 (m, 2H), 1.09 (t, *J* = 6.1 Hz, 2H), 0.85 (s, 3H), 0.84 (s, 3H). **2D-NOESY (400 MHz, $CDCl_3$)** crosspoint between: 2.43 – 2.34 (m, 1H) / 1.62 (m, 1 H). **^{13}C NMR (101 MHz, $CDCl_3$, HSQC)** δ 138.1, 114.4, 83.0, 38.8, 37.8, 36.1, 36.0, 35.2, 33.7, 30.5, 29.7, 25.2. [note: the carbon attached to boron was not observed due to quadrupole broadening caused by the ^{11}B nucleus]. **^{11}B NMR (128 MHz, $CDCl_3$)** δ 33.9. **HRMS (APCI)** *m/z* calcd for $C_{20}H_{36}BO_2$ $[M+H]^+$ 319.2807; found 319.2808.

(±)-2-((1*R*,2*S*)-2-(2-Bromoallyl)-7,7-dimethylspiro[3.5]nonan-1-yl)-4,4,5,5-tetramethyl-1,3,2-dioxaborolane (7b).

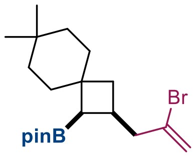

From bisboronate cyclobutane **6a** (122 mg, 0.3 mmol) following the general procedure GP.B [CuCN 30 mol%, CH_2=$C(Br)CH_2Br$, 24 h], compound **7b** (78.6 mg, 0.2 mmol) was obtained in 66% yield as a colorless oil, after purification by flash column chromatography (silica gel; CH_2Cl_2/Hexane, 5-15%).

Data for **7b**: R_f 0.70 (10% Et_2O/Hexane). **^{1}H NMR (400 MHz, $CDCl_3$)** δ 5.46 (s, 1H), 5.34 (s, 1H), 2.79 – 2.71 (m, 1H), 2.67 – 2.57 (m, 2H), 1.95 – 1.88 (m, 1H), 1.67 (d, *J* = 6.1 Hz, 1H), 1.61 – 1.39 (m, 5H), 1.26 (s, 6H),

1.24 – 1.21 (m, 8H), 1.11 – 1.08 (m, 2H), 0.85 (s, 3H), 0.85 (s, 3H**)**. **^{13}C NMR (101 MHz, $CDCl_3$, HSQC)** δ 134.1, 116.0, 83.2, 46.2, 38.0, 37.5, 36.1, 35.9, 35.1, 33.8, 29.7, 29.5, 25.2. [note: the carbon attached to boron was not observed due to quadrupole broadening caused by the ^{11}B nucleus]. **^{11}B NMR (128 MHz, $CDCl_3$)** δ 33.0. **HRMS (APCI)** *m/z* calcd for $C_{20}H_{35}BBrO_2$ $[M+H]^+$ 397.1912; found 397.1903.

(±)-2-((1*R*,2*R*)-2-Allyl-7-oxaspiro[3.5]nonan-1-yl)-4,4,5,5-tetramethyl-1,3,2-dioxaborolane (7c).

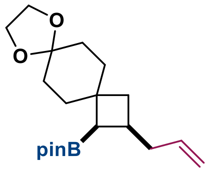

From bisboronate cyclobutane **6b** (153 mg, 0.35 mmol) following the general procedure GP.B (CuCN 30 mol%, CH_2=$CHCH_2Br$, 24 h), compound **7c** (68.5 mg, 0.23 mmol) was obtained in 67% yield as a colorless oil, after purification by flash column chromatography (silica gel; CH_2Cl_2/Hexane, 0.5-2%).

Data for **7c**: R_f 0.50 (40% EtOAc-Hexane). **^{1}H NMR (400 MHz, $CDCl_3$, COSY)** δ 5.70 (m, 1H), 4.97-4.88 (m, 2H), 3.91 (s, 4H), 2.45 – 2.39 (m, 1H), 2.29-2.17 (m, 2H), 1.98 (ddd, *J* = 10.9, 8.5, 2.9 Hz, 1H), 1.74 – 1.50 (m, 10H), 1.23 (s, 6H), 1.23 (s, 6H). **^{13}C NMR (101 MHz, $CDCl_3$, HSQC)** δ 137.8, 114.6, 109.0, 83.0, 64.29, 64.25, 38.8, 37.3, 36.8, 36.6, 34.6, 31.78, 31.71, 30.51, 25.23, 25.18, 25.1. [note: the carbon attached to boron was not observed due to quadrupole broadening caused by the ^{11}B nucleus]. **^{11}B NMR (128 MHz, $CDCl_3$)** δ 33.4. **HRMS (APCI)** *m/z* calcd for $C_{20}H_{34}BO_4$ $[M+H]^+$ 349.2548; found 349.2544.

(±)-2-((1*R*,2*R*)-2-Allyl-7-oxaspiro[3.5]nonan-1-yl)-4,4,5,5-tetramethyl-1,3,2-dioxaborolane (7d).

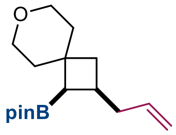

From bisboronate cyclobutane **6c** (153 mg, 0.35 mmol) following the general procedure GP.B (CuCN 30 mol%, CH_2=$CHCH_2Br$, 24 h), compound **7d** (68.5 mg, 0.23 mmol) was obtained in 67% yield as a colorless oil, after purification by flash column chromatography (silica gel; CH_2Cl_2/Hexane, 0.5-2%).

Data for **7d**: R_f 0.50 (40% EtOAc-Hexane). **^{1}H NMR (400 MHz, $CDCl_3$)** δ 5.70 (ddt, *J* = 16.9, 10.2, 6.4 Hz, 1H), 4.95 – 4.91 (m, 2H), 3.69 – 3.42 (m, 4H), 2.50 – 2.39 (m, 1H), 2.34 – 2.17 (m, 2H), 1.99 (m, 1H), 1.73 – 1.57 (m, 6H), 1.24 (s, 6H), 1.22 (s, 6H). **^{13}C NMR (101 MHz, $CDCl_3$)** δ 137.6, 114.7, 83.2, 65.2, 64.9, 39.4, 38.7, 38.1, 37.8, 35.3, 30.3, 25.2, 25.2. [note: the carbon attached to boron was not observed due to quadrupole broadening caused by the ^{11}B nucleus]. **^{11}B NMR (128 MHz, $CDCl_3$)** δ 33.3. **HRMS (APCI)** *m/z* calcd for $C_{17}H_{30}BO_3$ $[M+H]^+$ 293.2286; found 293.2286.

(±)-*tert*-Butyl (1*R*,2*S*)-2-(2-bromoallyl)-1-(4,4,5,5-tetramethyl-1,3,2-dioxaborolan-2-yl)-7-azaspiro[3.5] nonane-7-carboxylate (7e).

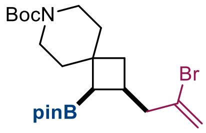

From bisboronate cyclobutane **6d** (143 mg, 0.3 mmol) following the general procedure GP.B [CuCN 30 mol%, CH_2=$C(Br)CH_2Br$, 24 h], compound **7e** (71.9 mg, 0.15 mmol) was obtained in 51% yield as a colorless oil, after purification by flash column chromatography (silica gel; Et_2O/CH_2Cl_2, 0.5-2%).

Data for **7e**: R_f 0.50 (40% Et_2O/Hexane). **^{1}H NMR (400 MHz, $CDCl_3$)** δ 5.46 (m, 1H), 5.36 (m, 1H), 3.46 – 3.40 (m, 1H), 3.35 – 3.25 (m, 2H), 3.13 (ddd, *J* = 13.3, 7.7, 4.2 Hz, 1H), 2.78 – 2.60 (m, 3H), 1.98 (ddd, *J* = 11.0, 7.9, 3.0 Hz, 1H), 1.73 (dd, *J* = 9.1, 2.9 Hz, 1H), 1.65 – 1.52 (m, 5H), 1.44 (s, 9H), 1.25 (s, 6H), 1.24 (s, 6H). **^{13}C NMR (101 MHz, $CDCl_3$, HSQC)** δ 155.1, 133.6, 116.3, 83.4, 79.3, 46.0, 40.7, 38.1, 37.0, 36.9, 36.4, 31.1, 29.4, 28.6, 25.4, 25.2. **^{11}B NMR (128 MHz, $CDCl_3$)** δ 34.4. **HRMS (APCI)** *m/z* calcd for $C_{22}H_{38}BBrNO_4$ $[M+H]^+$ 470.2076; found 470.2075.

(±)-4,4,5,5-Tetramethyl-2-((1*R*,2*R*)-2-(phenylethynyl)-7-oxaspiro[3.5]nonan-1-yl)-1,3,2-dioxaborolane (7f).

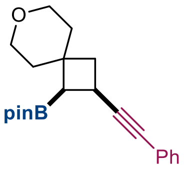

From bisboronate cyclobutane **6c** (113 mg, 0.30 mmol) following the general procedure GP.B (CuCN 30 mol%, (bromoethynyl)benzene, 24 h), compound **7f** (50.7 mg, 0.14 mmol) was obtained in 48% yield as a colorless oil, after purification by flash column chromatography (Florisil; Et_2O/CH_2Cl_2, 0.5-2%).

Data for **7f**: R_f 0.40 (40% Et_2O/Hexane). **^{1}H NMR (400 MHz, $CDCl_3$)** δ 7.38 – 7.35 (m, 2H), 7.27 – 7.24 (m, 3H), 3.70 – 3.48 (m, 4H), 3.40 (m, 1H), 2.29 – 2.20 (m, 2H), 1.99 (dd, *J* = 10.0, 2.1 Hz, 1H), 1.89 – 1.77 (m, 2H), 1.75 – 1.69 (m, 1H), 1.64 – 1.60 (m, 1H), 1.24 (s, 12H). **^{13}C NMR (101 MHz, $CDCl_3$)** δ 131.6, 128.2, 127.5, 124.3, 93.6, 83.5, 82.2, 65.0, 64. 9, 39.1, 38.9, 37.2, 36.4, 33.9, 25.3, 25.2, 21.3**. ^{11}B NMR (128 MHz, $CDCl_3$)** δ 33.3. **HRMS (APCI)** *m/z* calcd for $C_{22}H_{30}BO_3$ $[M+H]^+$ 353.2287; found 353.2291.

(±)-4,4,5,5-Tetramethyl-2-((1*R*,2*R*)-2-(1-phenylpropa-1,2-dien-1-yl)-7-oxaspiro[3.5]nonan-1-yl)-1,3,2-dioxaborolane (7g).

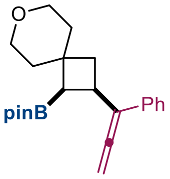

From bisboronate cyclobutane **6c** (113 mg, 0.30 mmol) following the general procedure GP.B (CuCN 30 mol%, 3-phenylprop-2-yn-1-yl 4-methylbenzenesulfonate, 3 h), compound **7g** (58.6 mg, 0.16 mmol) was obtained in 52% yield as a colorless oil, after purification by flash column chromatography (Florisil; Et_2O/CH_2Cl_2, 0.5-2%).

Data for **7g**: R_f 0.40 (40% Et_2O/Hexane). **^{1}H NMR (400 MHz, $CDCl_3$)** δ 7.34 – 7.24 (m, 4H), 7.15 – 7.12 (m, 1H), 5.21 (m, 1H), 5.11 (m, 1H), 3.74 – 3.69 (m, 2H), 3.55 – 3.43 (m, 3H), 2.18 (t, *J* = 11.1 Hz, 1H), 2.11 – 2.05 (m, 2H), 1.85 – 1.80 (m, 2H), 1.77 – 1.71 (m, 2H), 1.01 (s, 6H), 0.89 (s, 6H). **^{13}C NMR (101 MHz, $CDCl_3$)** δ 209.1, 135.8, 128.4, 126.5, 126.3, 108.4, 83.0, 79.8, 65.3, 65.1, 39.3, 37.7, 35.5, 35.0, 29.7, 25.0, 24.7. [note: the carbon attached to boron was not observed due to quadrupole broadening caused by the ^{11}B nucleus]. **^{11}B NMR (128 MHz, $CDCl_3$)** δ 33.9. **HRMS (APCI)** *m/z* calcd for $C_{23}H_{32}BO_3$ $[M+H]^+$ 367.2443; found 367.2437.

(±)-*tert*-Butyl (1*R*,2*R*)-2-(1-phenylpropa-1,2-dien-1-yl)-1-(4,4,5,5-tetramethyl-1,3,2-dioxaborolan-2-yl)-7-azaspiro[3.5]nonane-7-carboxylate (7h).

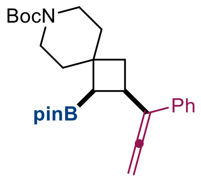

From bisboronate cyclobutane **6d** (143 mg, 0.30 mmol) following the general procedure GP.B (CuCN 30 mol%, 3-phenylprop-2-yn-1-yl 4-methylbenzenesulfonate, 3 h), compound **7h** (86.6 mg, 0.16 mmol) was obtained in 62% yield as a colorless oil, after purification by flash column chromatography (Florisil; Et_2O/CH_2Cl_2, 0.5-2%)

Data for **7h**: R_f 0.40 (40% Et_2O/Hexane). **^{1}H NMR (500 MHz, $CDCl_3$)** δ 7.31 – 7.24 (m, 4H), 7.16 – 7.12 (m, 1H), 5.21 (m, 1H), 5.10 (m, 1H), 3.50 – 3.41 (m, 3H), 3.25 (brs, 2H), 2.17 (t, *J* = 10.5 Hz, 1H), 2.06 – 1.98 (m, 2H), 1.76 (brs, 2H), 1.61 (brs, 2H), 1.45 (s, 9H), 1.01 (s, 6H), 0.89 (s, 6H). **^{13}C NMR (126 MHz, $CDCl_3$, HSQC, HMBC)** δ 209.0, 155.1, 135.8, 131.7, 128.4, 126.5, 126.2, 108.4, 83.0, 79.0, 79.3, 41.5, 40.6, 38.2, 36.5, 35.8, 34.9, 33.8, 29.7, 28.6, 25.0, 24.7. **^{11}B NMR (128 MHz, $CDCl_3$)** δ 32.9. **HRMS (APCI)** *m/z* calcd for $C_{23}H_{33}BNO_2$ [M-CO_2*t*-Bu+2H]$^+$ 366.2603; found 366.2594.

General Procedure for Matteson Homologation

To a solution of the boronic ester (1.0 equiv) in Et_2O (10 mL/mmol), under an argon atmosphere, was added bromochloromethane (2.5 equiv). The mixture was cooled to -78 °C and *n*-BuLi (1.6 M in Hexane, 2.5 equiv) was added dropwise, and the solution was stirred at this temperature for 1 h. Then, the mixture was allowed to warm slowly to room temperature (TLC, 2 h). The reaction was then partitioned with H_2O (20 mL/mmol) and Et_2O and the aqueous layer was extracted with Et_2O (x2). The combined organic layers were dried over $MgSO_4$, filtered and concentrated under vacuum. The crude product was purified by flash column chromatography on silica gel to afford the corresponding product.

(±)-4,4,5,5-Tetramethyl-2-(((1*R*,2*R*)-2-(1-phenylpropa-1,2-dien-1-yl)-7-oxaspiro[3.5]nonan-1-yl)methyl)-1,3,2-dioxaborolane (8).

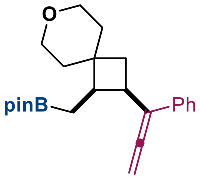

From spirocyclobutane **7g** (27.0 mg, 0.07 mmol) following the general procedure described above compound **8** was obtained along with 15% of starting boronate. After purification by flash column chromatography (silica gel; Et_2O/CH_2Cl_2 0.5-2%), compound **8** (36.4 mg, 0.09 mmol) was obtained in 64% yield as a colorless oil.

Data for **8**: R_f 0.50 (1% Et_2O/CH_2Cl_2). **^{1}H NMR (500 MHz, $CDCl_3$)** δ 7.36 (d, *J* =7.6 Hz, 2H), 7.29 – 7.26 (m, 2H), 7.16 (t, *J* = 7.3 Hz, 1H), 5.22 – 5.14 (m, 2H), 3.84 (dt, *J* = 11.5, 3.7 Hz, 1H), 3.75 (dt, *J* = 11.3 Hz, 3.7 Hz, 1H), 3.56 (td, *J* = 11.3, 2.8 Hz, 1H), 3.47 – 3.38 (m, 2H), 2.62 – 2.57 (m, 1H), 2.06 (ddd, *J* = 11.2, 8.4, 2.8 Hz, 1H), 1.95 – 1.77 (m, 3H), 1.56 – 1.49 (m, 1H), 1.42 (m, 1H), 1.11 (s, 12H), 0.89 (m, 1H), 0.62 (dd, *J* = 16.4, 5.9 Hz, 1H). **^{13}C NMR (126 MHz, $CDCl_3$)** δ 210.1, 136.9, 128.4, 126.6, 126.3, 126.2, 106.4, 83.1, 79.9, 65.3, 64.7, 44.3, 38.5, 36.7, 34.6, 32.5, 32.5, 25.3, 24.6. **^{11}B NMR (128 MHz, $CDCl_3$)** δ 34.3. **HRMS (APCI)** *m/z* calcd for $C_{24}H_{34}BO_3$ [M+H]$^+$ 381.2600; found 381.2601.

(±)-4,4,5,5-Tetramethyl-2-(((1*R*,2*S*,3*R*)-2-methyl-2-phenyl-3-(phenylethynyl)cyclopropyl) methyl)-1,3,2-dioxaborolane (9).

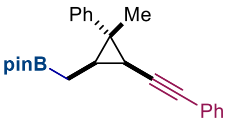

From cyclopropane **4c** (52.0 mg, 0.15 mmol) following the general procedure described above compound **9** was obtained along with 15% of starting boronate. After purification by flash column chromatography (silica gel; Et_2O/Hexane 1-3%), compound **9** (36.4 mg, 0.09 mmol) with a trace amount of starting boronate was obtained in 64% yield as a colorless oil.

Data for **9**: R_f 0.40 (2% Et_2O/Hexane). **^{1}H NMR (400 MHz, $CDCl_3$)** δ 7.47 – 7.42 (m, 4H), 7.32 – 7.27 (m, 5H), 7.21 – 7.16 (m, 1H), 1.94 (d, *J* = 8.8 Hz, 1H), 1.68 – 1.62 (m, 1H), 1.43 (s, 3H), 1.30 (s, 6H), 1.29 (s, 6H), 1.22 (m, 1H), 1.10 (dd, *J* = 16.9, 8.9 Hz, 1H). **^{13}C NMR (101 MHz, $CDCl_3$)** δ 148.3, 131.8, 131.6, 128.4, 128.4, 128.32, 128.26, 127.6, 127.5, 126.2, 124.4, 88.8, 83.4, 81.7, 31.4, 25.7, 25.1, 25.1, 25.0, 19.3, 17.9. **^{11}B NMR (128 MHz, $CDCl_3$)** δ 34.6. **HRMS (APCI)** *m/z* calcd for $C_{25}H_{30}BO_2$ $[M+H]^+$ 373.2340; found 373.2338.

(±)-2-(((1*R*,2*S*,3*S*)-3-Allyl-2-isopropyl-2-phenylcyclopropyl)methyl)-4,4,5,5-tetramethyl-1,3,2-dioxaborolane (10).

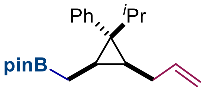

From cyclopropane **2c** (44.0 mg, 0.13 mmol) following the general procedure described above, compound **10** (35.4 mg, 0.10 mmol) was obtained in 80% yield as a colorless oil, after purification by flash column chromatography (silica gel; CH_2Cl_2/Hexane, 10-30%).

Data for **10**: R_f 0.40 (2% Et_2O/Hexane). **^{1}H NMR (400 MHz, $CDCl_3$)** δ 7.32-7.14 (m, 5H), 6.05 – 5.92 (m, 1H), 5.15 (dd, *J* = 17.1, 1.8 Hz, 1H), 5.01 (dd, *J* = 10.2, 1.8 Hz, 1H), 2.36 (m, 1H), 2.16 (m, 1H), 1.75 – 1.68 (m, 1H), 1.34 – 1.26 (m 13H), 1.11 – 0.93 (m, 3H), 0.85 – 0.84 (m, 6H). **^{13}C NMR (101 MHz, $CDCl_3$)** δ 145.8, 139.0, 132.0, 127.2, 125.7, 114.5, 83.3, 36.4, 28.1, 26.5, 25.7, 25.08, 25.06, 21.9, 20.6, 20.2. [note: the carbon attached to boron was not observed due to quadrupole broadening caused by the ^{11}B nucleus]. **^{11}B NMR (128 MHz, $CDCl_3$)** δ 33.9. **HRMS (APCI)** *m/z* calcd for $C_{22}H_{34}BO_2$ $[M+H]^+$ 341.2650; found 341.2649.

General Procedure for Oxidation and Benzoylation

The corresponding boronate (1 equiv) was dissolved in THF (10 mL x mmol), and the mixture was cooled to 0 °C, followed by the addition of an aqueous solution of NaOH 1 M (2-12 equiv) and a solution of H_2O_2 30% (w/v) (4-24 equiv). After consumption of starting material monitored by TLC (30-60 min), a solution of sodium sulfite (1 M) was added. The reaction mixture was extracted with EtOAc (x3), washed with brine, dried over Na_2SO_4, filtered and the solvent was evaporated to afford the oxidized crude product that was used in the next step without further purification. The crude product was taken up in another flask together with 3,5-dibromobenzoic acid (1.5 equiv), EDC (1.5 equiv) and DMAP (0.2 equiv). The solids were dissolved in CH_2Cl_2 (2 mL x mmol) and the reaction was stirred overnight at room temperature. An excess of 20% of reagents was added to the reaction if the starting alcohol was not fully consumed. Then,

water was added and the phases separated. The aqueous phase was washed with CH_2Cl_2. The organic phase was washed with a saturated solution of $NaHCO_3$, dried over Na_2SO_4, filtered and evaporated under vacuum. The residue was purified by flash chromatography on silica gel to afford the corresponding product.

(±)-(1*R*,2*S*,3*R*)-3-Allyl-2-methyl-2-phenylcyclopropyl 3,5-dibromobenzoate (11).

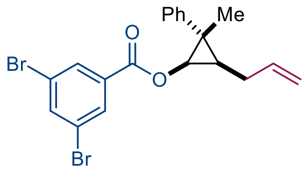

From cyclopropane **2a** (30.0 mg, 0.1 mmol) following the general procedure described above [NaOH 1 M (2 equiv), H_2O_2 30% (w/v) (4 equiv)] compound **11** (42.8 mg, 0.095 mmol) was obtained in 95% yield as a white solid, after purification by flash column chromatography (silica gel; CH_2Cl_2/Hexane, 5-20%).

Data for **11**: R_f 0.80 (10% Et_2O/Hexane). Mp = 60-61 °C **^{1}H NMR (400 MHz, $CDCl_3$)** δ 8.11 (d, *J* = 1.7 Hz, 2H), 7.89 (t, *J* = 1.3 Hz, 1H), 7.43 (d, *J* = 7.8 Hz, 2H), 7.34 (t, *J* = 7.1 Hz, 2H), 7.24 – 7.21 (m, 1H), 6.02 (m, 1H), 5.22 (dd, *J* = 17.2, 1.7 Hz, 1H), 5.11 (dd, *J* = 10.1, 1.5 Hz, 1H), 4.46 (d, *J* = 7.3 Hz, 1H), 2.41 (ddd, *J* = 7.6, 6.2, 1.7 Hz, 2H), 1.56 – 1.51 (m, 1H), 1.34 (s, 3H). **^{13}C NMR (101 MHz, $CDCl_3$)** δ 165.0, 146.1, 138.7, 137.0, 133.2, 131.4, 128.8, 128.1, 126.7, 123.34, 115.7, 60.8, 28.4, 27.5, 25.9, 15.2. **HRMS (APCI)** *m/z* calcd for $C_{20}H_{19}Br_2O_2$ $[M+H]^+$ 450.9727; found 450.9726.

(±)-(1*R*,2*R*)-2-Allyl-8,11-dioxadispiro[3.2.4^7.2^4]tridecan-1-yl 3,5-dibromobenzoate (12).

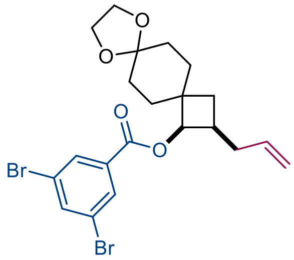

From cyclobutane **7c** (25.0 mg, 0.07 mmol) following the general procedure described above [NaOH 1 M (10 equiv), H_2O_2 30% (w/v) (20 equiv)] compound **12** (27.0 mg, 0.05 mmol) was obtained in 77% yield as an oil, after purification by flash column chromatography (silica gel; CH_2Cl_2/Hexane, 5-10%).

Data for **12**: R_f 0.60 (40% Et_2O/Hexane**). ^{1}H NMR (400 MHz, $CDCl_3$, COSY, NOESY)** δ 8.07 (s, 2H), 7.84 (s, 1H), 5.67 (m, 1H), 5.19 (d, *J* = 7.5 Hz, 1H), 4.98 – 4.91 (m, 2H), 3.91 (s, 4H), 2.77 – 2.67 (m, 1H), 2.36 – 2.28 (m, 1H), 2.20 – 2.13 (m, 1H), 1.92 – 1.80 (m, 2H), 1.77 – 1.72 (m, 2H), 1.66-1.55 (m, 6H) . **2D-NOESY (400 MHz, $CDCl_3$)** crosspoint between: 5.19 (d, *J* = 7.5 Hz, 1H) / 2.77 – 2.67 (m, 1H). **^{13}C NMR (101 MHz, $CDCl_3$, HSQC)** δ 163.8, 138.4, 136.0, 133.6, 131.4, 123.2, 115.9, 108.5, 77.8, 64.40, 64.38, 41.1, 34.8, 34.4, 33.9, 33.5, 31.4, 30.9, 30.2. **HRMS (APCI)** *m/z* calcd for $C_{21}H_{25}Br_2O_4$ $[M+H]^+$ 501.0095; found 501.0092.

General Procedure for Zweifel Olefination

To a cold solution (0 °C) of boronic ester (1 equiv) in anhydrous THF (10 mL x mmol) under argon was added dropwise 6 equiv of vinylmagnesium bromide (1 M in THF). The mixture was stirred from 0 °C to room temperature until full conversion (TLC, 3-4 h). Then, the mixture was cooled to 0 °C and 3 M solution

of NaOMe (5 equiv) in MeOH and a 0.5 M solution of I_2 (4 equiv) in MeOH were sequentially added. The mixture was stirred at room temperature until full conversion (TLC, 10-18 h). Then, a 1 M solution of $Na_2S_2O_4$ and H_2O were added and the mixture was extracted with CH_2Cl_2 (x3). The combined organic layers were dried ($MgSO_4$), filtered and evaporated under vacuum. The crude mixture was purified by flash column chromatography on silica gel to afford the corresponding product.

(±)-((1*R*,2*S*,3*R*)-2-Allyl-1-methyl-3-vinylcyclopropyl)benzene (13).

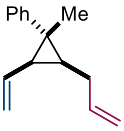

From cyclopropane **2a** (45.0 mg, 0.15 mmol) following the general procedure described above, compound **13** (24.7 mg, 0.12 mmol) was obtained in 83% yield as a colorless oil, after purification by flash column chromatography (silica gel; CH_2Cl_2/Hexane, 2-5%).

Data for **13**: R_f 0.90 (40% CH_2Cl_2/Hexane). **^{1}H NMR (400 MHz, $CDCl_3$)** δ 7.33 – 7.25 (m, 4H), 7.21 – 7.16 (m, 1H), 6.03 – 5.92 (m, 1H), 5.75 (dt, *J* = 16.9, 9.9 Hz, 1H), 5.28 (m, 1H), 5.24 – 5.12 (m, 2H), 5.06 (m, 1H), 2.32 (t, *J* = 6.7 Hz, 2H), 1.89 (t, *J* = 9.4 Hz, 1H), 1.50 – 1.44 (m, 1H), 1.37 (s, 3H). **^{13}C NMR (101 MHz, $CDCl_3$)** δ 149.3, 137.9, 134.4, 128.5, 127.4, 125.9, 116.7, 115.1, 31.6, 29.7, 29.2, 29.1, 16.3. **HRMS (APCI)** *m/z* calcd for $C_{15}H_{19}$ $[M+H]^+$ 199.1481; found 199.1479.

(±)-(1*R*,2*S*)-2-(2-Bromoallyl)-7,7-dimethyl-1-vinylspiro[3.5]nonane (14).

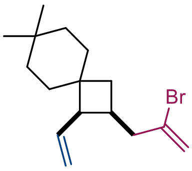

From cyclobutane **7b** (52.0 mg, 0.13 mmol) following the general procedure described above, compound **14** (26.9 mg, 0.09 mmol) was obtained in 69% yield as a colorless oil, after purification by flash column chromatography (silica gel; Hexane).

Data for **14**: R_f 0.40 (Hexane). **^{1}H NMR (400 MHz, $CDCl_3$)** δ 6.01 (dt, *J* = 16.8, 10.4 Hz, 1H), 5.48 (s, 1H), 5.35 (s, 1H), 5.05 (dd, *J* = 10.2, 2.3 Hz, 1H), 5.00 (dd, *J* = 16.8, 2.4 Hz, 1H), 2.75 – 2.65 (m, 1H), 2.61 (t, *J* = 8.4 Hz, 1H), 2.49 (dd, *J* = 14.4, 5.9 Hz, 1H), 2.39 (dd, *J* = 15.4, 9.2 Hz, 1H), 1.93 (t, *J* = 11.3 Hz, 1H), 1.59 – 1.54 (m, 2H), 1.48 (dd, *J* = 11.5, 8.8 Hz, 1H), 1.42 – 1.29 (m, 2H), 1.24 (t, *J* = 6.1 Hz, 2H), 1.17 – 1.04 (m, 2H), 0.86 (s, 3H), 0.84 (s, 3H). **^{13}C NMR (101 MHz, $CDCl_3$)** δ 135.8, 133.3, 117.0, 116.6, 50.5, 44.2, 39.4, 36.4, 35.9, 35.4, 34.5, 31.4, 31.3, 29.7. **HRMS (APCI)** *m/z* calcd for $C_{16}H_{25}$ $[M-Br]^+$ 217.1951; found 217.1942.

References

1) Rössle, M.; Del Valle, D. J.; Krische, M. J. Synthesis of the Cytotrienin A Core via Metal Catalyzed C-C Coupling. *Org. Lett*. **2011**, 13, 1482–1485.
2) Tomita, R.; Koike, T.; Akita, M. Photoredox-catalyzed oxytrifluoromethylation of allenes: stereoselective synthesis of 2-trifluoromethylated allyl acetates. *Chem. Commun*. **2017**, *53*, 4681–4684.
3) Sridharan, V.; Fan, L.; Takizawa, S.; Suzuki, T.; Sasai, H. Pd(II)–SDP-catalyzed enantioselective 5-*exo-dig* cyclization of γ-alkynoic acids: application to the synthesis of functionalized dihydrofuran-2(3*H*)-ones containing a chiral quaternary carbon center. *Org. Biomol. Chem.* **2013**, *11*, 5936–5943.
4) Szyling, J.; Szymańska, A.; Walkowiak, J. Selective synthesis of boron-substituted enynes via a one-pot diboration/protodeboration sequence. *Chem. Commun*. **2023**, *59*, 9541–9544.
5) Banerjee, A.; Yamamoto, H. Direct N–O bond formation via oxidation of amines with benzoyl peroxide. *Chem. Sci*. **2019**, *10*, 2124–2129.
6) Huang, R.-Z.; Lau, K. K.; Li, Z.; Liu, T.-L.; Zhao, Y. Rhodium-Catalyzed Enantioconvergent Isomerization of Homoallylic and Bishomoallylic Secondary Alcohols. *J. Am. Chem. Soc*. **2018**, *140*, 14647−14654.
7) Teresa, J.; Velado, M.; Fernández de la Pradilla, R.; Viso, A.; Lozano, B.; Tortosa, M. Enantioselective Suzuki cross-coupling of 1,2-diboryl cyclopropanes. *Chem. Sci*. **2023**, *14*, 1575-1581.
8) Novoa, L.; Trulli, L.; Parra, A.; Tortosa, M. Stereoselective Diboration of Spirocyclobutenes: A Platform for the Synthesis of Spirocycles with Orthogonal Exit Vectors. *Angew. Chem. Int. Ed*. **2021**, *60*, 11763 – 1176.
9) Fan, L.; Hao, J.; Yu, J.; Ma, X.; Liu, J.; Luan, X. Hydroxylamines as Bifunctional Single-Nitrogen Sources for the Rapid Assembly of Diverse Tricyclic Indole Scaffolds. *J. Am. Chem. Soc.* **2020**, *142*, 6698−6707.
10) Luo, X.-L.; Huang, M.-S.; Li, S.-S.; Jiang, Y.-S.; Chen, L.-N.; Li, S.-H.; Xia, P.-J. Photocatalytic Multicomponent 1,n-Carboimination with Alkyl Iodides and O-Benzoyl Oxime through EnT and XAT Processes. *Org. Lett.* **2023**, *25*, 6407−6412.
11) Bai, Z.; Zhu; S.; Hu, Y.; Yang, P.; Chu, X.; He, C.; Wang, H.; Chen, G. Synthesis of N-acyl sulfenamides via copper catalysis and their use as S-sulfenylating reagents of thiols. *Nature Commun*. **2022**, *13*, 6445–6453.
12) Parra, A.; Amenós, L.; Guisán-Ceinos, M.; López, A.; García Ruano, J. L.; Tortosa, M. Copper-Catalyzed Diastereo- and Enantioselective Desymmetrization of Cyclopropenes: Synthesis of Cyclopropylboronates. *J. Am. Chem. Soc*. **2014**, *136*, 15833−15836.

Proofs of stereochemistry

The retention of the configuration in the transmetalation has been demonstrated by performing the NOESY experiments of the products.

NOESY, **2a**

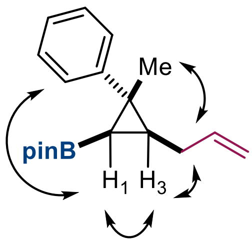

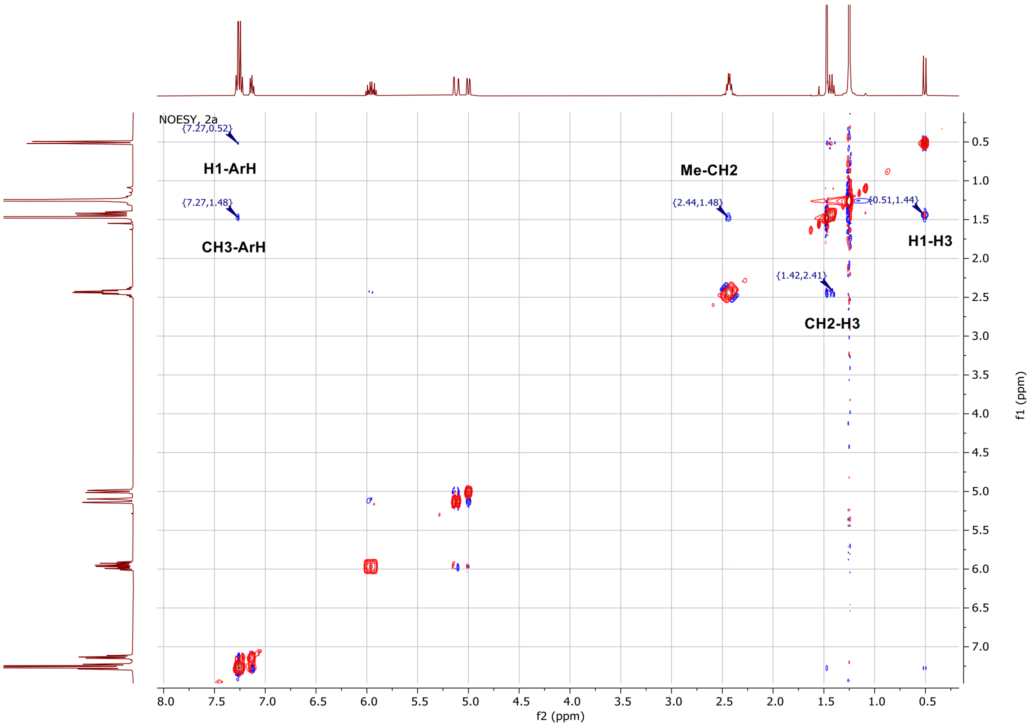

NOESY, **2k**

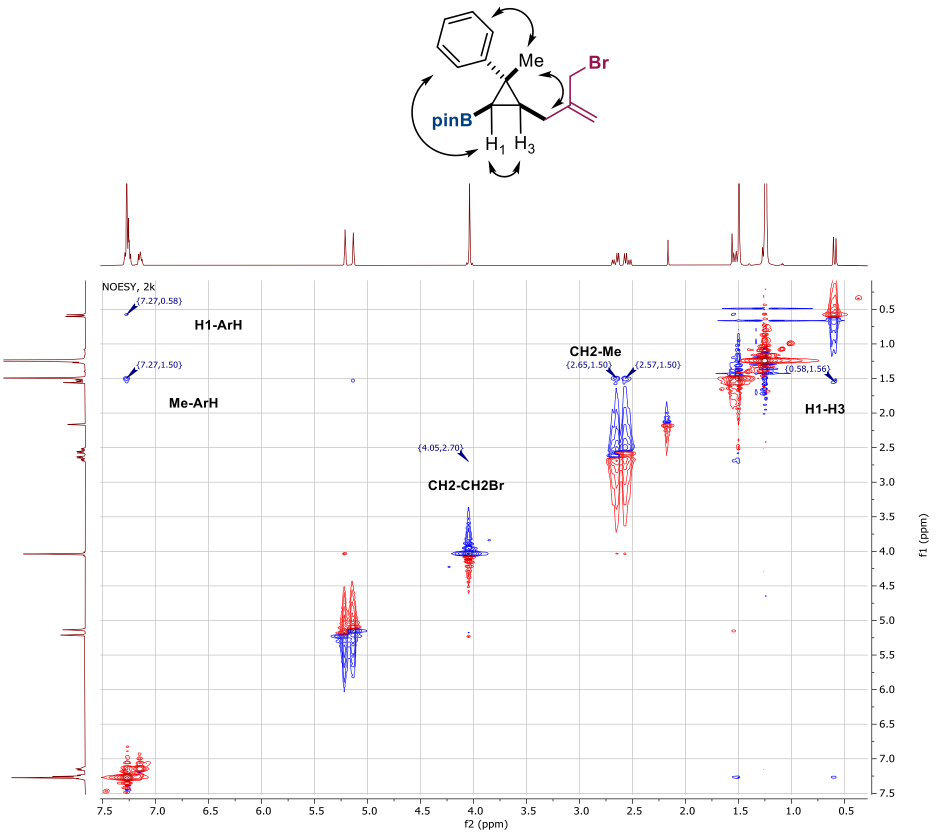

NOESY, **3a**

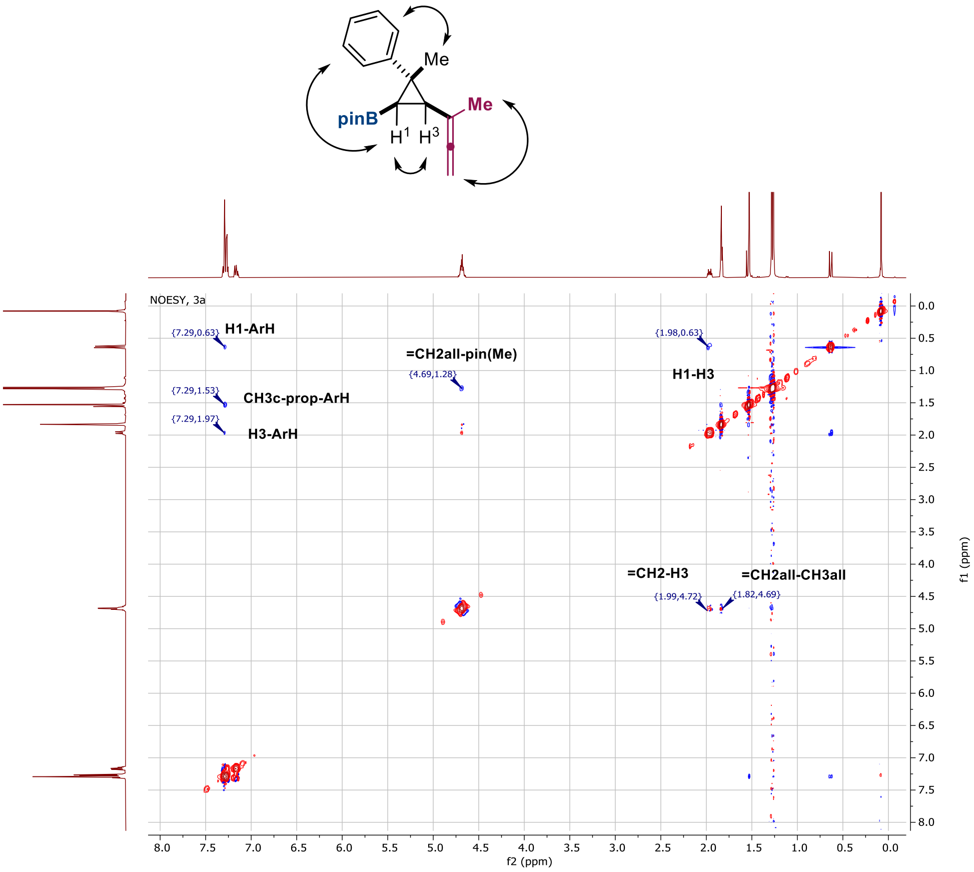

NOESY, **4c**

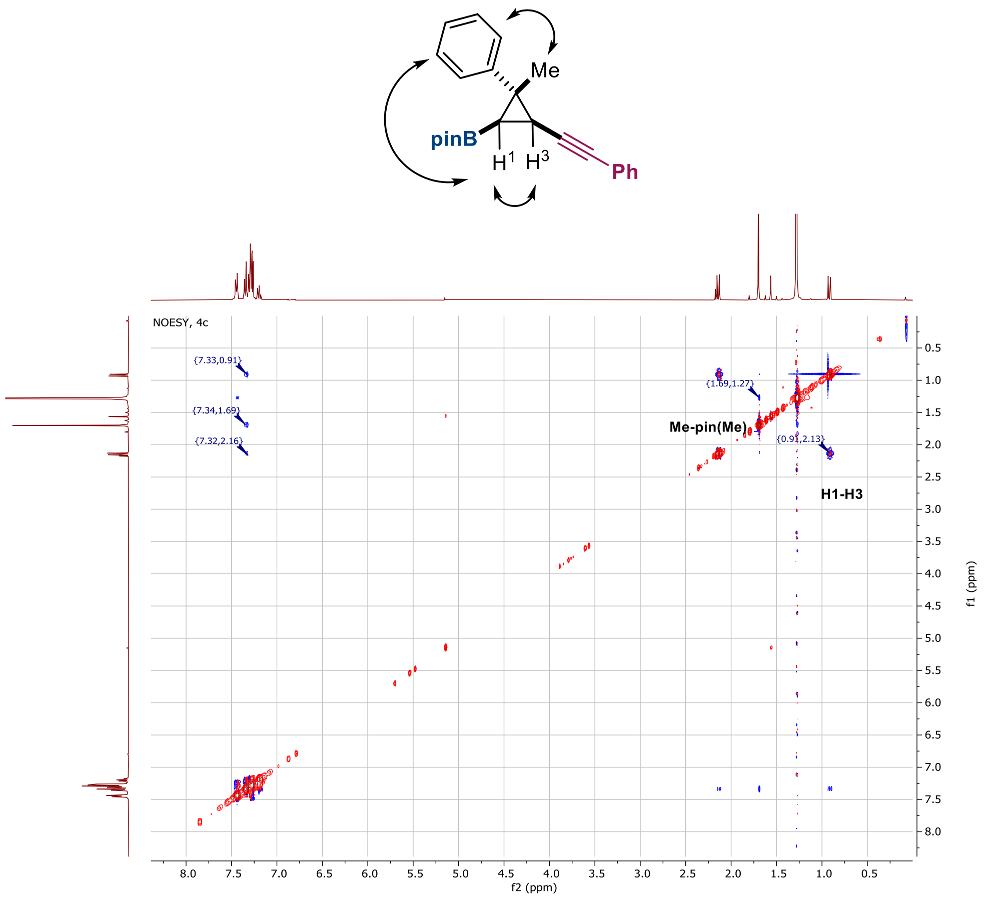

NOESY, **12**

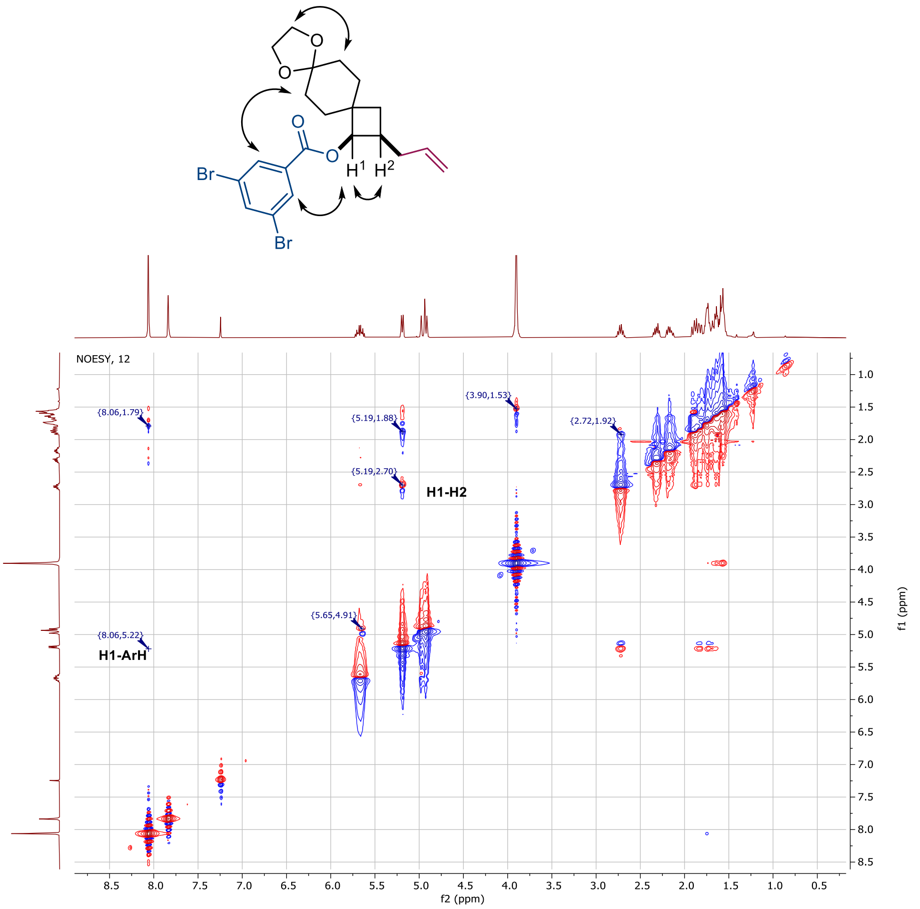

NMR data

Allylation of cyclopropyl bisboronates

Compound 2a

^{1}H NMR (400 MHz, $CDCl_3$)

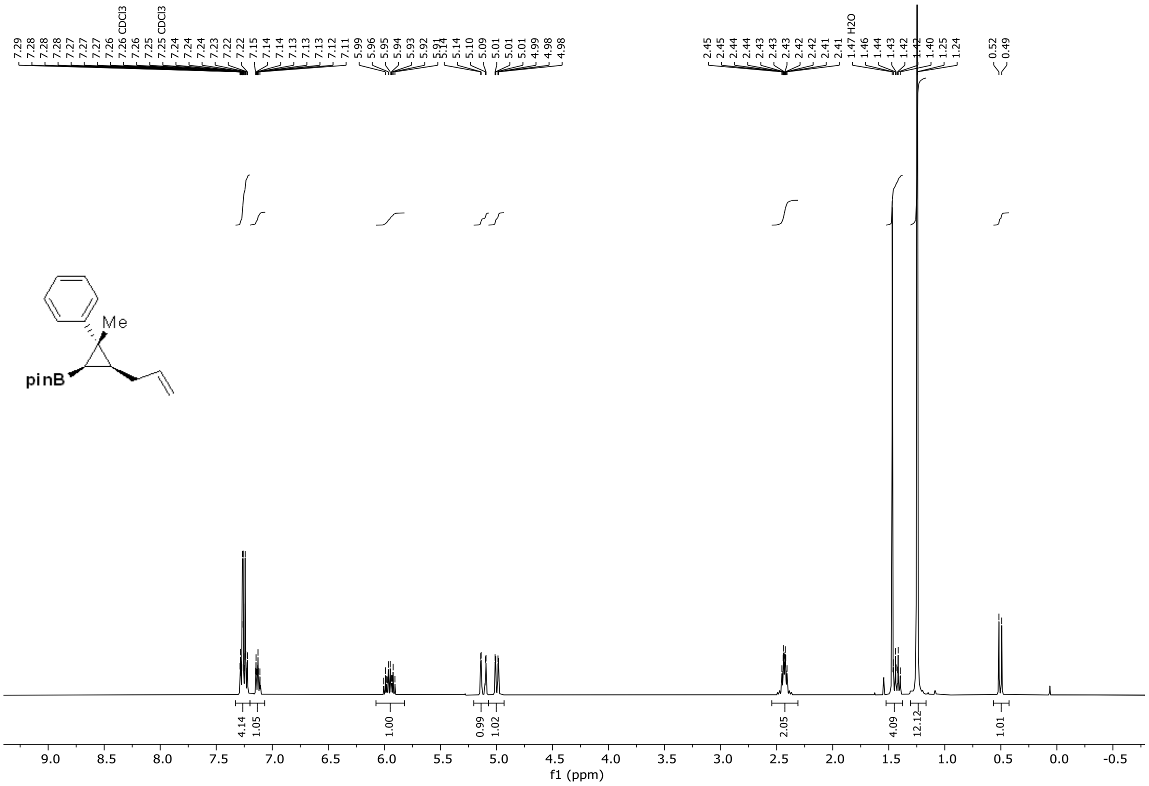

^{13}C NMR (101 MHz, $CDCl_3$)

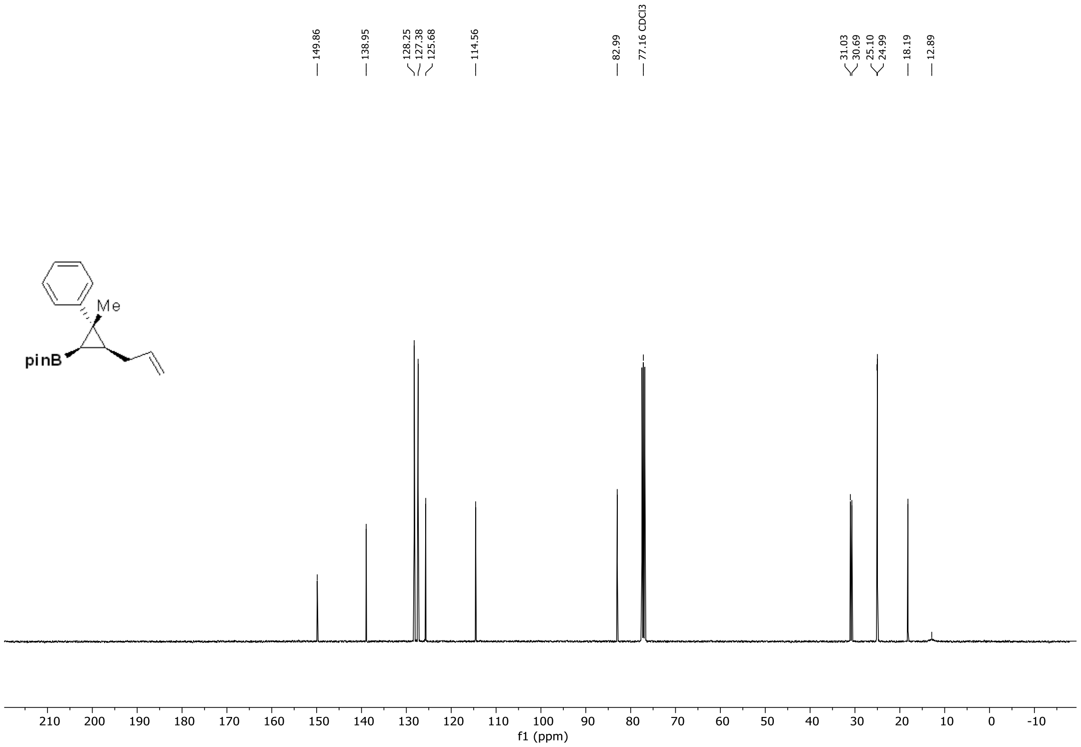

^{11}B NMR (128 MHz, $CDCl_3$)

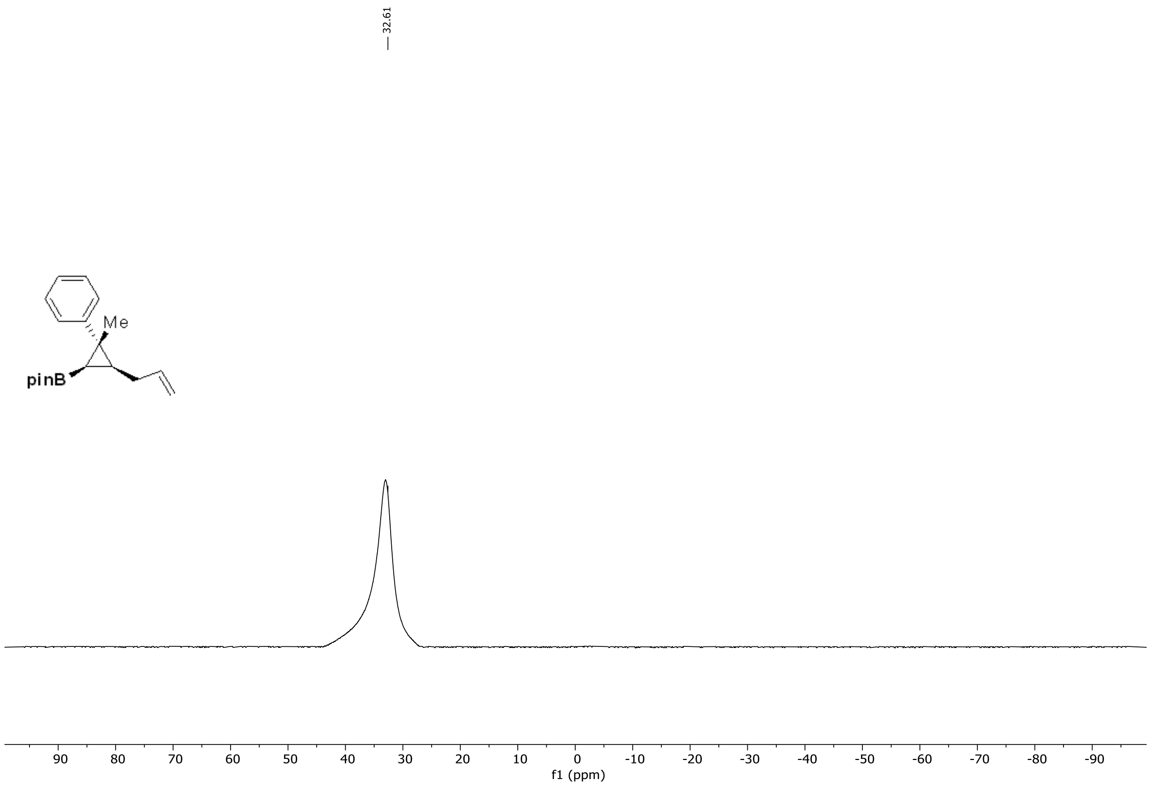

2D-NOESY (400 MHz, $CDCl_3$)

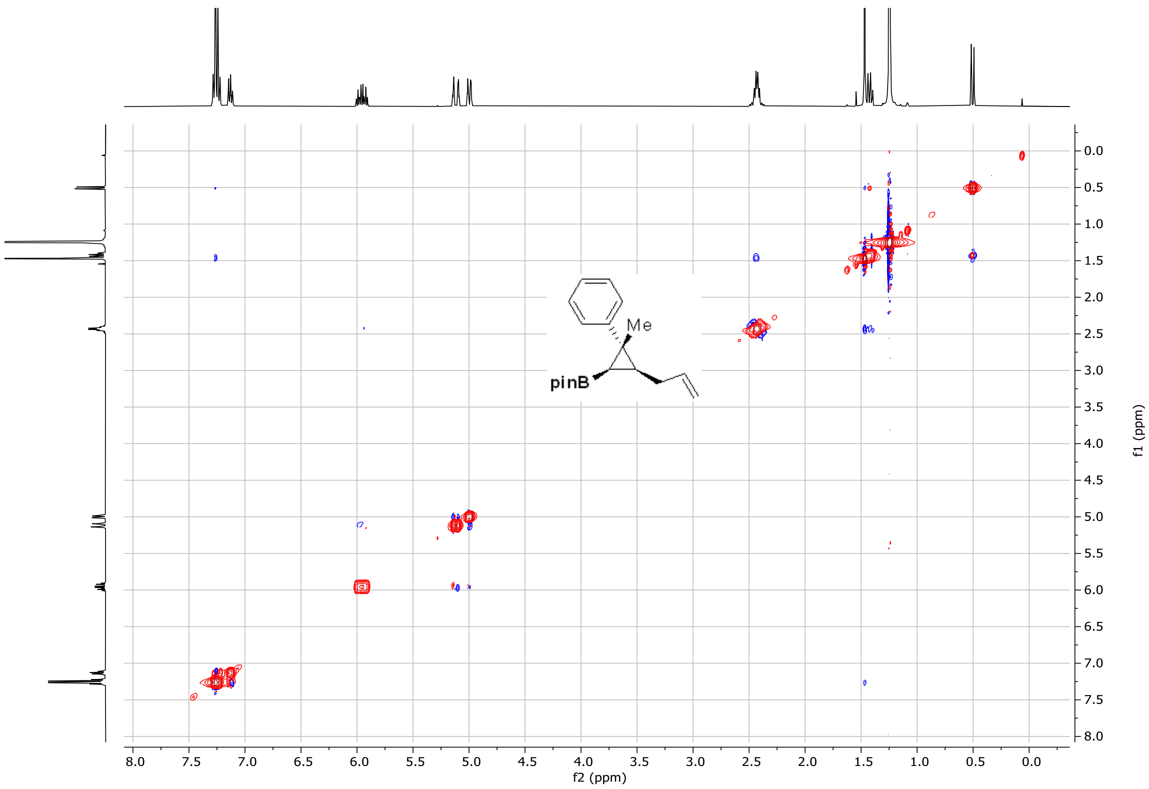

Compound 2b

^{1}H NMR (400 MHz, $CDCl_3$)

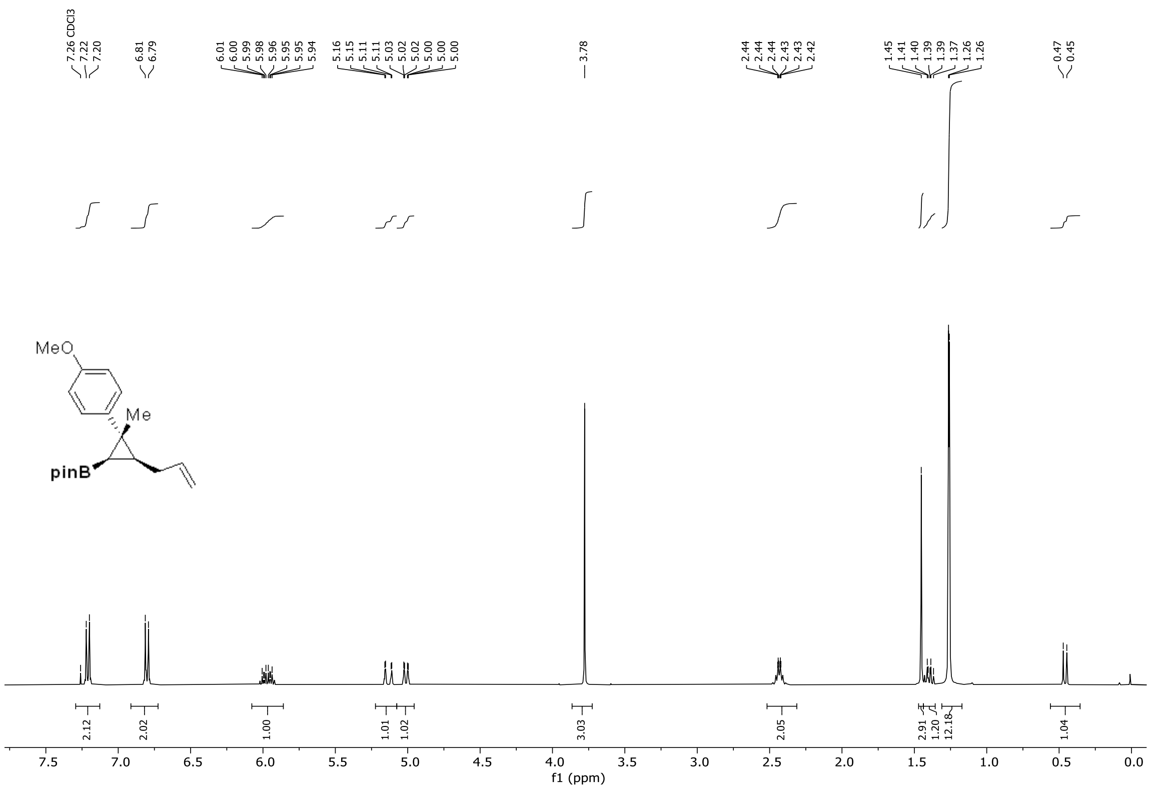

^{13}C NMR (101 MHz, $CDCl_3$)

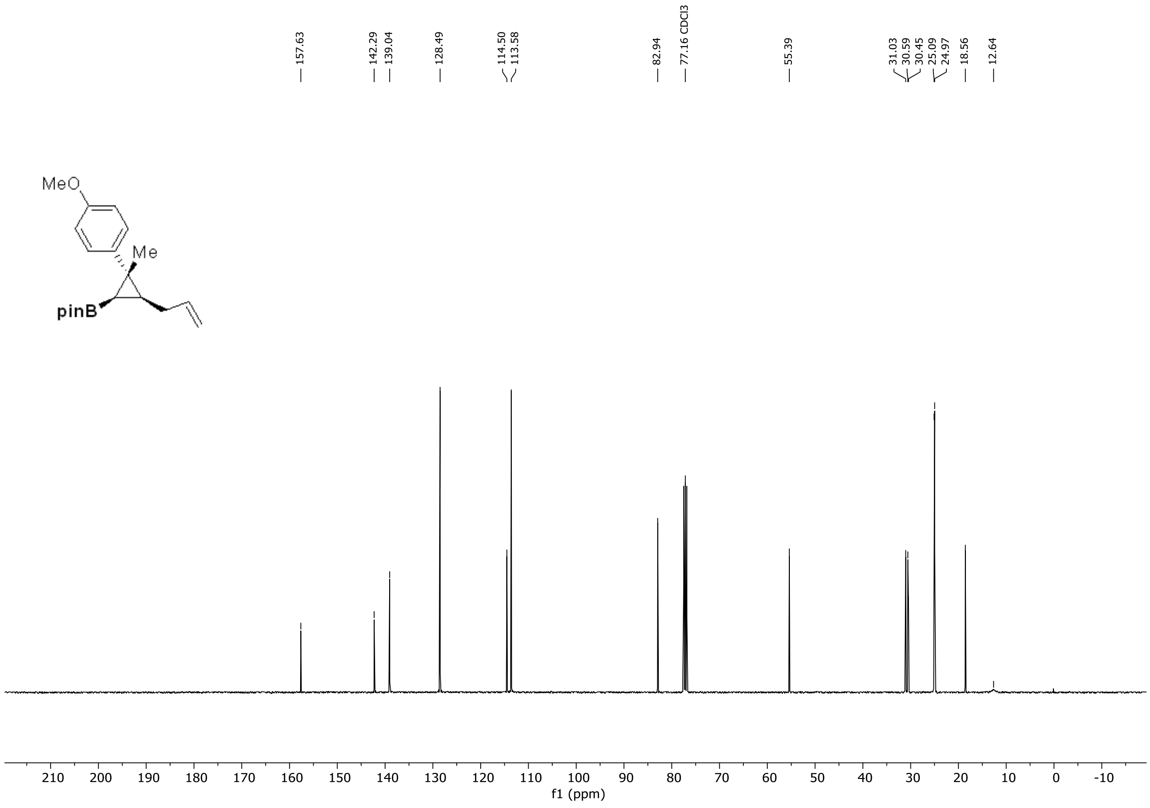

^{11}B NMR (128 MHz, $CDCl_3$)

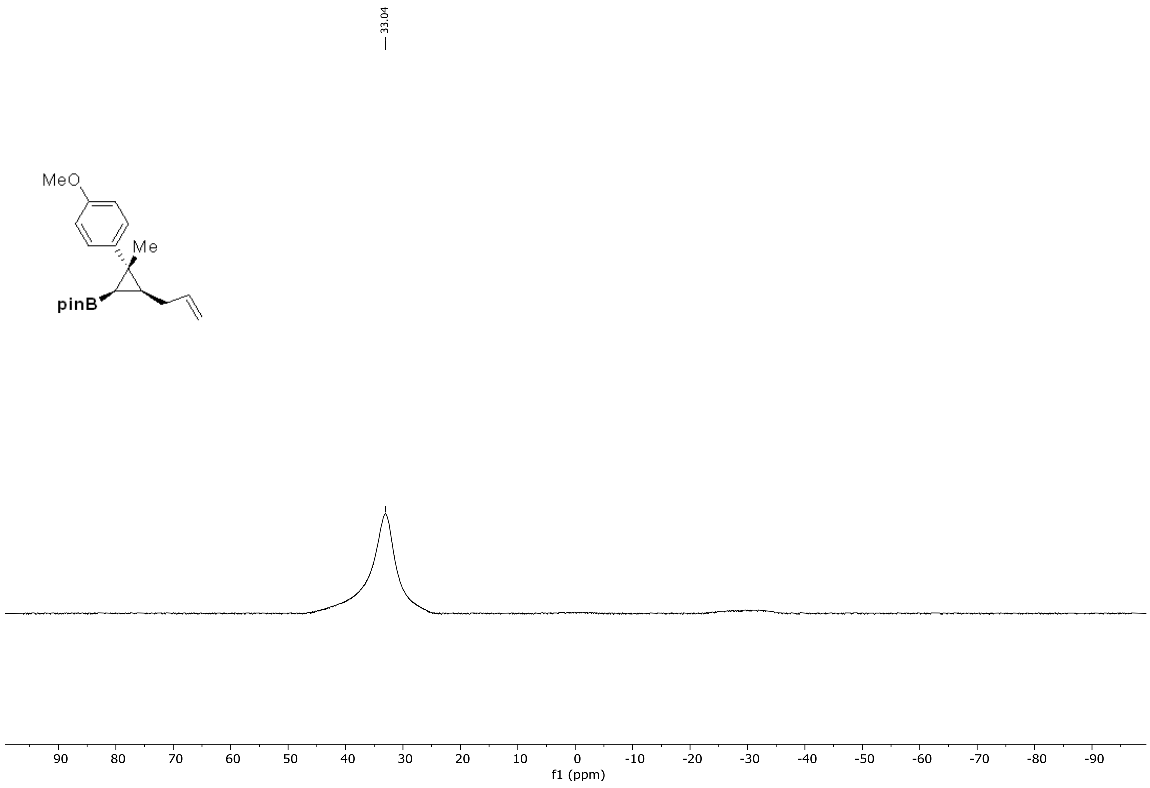

Compound 2c

^{1}H NMR (400 MHz, $CDCl_3$)

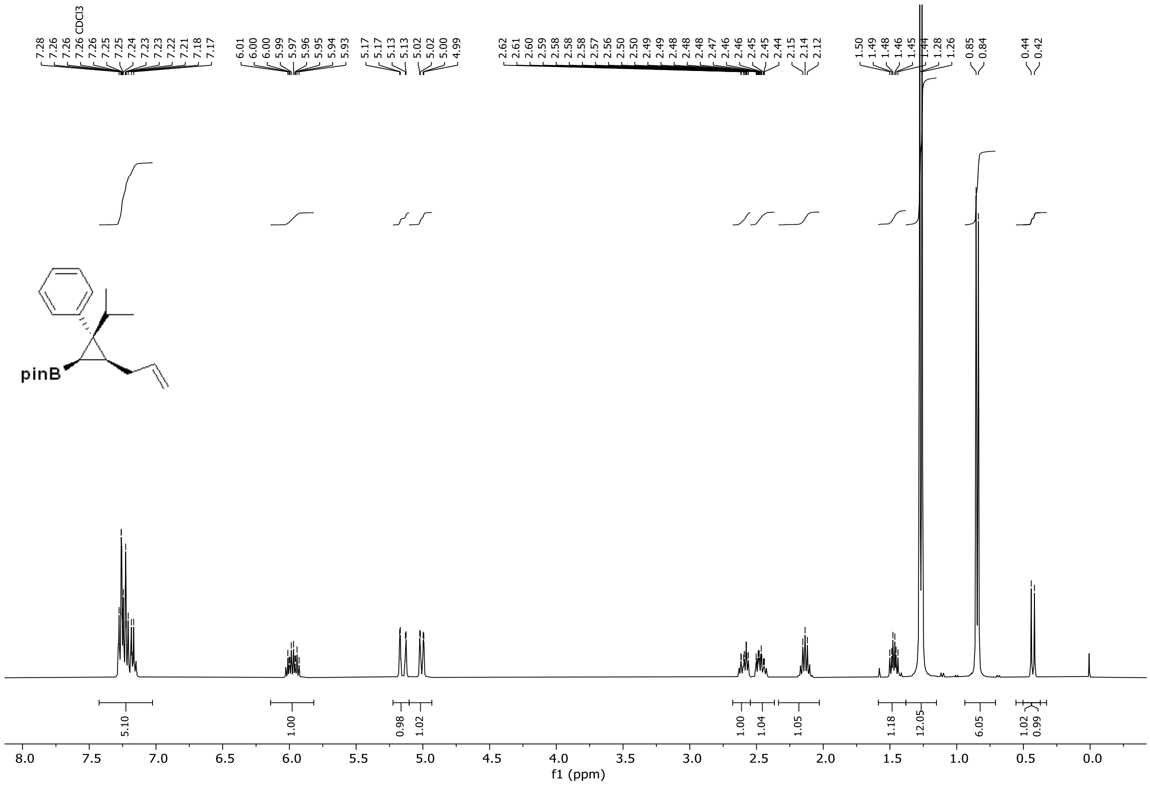

^{13}C NMR (101 MHz, $CDCl_3$)

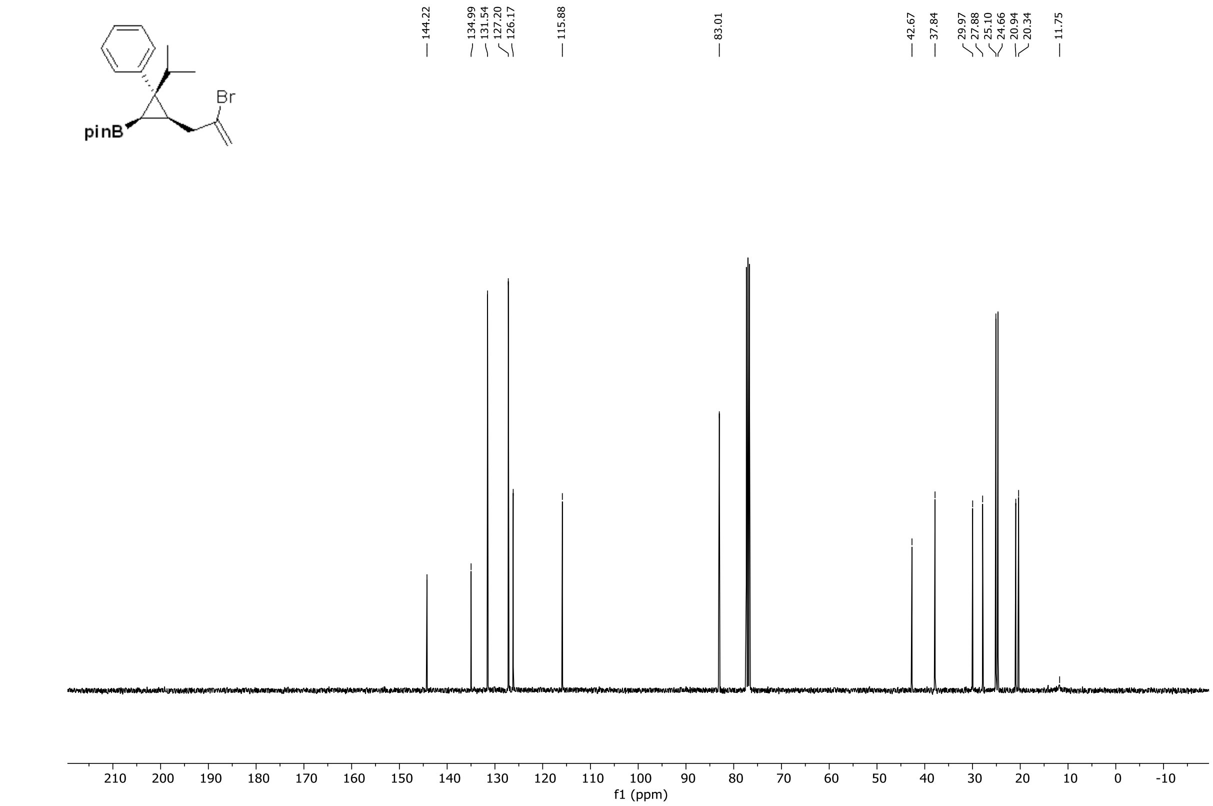

^{11}B NMR (128 MHz, $CDCl_3$)

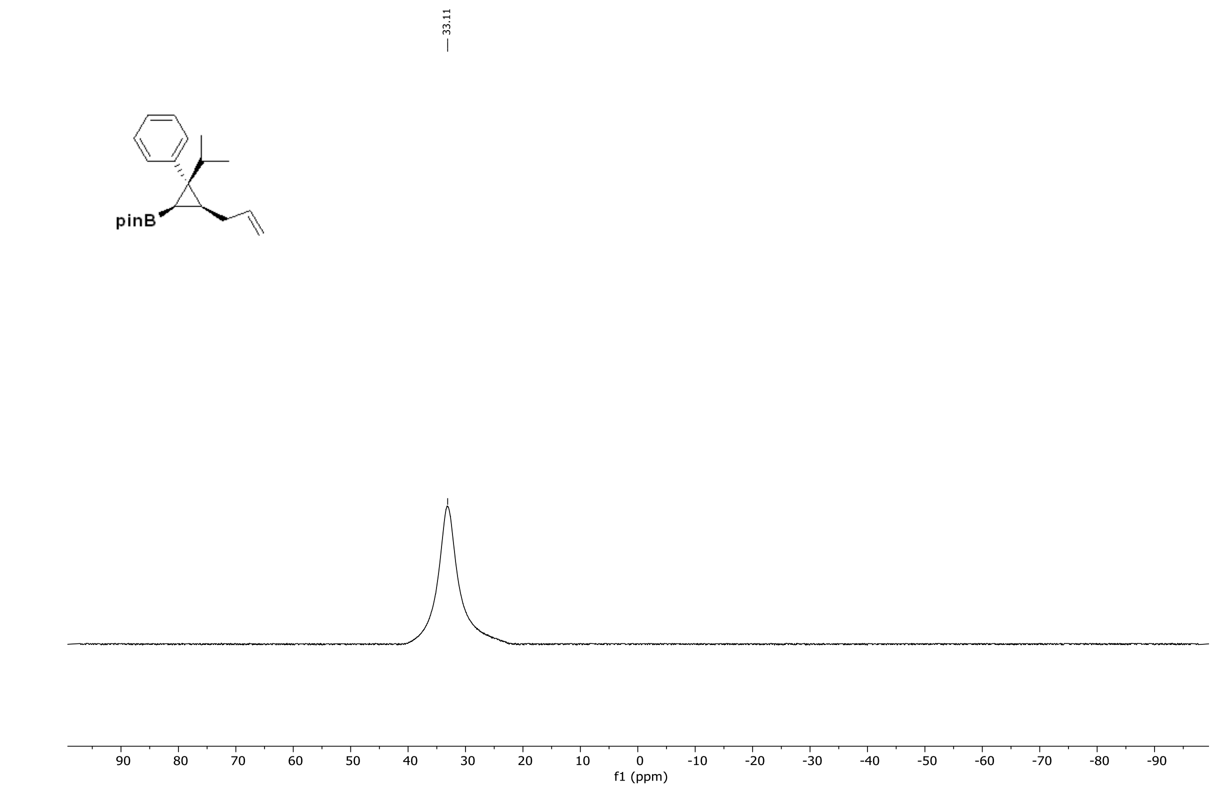

Compound 2d

^{1}H NMR (400 MHz, $CDCl_3$)

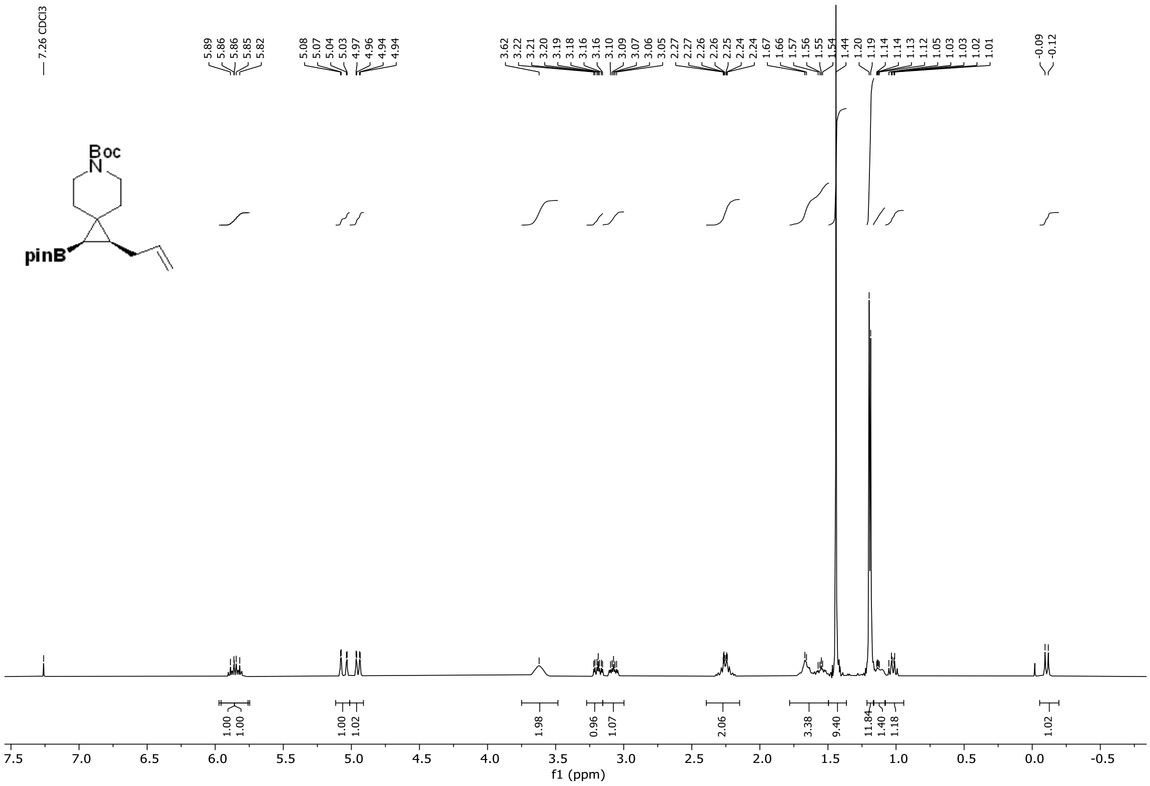

^{13}C NMR (101 MHz, $CDCl_3$)

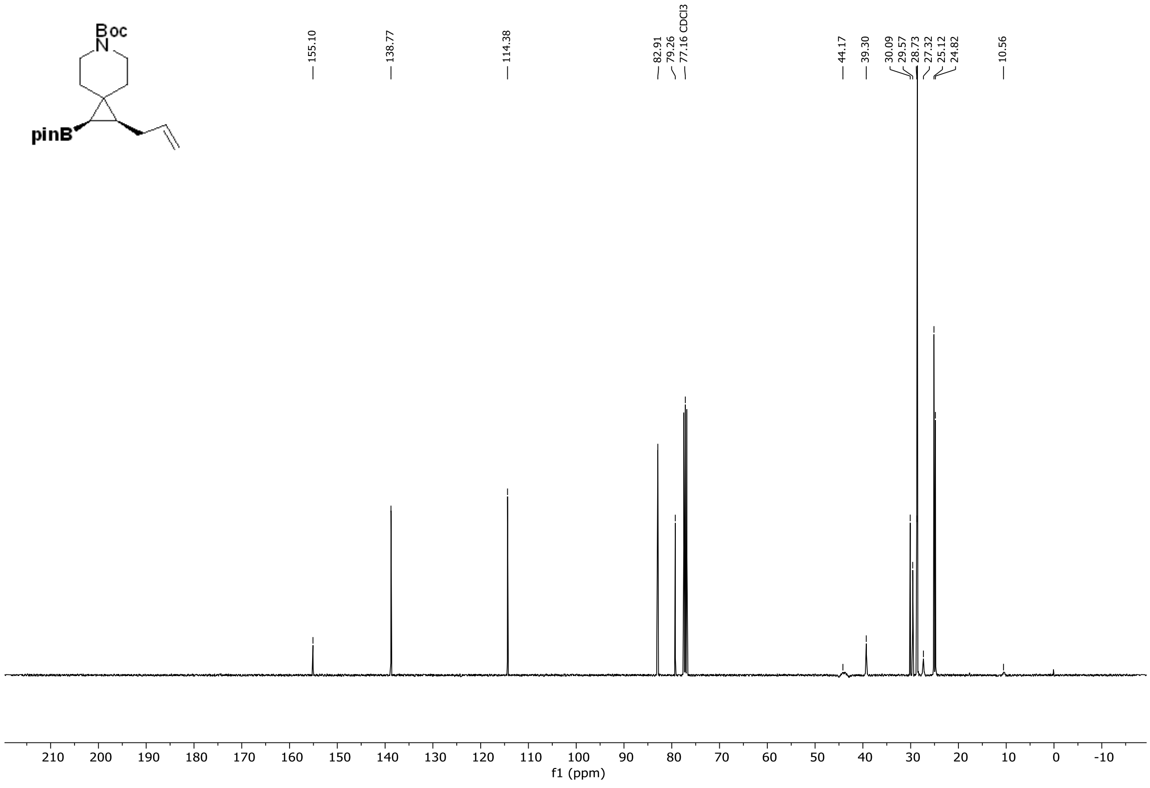

^{11}B NMR (128 MHz, $CDCl_3$)

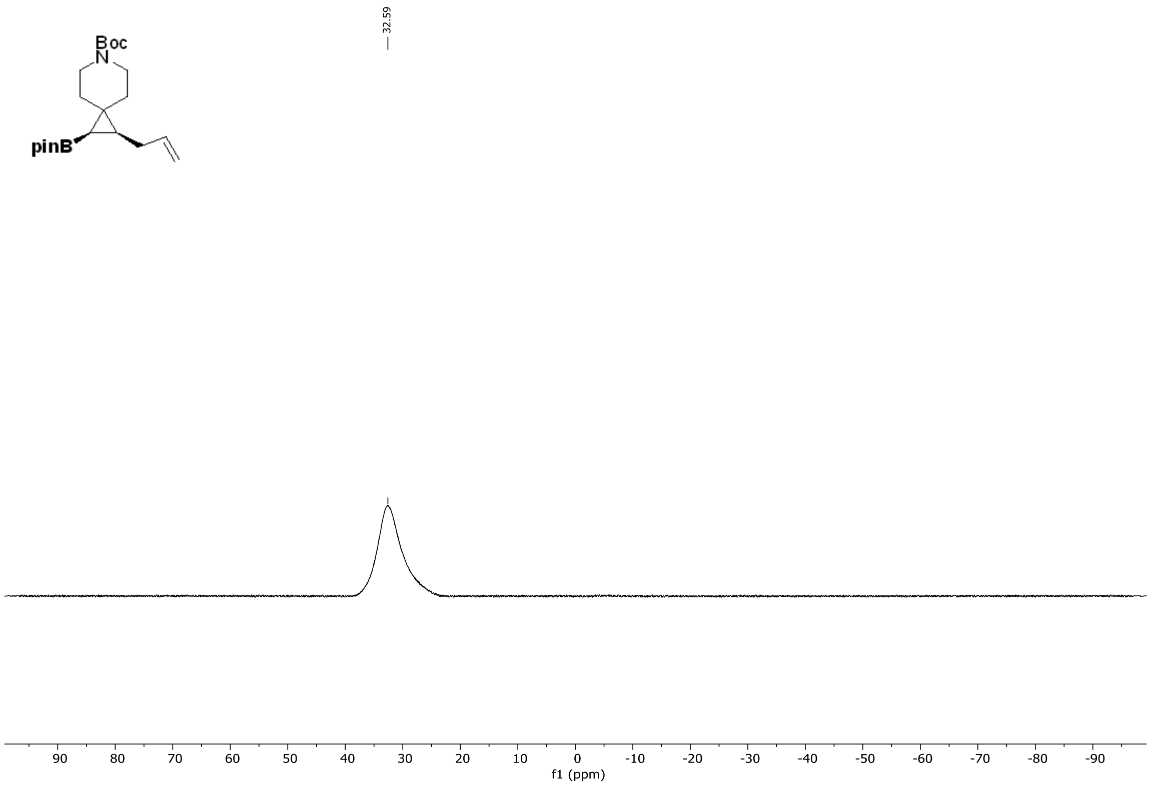

Compound 2e

^{1}H NMR (400 MHz, $CDCl_3$)

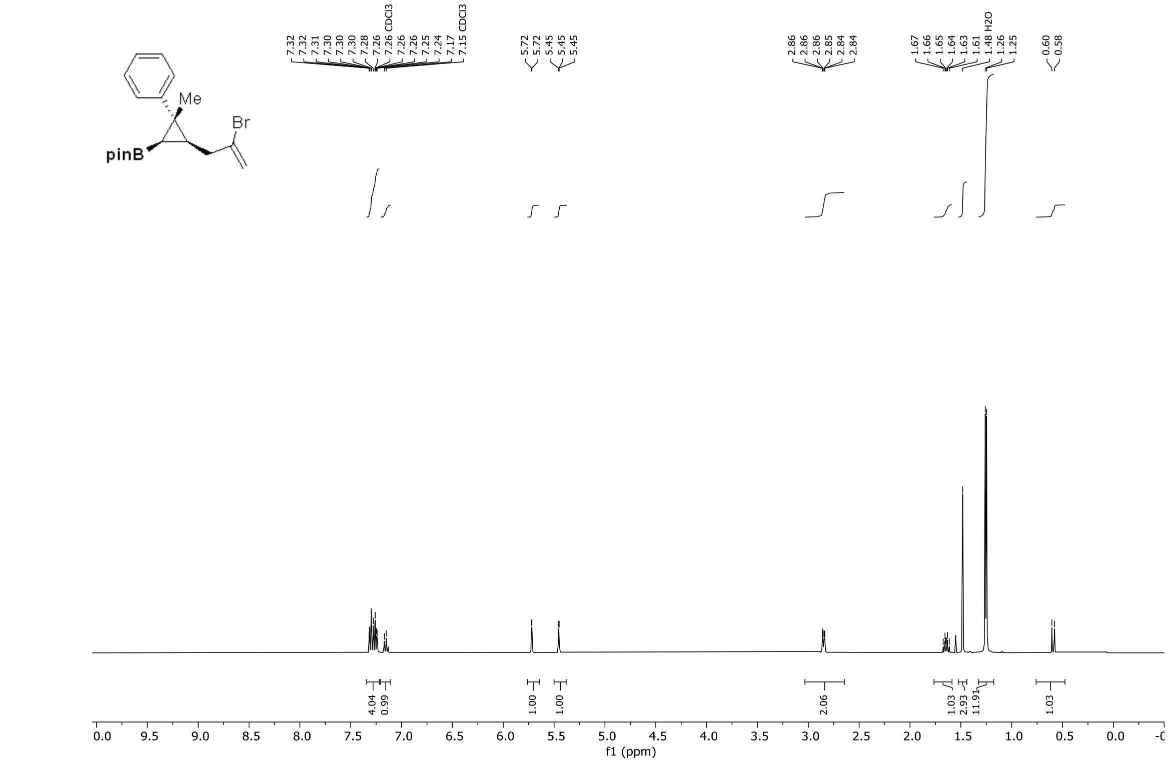

^{13}C NMR (101 MHz, $CDCl_3$)

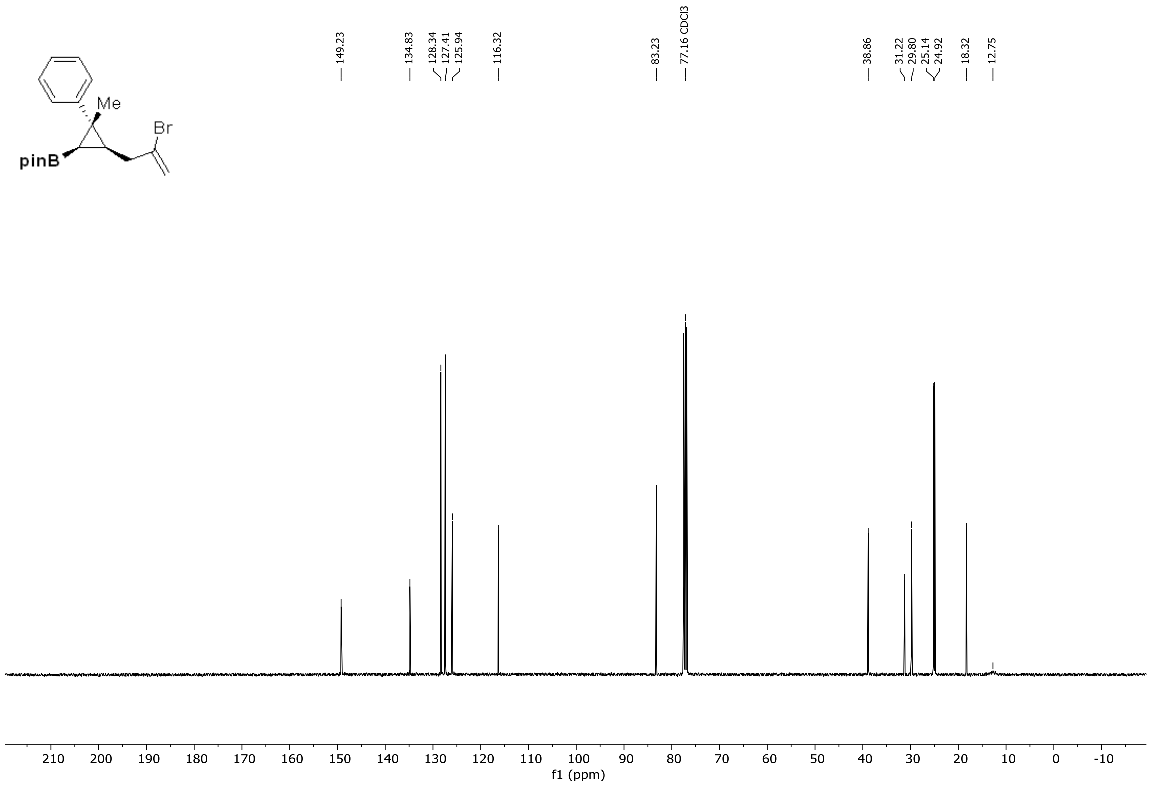

^{11}B NMR (128 MHz, $CDCl_3$)

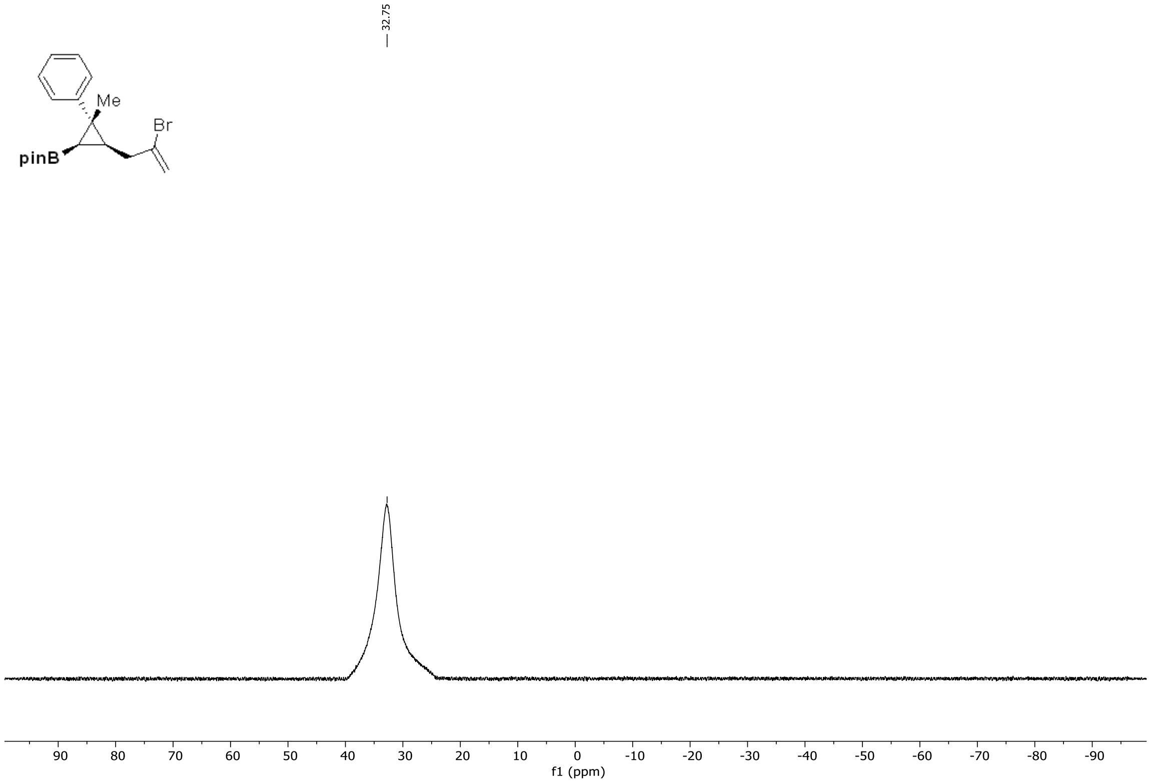

Compound 2f

^{1}H NMR (400 MHz, $CDCl_3$)

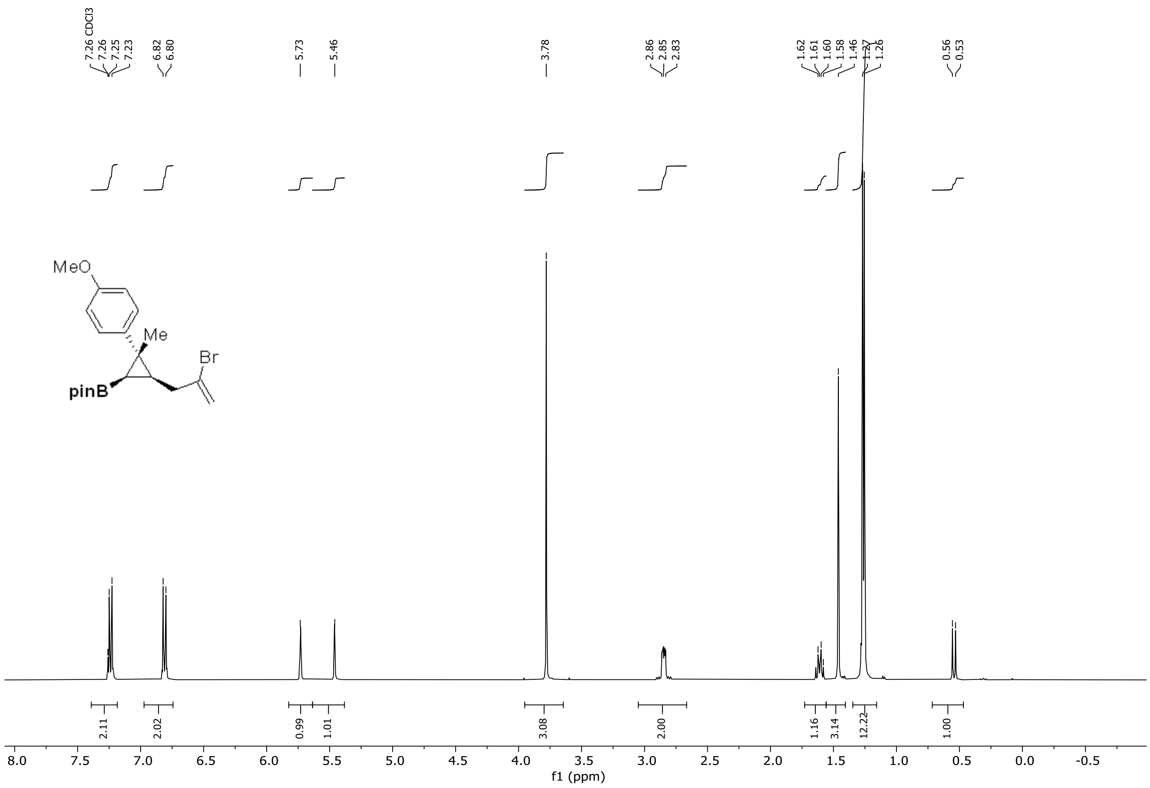

^{13}C NMR (101 MHz, $CDCl_3$)

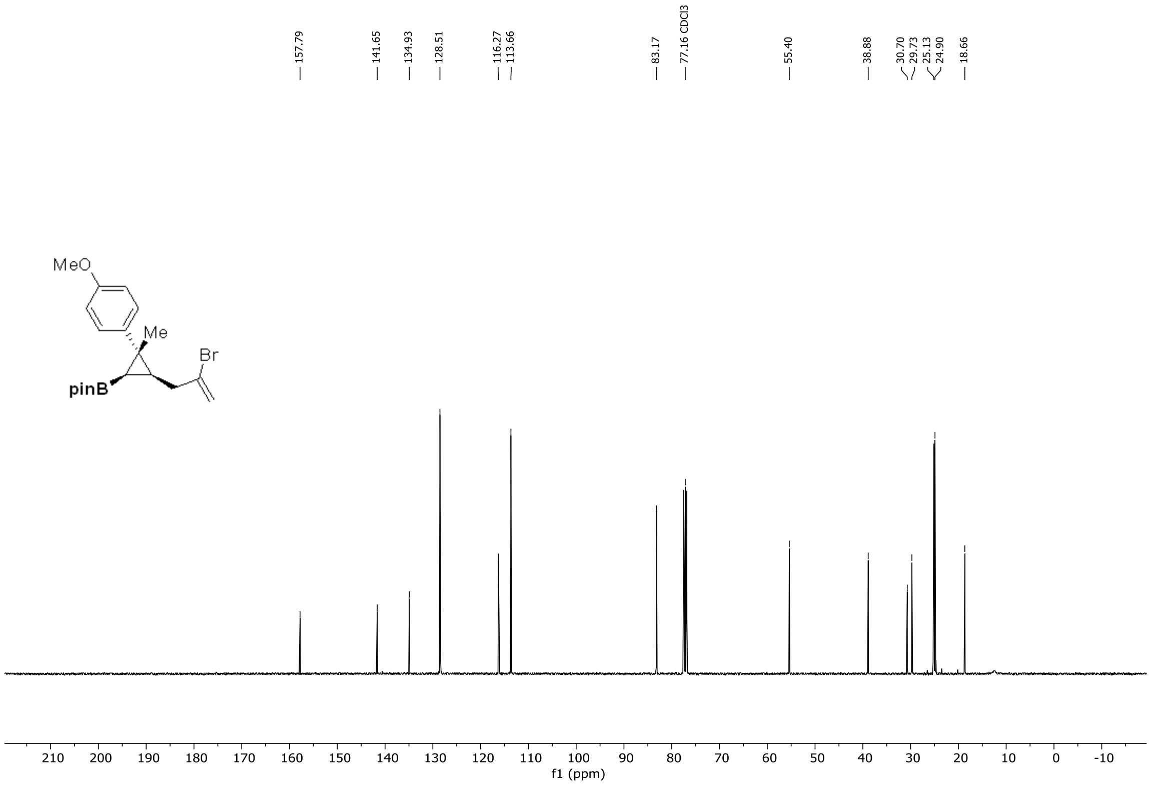

^{11}B NMR (128 MHz, $CDCl_3$)

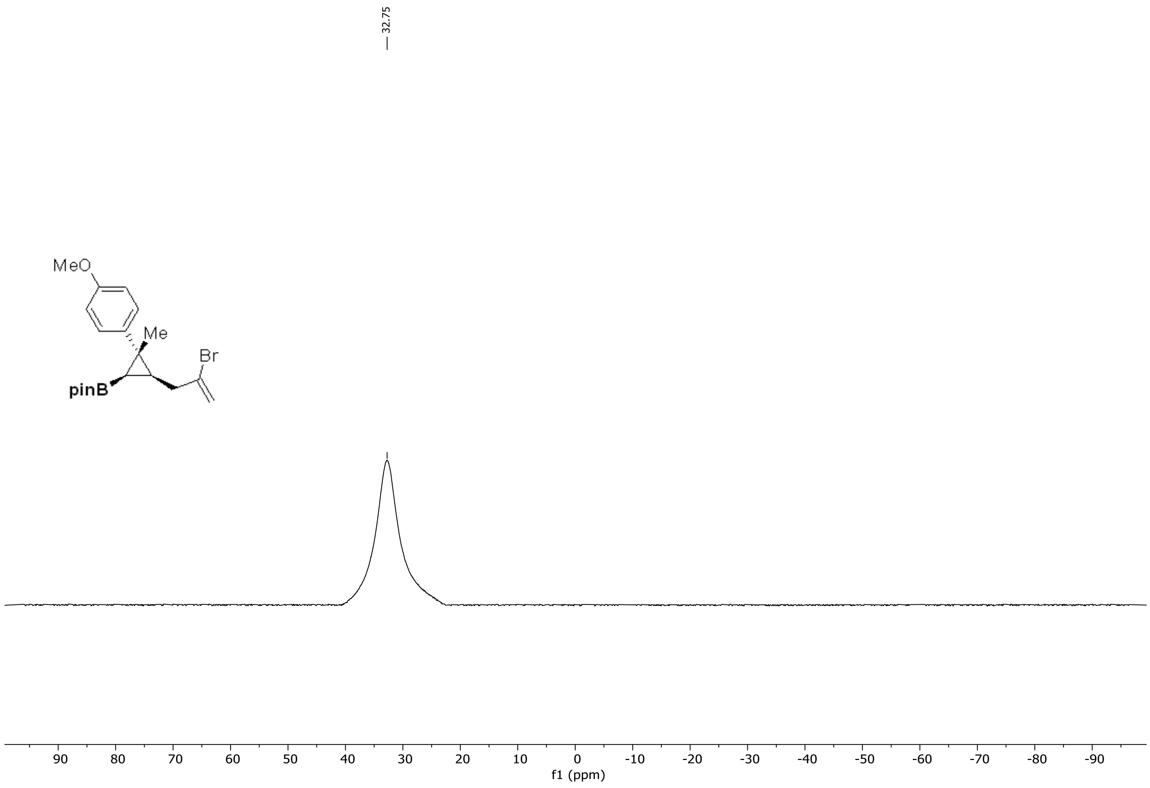

Compound 2g

^{1}H NMR (400 MHz, $CDCl_3$)

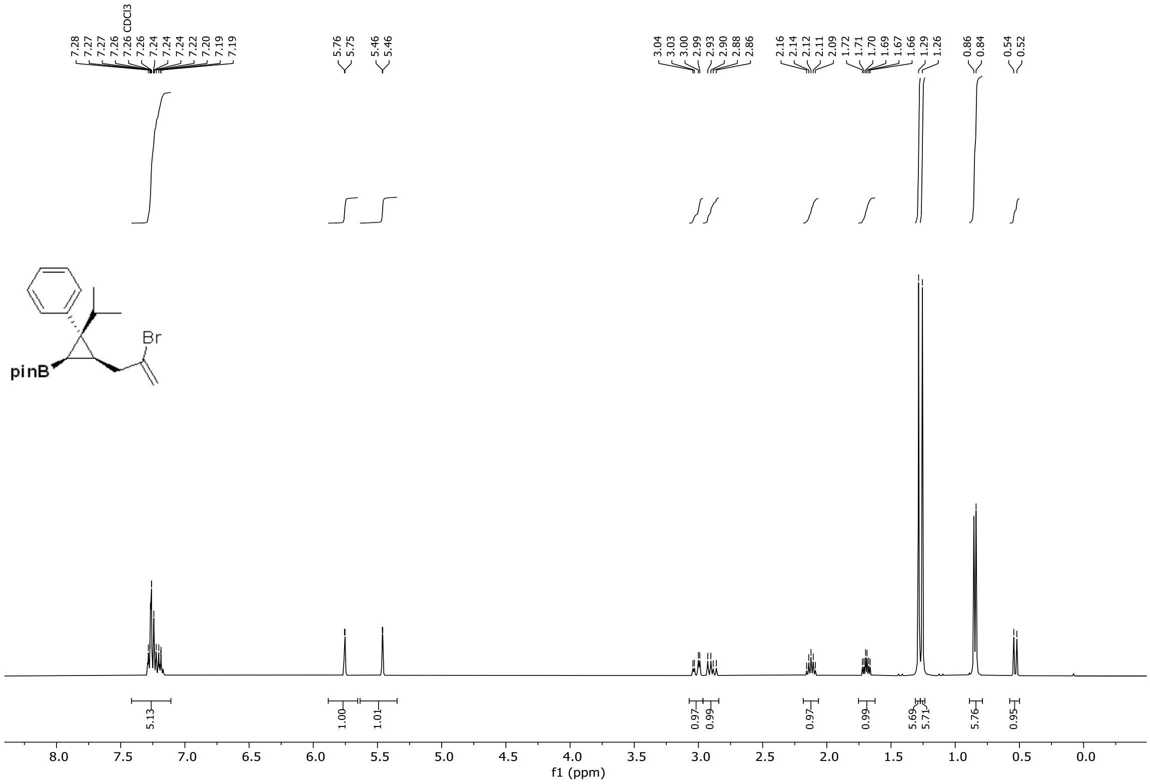

^{13}C NMR (101 MHz, $CDCl_3$)

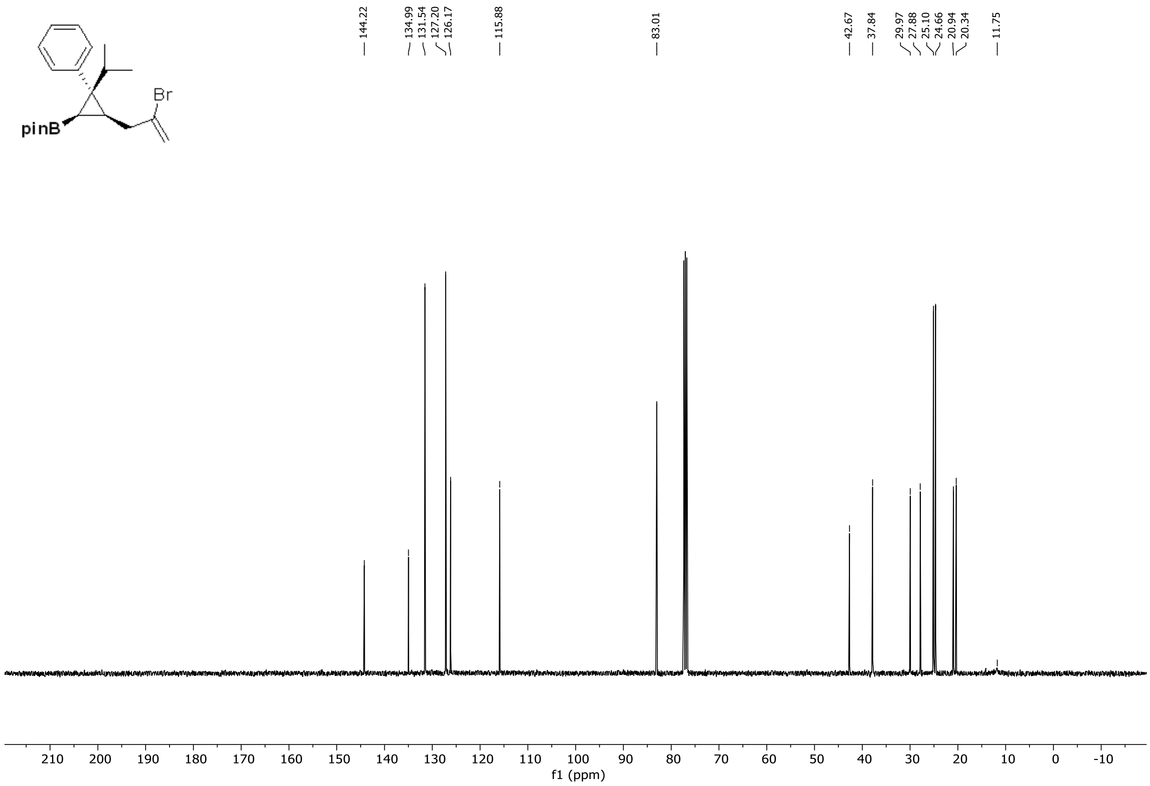

^{11}B NMR (128 MHz, $CDCl_3$)

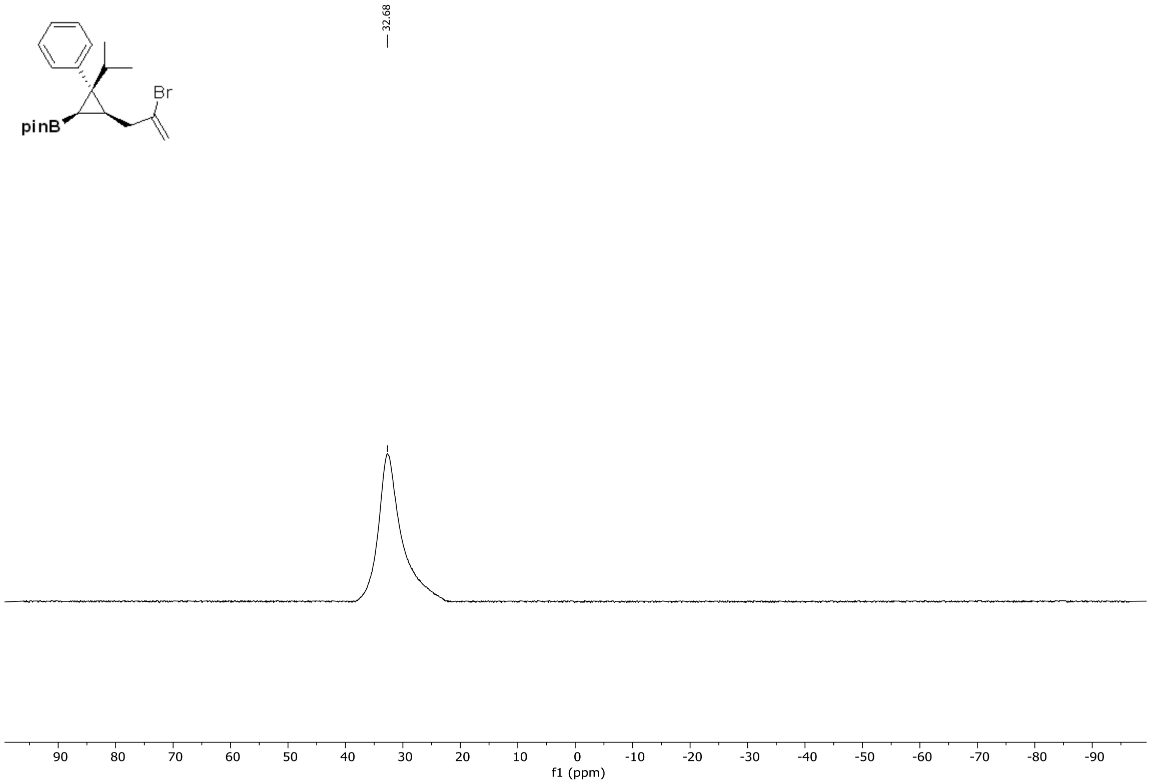

Compound 2h

^{1}H NMR (400 MHz, $CDCl_3$)

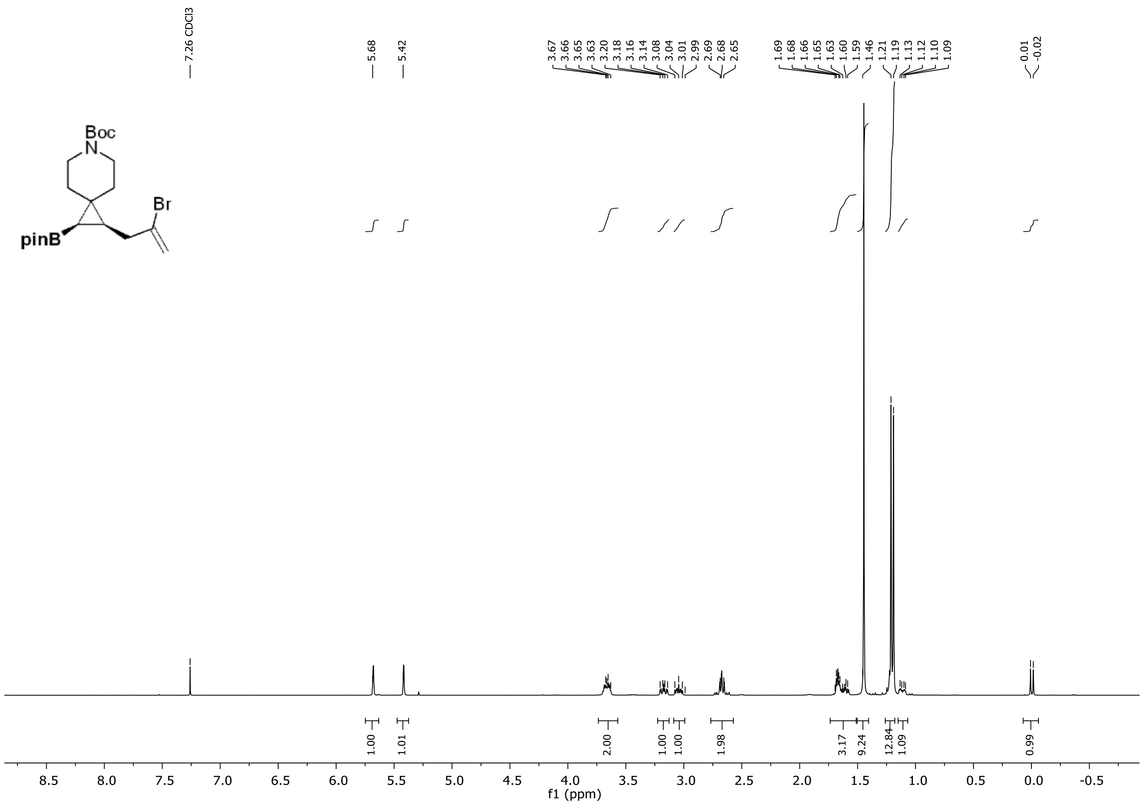

^{13}C NMR (101 MHz, $CDCl_3$)

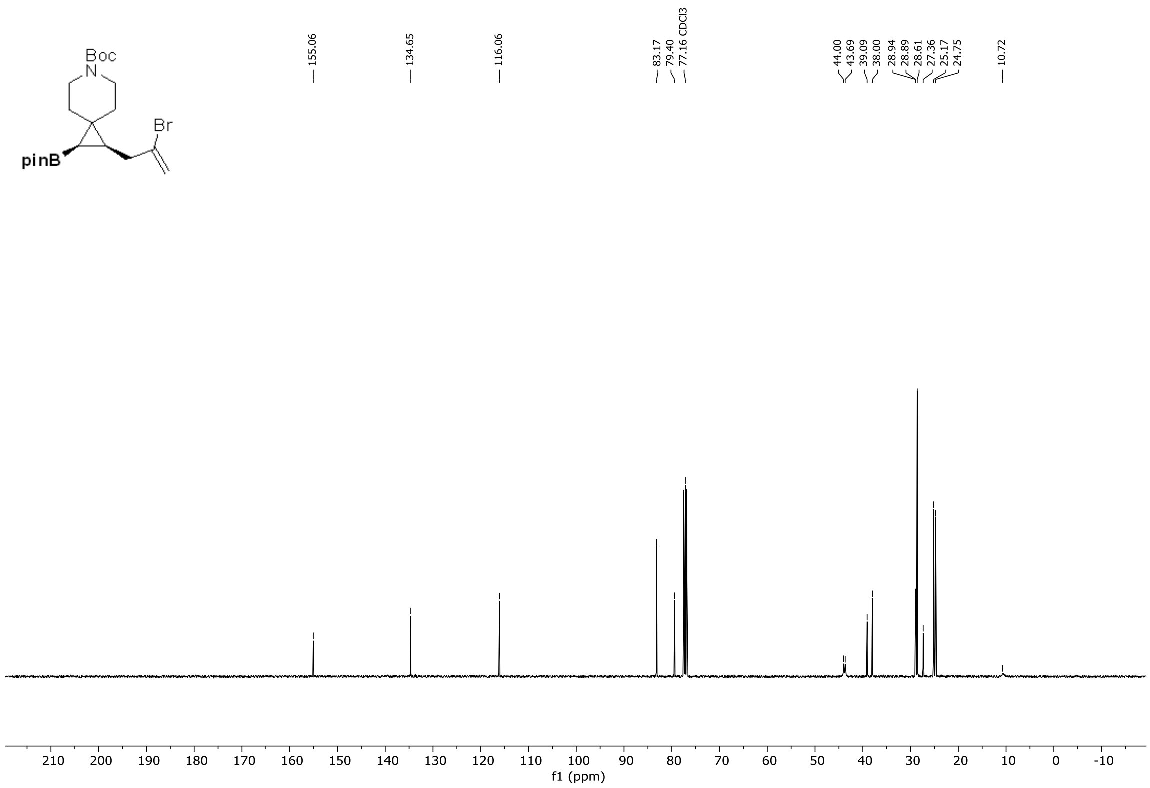

^{11}B NMR (128 MHz, $CDCl_3$)

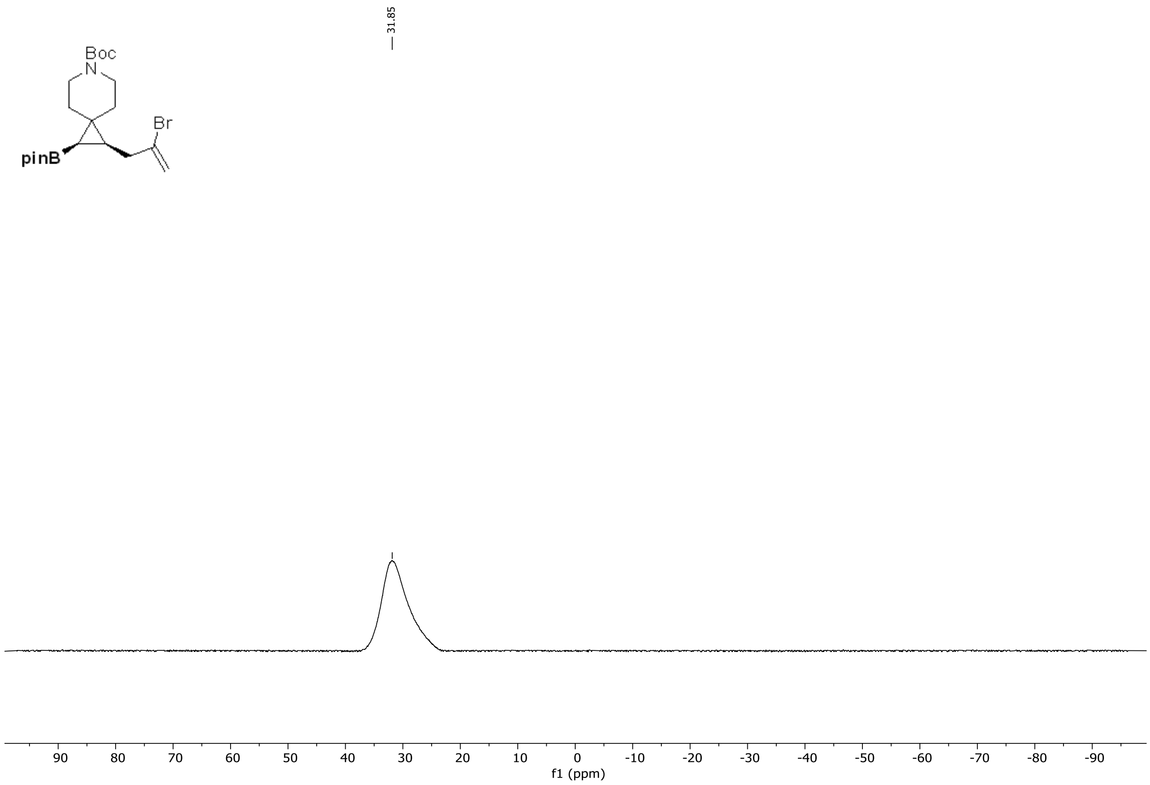

$^{13}C/^{1}H$ NMR (HSQC, $CDCl_3$)

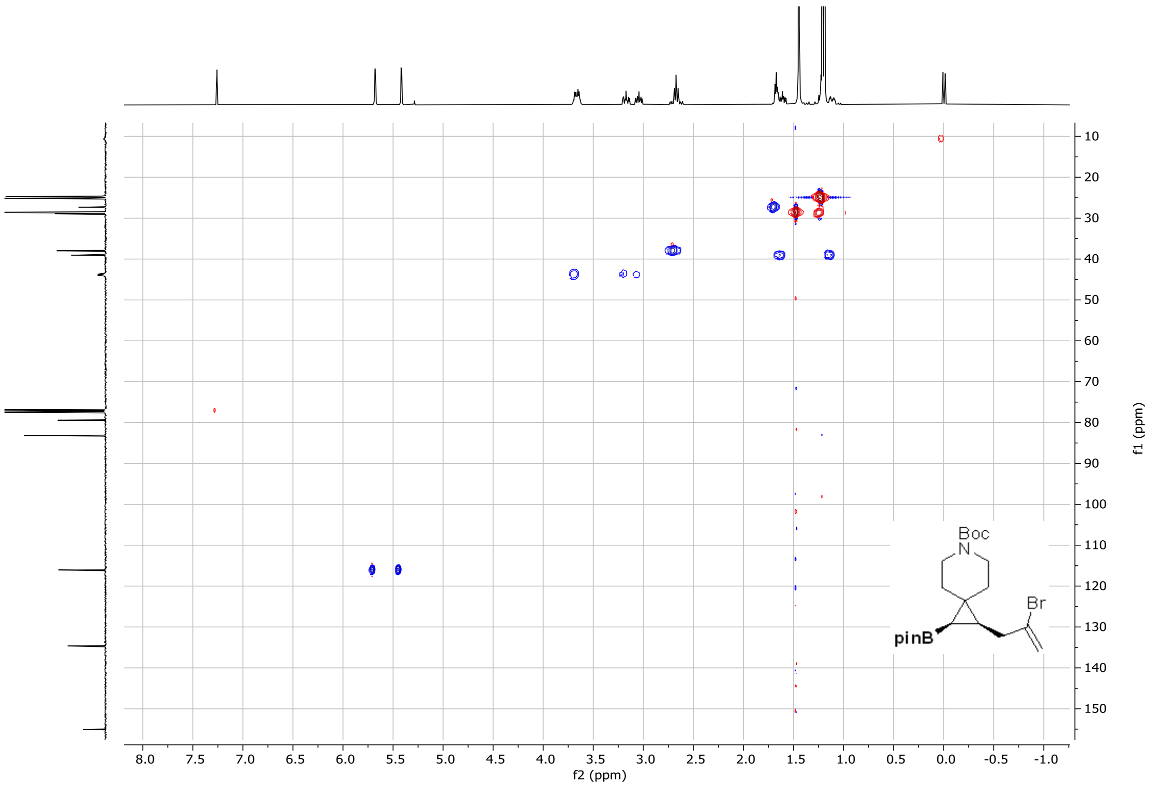

Compound 2i

^{1}H NMR (400 MHz, $CDCl_3$)

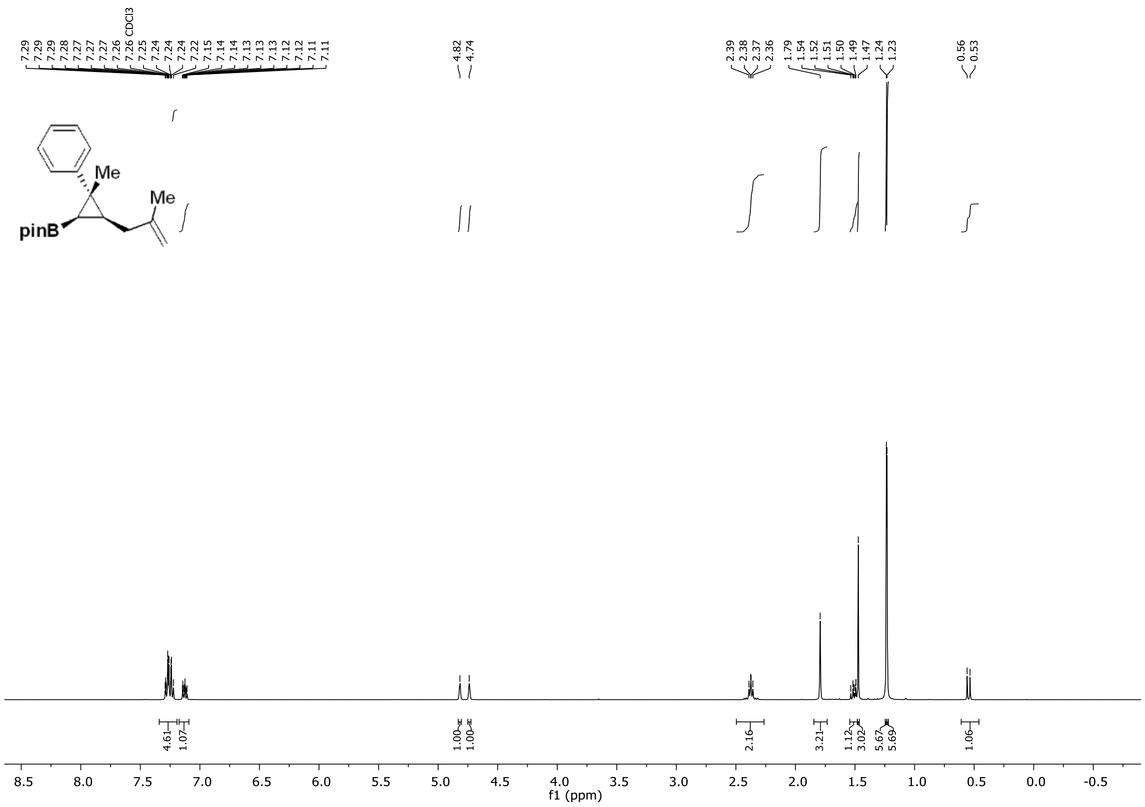

^{13}C NMR (101 MHz, $CDCl_3$)

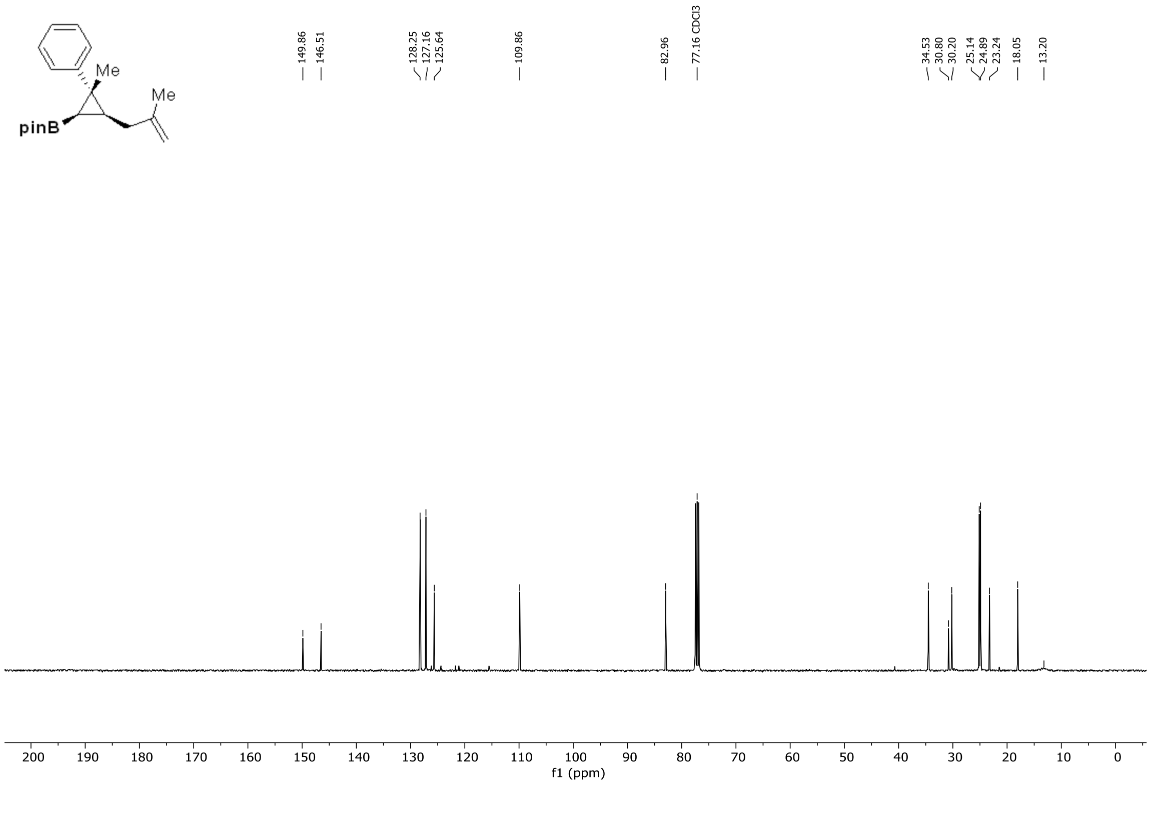

^{11}B NMR (128 MHz, $CDCl_3$)

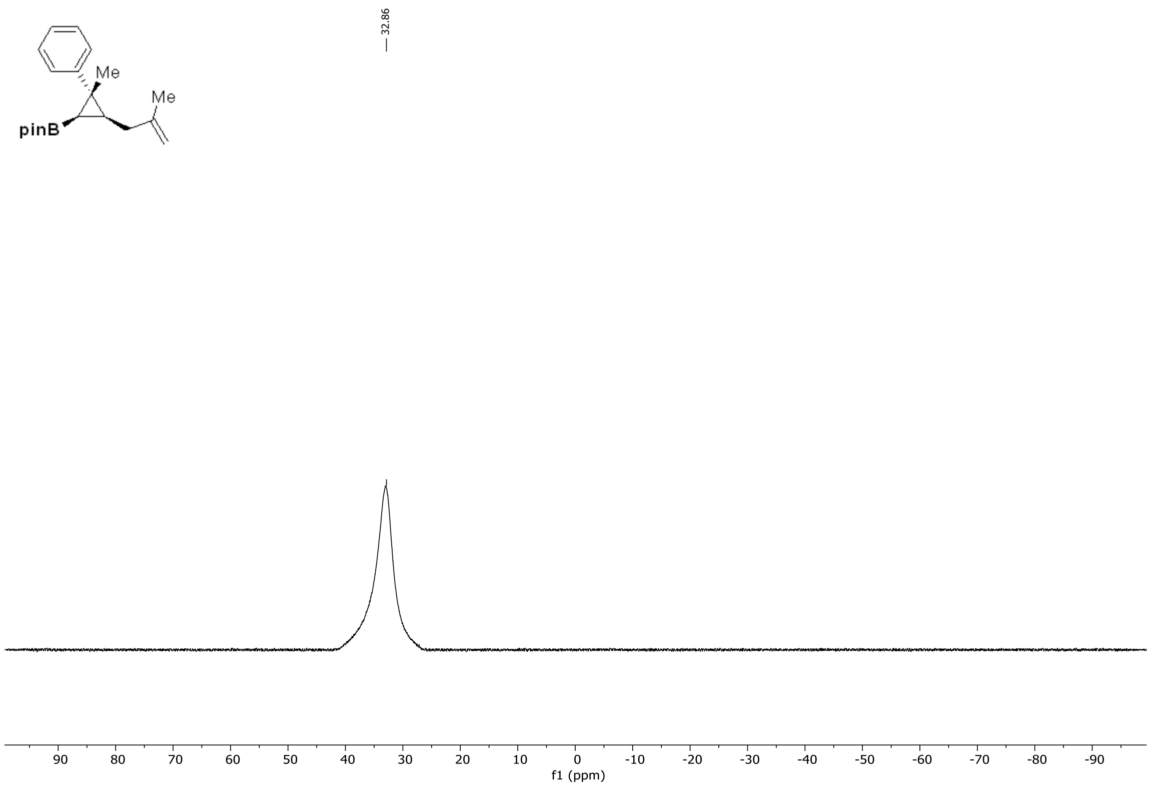

Compound 2j

^1H NMR (400 MHz, $CDCl_3$)

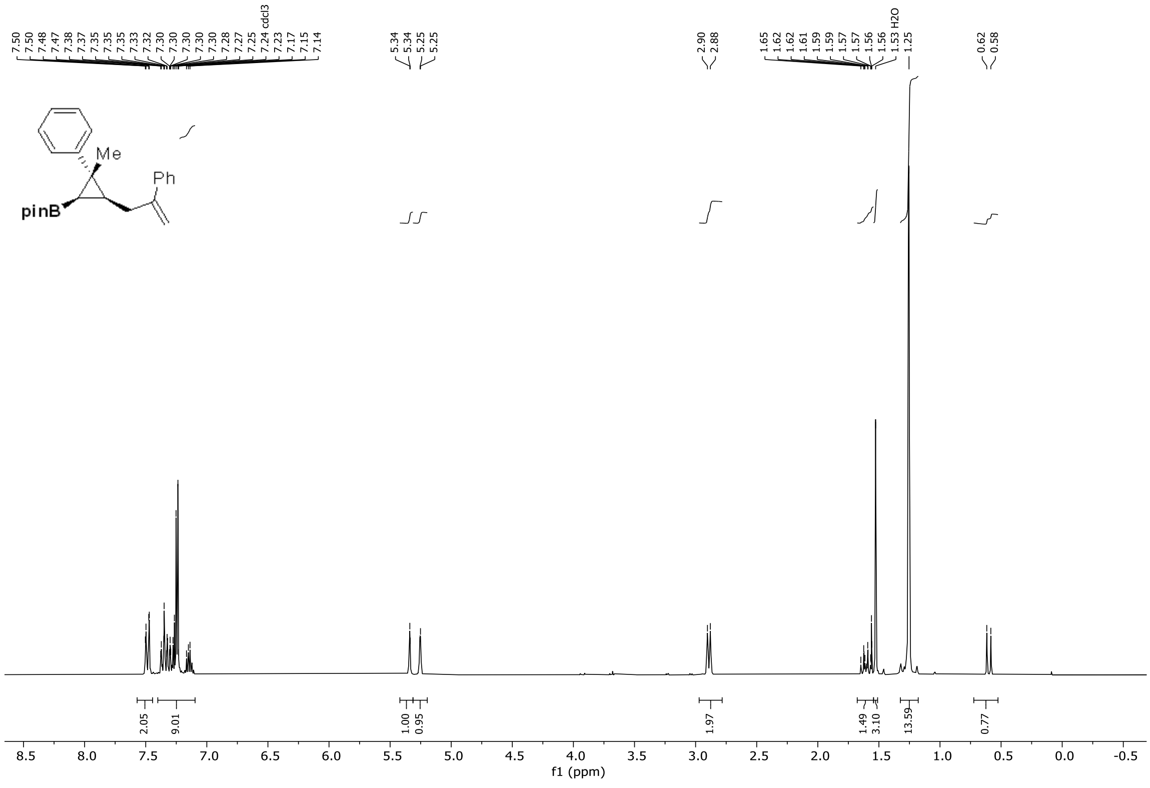

^{13}C NMR (101 MHz, $CDCl_3$)

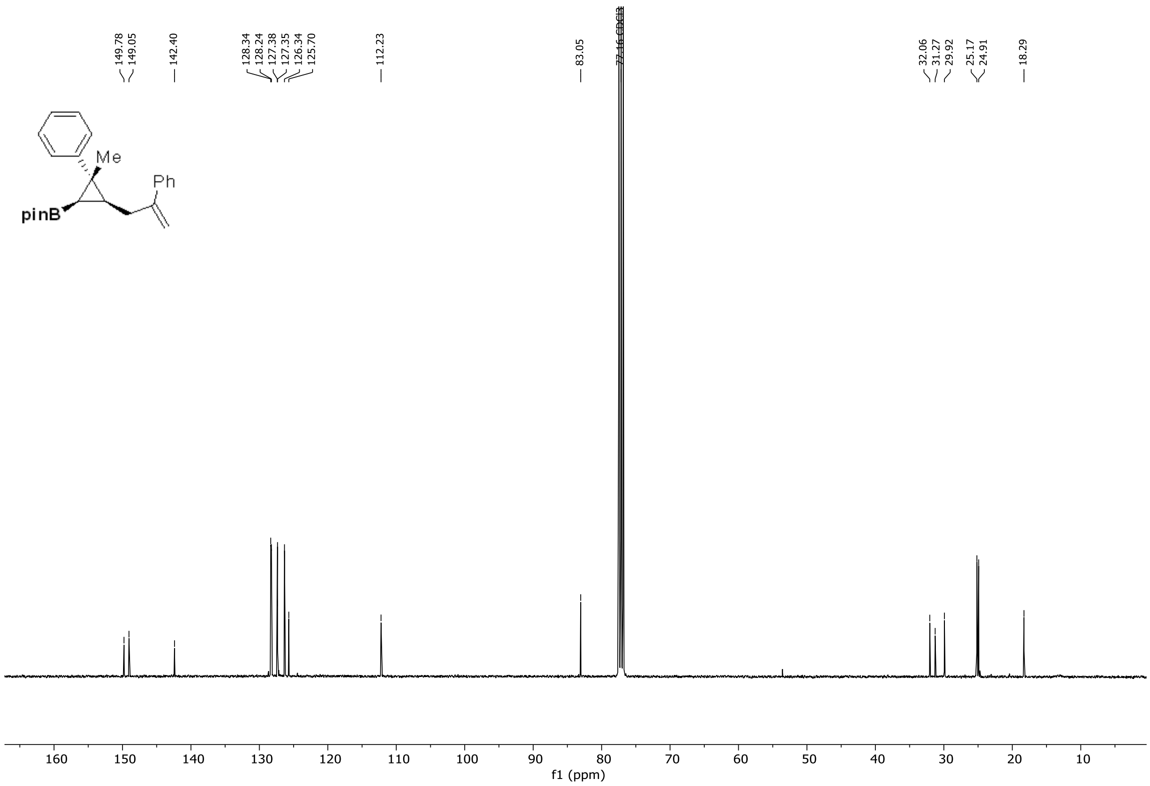

^{11}B NMR (128 MHz, $CDCl_3$)

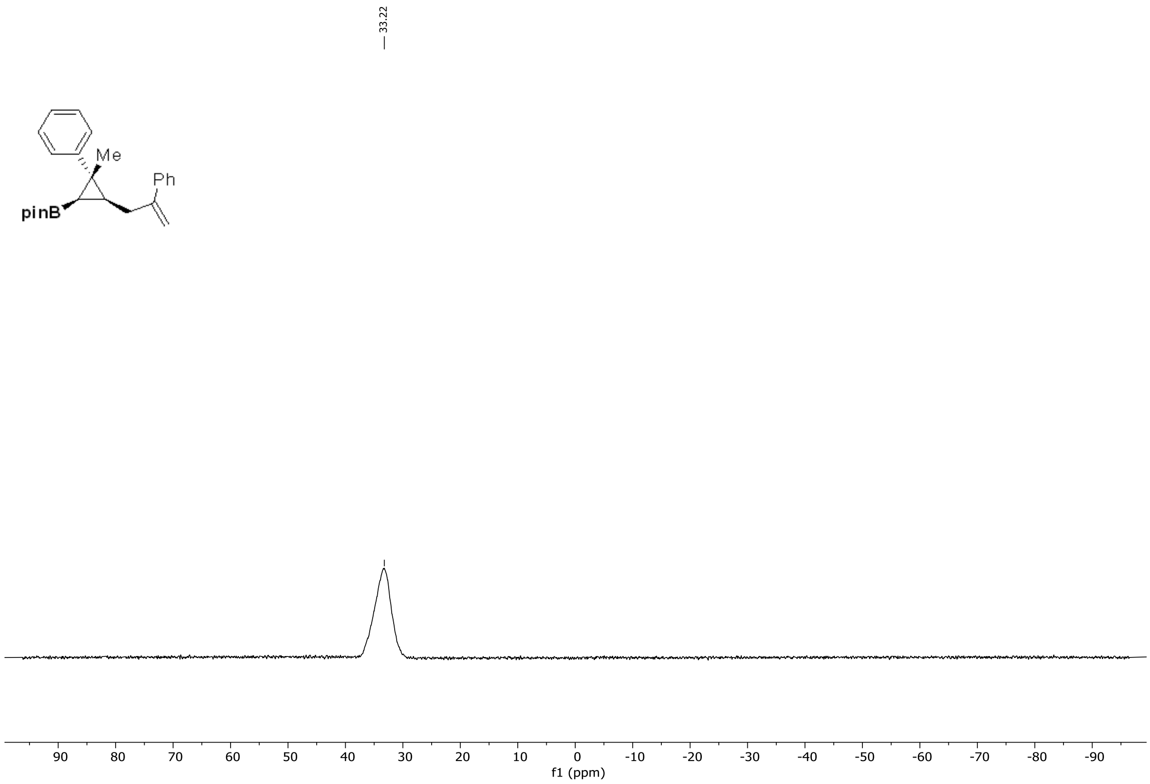

Compound 2k

^{1}H NMR (400 MHz, $CDCl_3$)

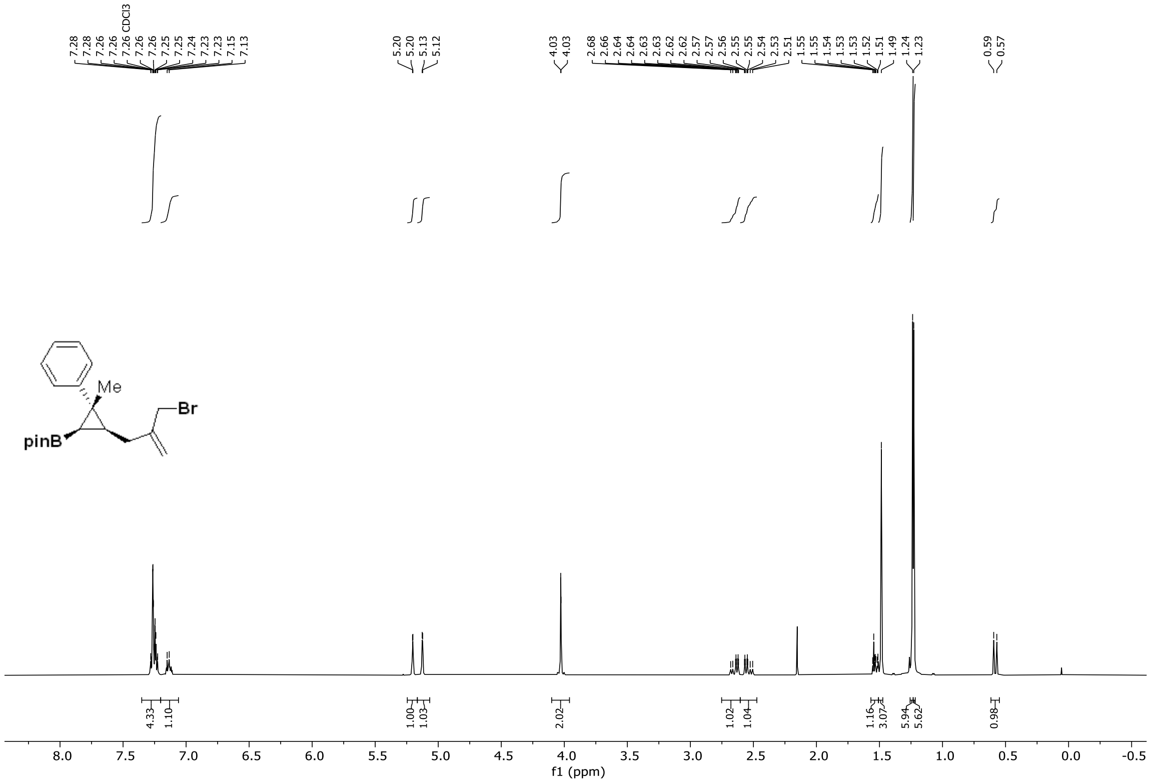

^{13}C NMR (101 MHz, $CDCl_3$)

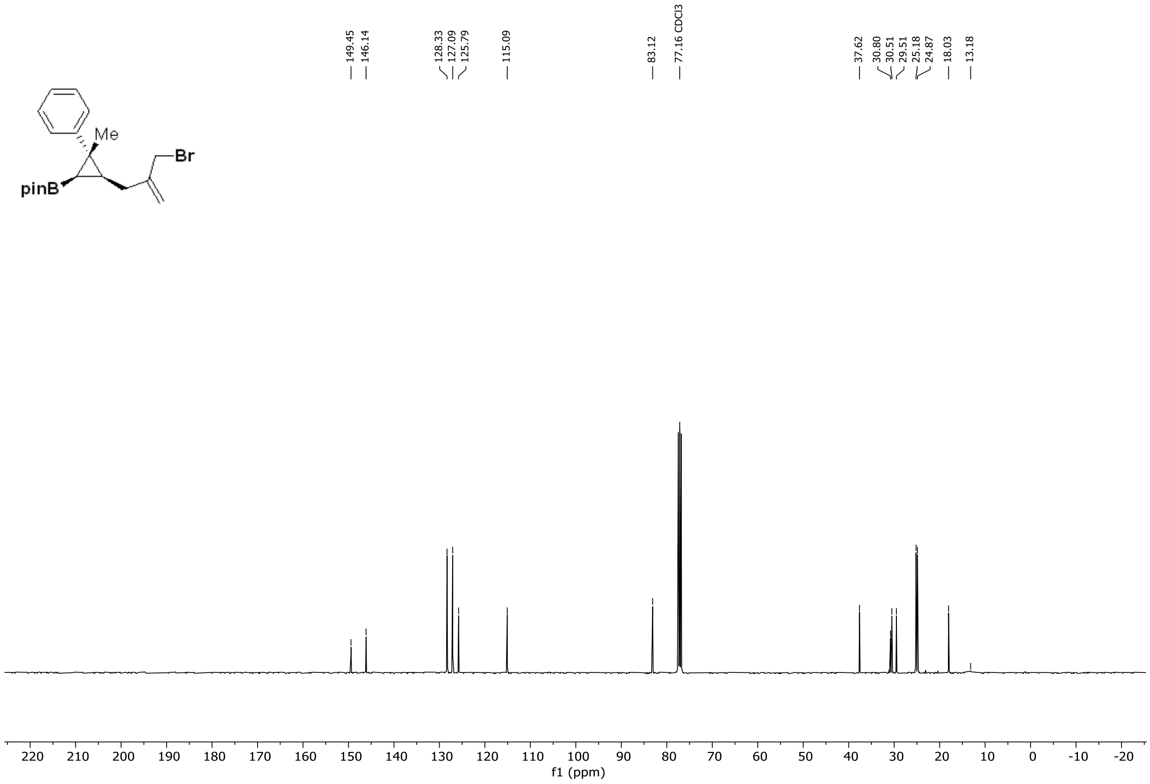

^{11}B NMR (128 MHz, $CDCl_3$)

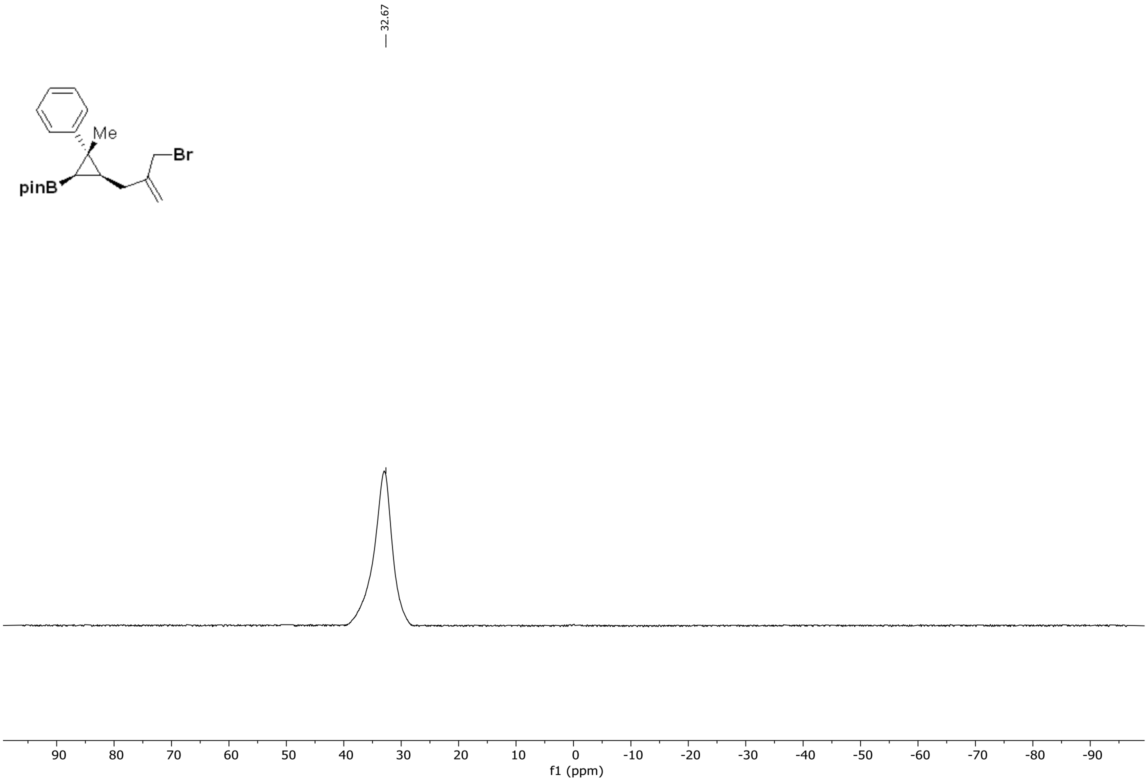

2D-NOESY (400 MHz, $CDCl_3$)

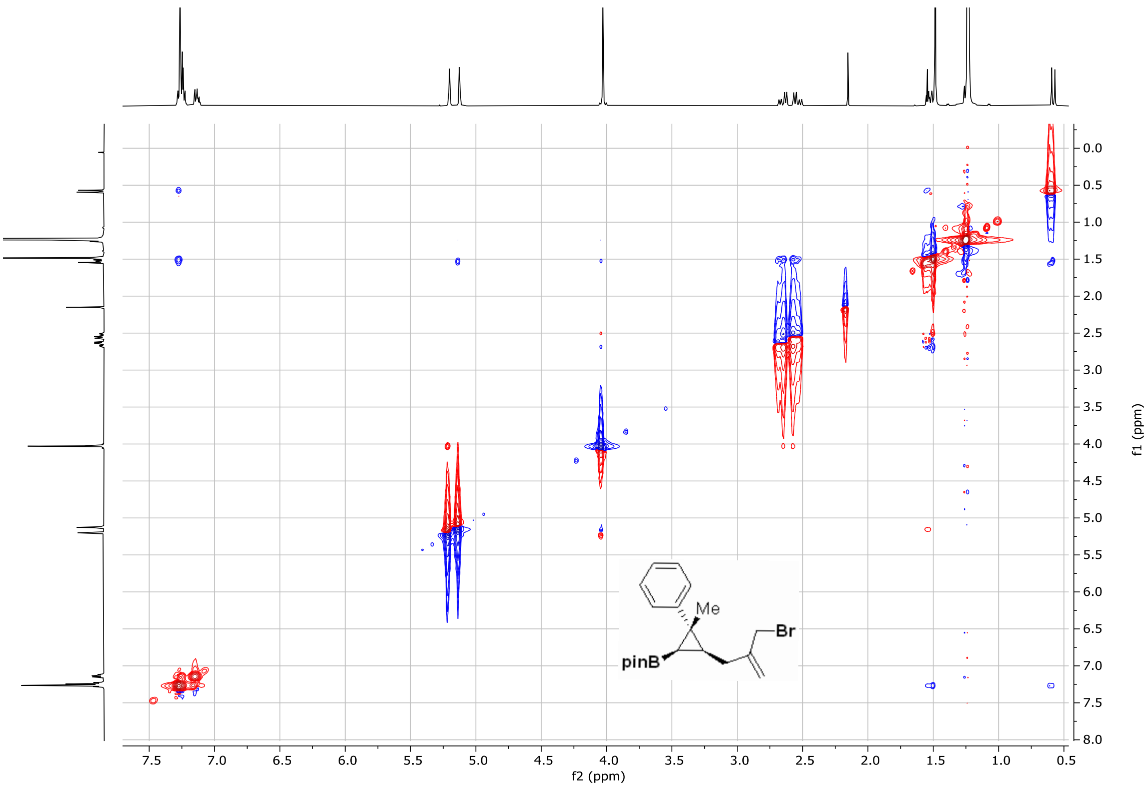

Compound 2k’

^{1}H NMR (400 MHz, $CDCl_3$)

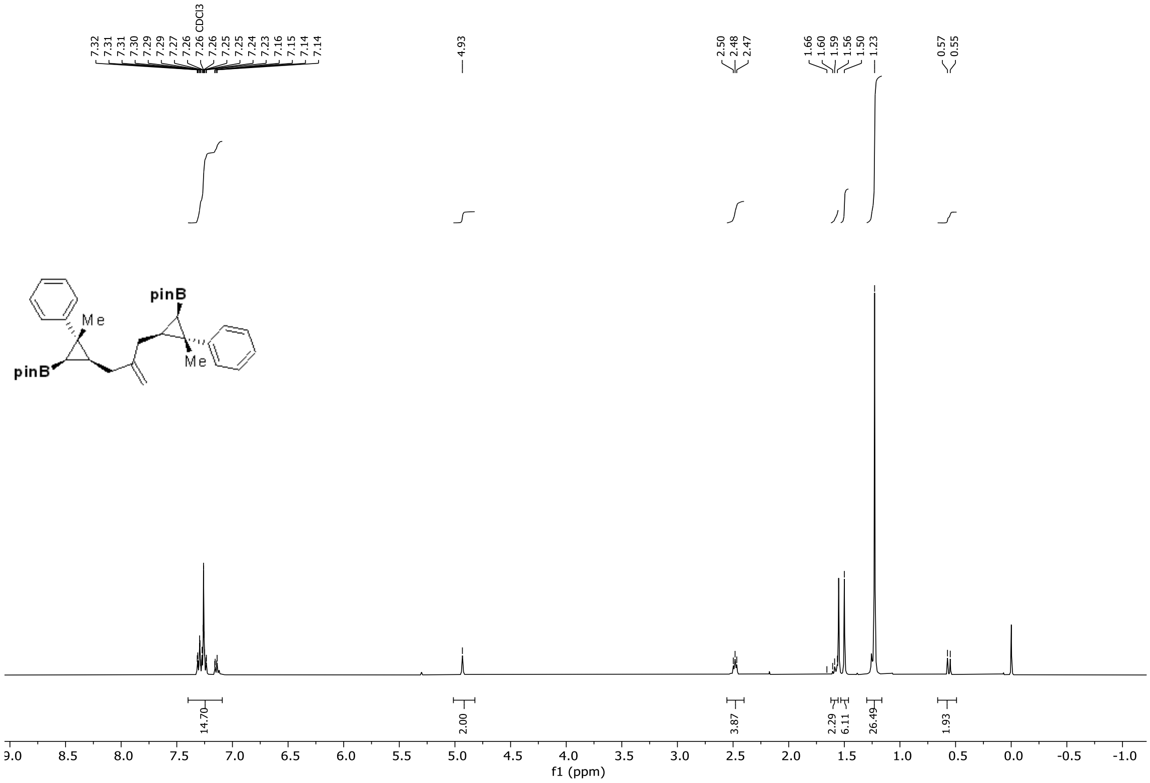

^{13}C NMR (101 MHz, $CDCl_3$)

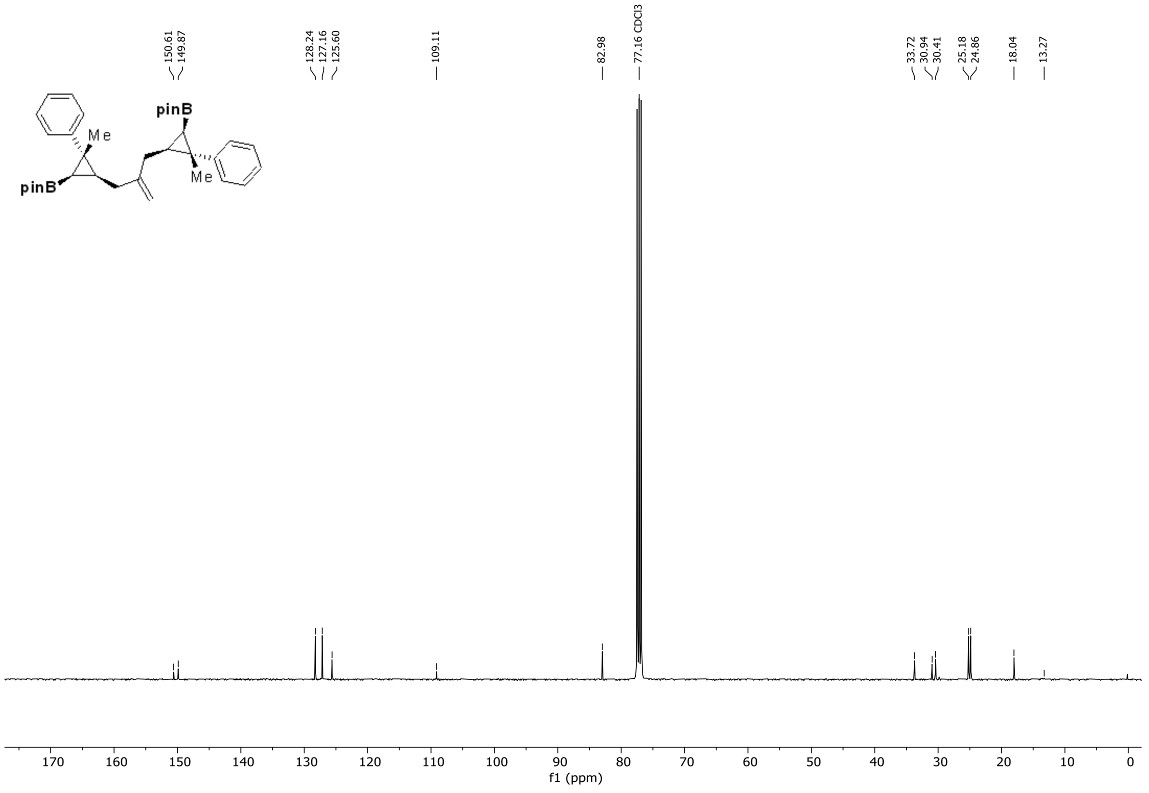

^{11}B NMR (128 MHz, $CDCl_3$)

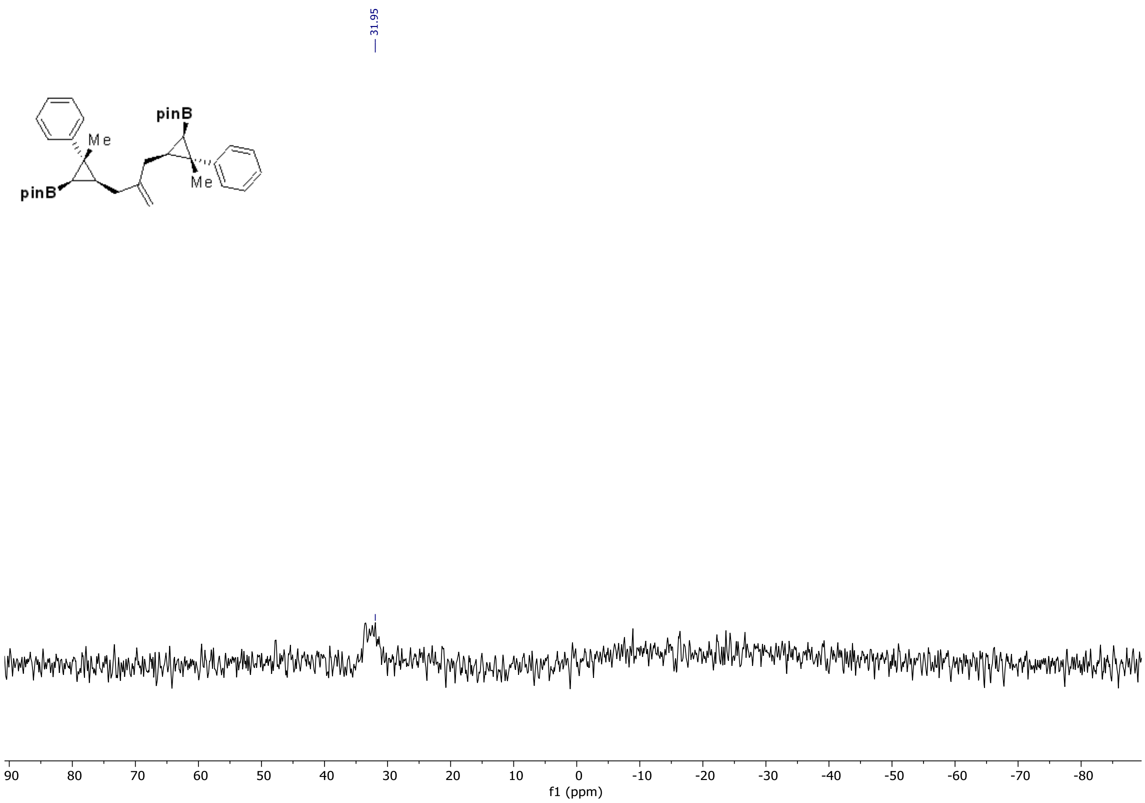

Compound 2l

^{1}H NMR (400 MHz, $CDCl_3$)

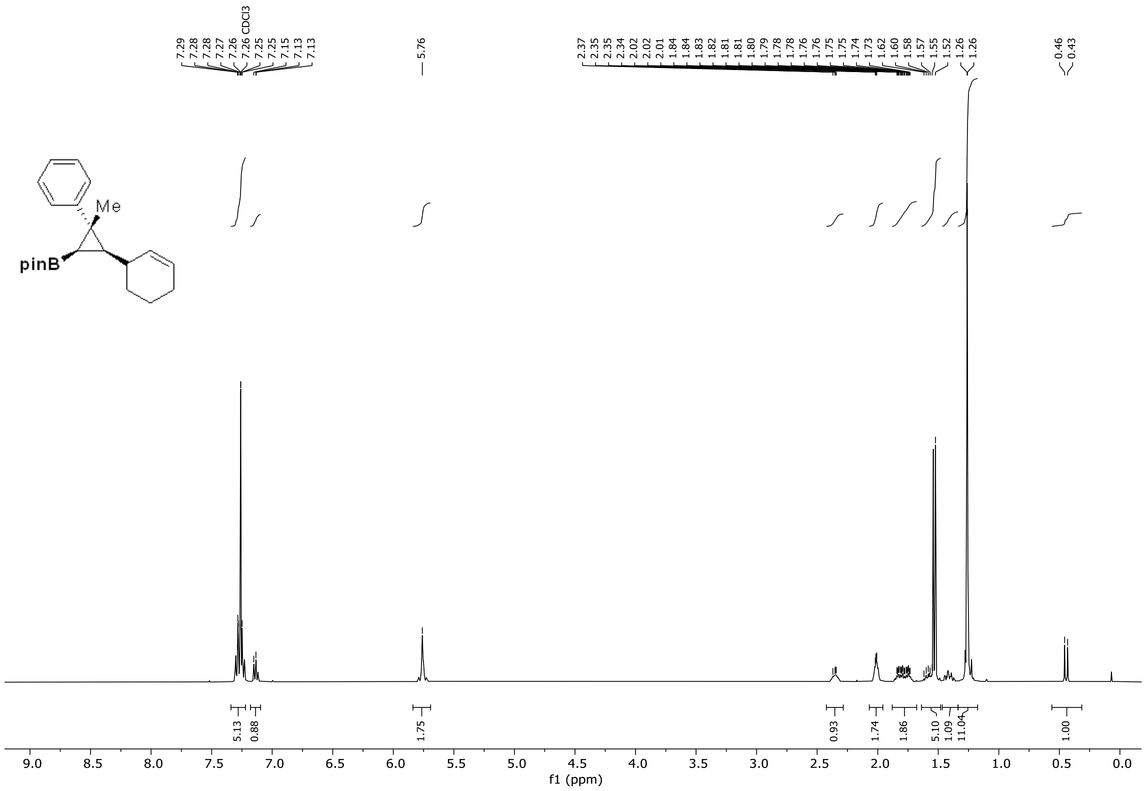

^{13}C NMR (101 MHz, $CDCl_3$)

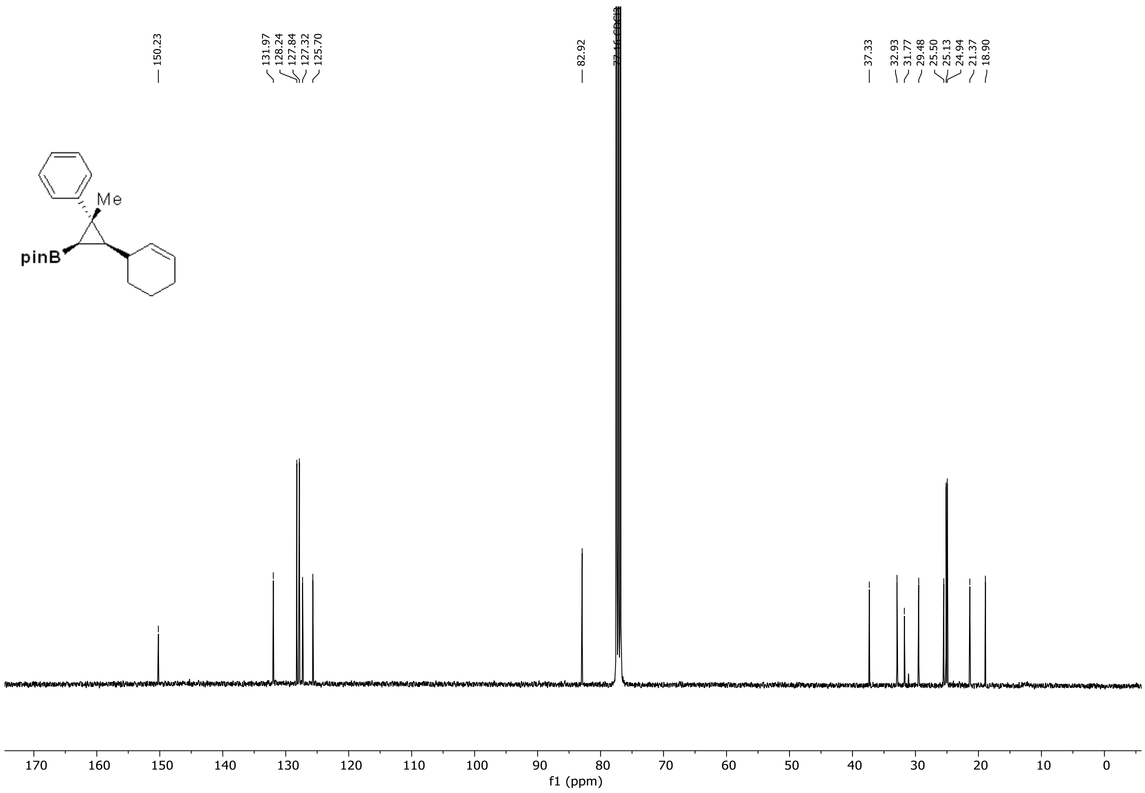

^{11}B NMR (128 MHz, $CDCl_3$)

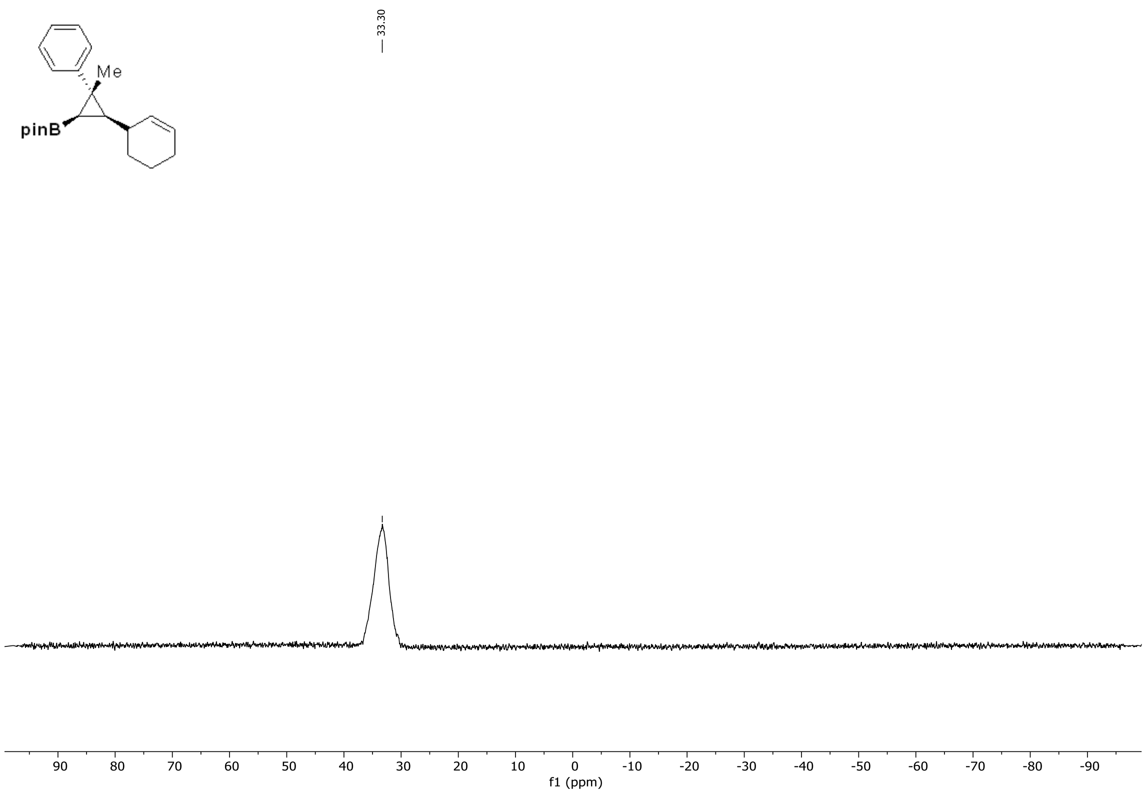

2D-NOESY (400 MHz, $CDCl_3$)

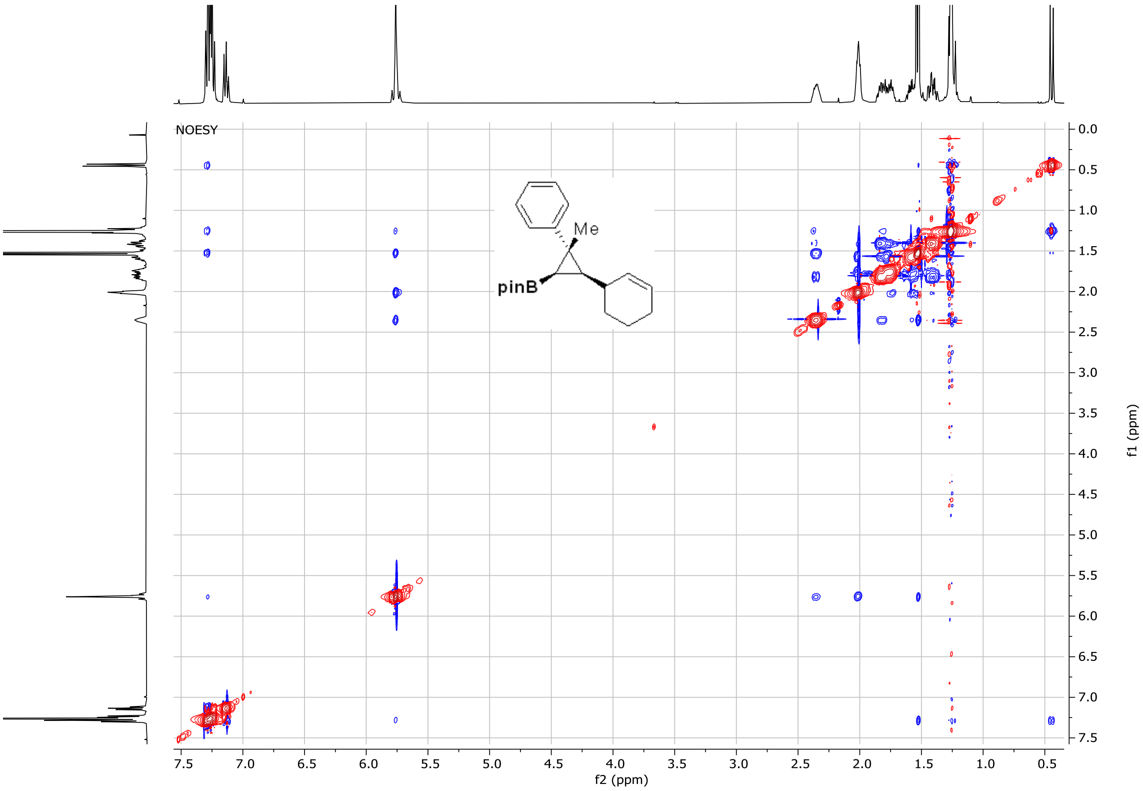

Compound 2m

^1H NMR (400 MHz, $CDCl_3$)

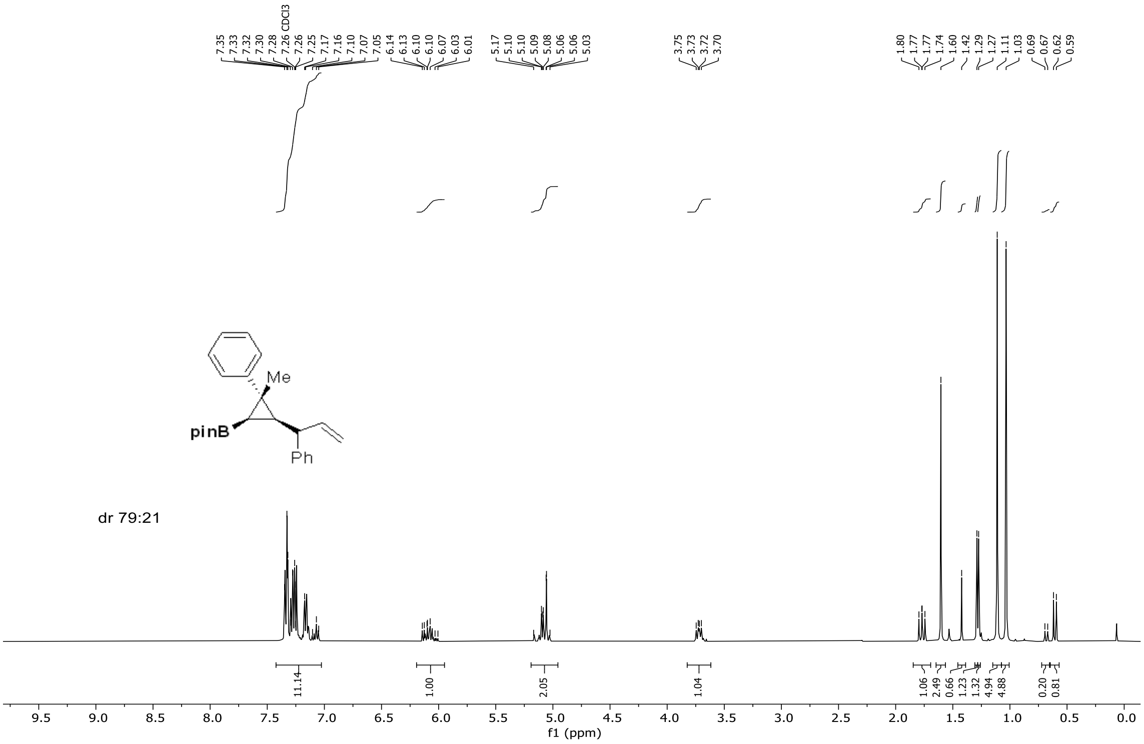

^{13}C NMR (101 MHz, $CDCl_3$)

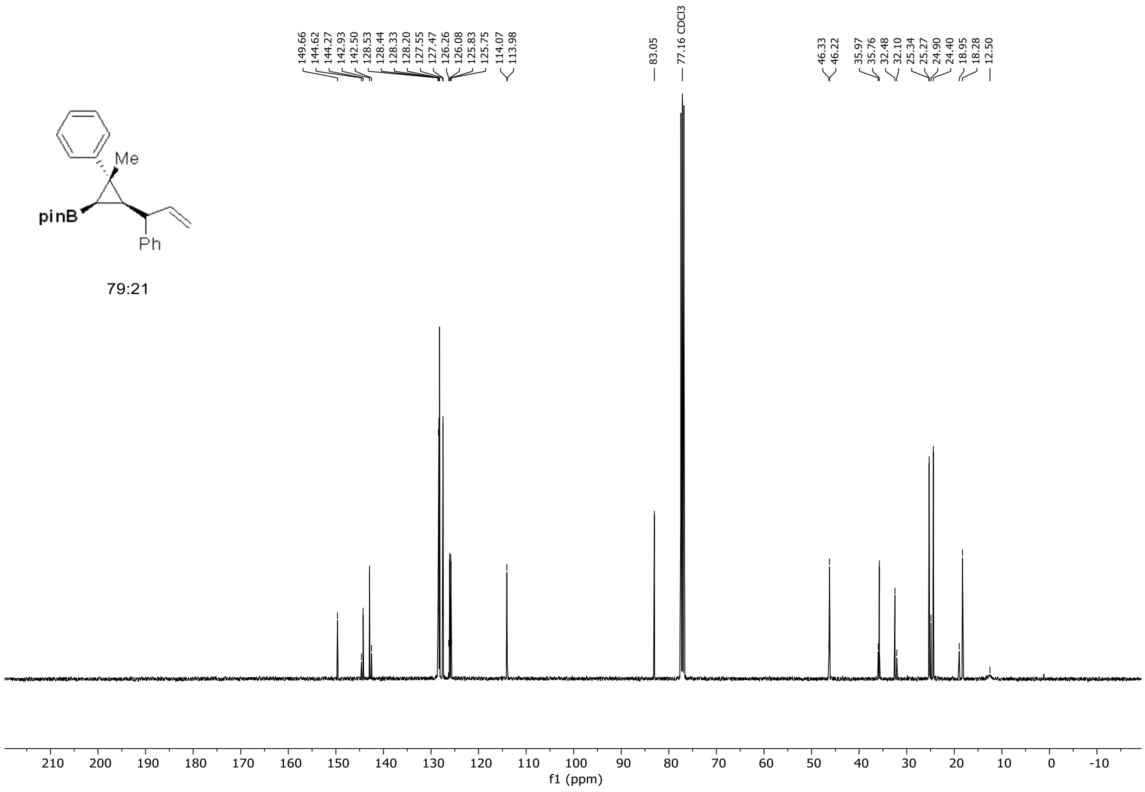

^{11}B NMR (128 MHz, $CDCl_3$)

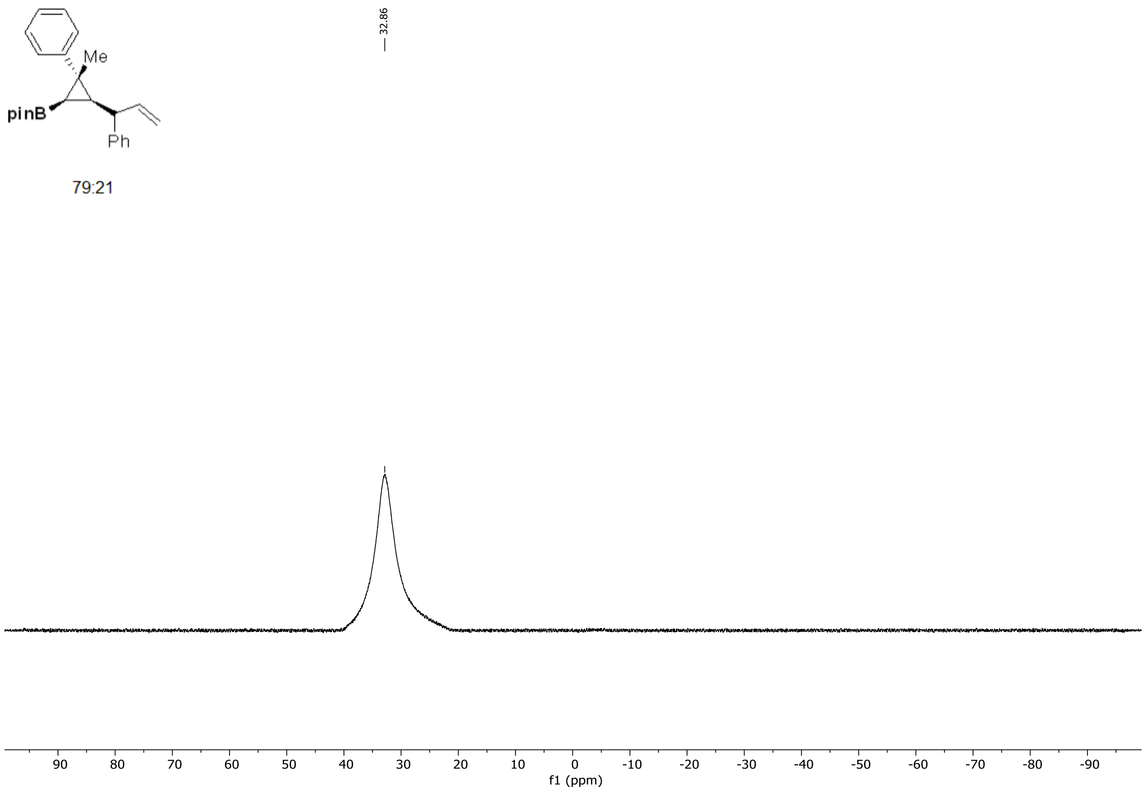

Compound 2n

^{1}H NMR (300 MHz, $CDCl_3$)

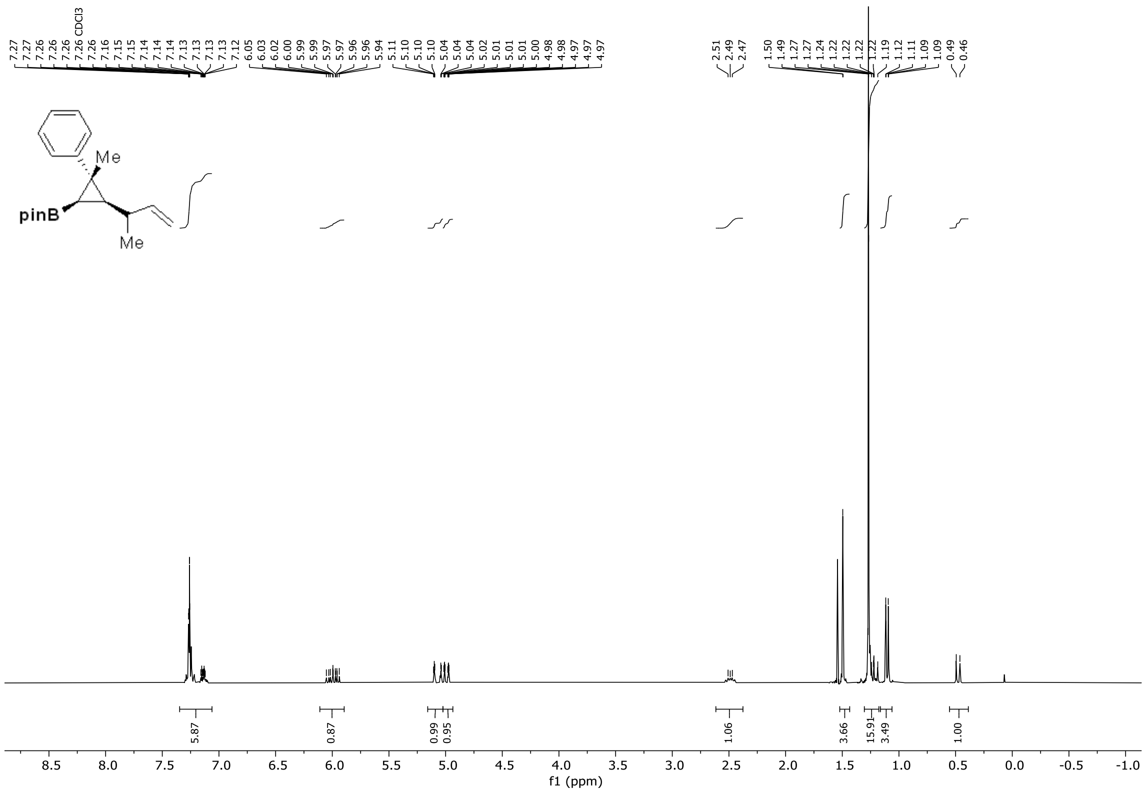

^{13}C NMR (101 MHz, $CDCl_3$)

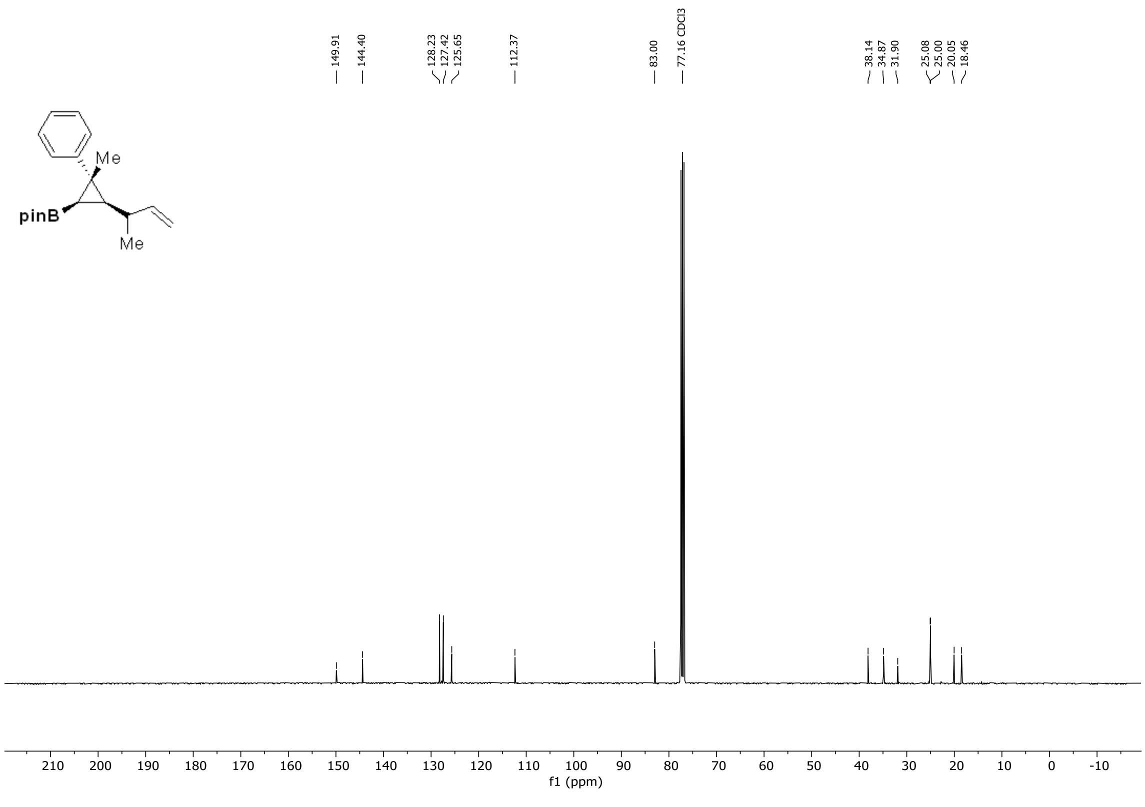

^{11}B NMR (128 MHz, $CDCl_3$)

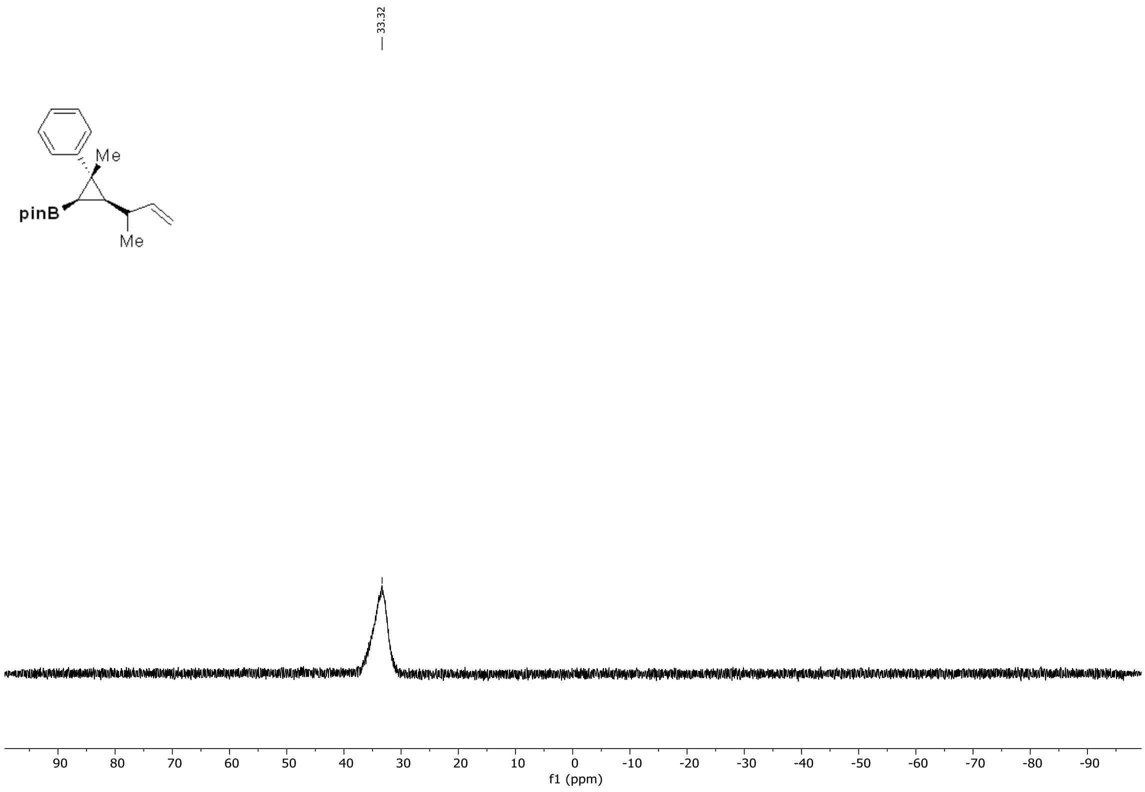

Compound 2o

^{1}H NMR (500 MHz, $CDCl_3$)

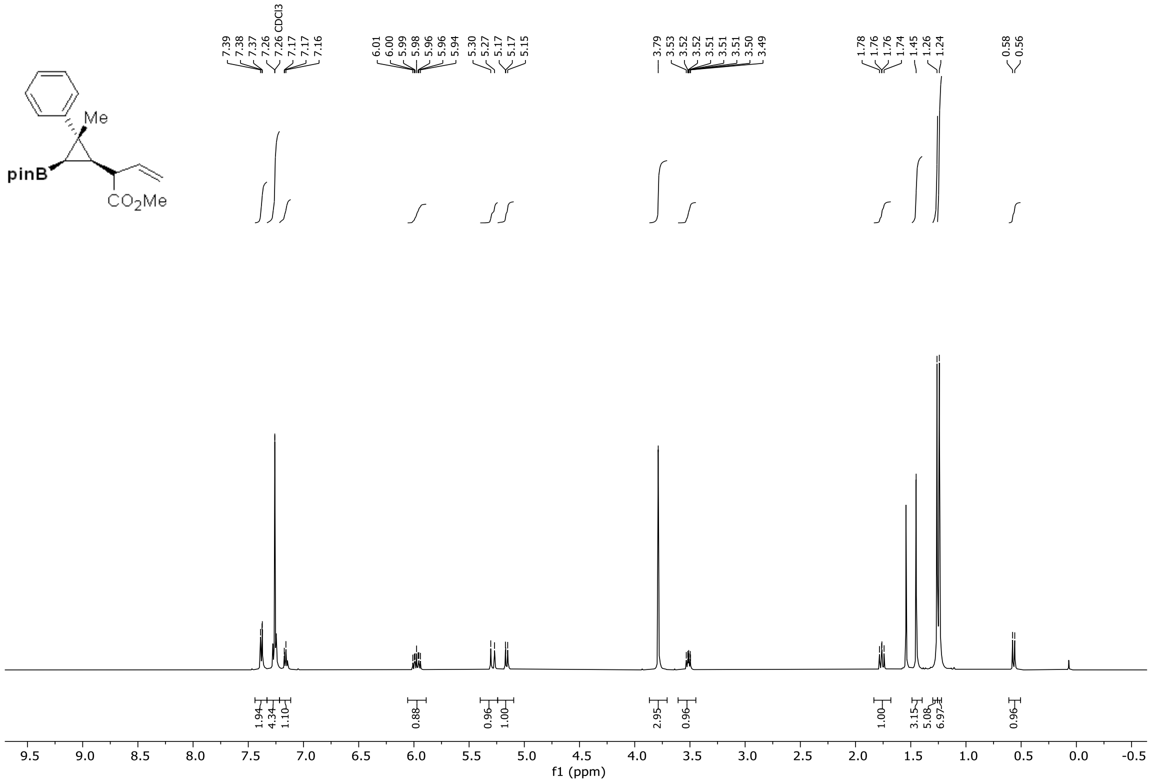

^{13}C NMR (126 MHz, $CDCl_3$)

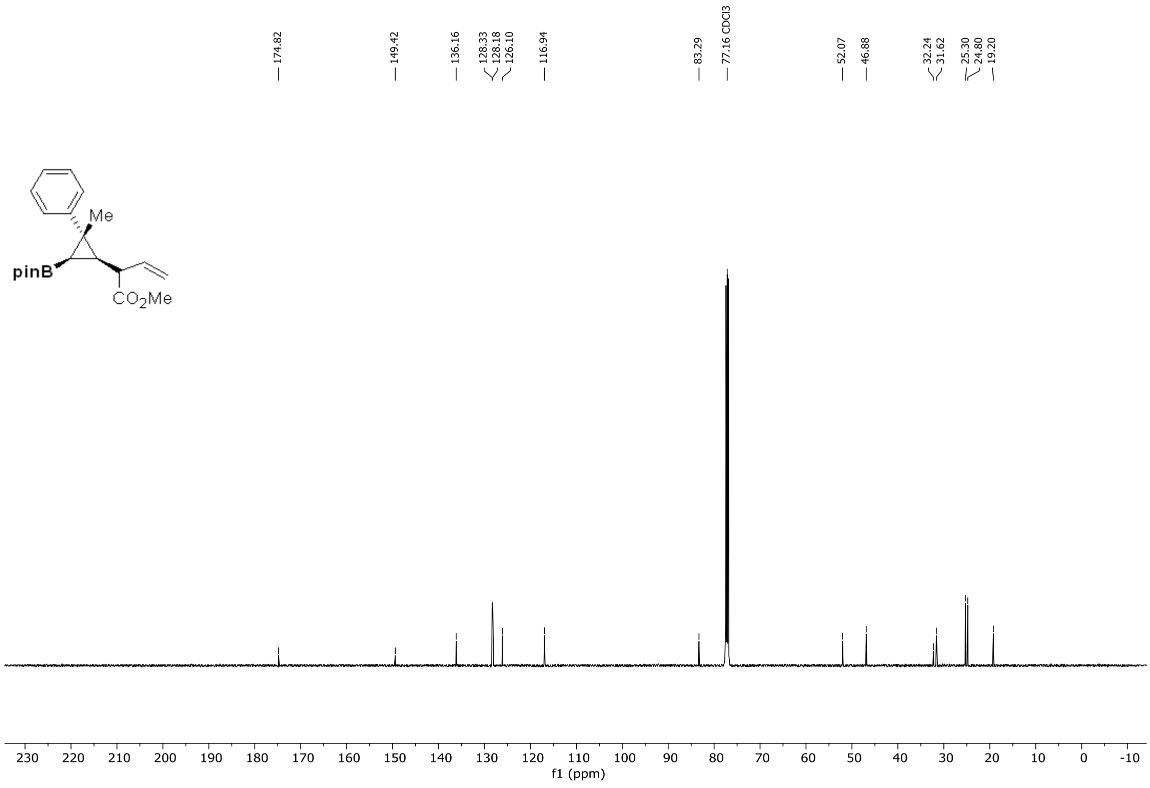

^{11}B NMR (128 MHz, $CDCl_3$)

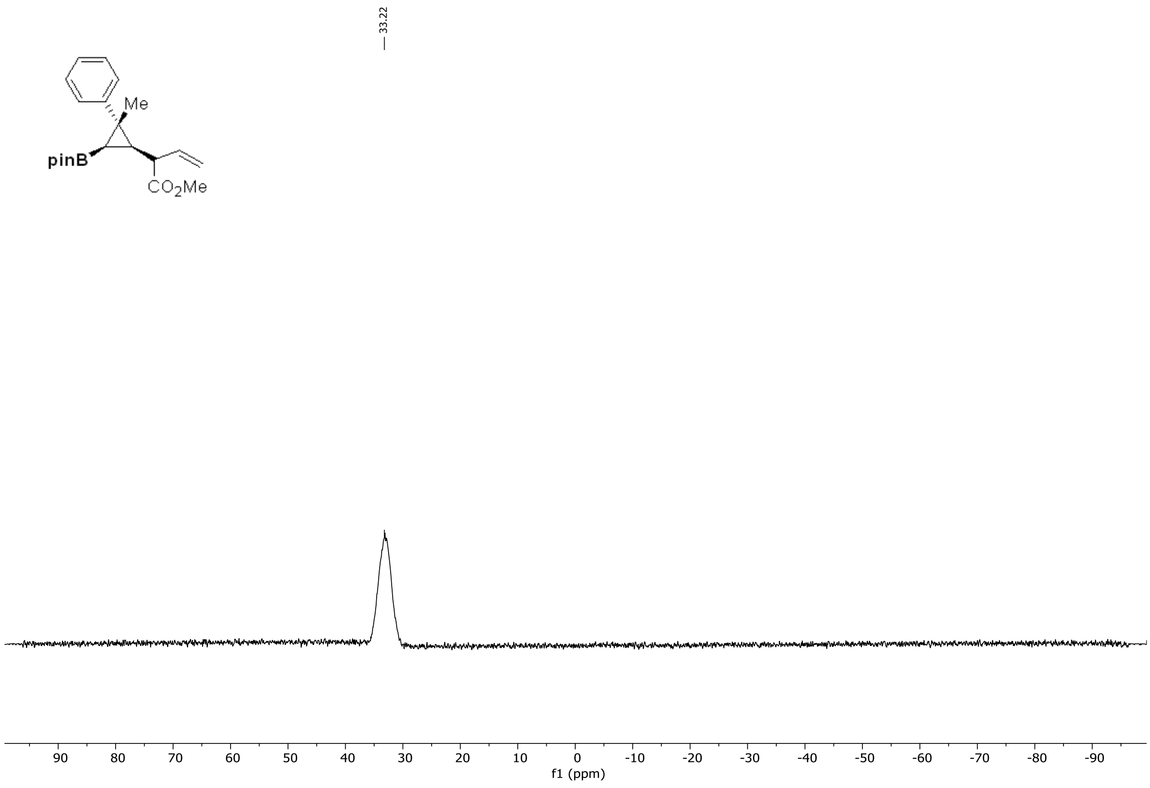

2D-NOESY (400 MHz, $CDCl_3$)

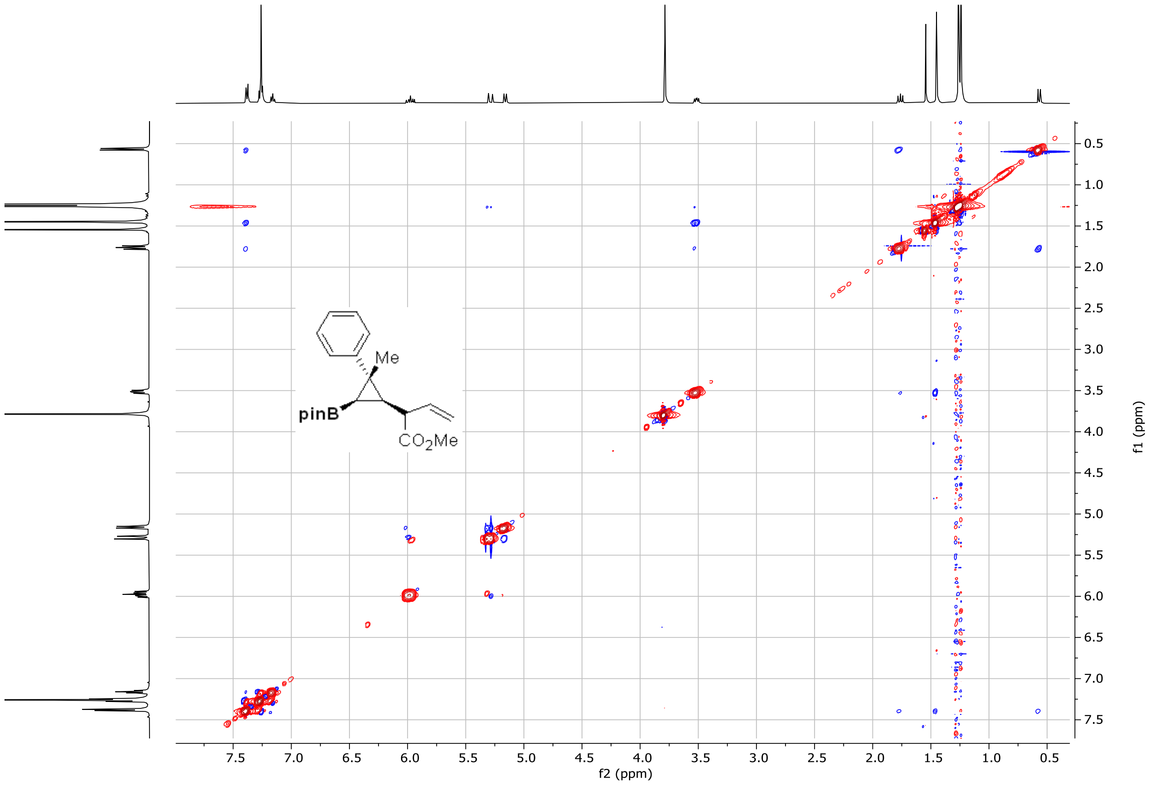

Propargylation/alkynylation of cyclopropyl bisboronates

Compound 3a

^{1}H NMR (400 MHz, $CDCl_3$)

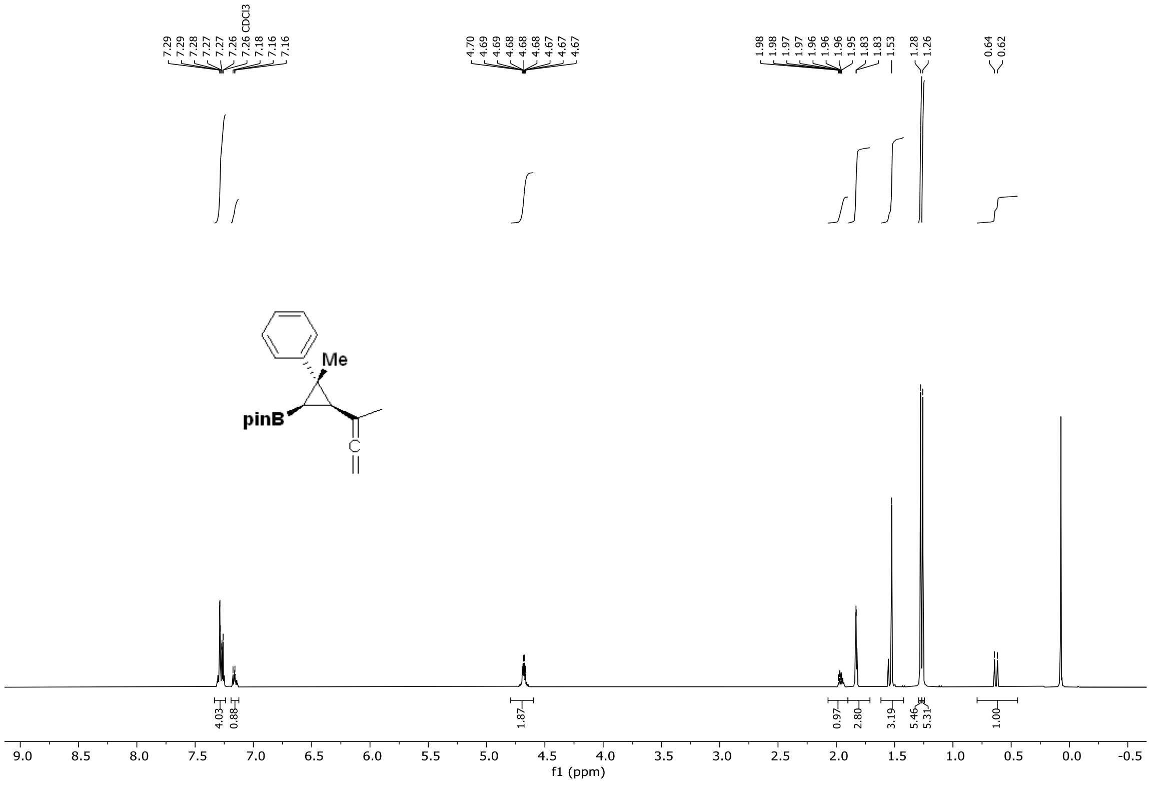

^{13}C NMR (101 MHz, $CDCl_3$)

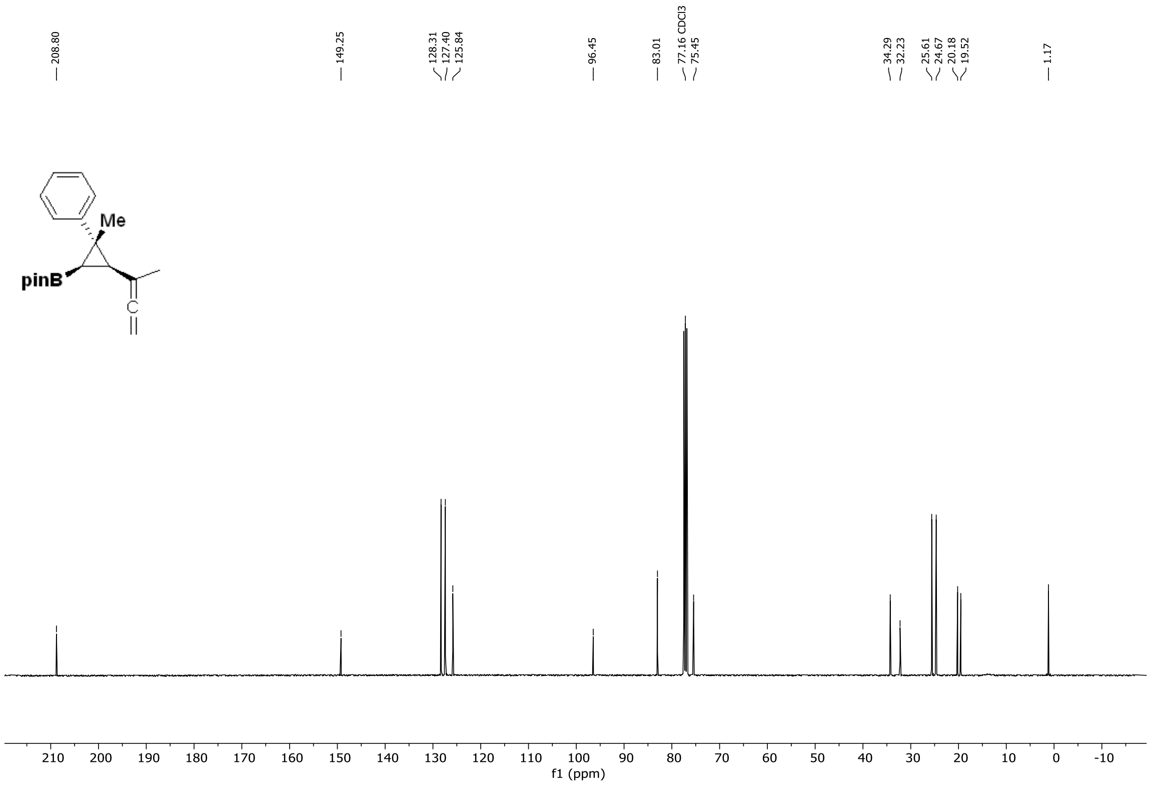

^{11}B NMR (128 MHz, $CDCl_3$)

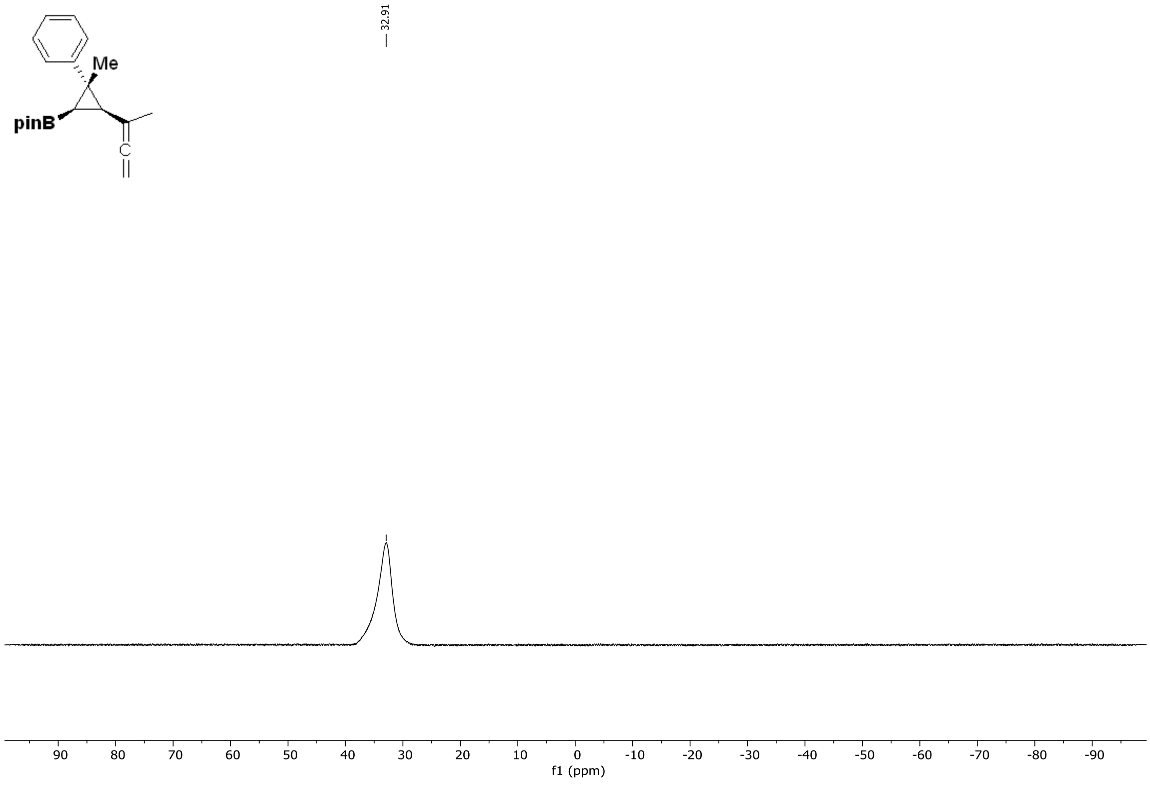

2D-NOESY (400 MHz, $CDCl_3$)

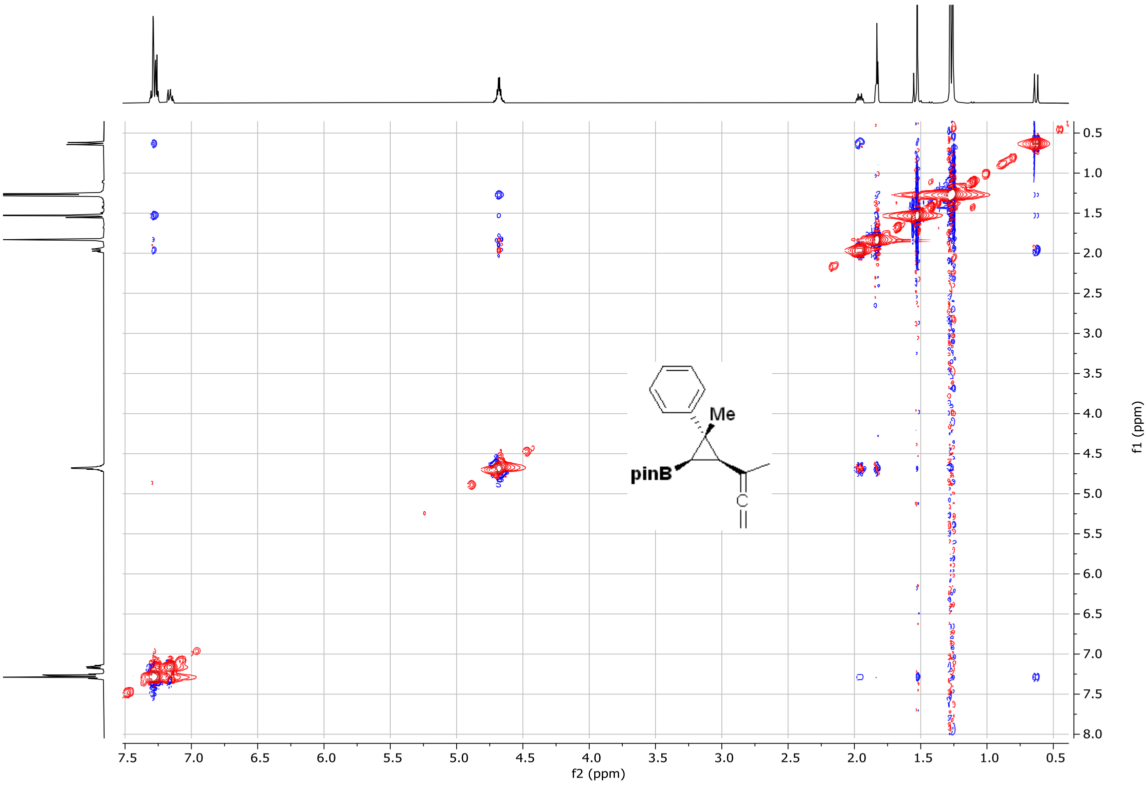

Compound 3b

^1H NMR (400 MHz, $CDCl_3$)

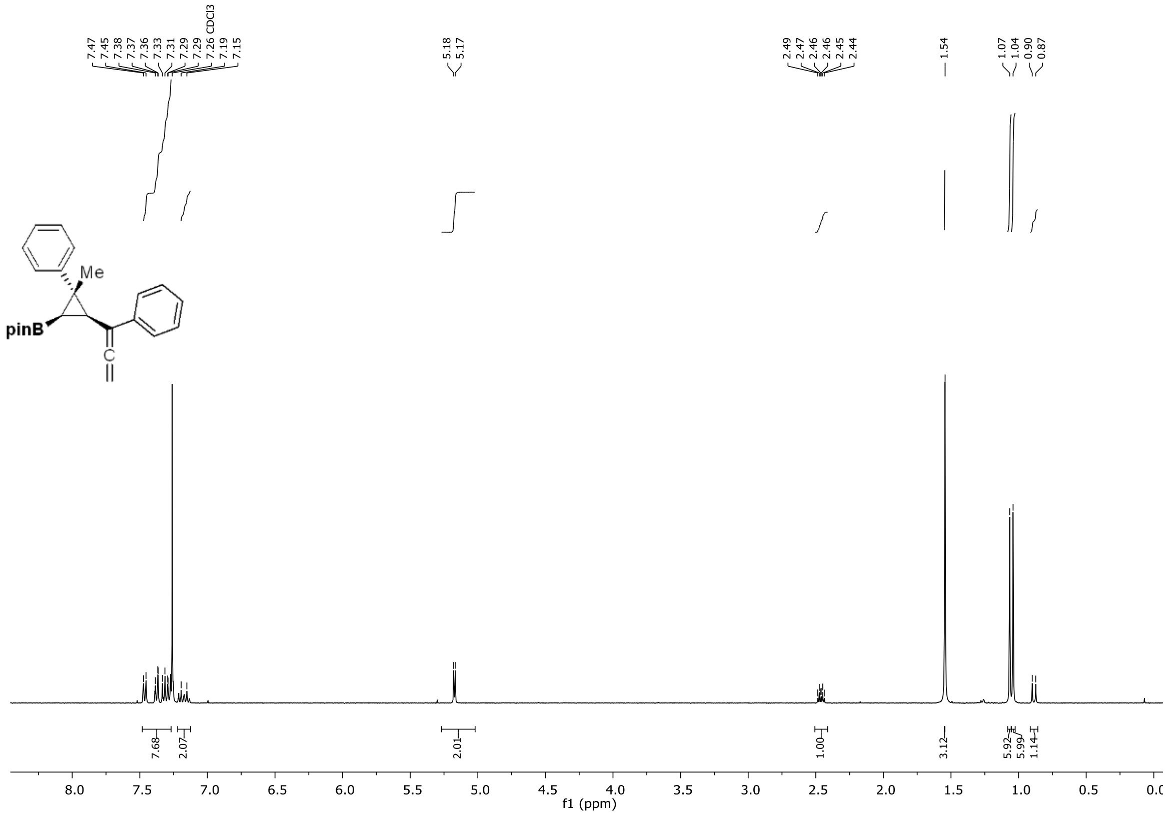

^{13}C NMR (101 MHz, $CDCl_3$)

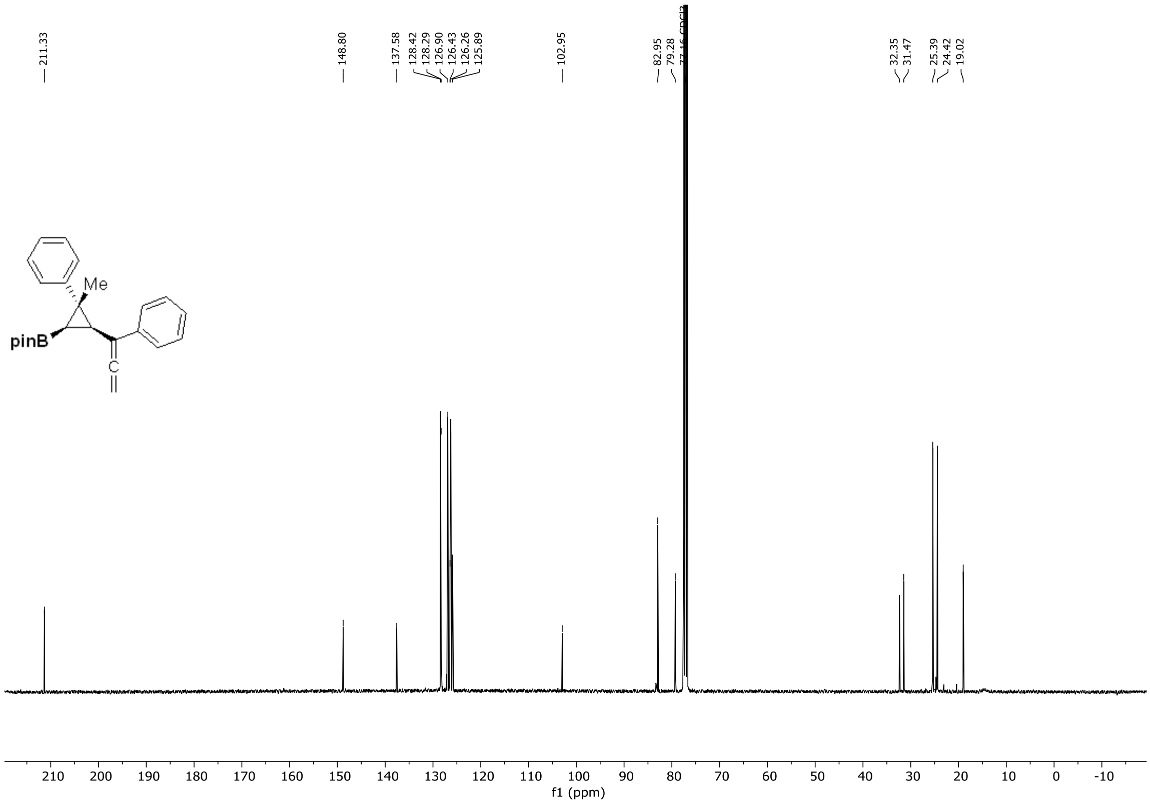

^{11}B NMR (128 MHz, $CDCl_3$)

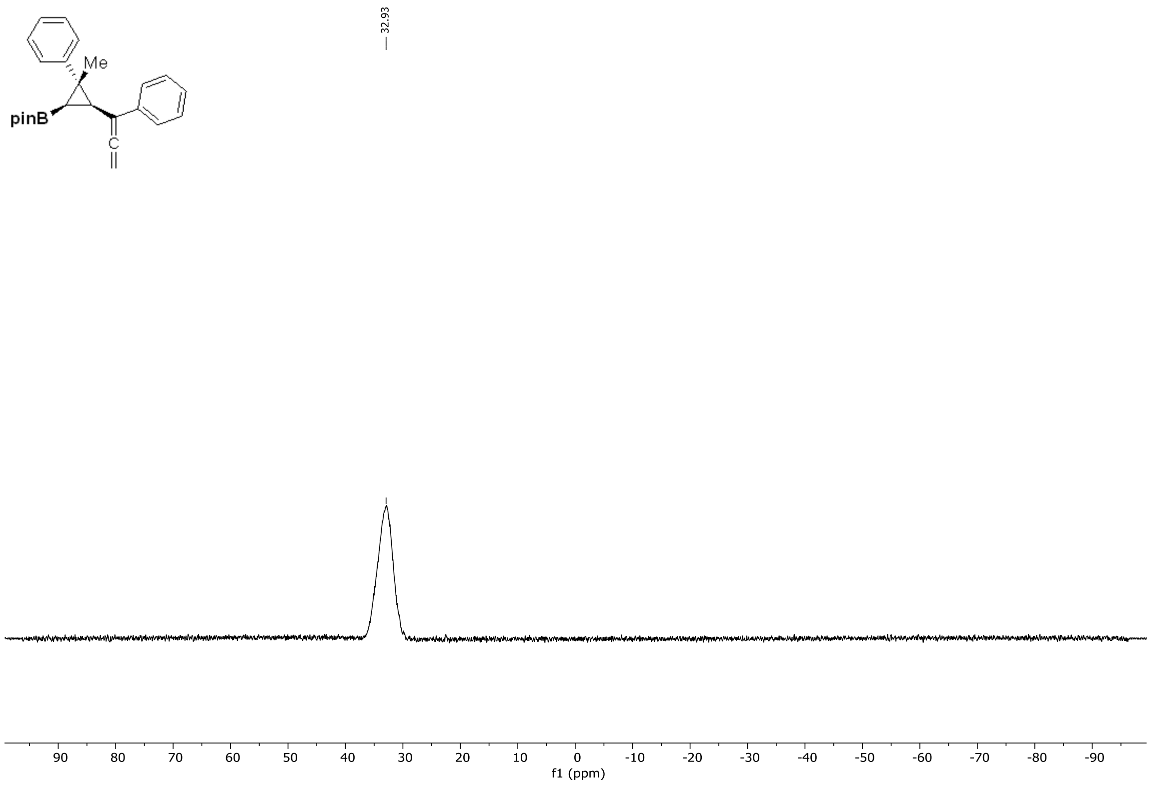

Compound 3c

^1H NMR (400 MHz, $CDCl_3$)

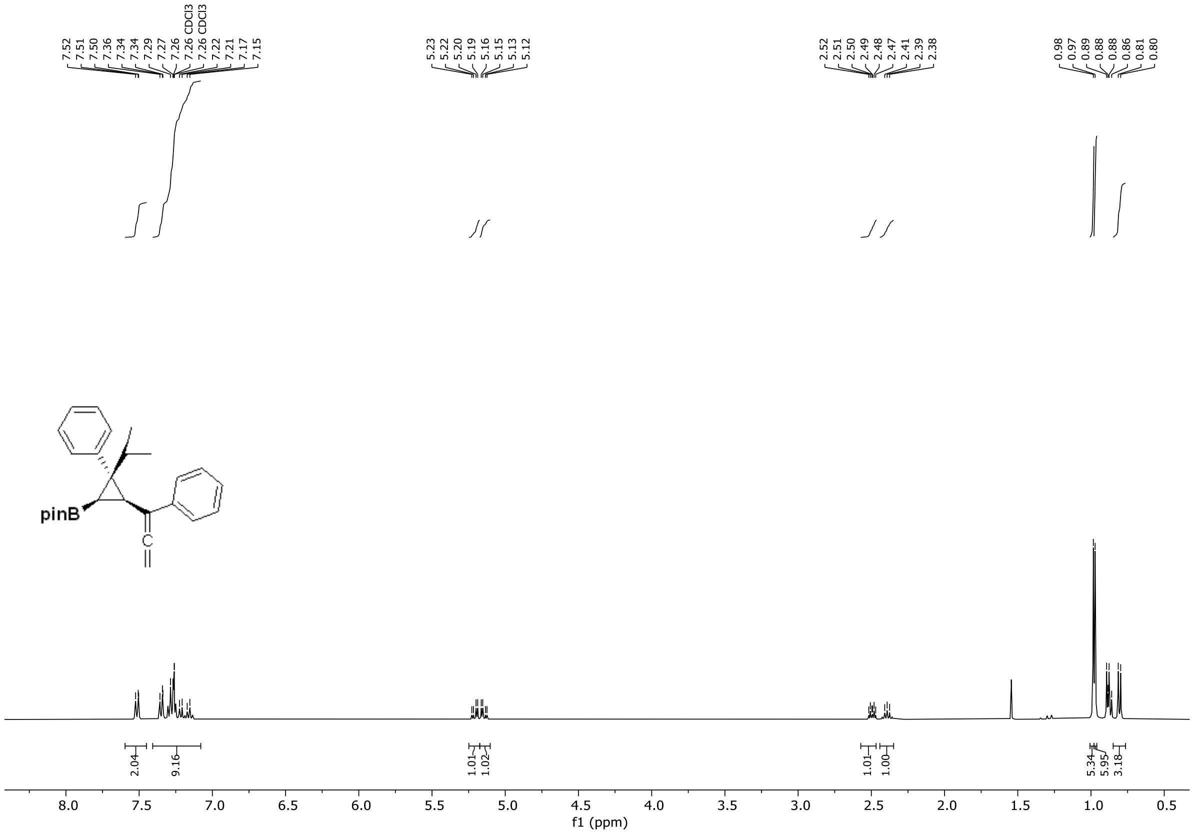

^{13}C NMR (101 MHz, $CDCl_3$)

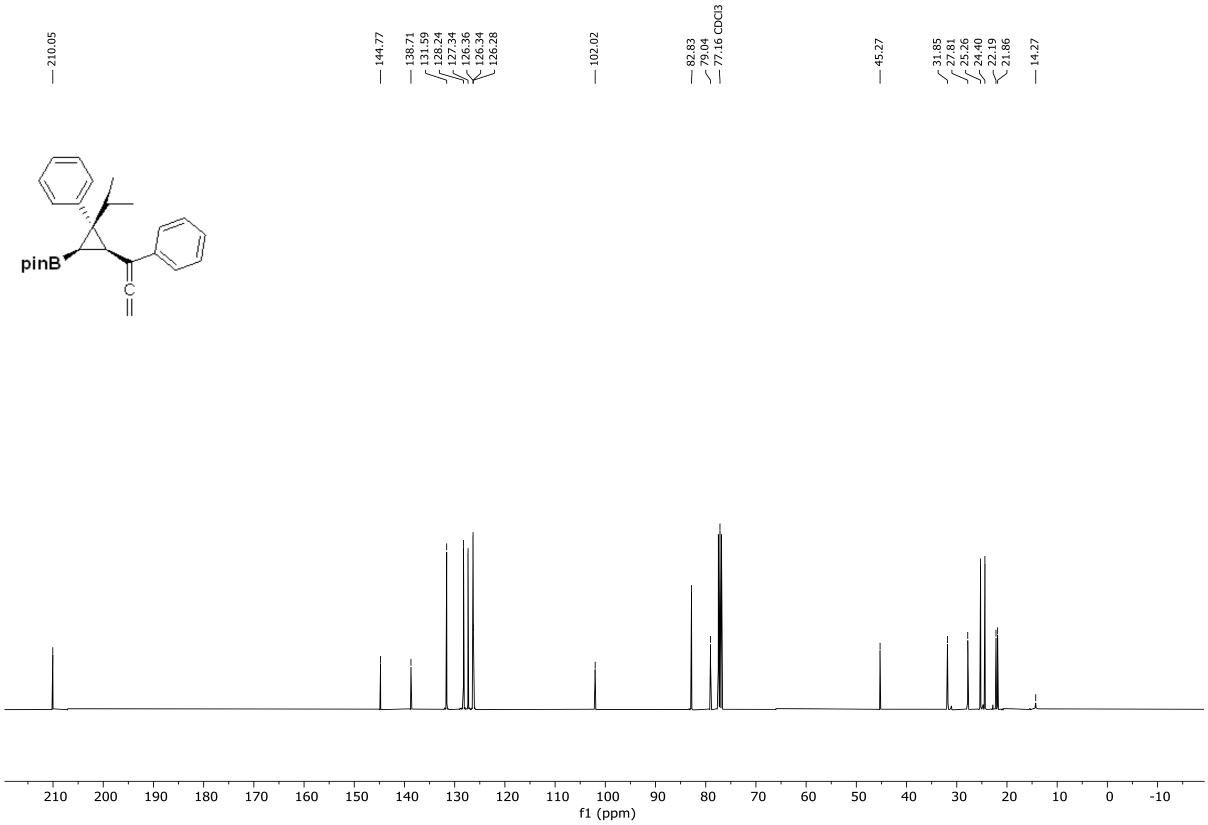

^{11}B NMR (128 MHz, $CDCl_3$)

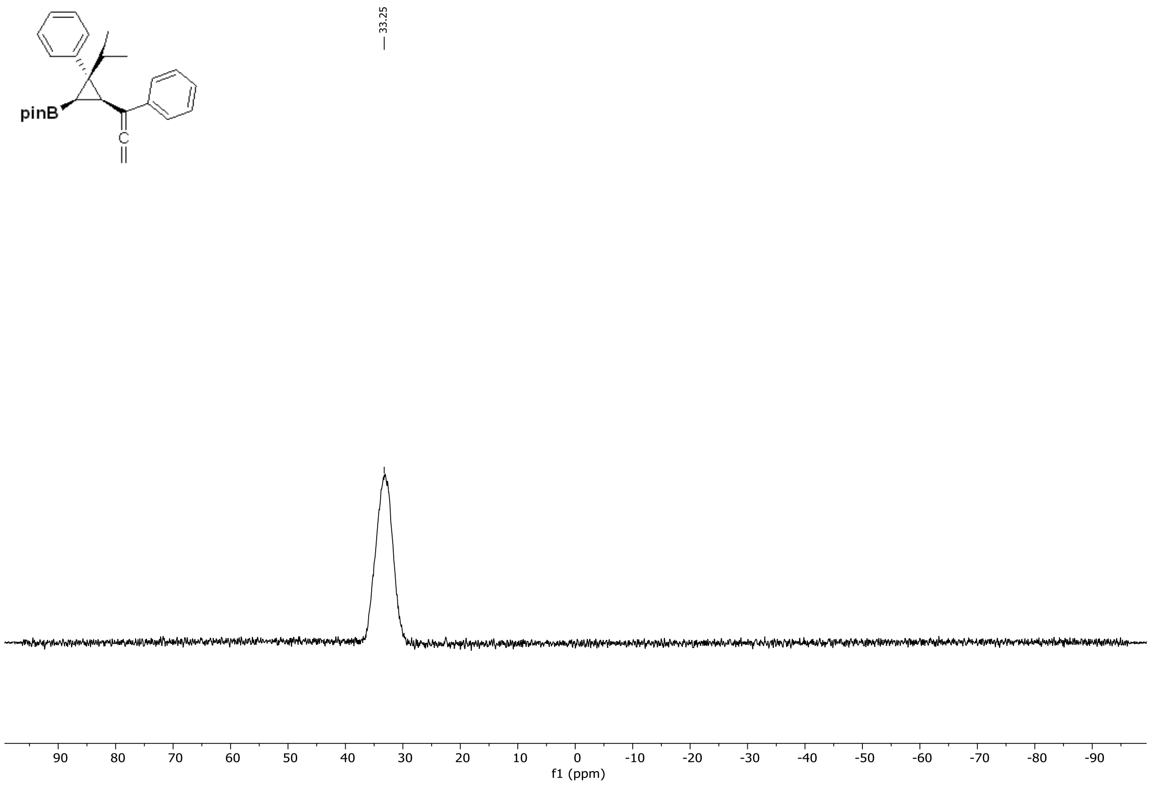

Compound 3d

^{1}H NMR (400 MHz, $CDCl_3$)

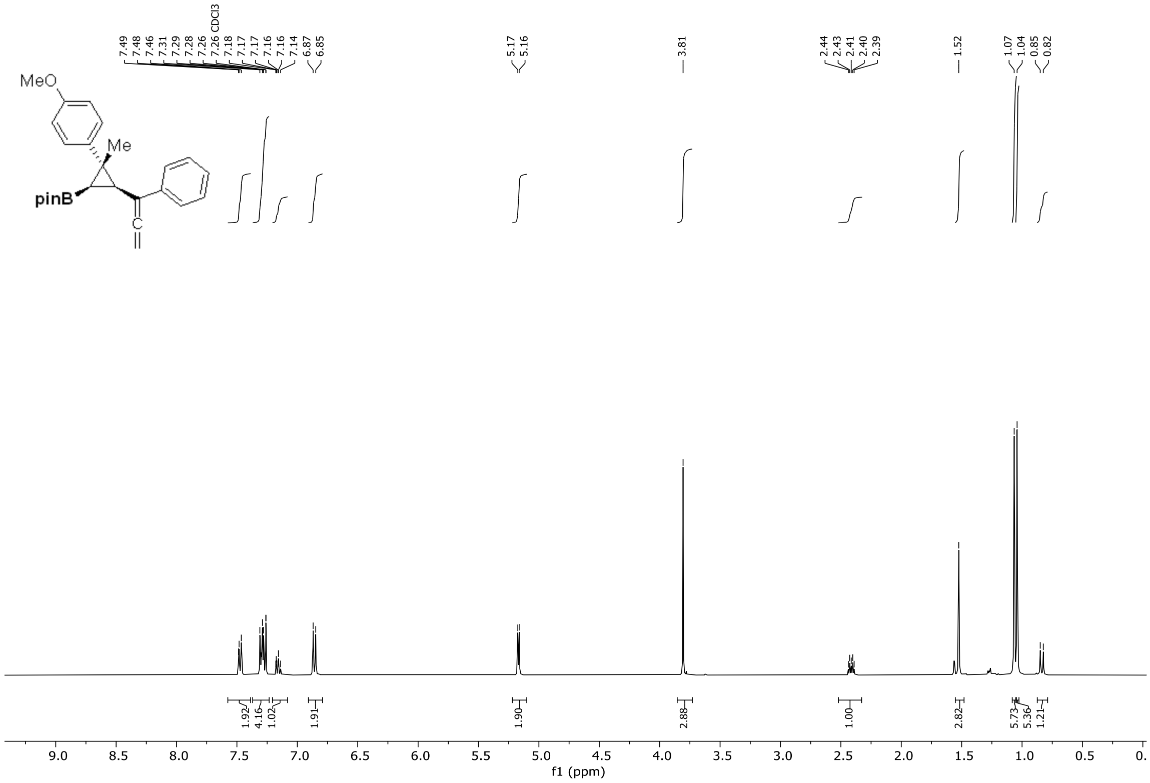

^{13}C NMR (101 MHz, $CDCl_3$)

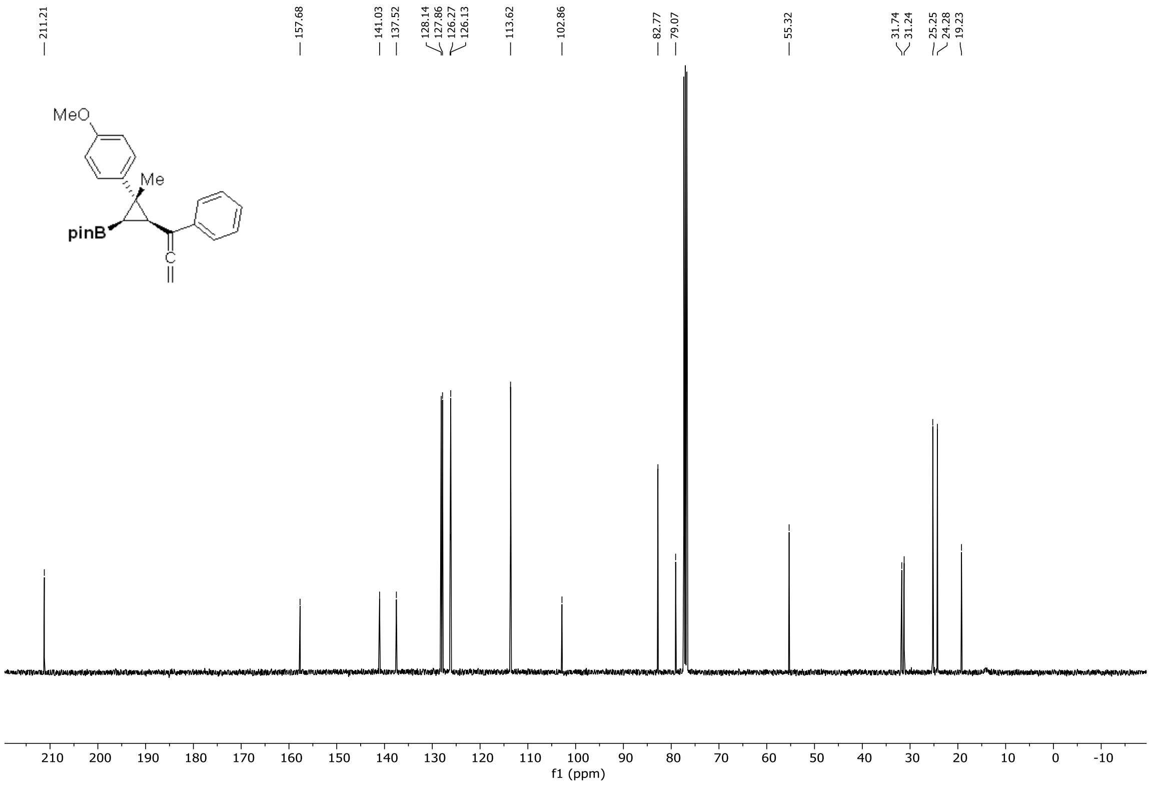

^{11}B NMR (128 MHz, $CDCl_3$)

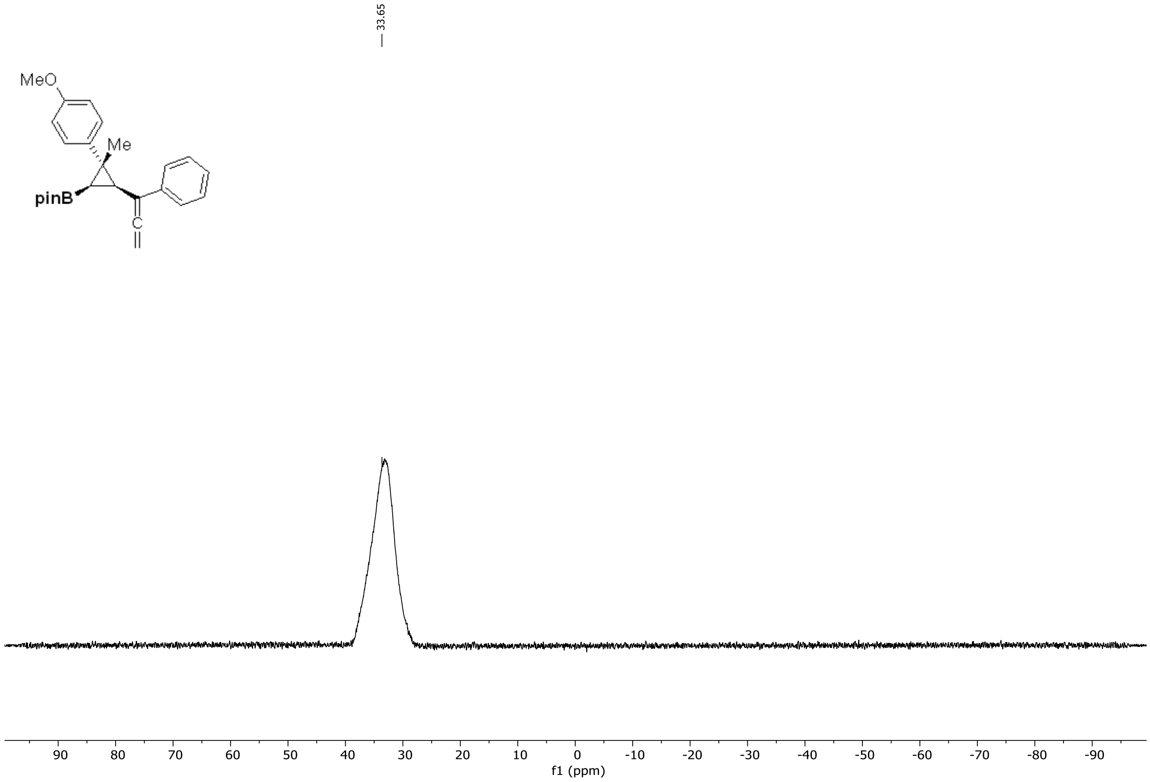

Compound 3e

^{1}H NMR (400 MHz, $CDCl_3$)

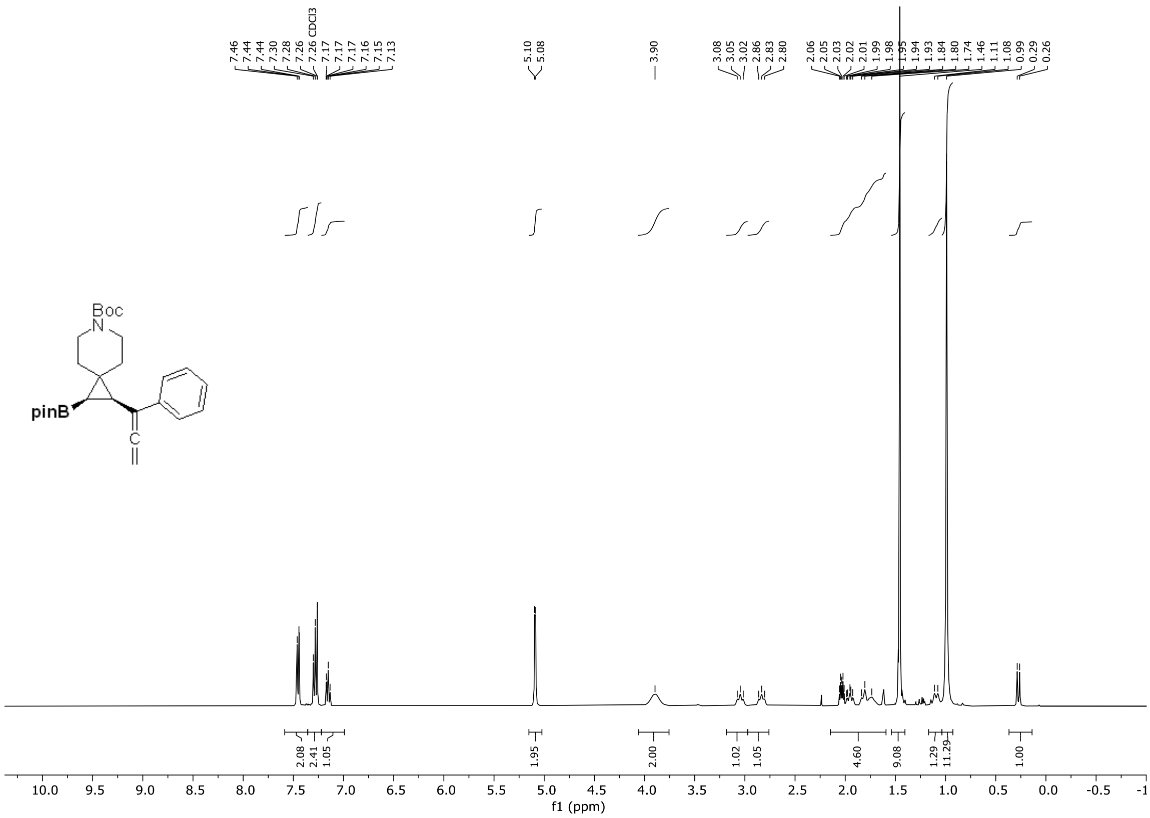

^{13}C NMR (101 MHz, $CDCl_3$)

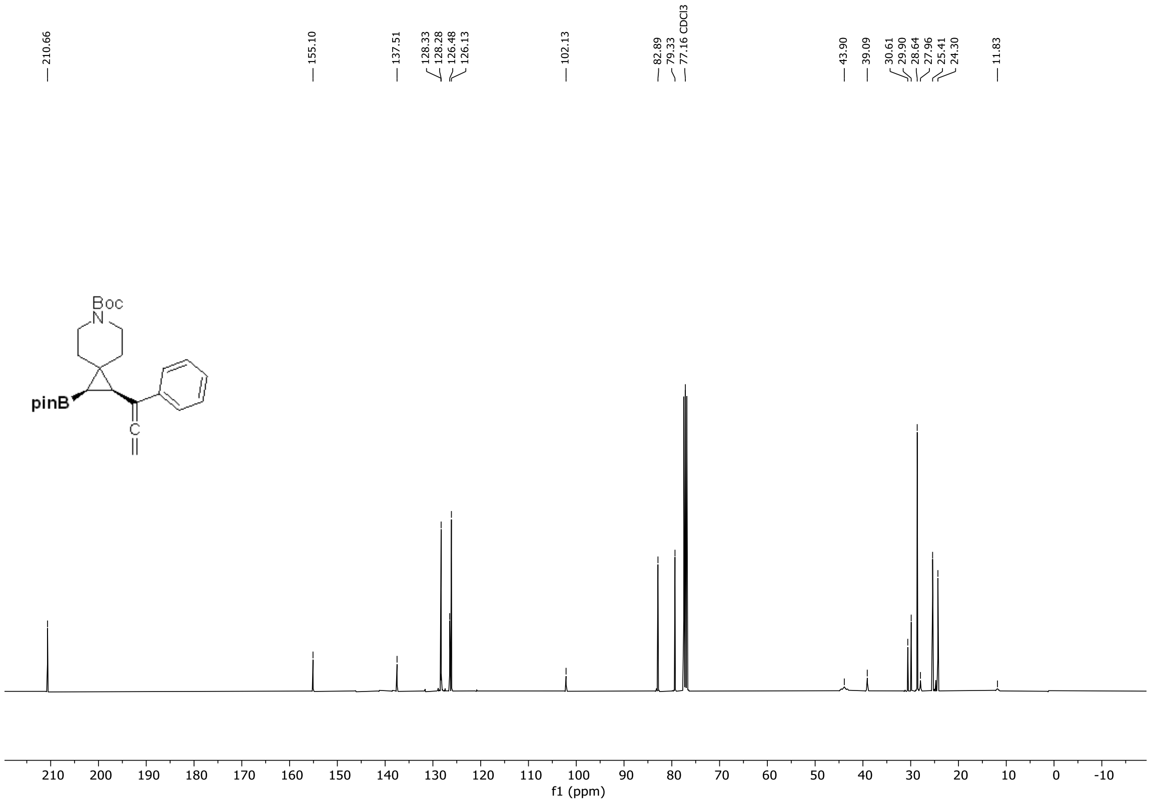

^{11}B NMR (128 MHz, $CDCl_3$)

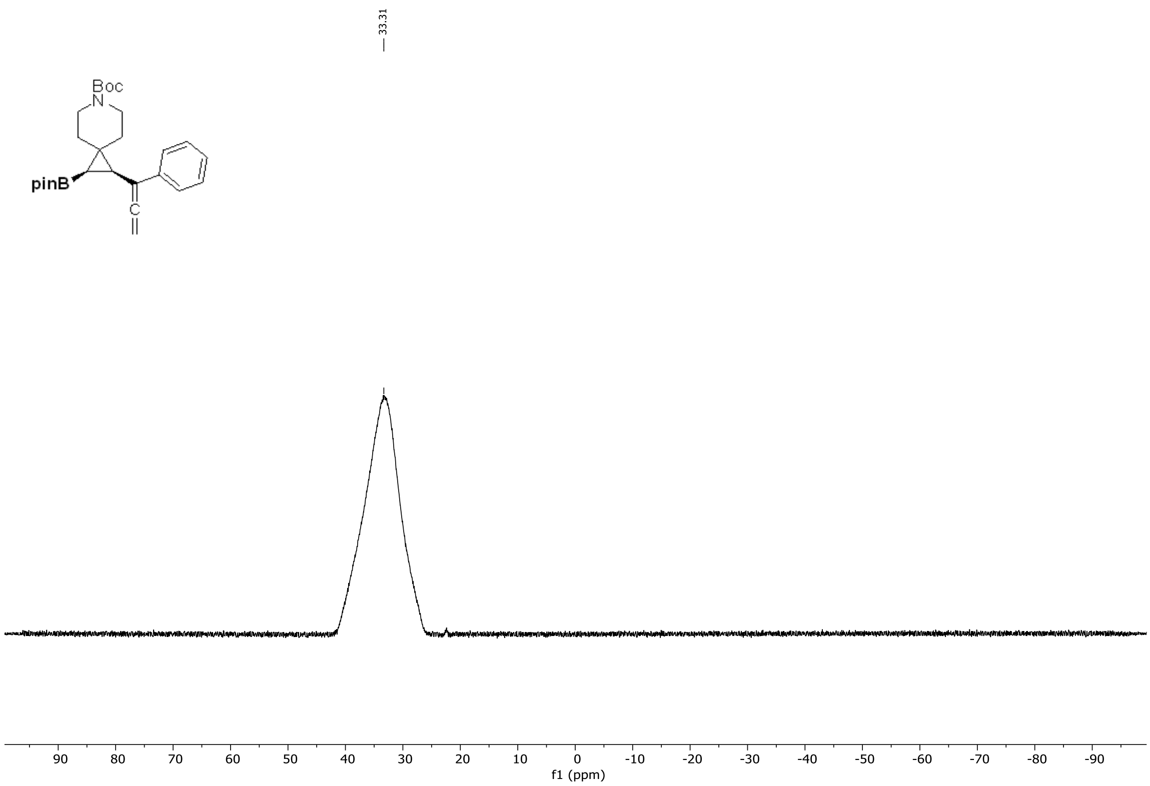

2D-COSY (400 MHz, $CDCl_3$)

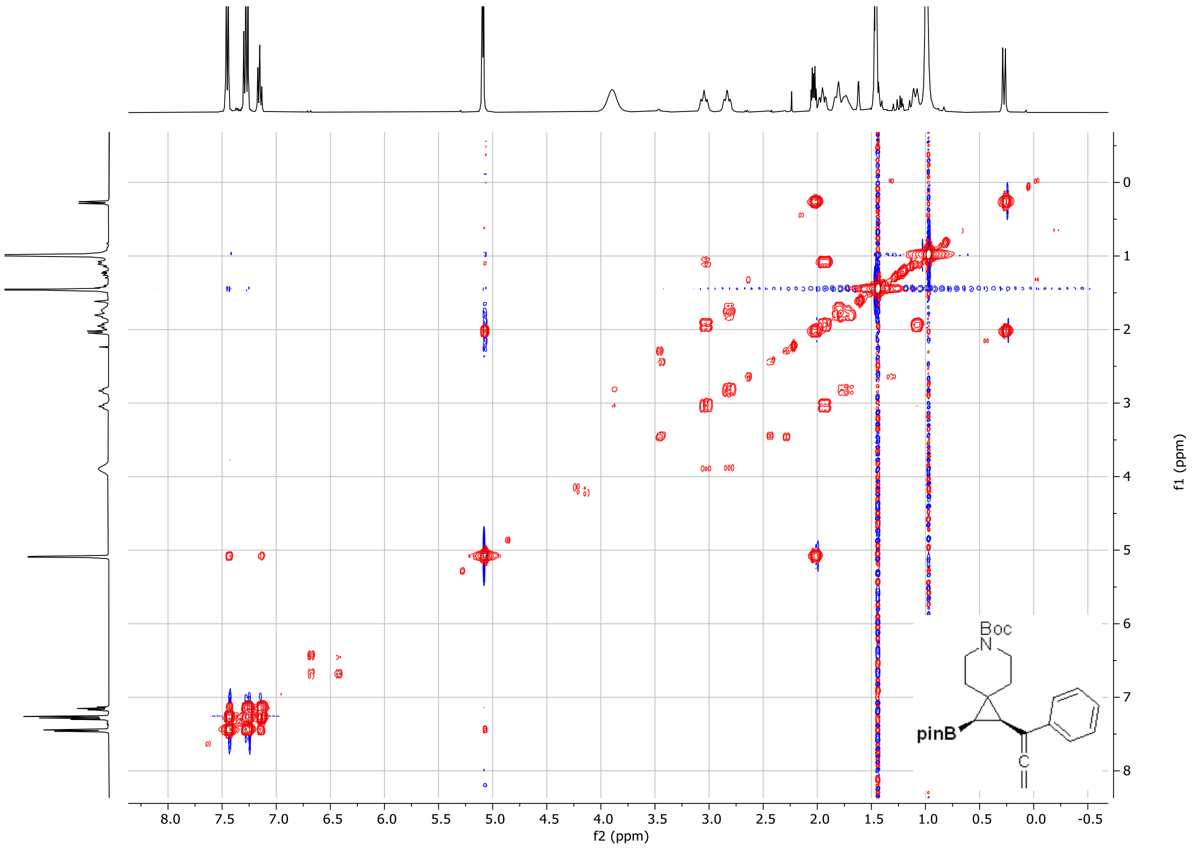

Compound 3f

^{1}H NMR (400 MHz, $CDCl_3$)

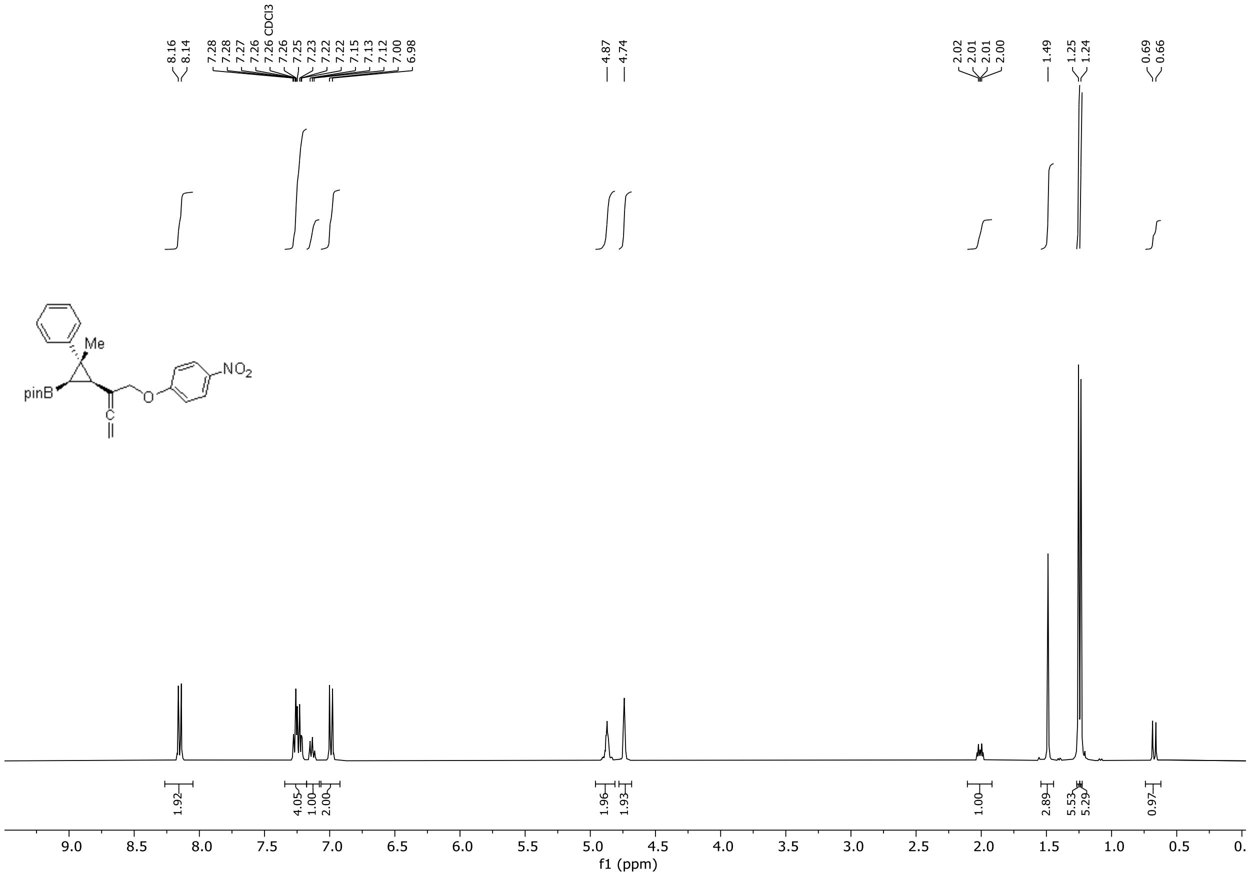

^{13}C NMR (101 MHz, $CDCl_3$)

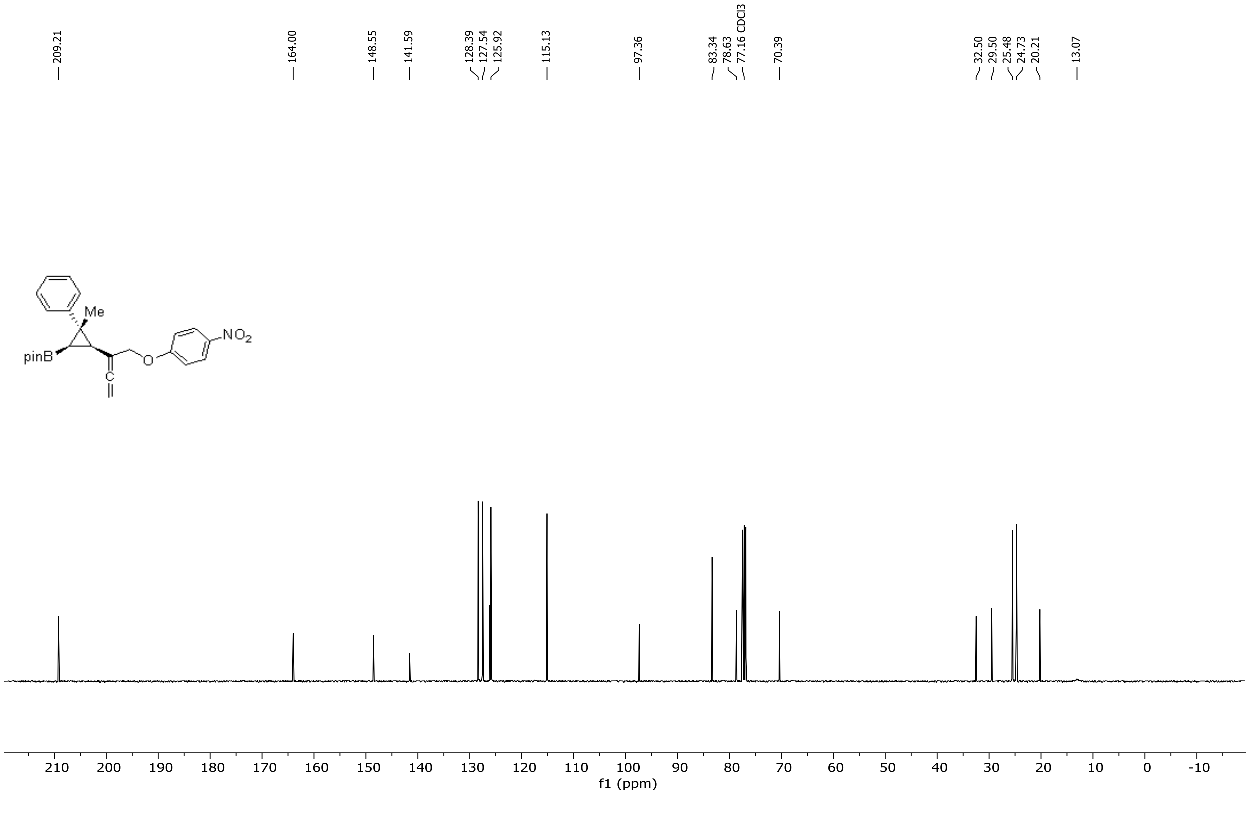

^{11}B NMR (128 MHz, $CDCl_3$)

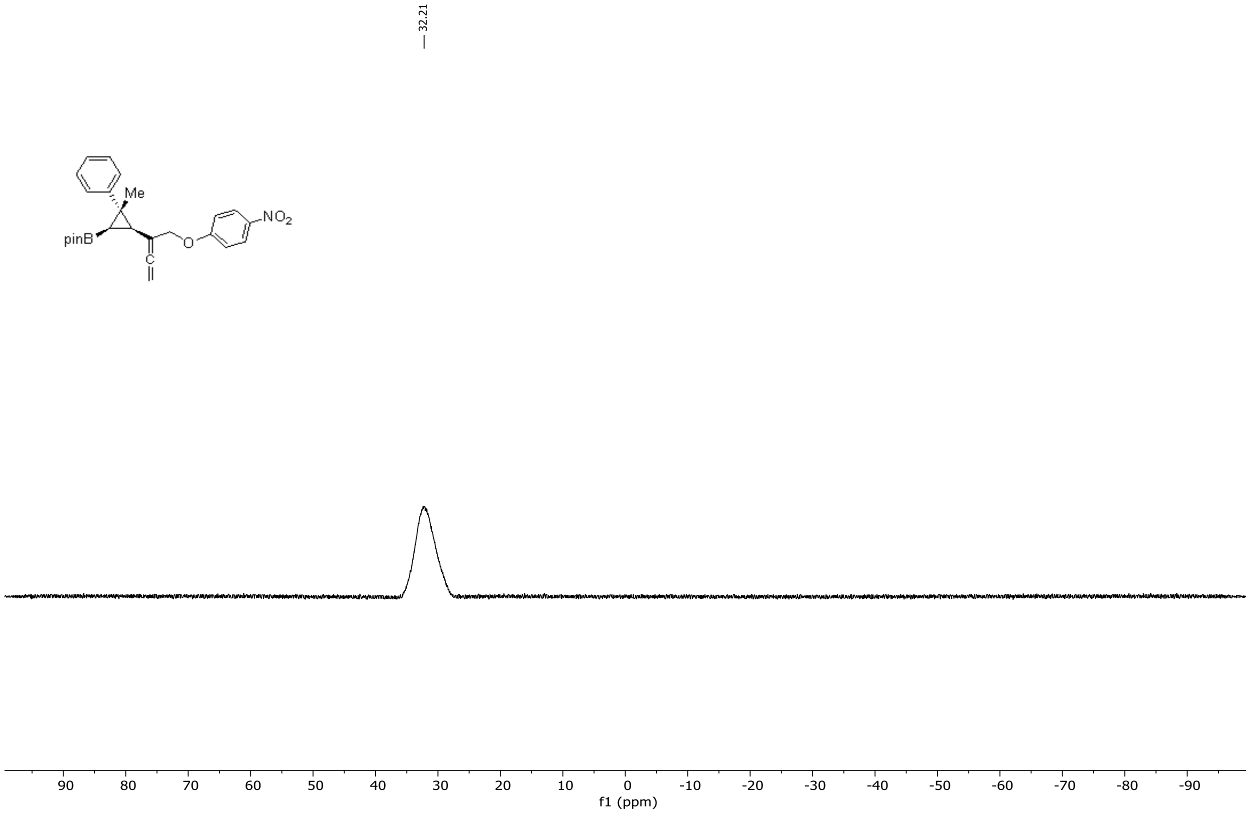

Compound 3g

^{1}H NMR (400 MHz, $CDCl_3$)

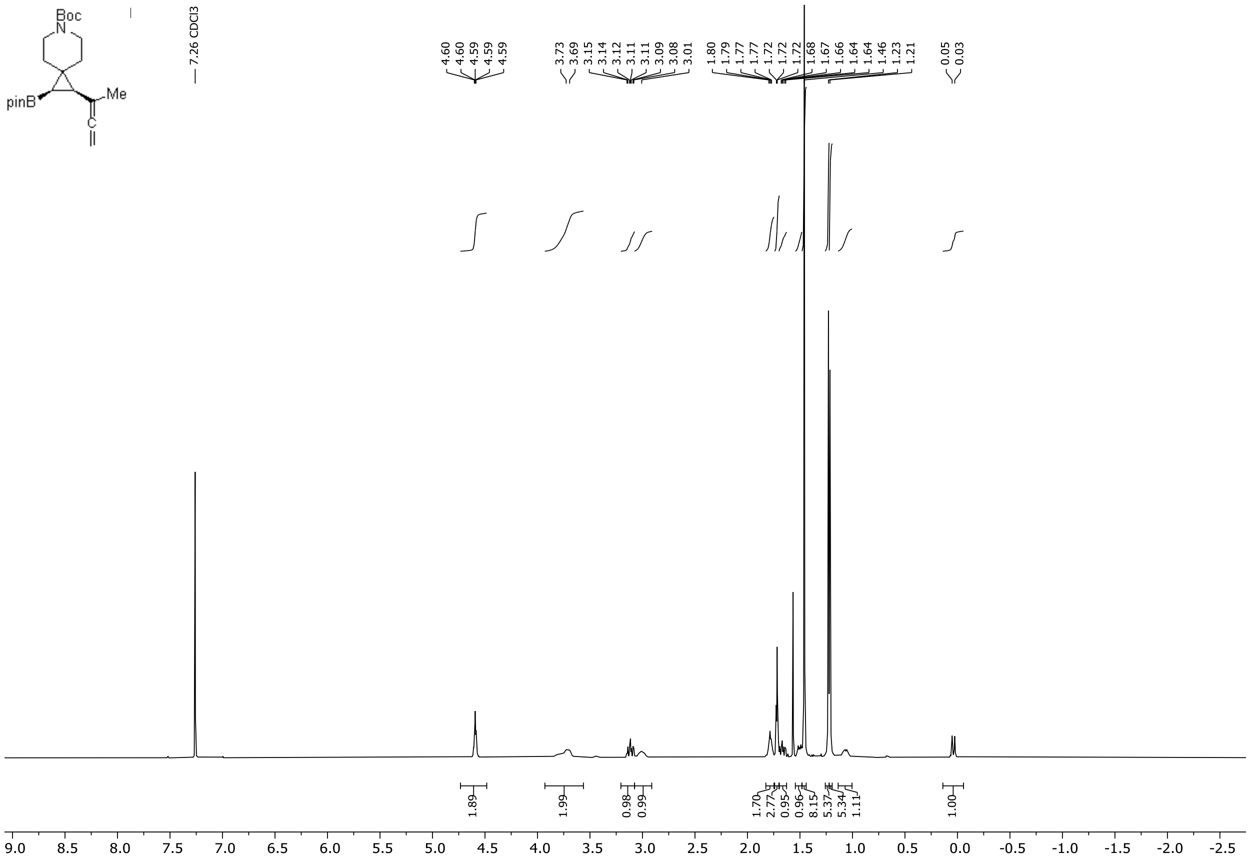

^{13}C NMR (101 MHz, $CDCl_3$)

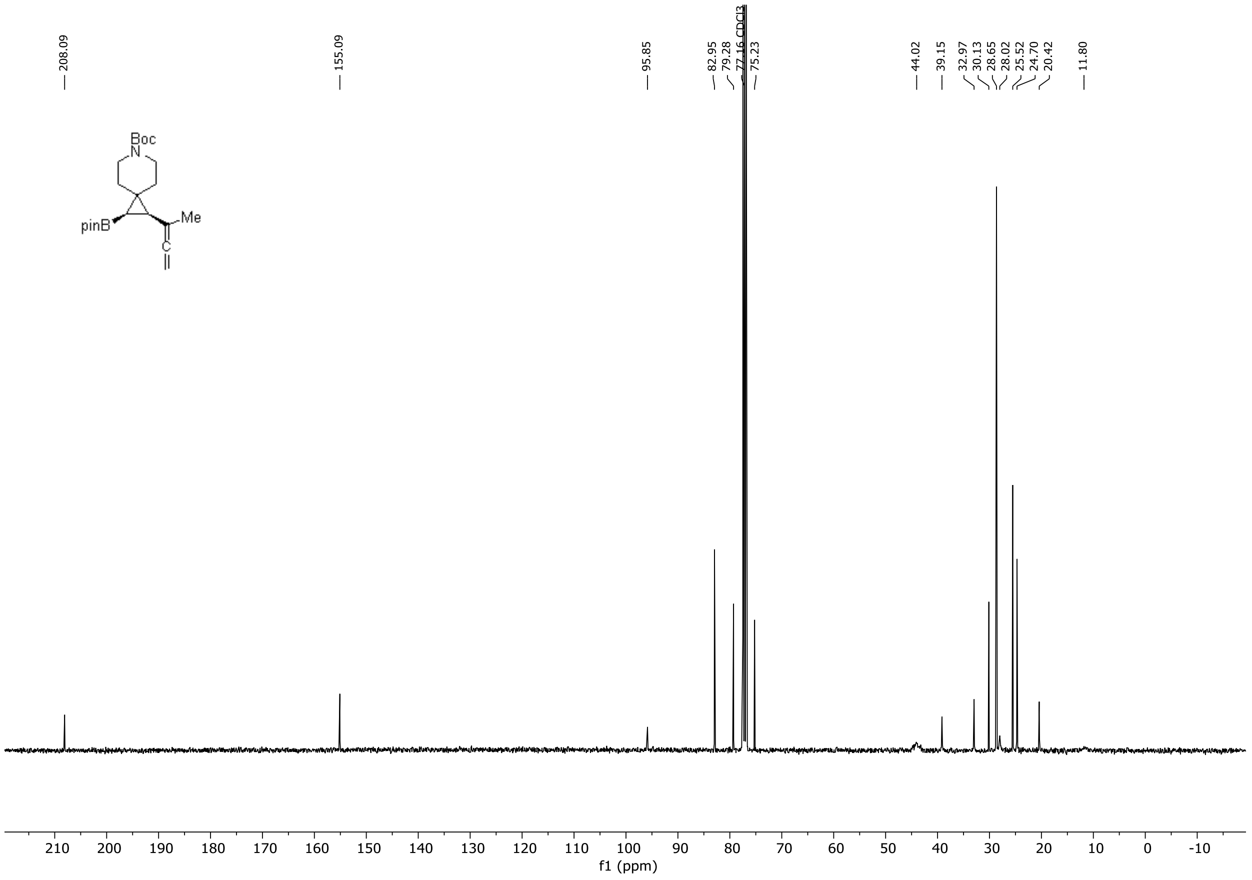

^{11}B NMR (128 MHz, $CDCl_3$)

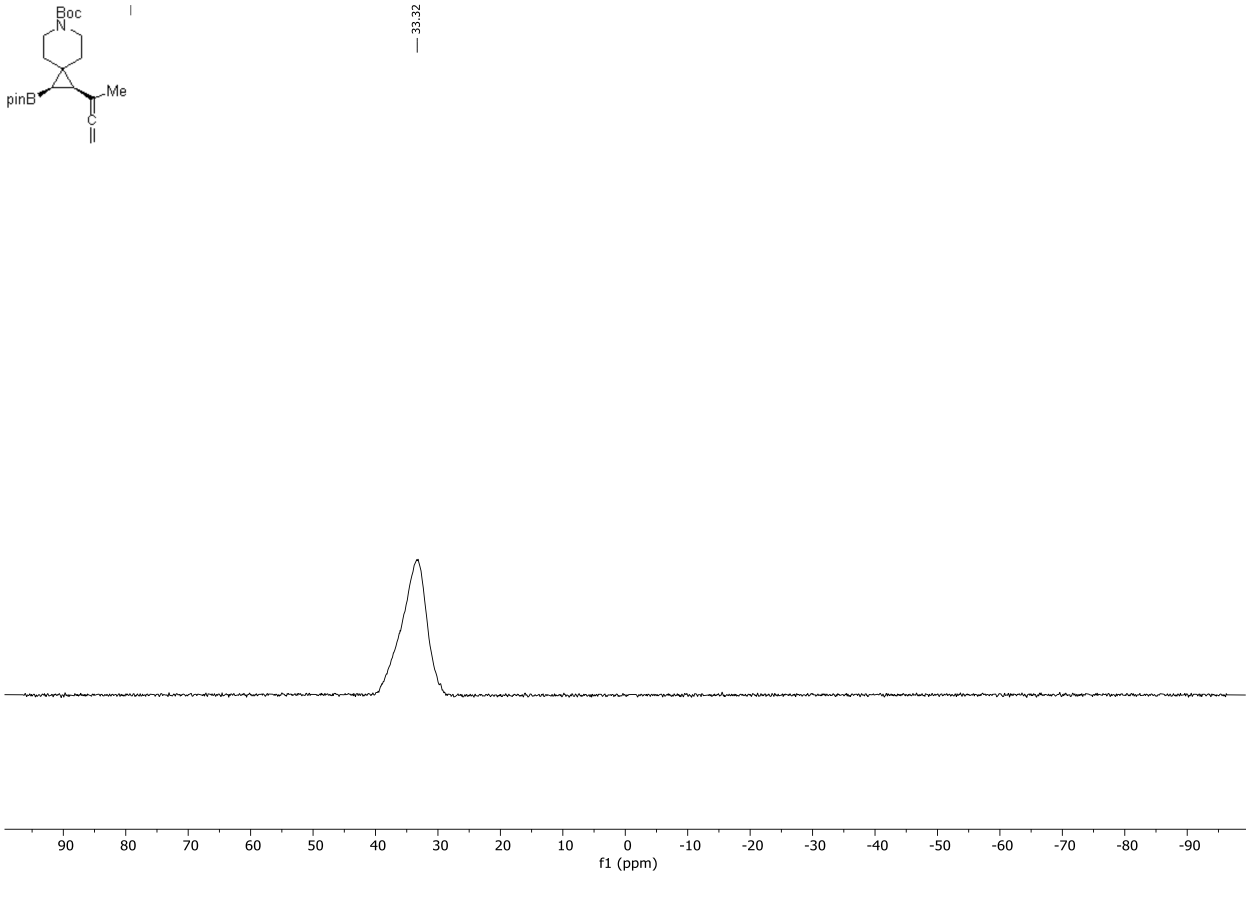

Compound 4a

^{1}H NMR (400 MHz, $CDCl_3$)

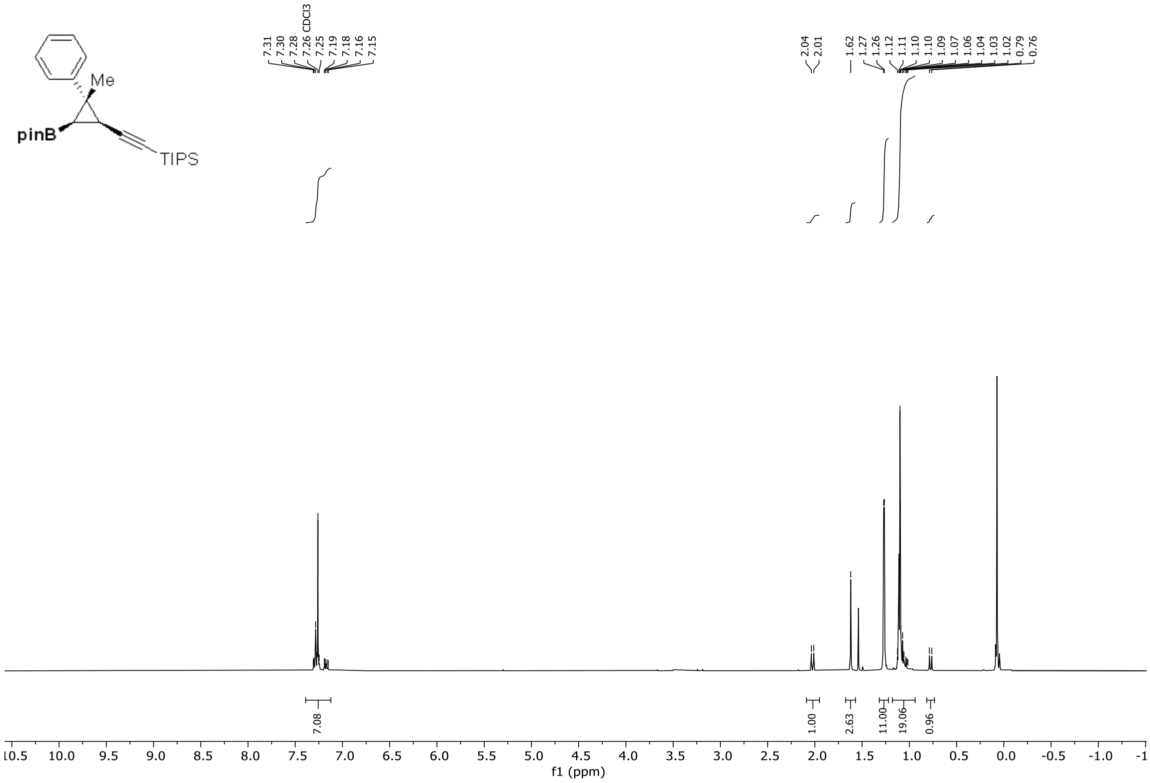

^{13}C NMR (101 MHz, $CDCl_3$)

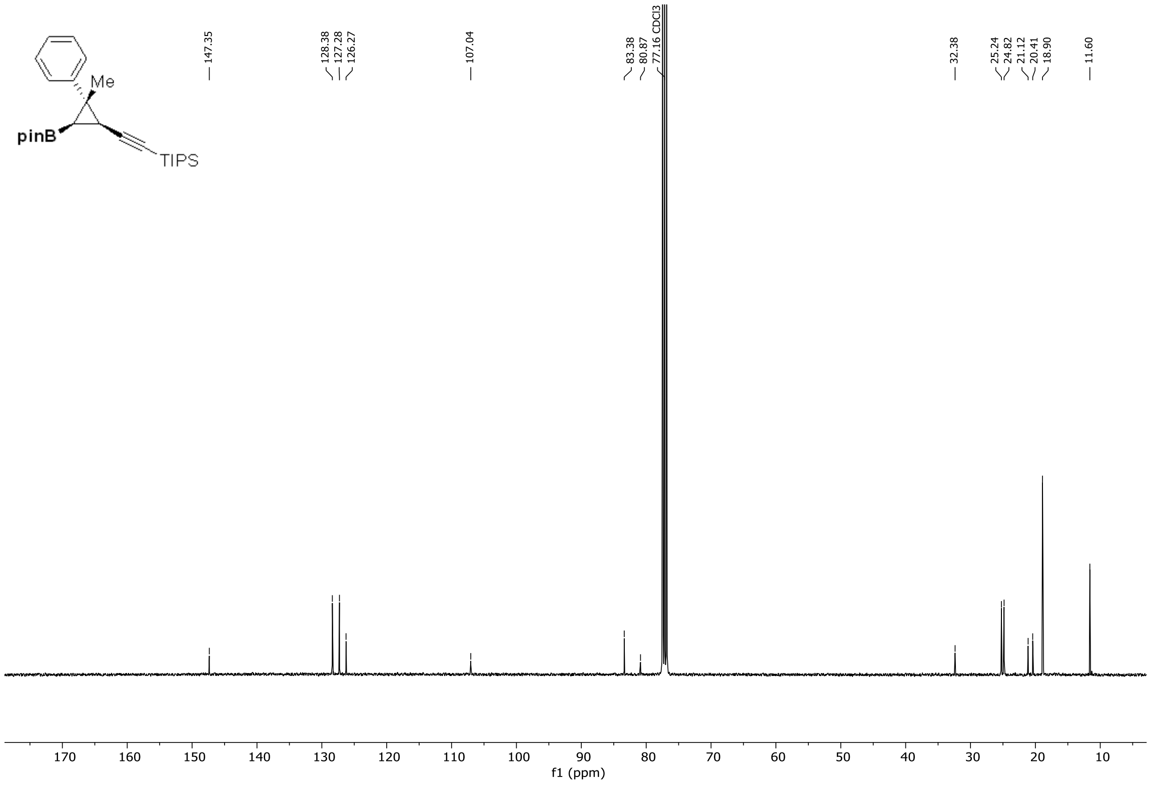

^{11}B NMR (128 MHz, $CDCl_3$)

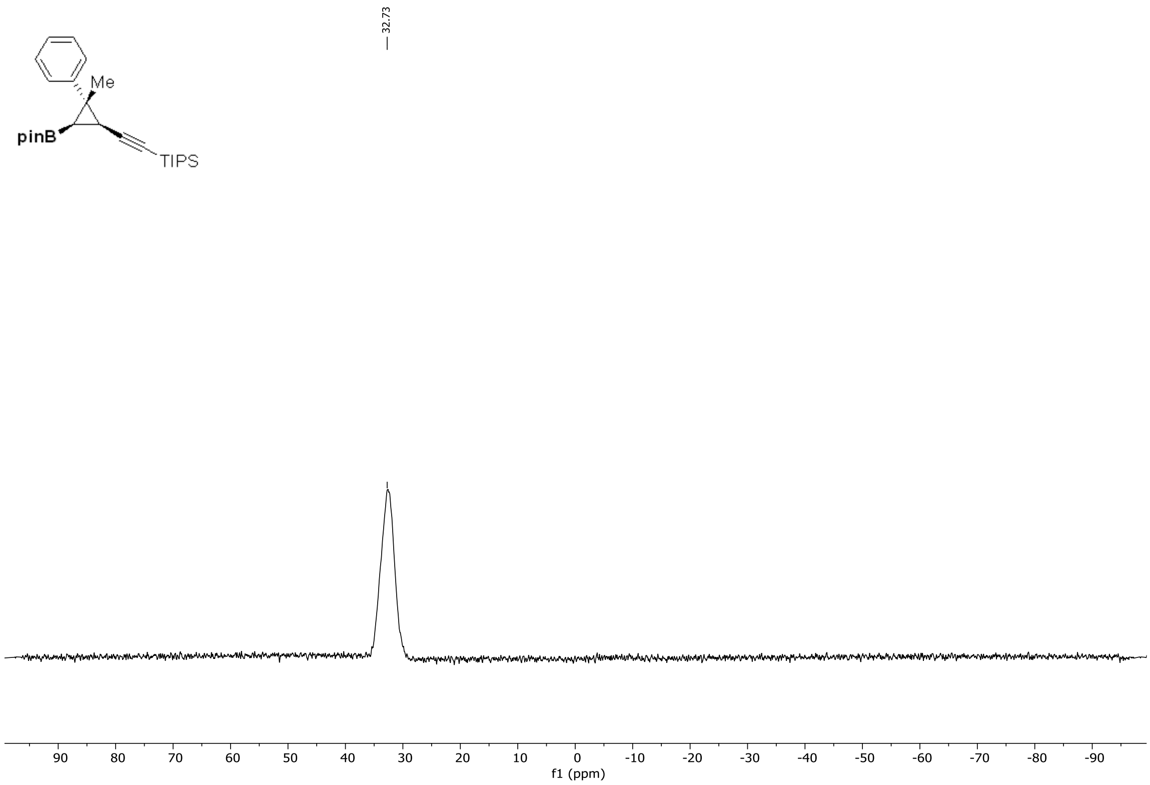

Compound 4b

^{1}H NMR (300 MHz, $CDCl_3$)

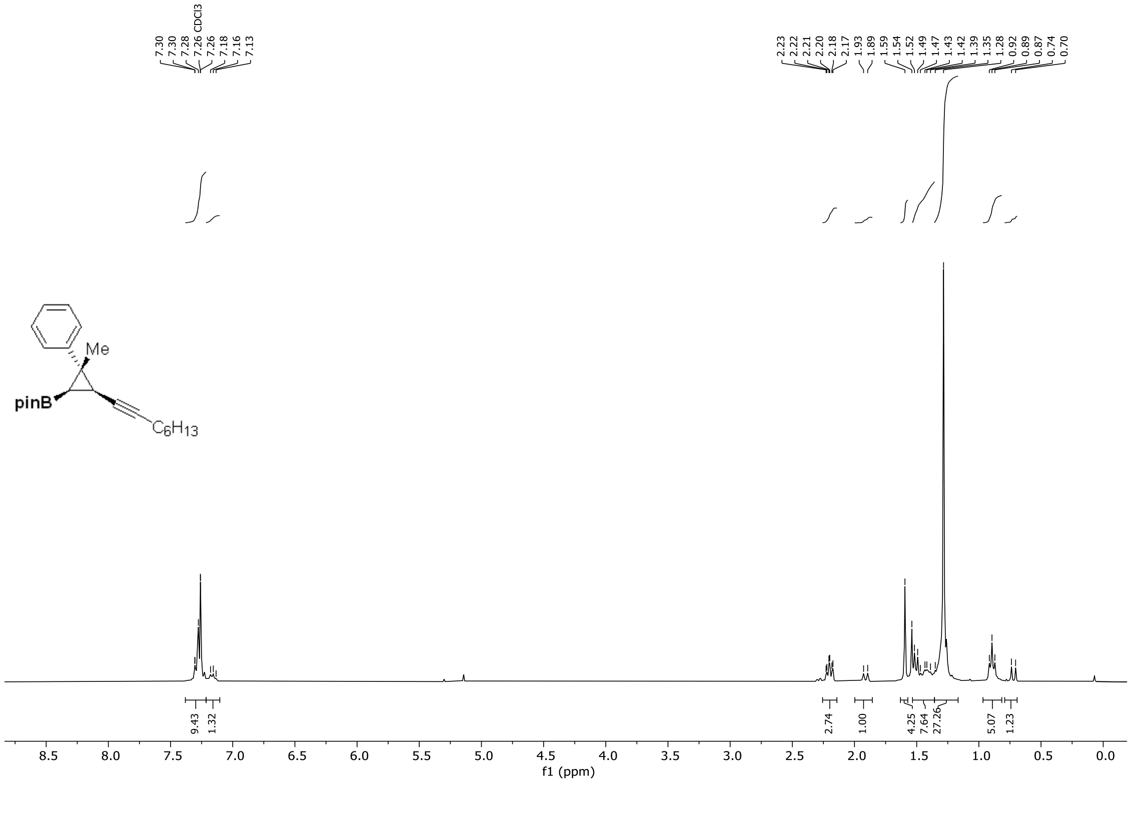

^{13}C NMR (101 MHz, $CDCl_3$)

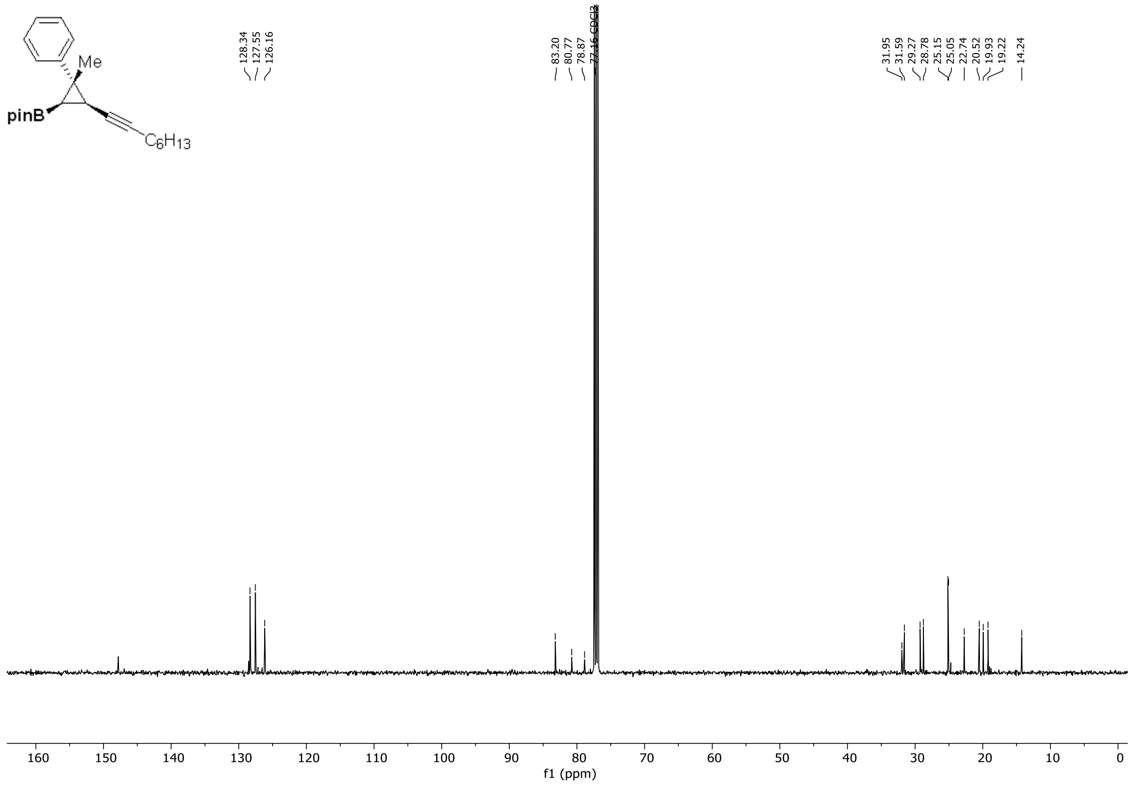

^{11}B NMR (128 MHz, $CDCl_3$)

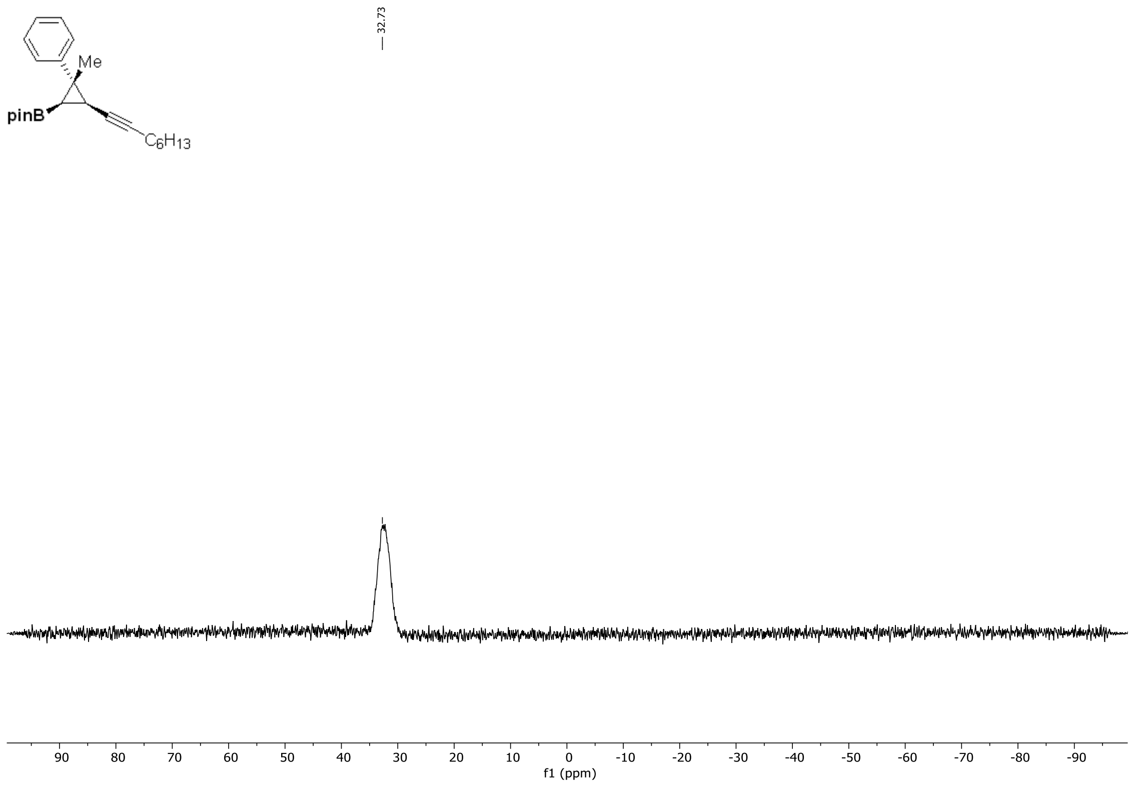

Compound 4c

^{1}H NMR (400 MHz, CDCl$_3$)

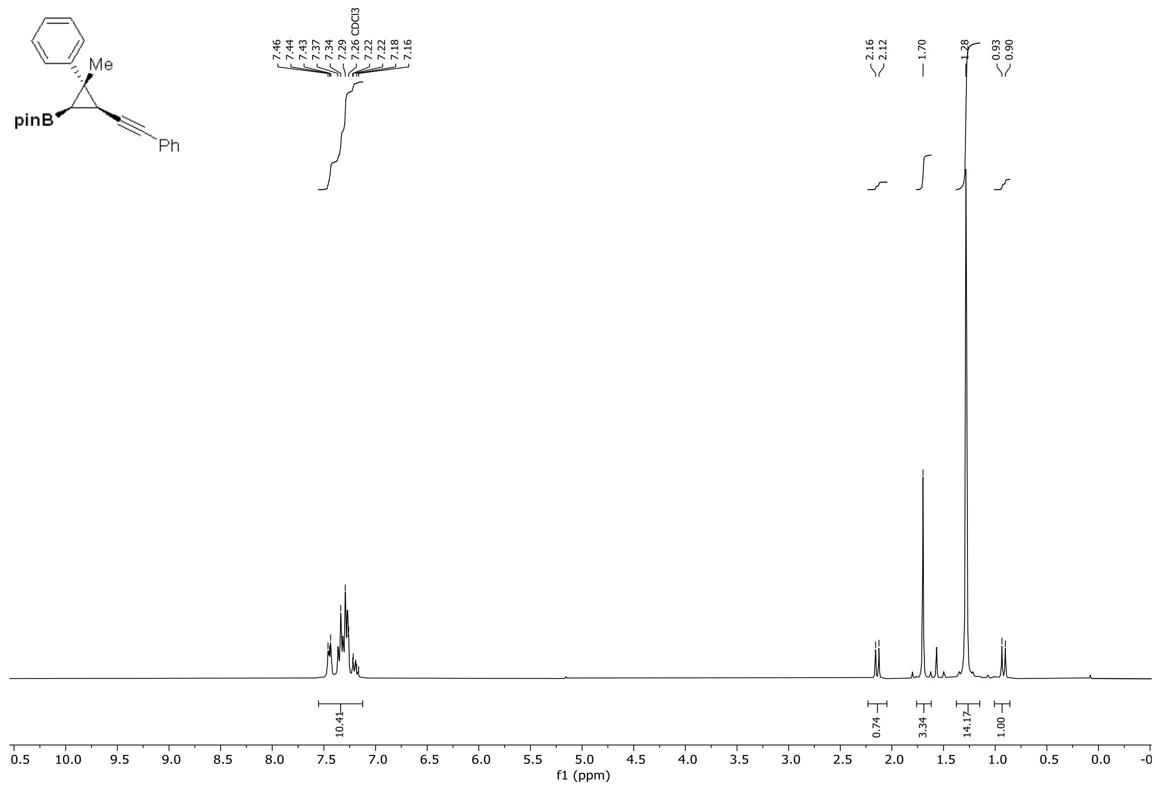

^{13}C NMR (101 MHz, CDCl$_3$)

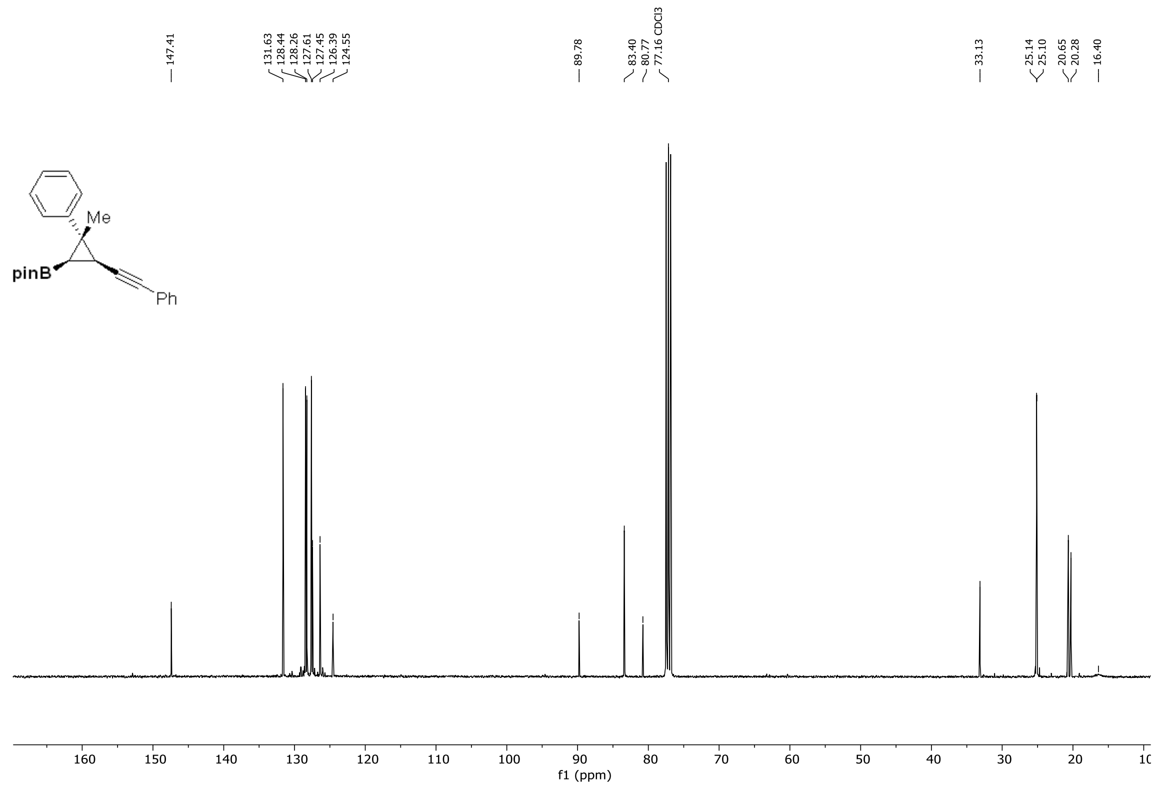

^{11}B NMR (128 MHz, $CDCl_3$)

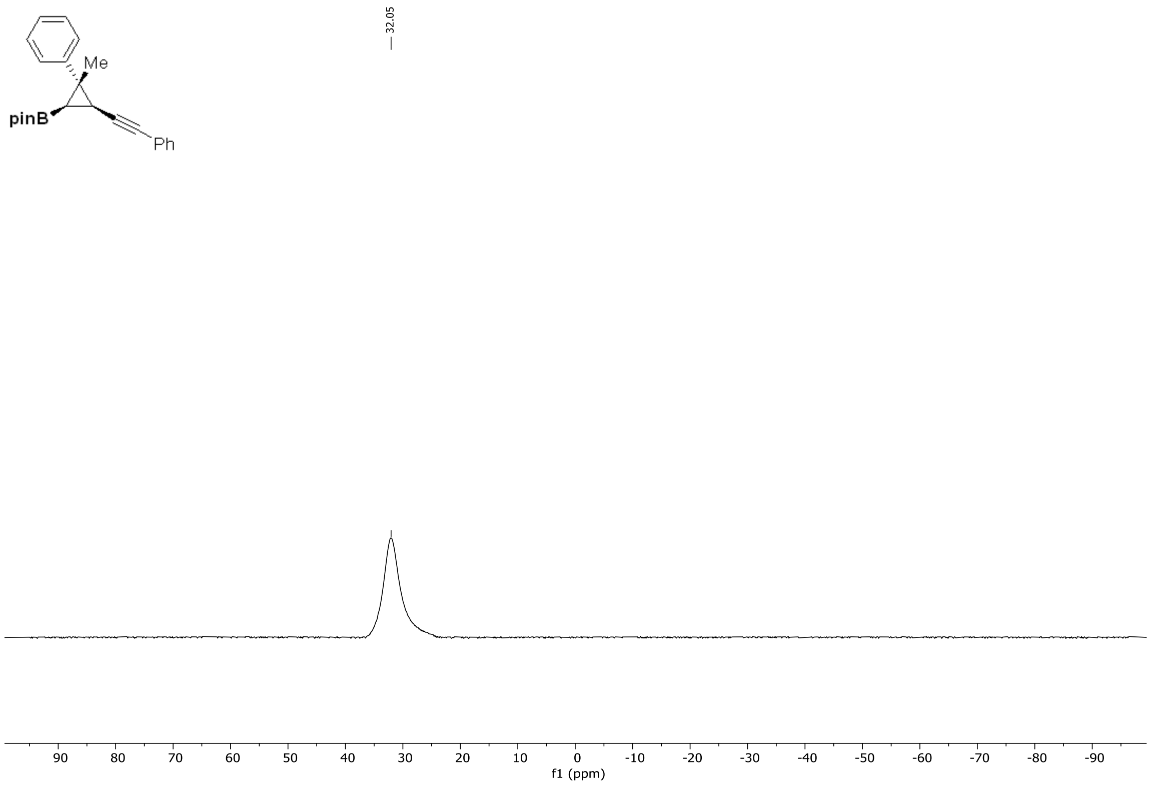

2D-NOESY (400 MHz, $CDCl_3$)

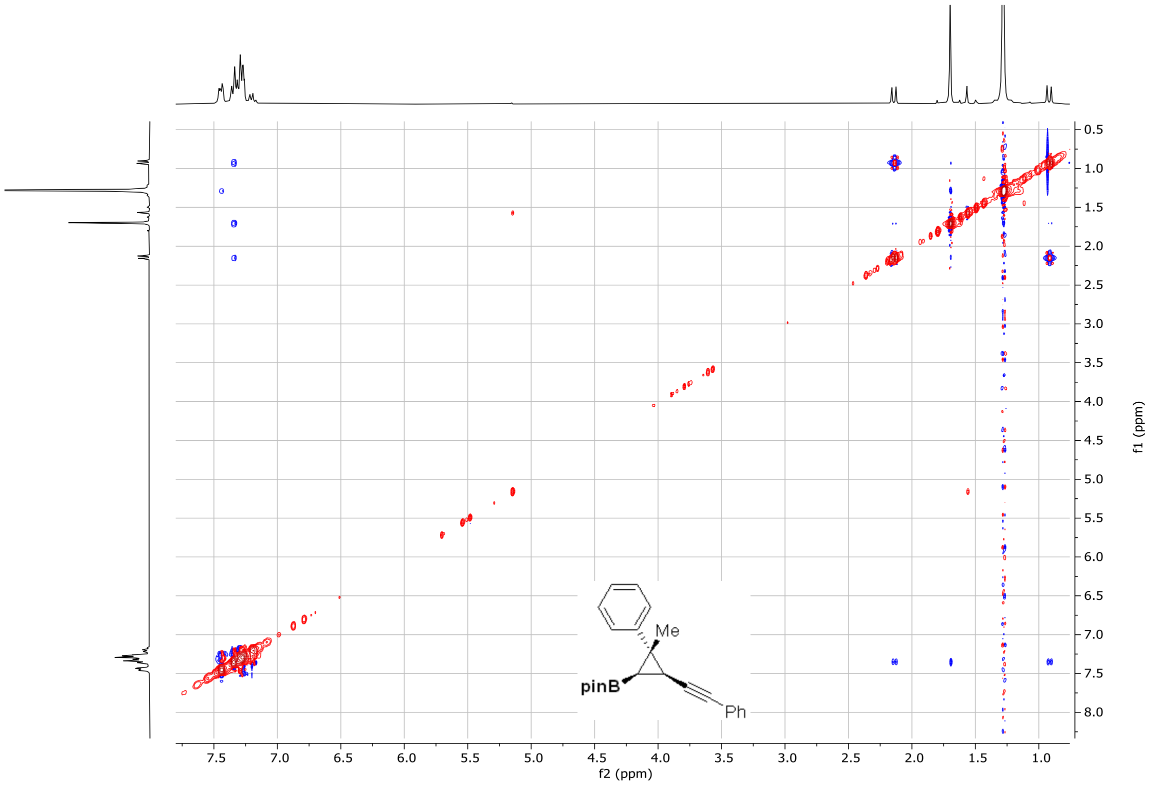

Compound 4d

^{1}H NMR (300 MHz, $CDCl_3$)

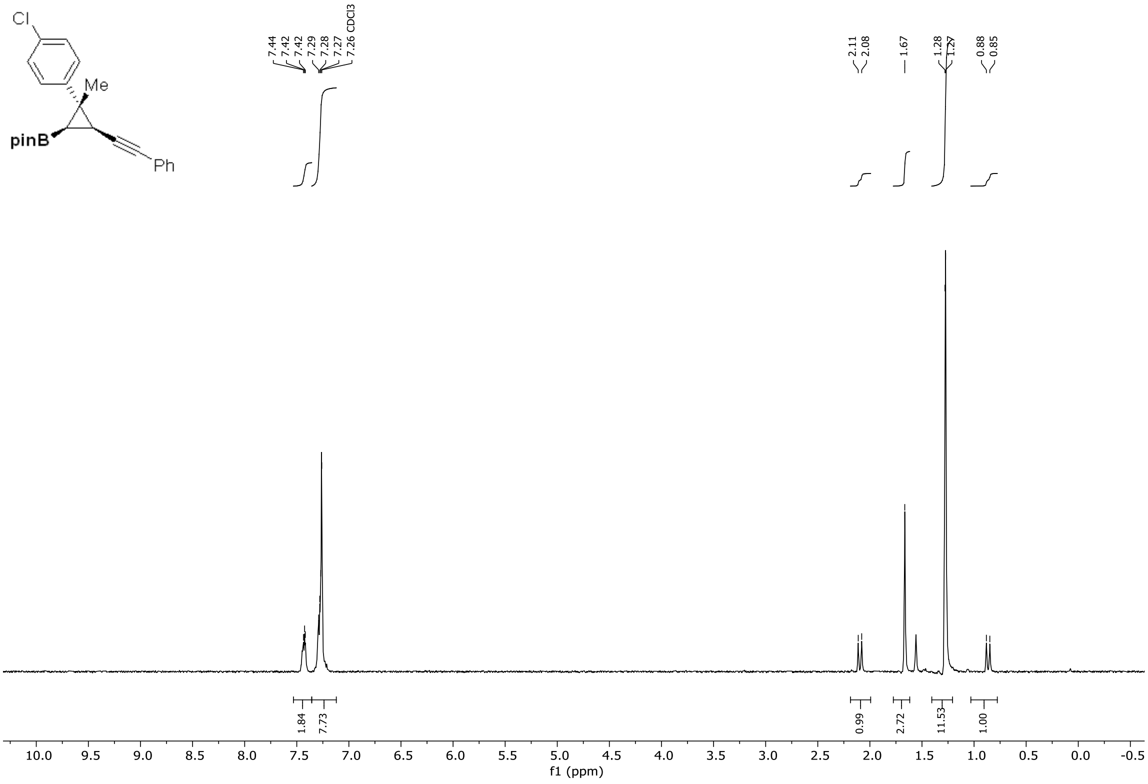

^{13}C NMR (101 MHz, $CDCl_3$)

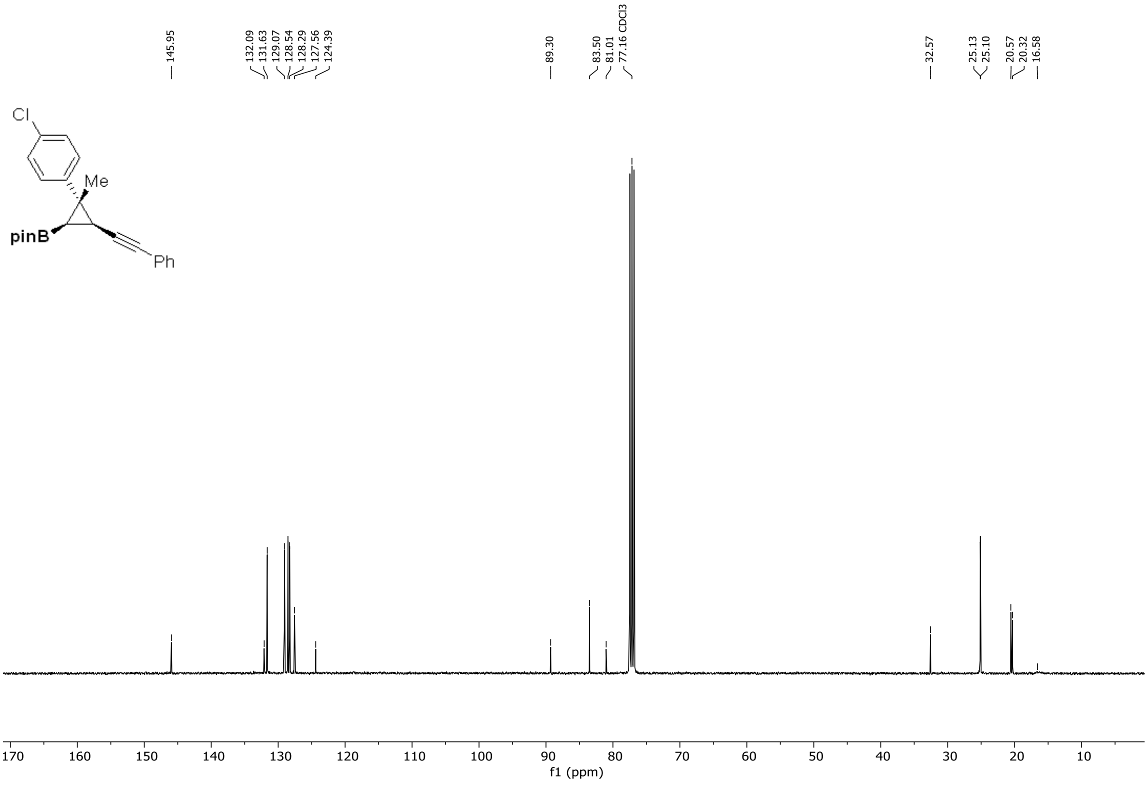

^{11}B NMR (128 MHz, $CDCl_3$)

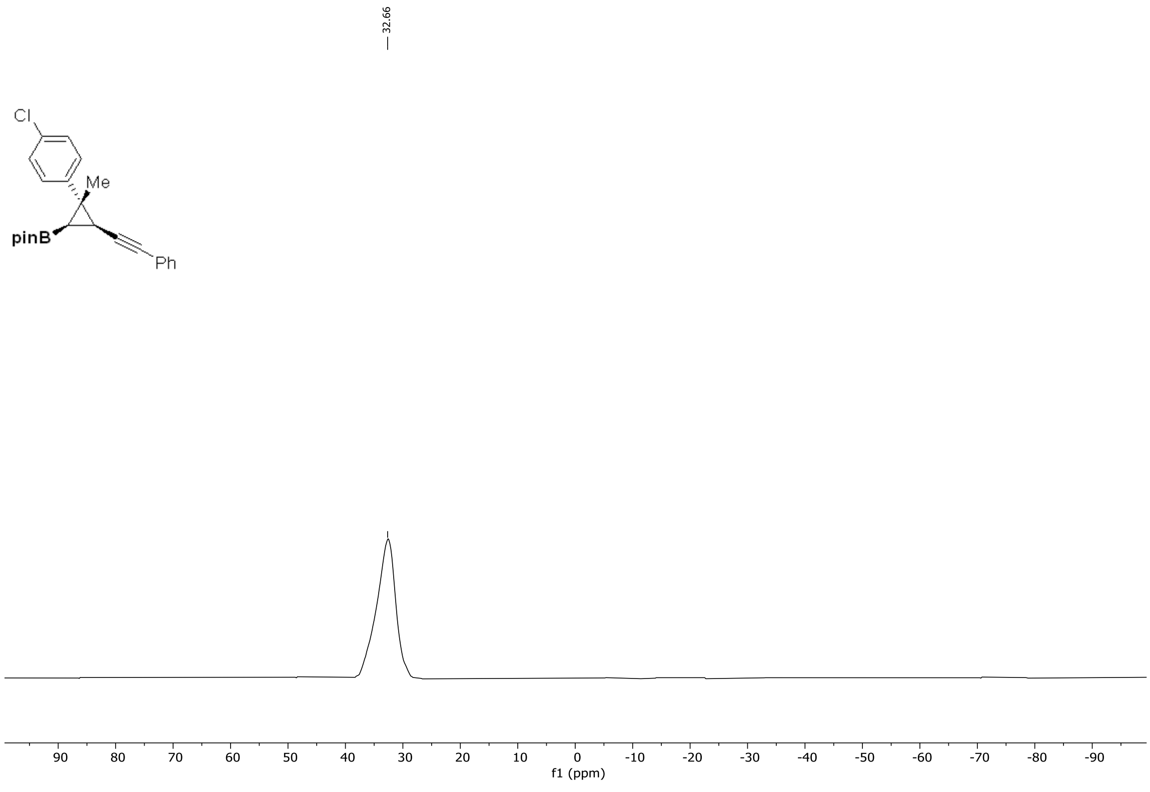

Compound 4e

^{1}H NMR (400 MHz, $CDCl_3$)

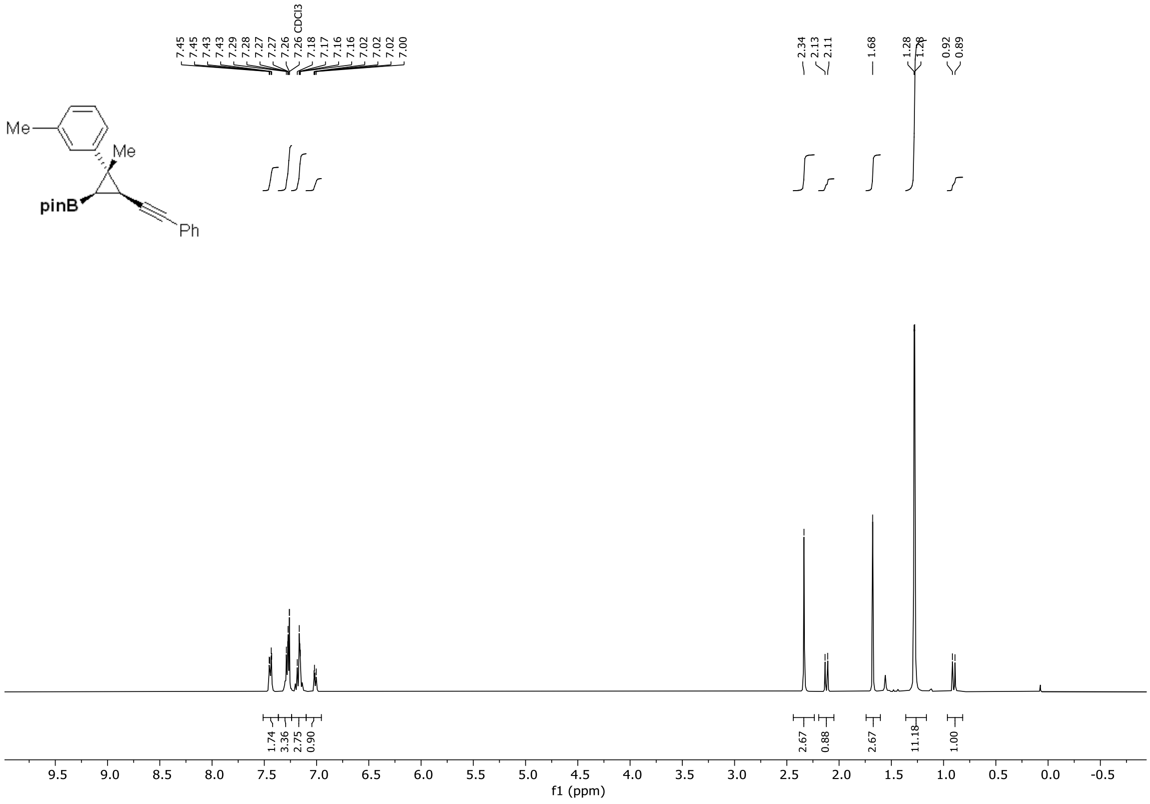

^{13}C NMR (101 MHz, $CDCl_3$)

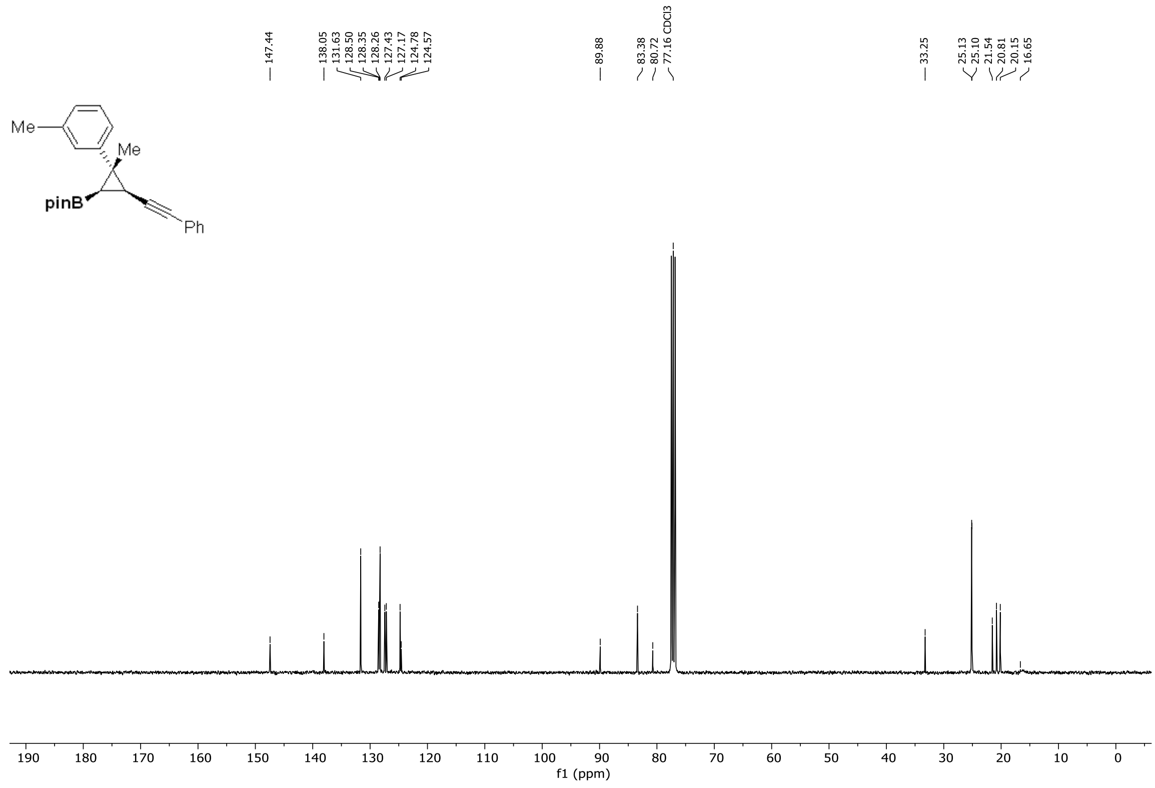

^{11}B NMR (128 MHz, $CDCl_3$)

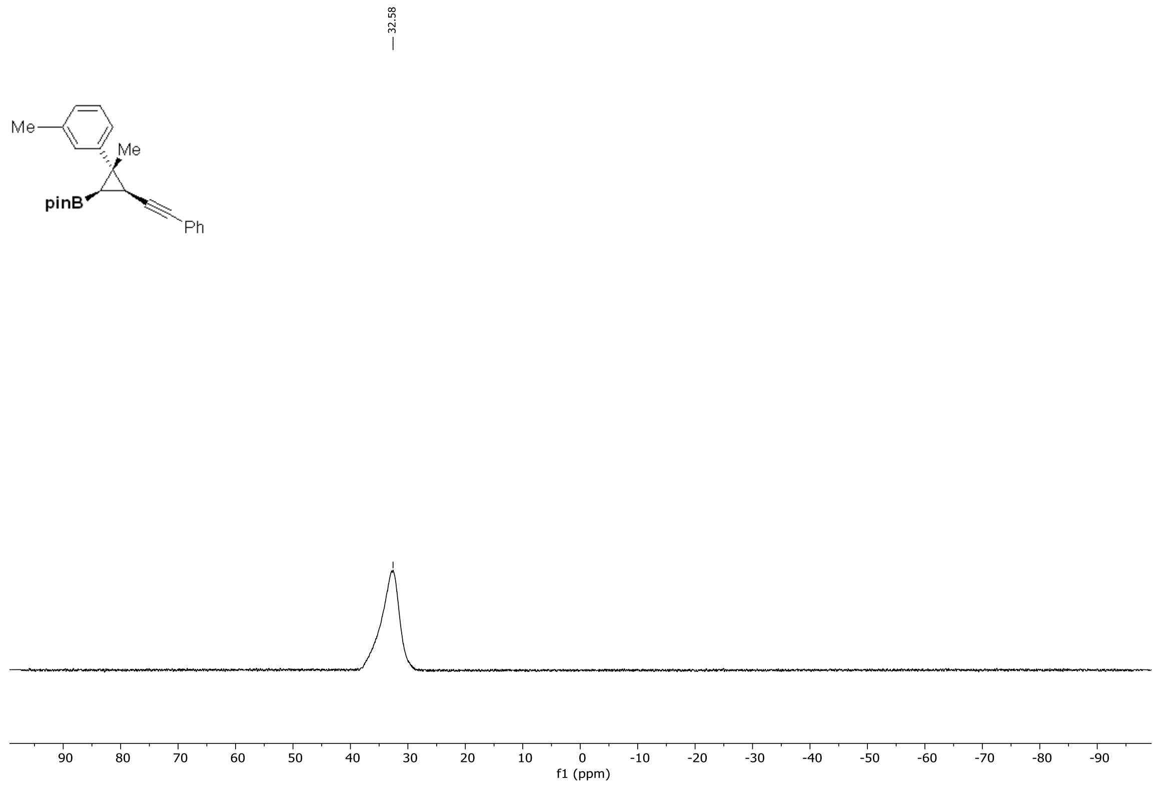

Compound 4f

^{1}H NMR (400 MHz, $CDCl_3$)

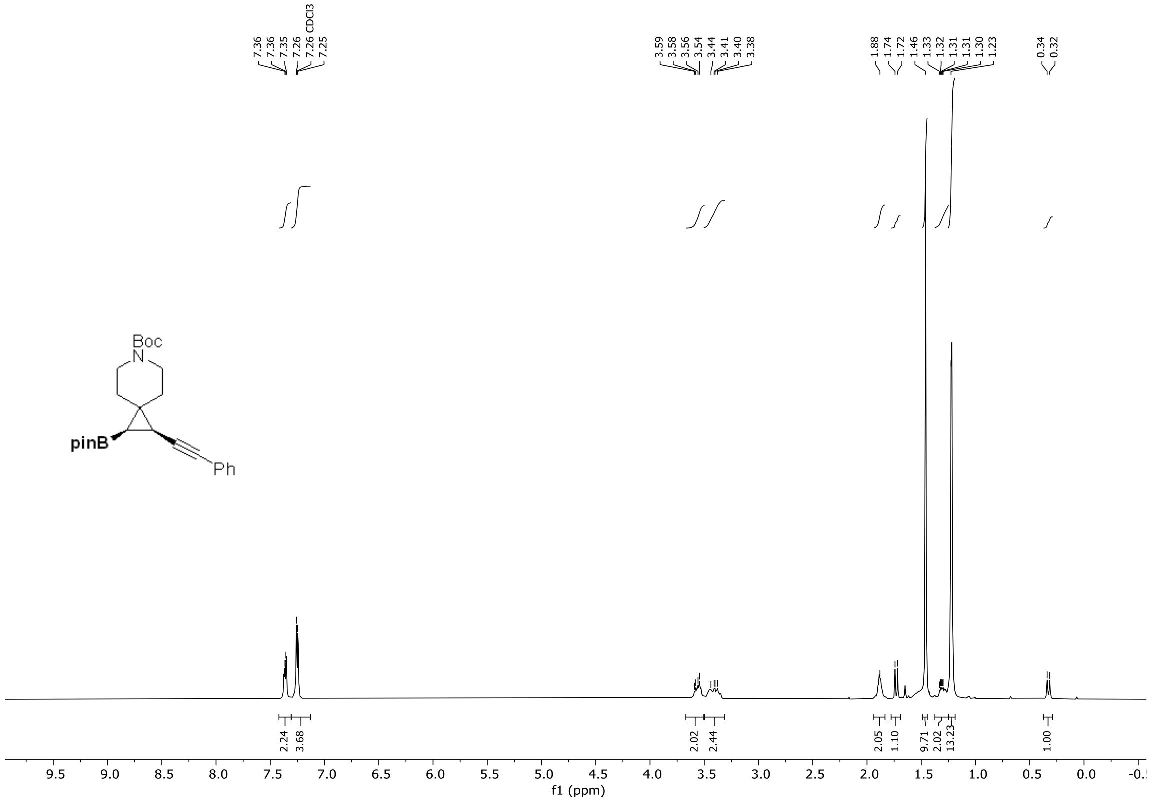

^{13}C NMR (101 MHz, $CDCl_3$)

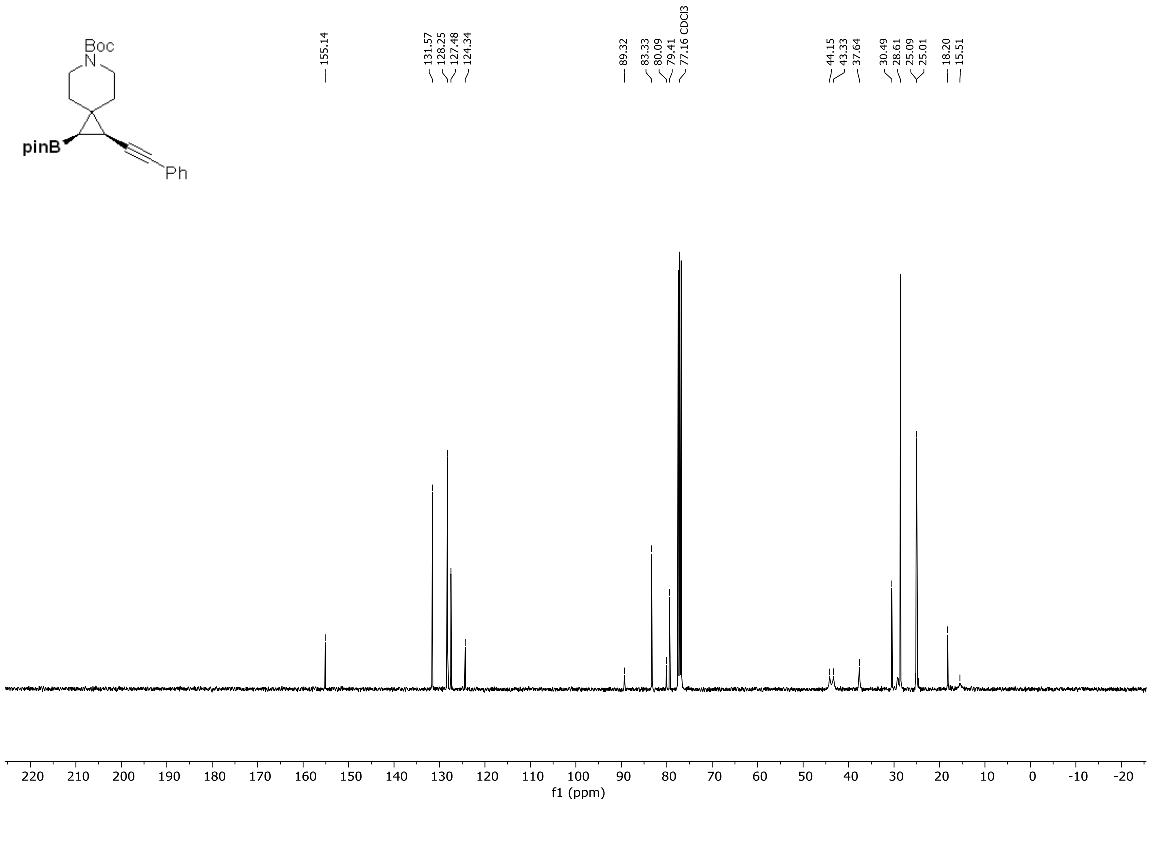

^{11}B NMR (128 MHz, $CDCl_3$)

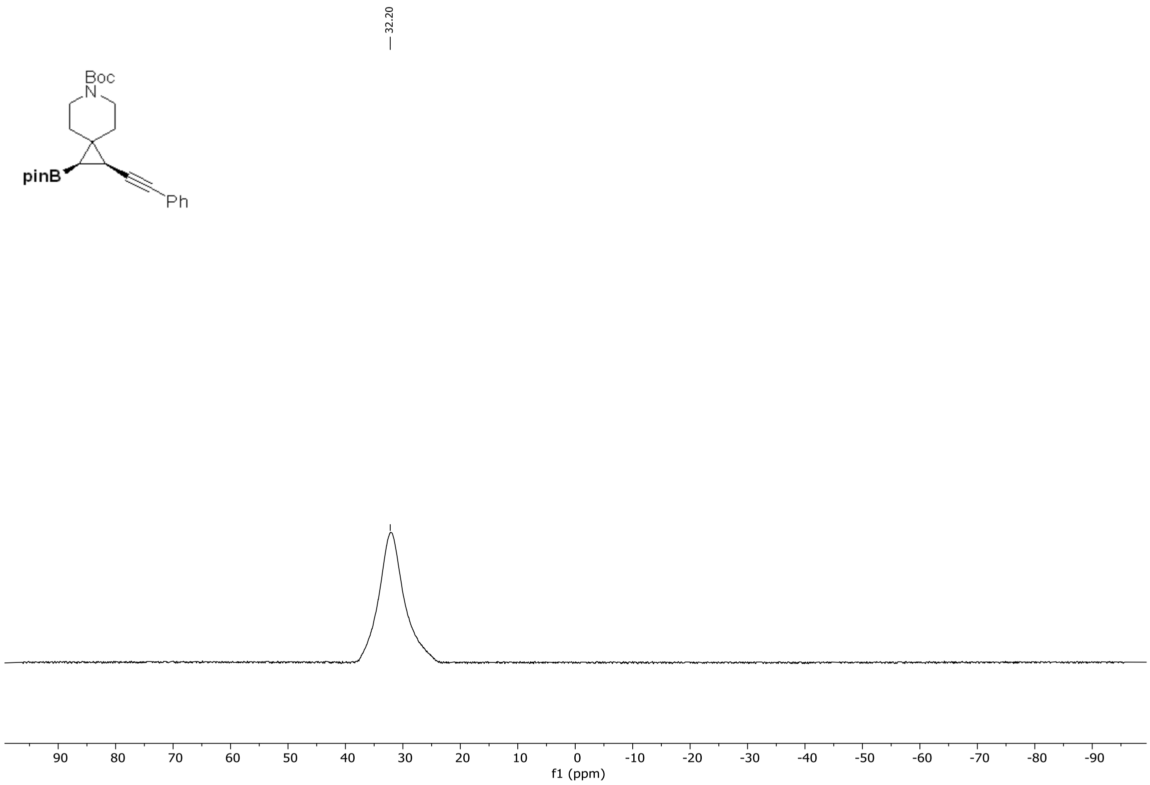

Amination of cyclopropyl bisboronates

Compound 5a

^{1}H NMR (400 MHz, $CDCl_3$)

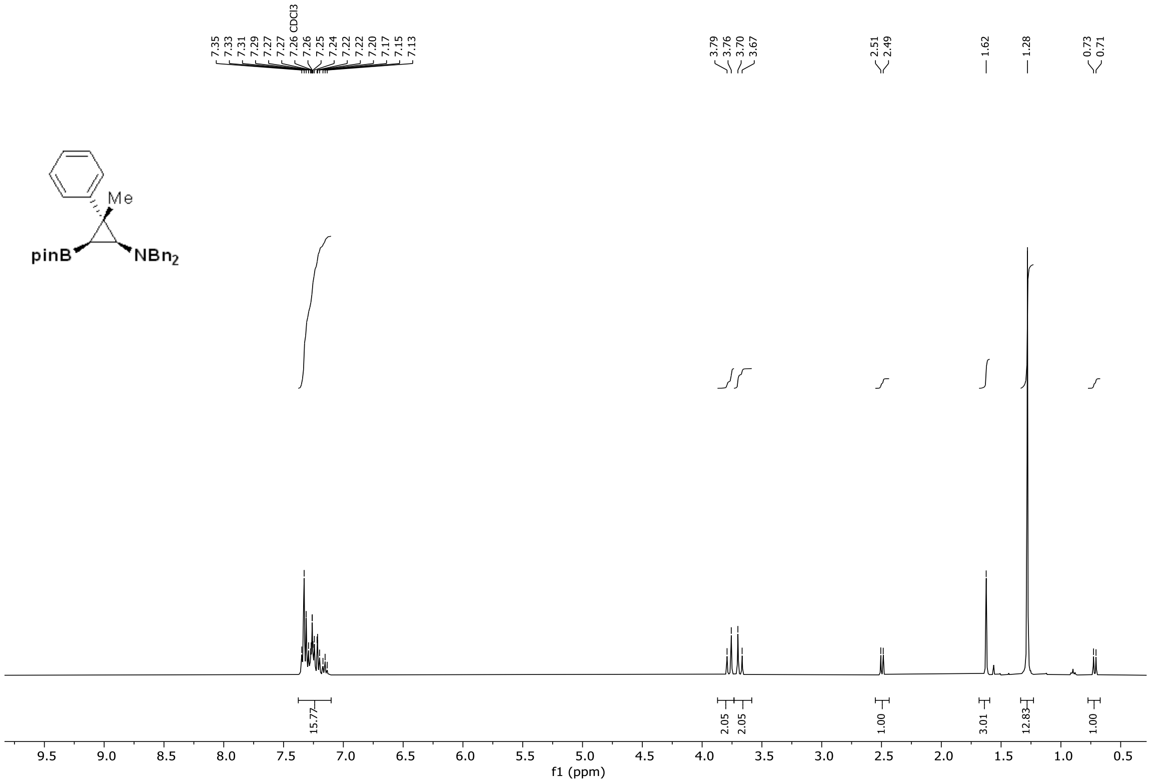

^{13}C NMR (101 MHz, $CDCl_3$)

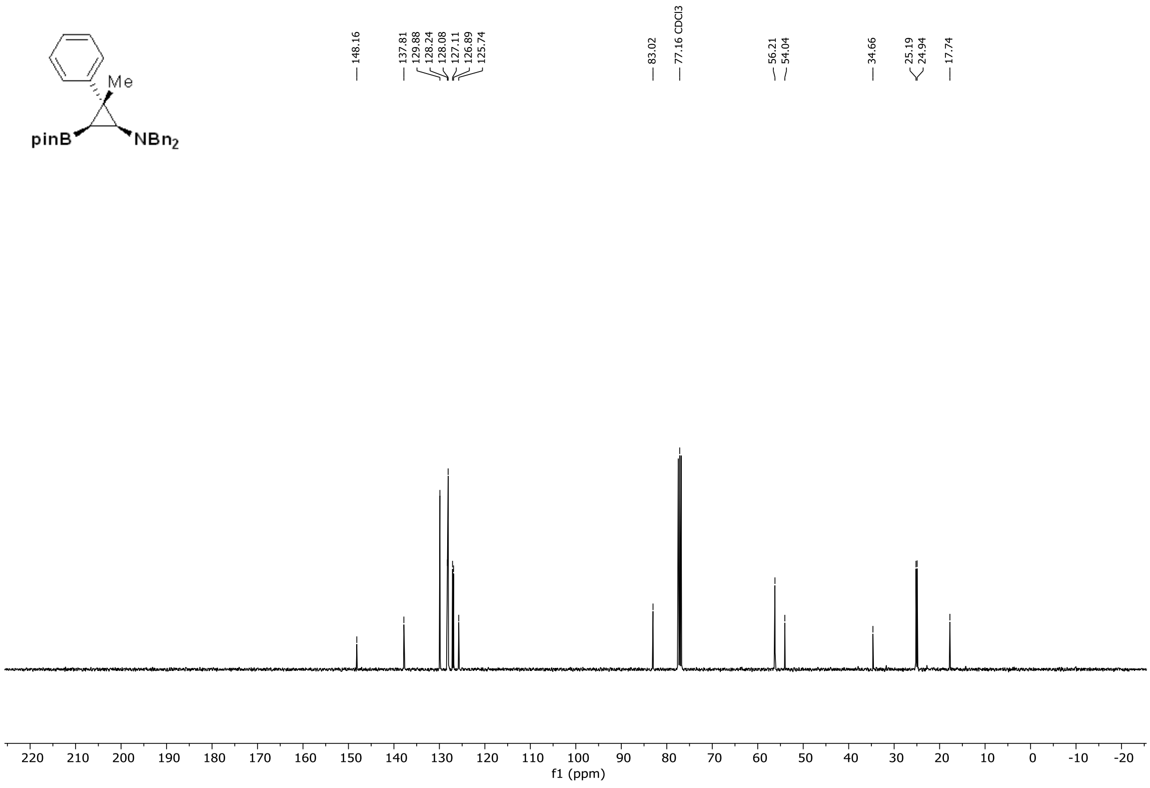

^{11}B NMR (128 MHz, $CDCl_3$)

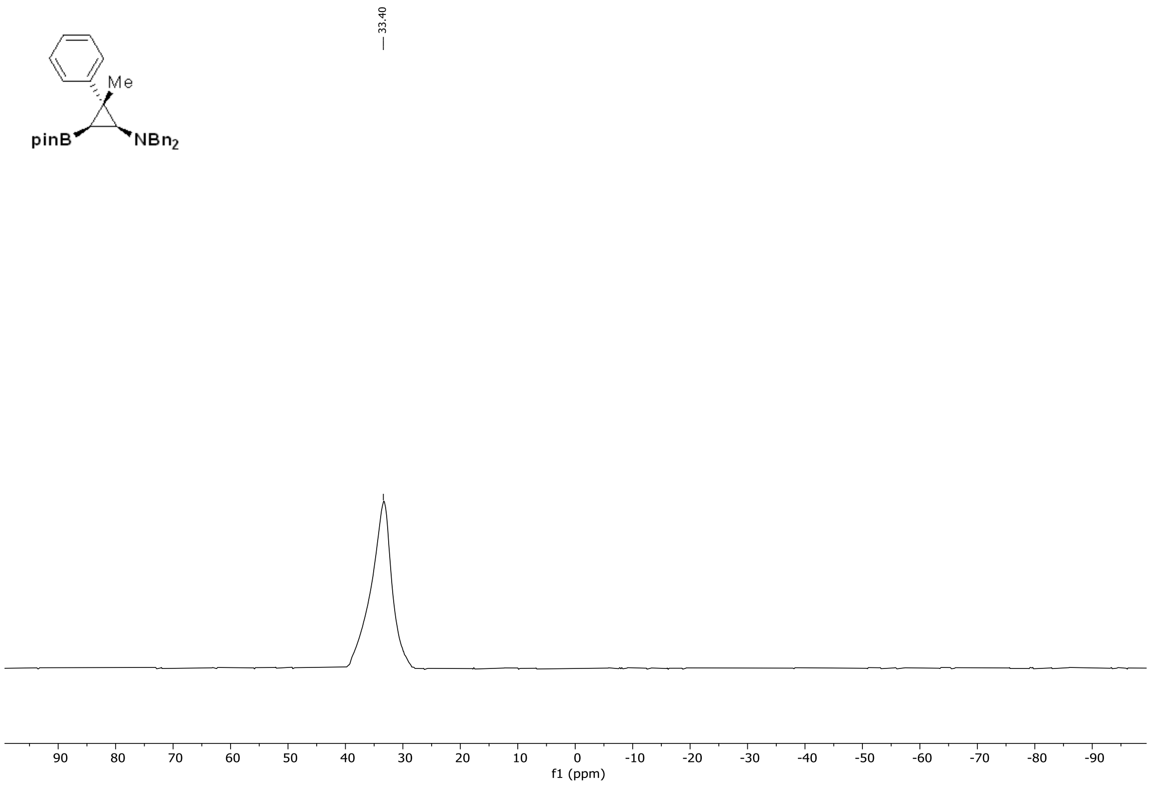

Compound 5b

^{1}H NMR (400 MHz, $CDCl_3$)

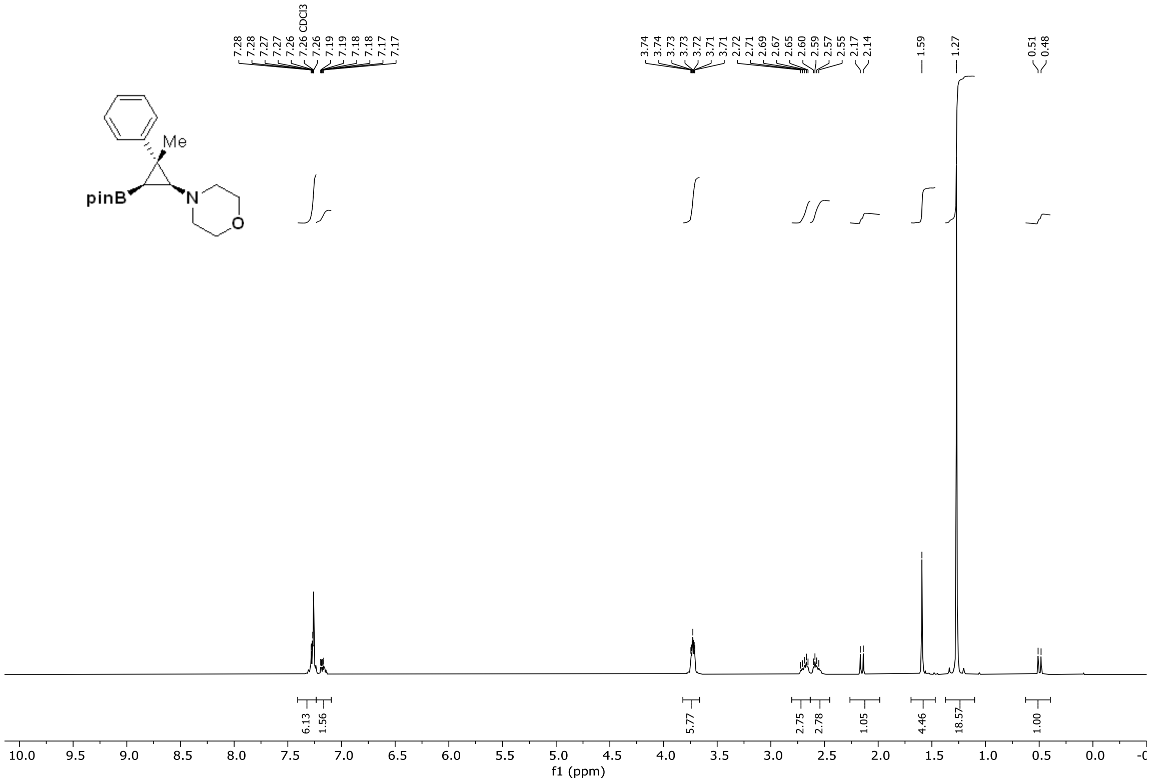

^{13}C NMR (101 MHz, $CDCl_3$)

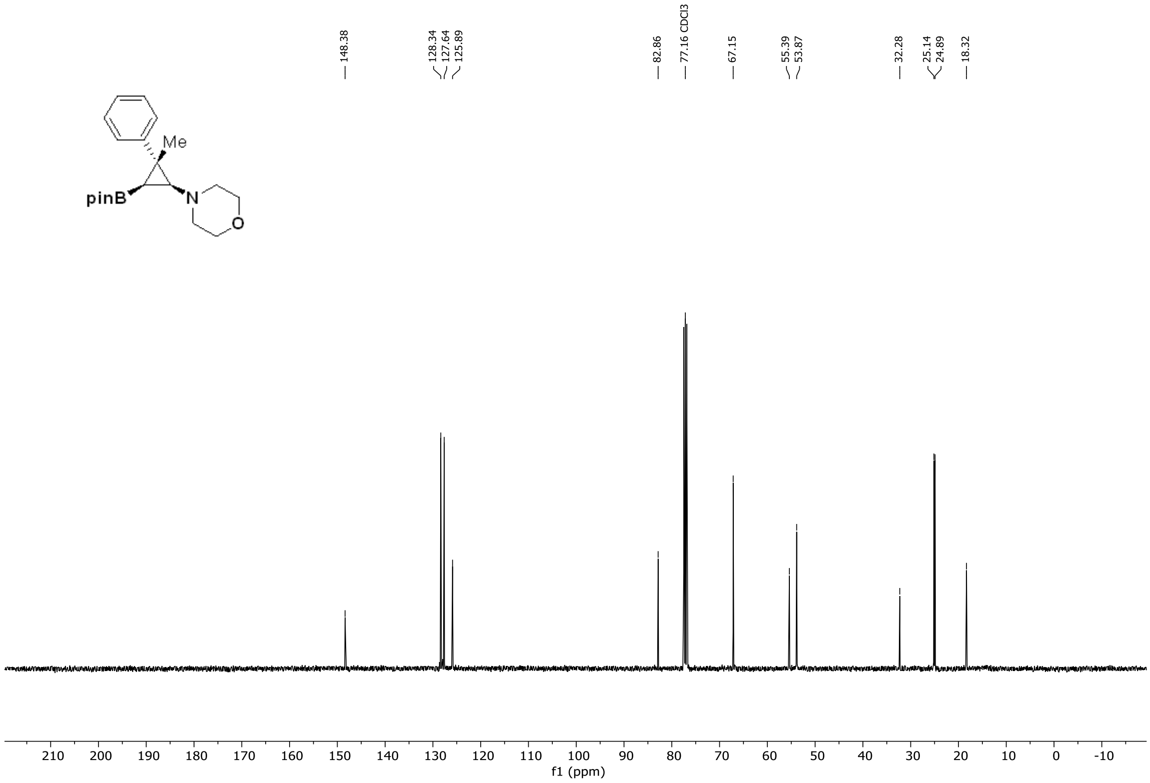

^{11}B NMR (128 MHz, $CDCl_3$)

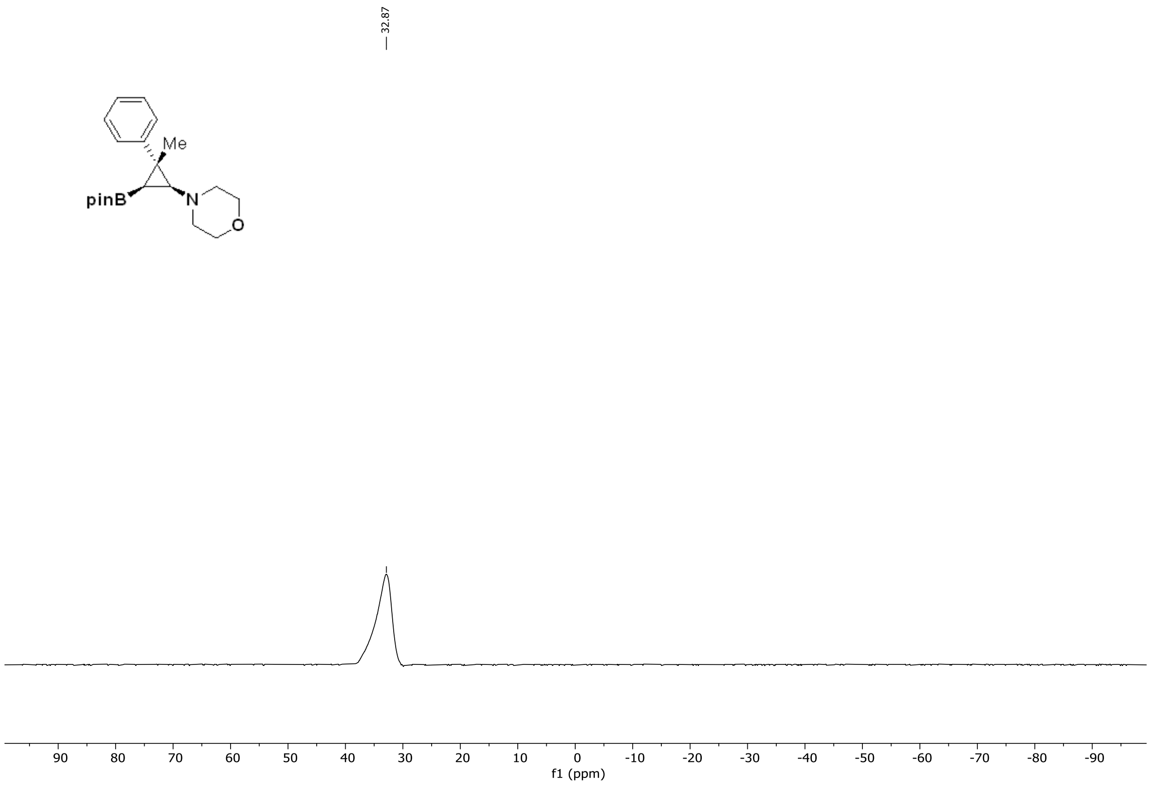

Compound 5c

^{1}H NMR (300 MHz, $CDCl_3$)

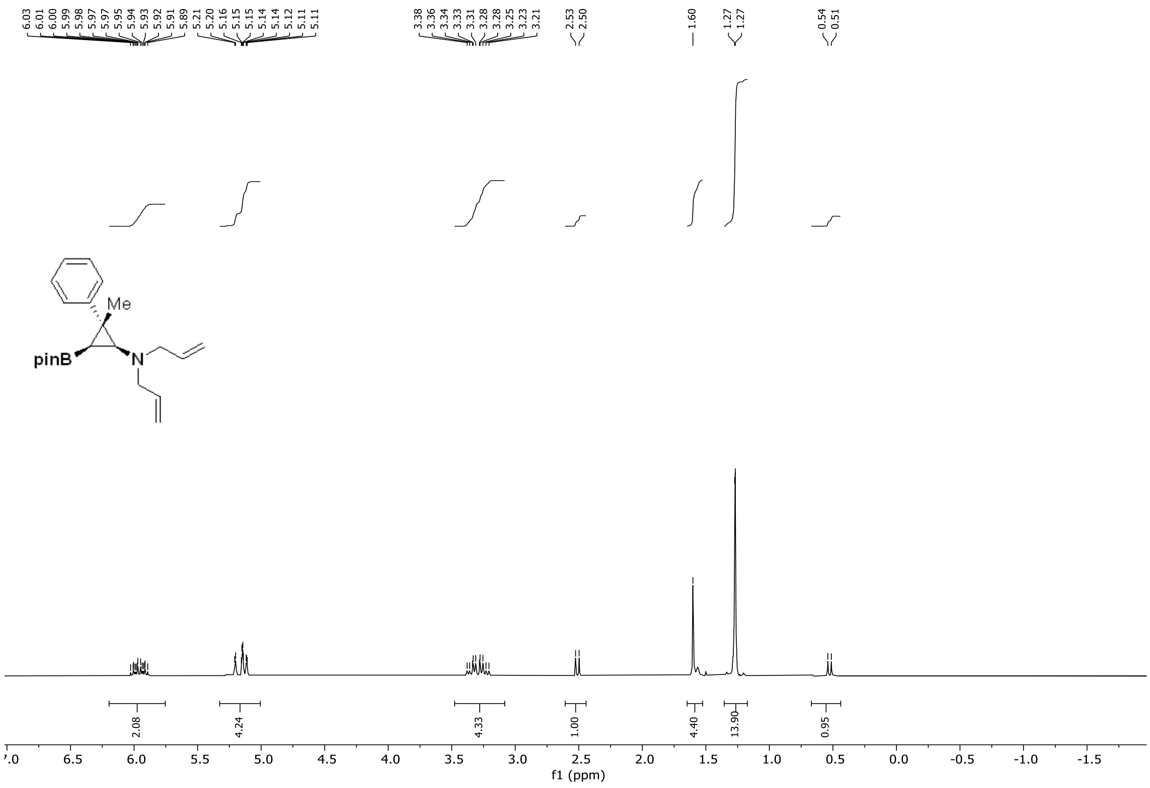

^{13}C NMR (101 MHz, $CDCl_3$)

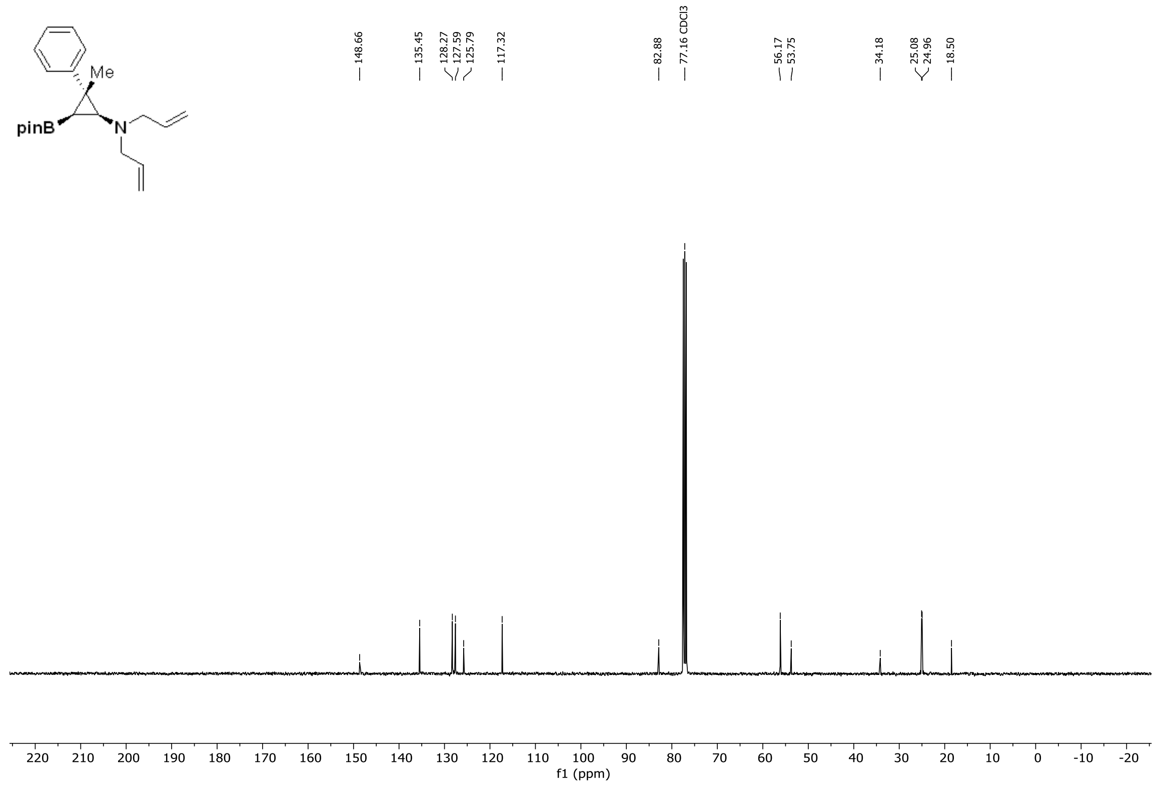

^{11}B NMR (128 MHz, $CDCl_3$)

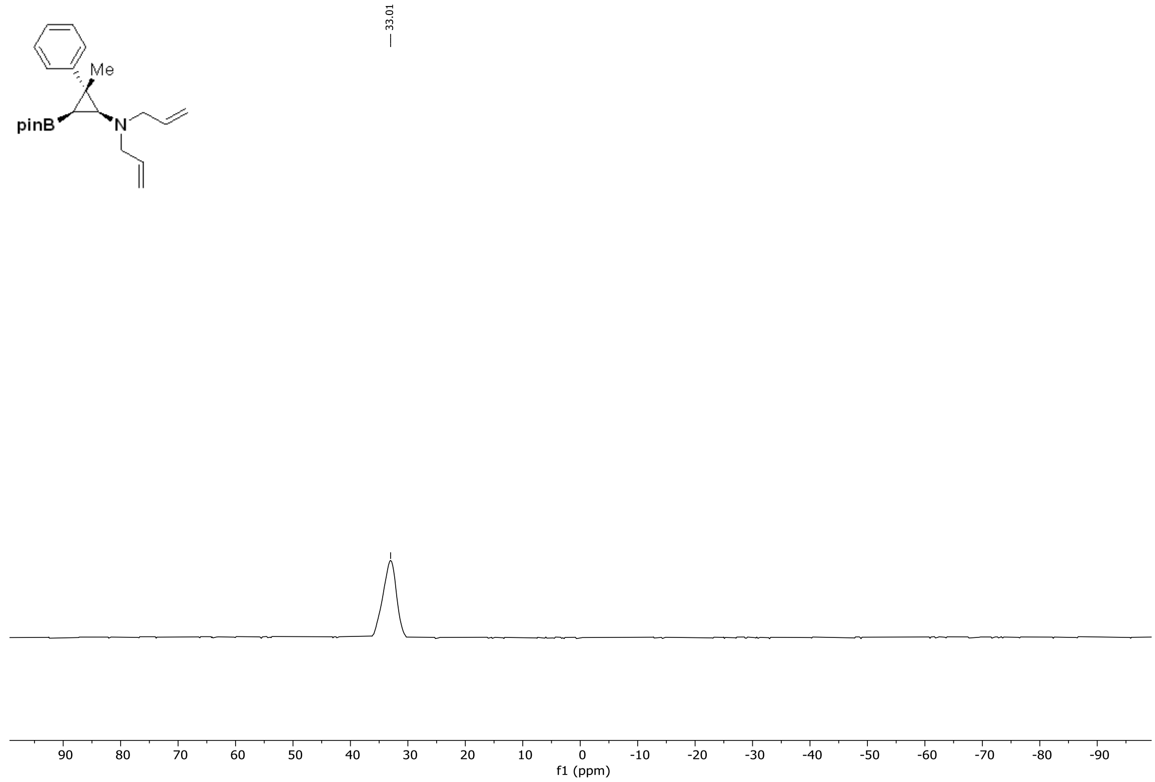

Compound 5d

^{1}H NMR (400 MHz, $CDCl_3$)

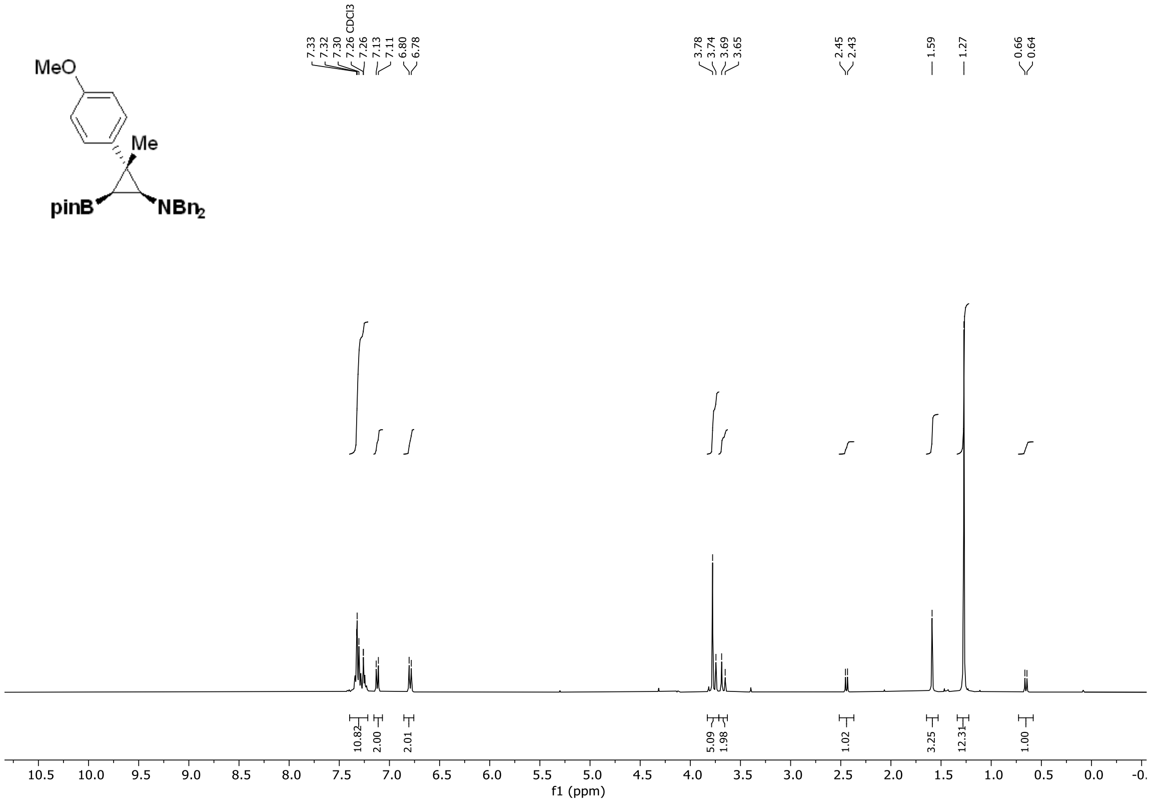

^{13}C NMR (101 MHz, $CDCl_3$)

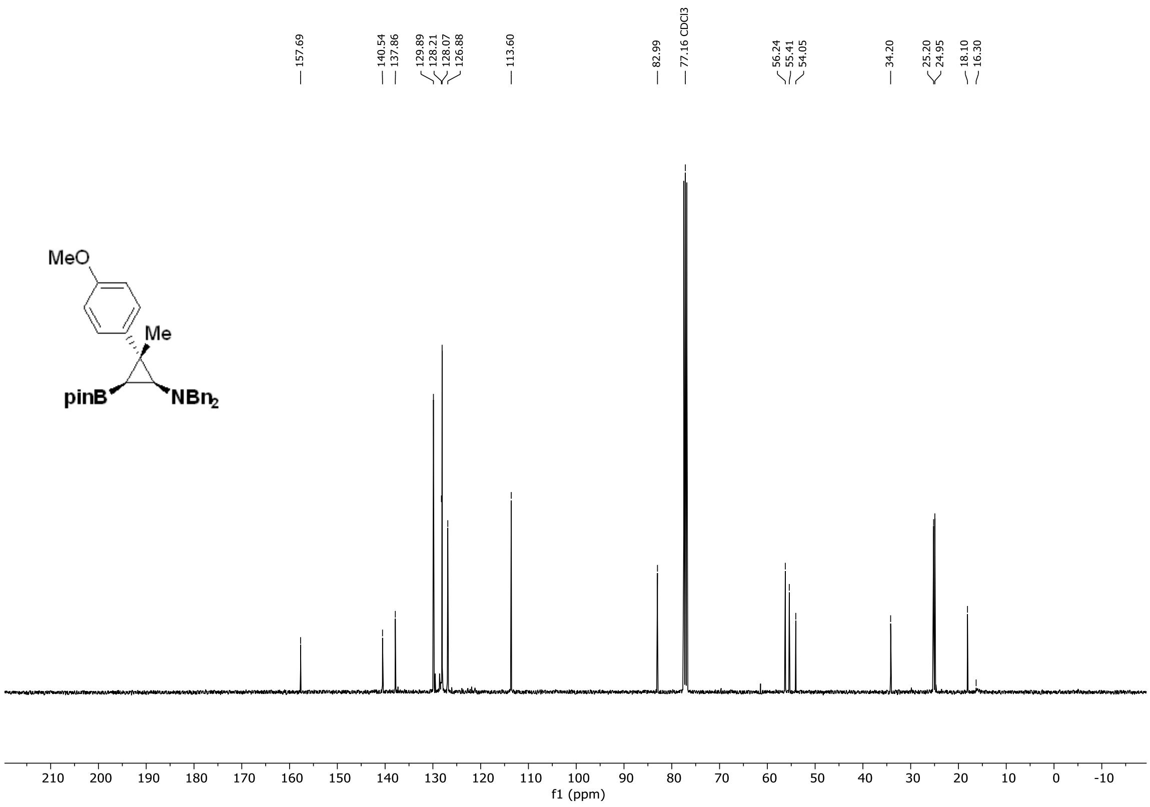

^{11}B NMR (128 MHz, $CDCl_3$)

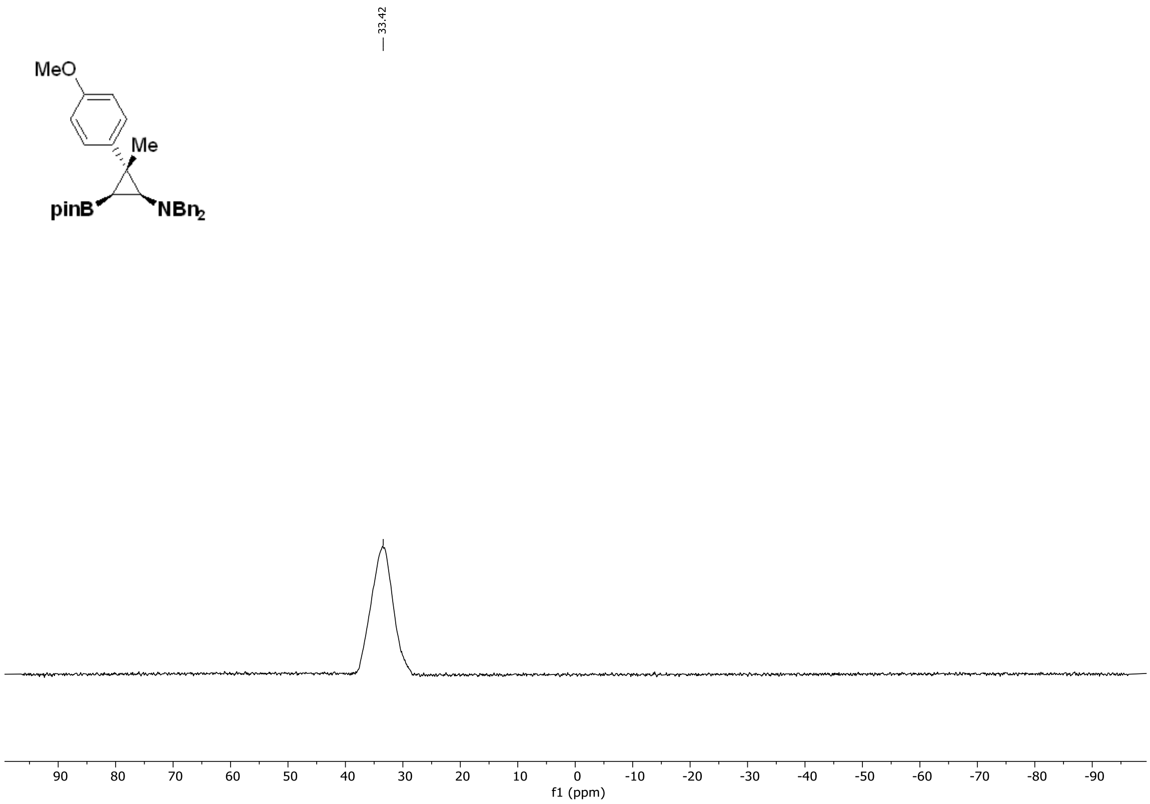

Compound 5e

^{1}H NMR (400 MHz, $CDCl_3$)

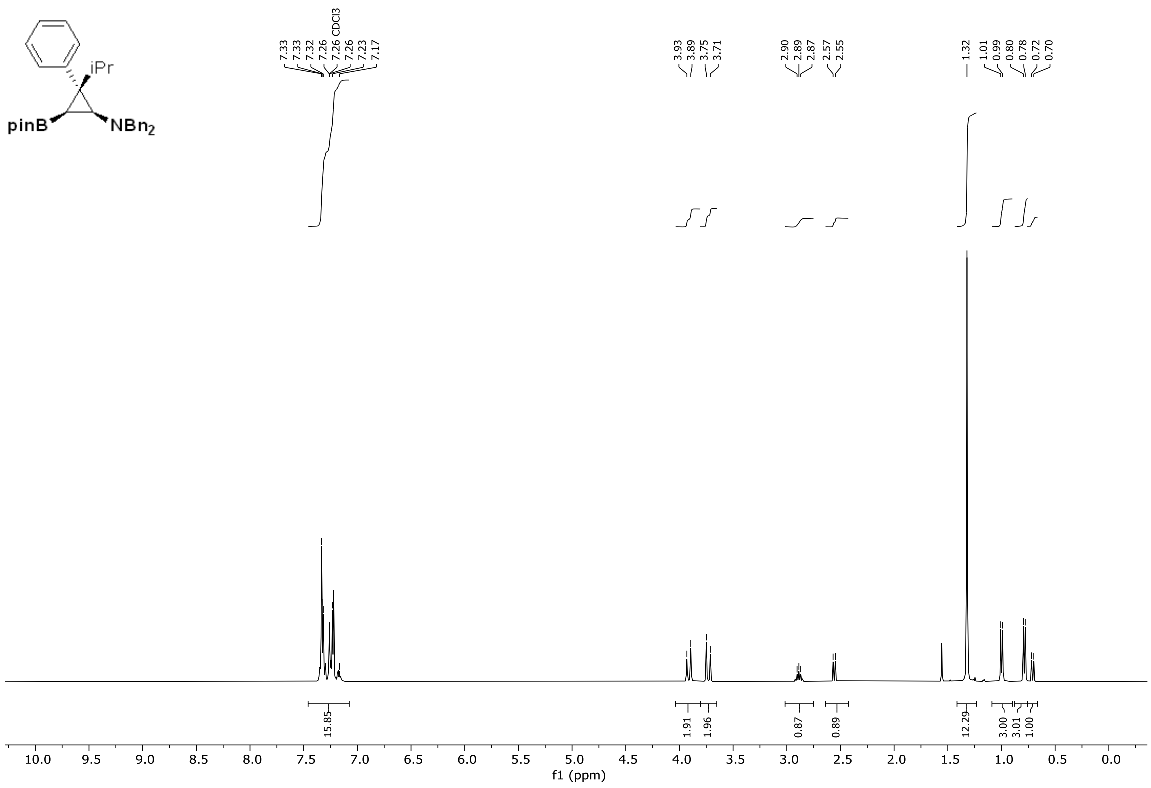

^{13}C NMR (101 MHz, $CDCl_3$)

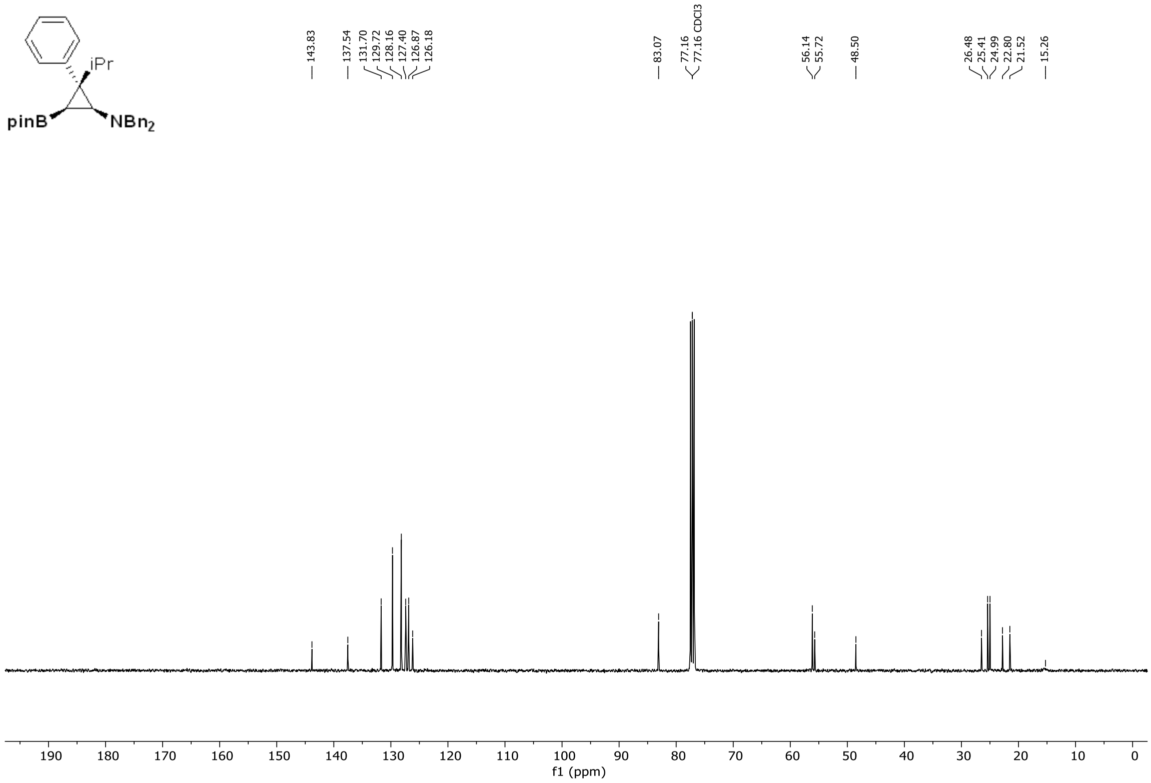

^{11}B NMR (128 MHz, $CDCl_3$)

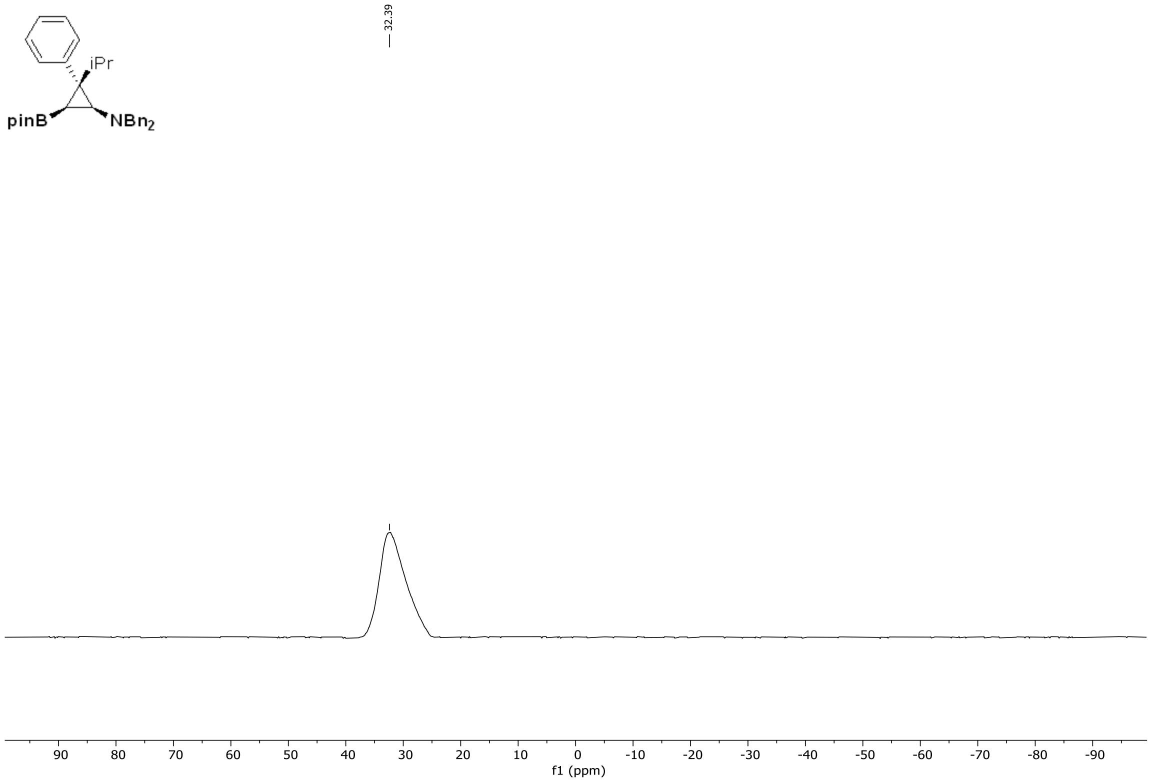

Compound 5f

^{1}H NMR (400 MHz, $CDCl_3$)

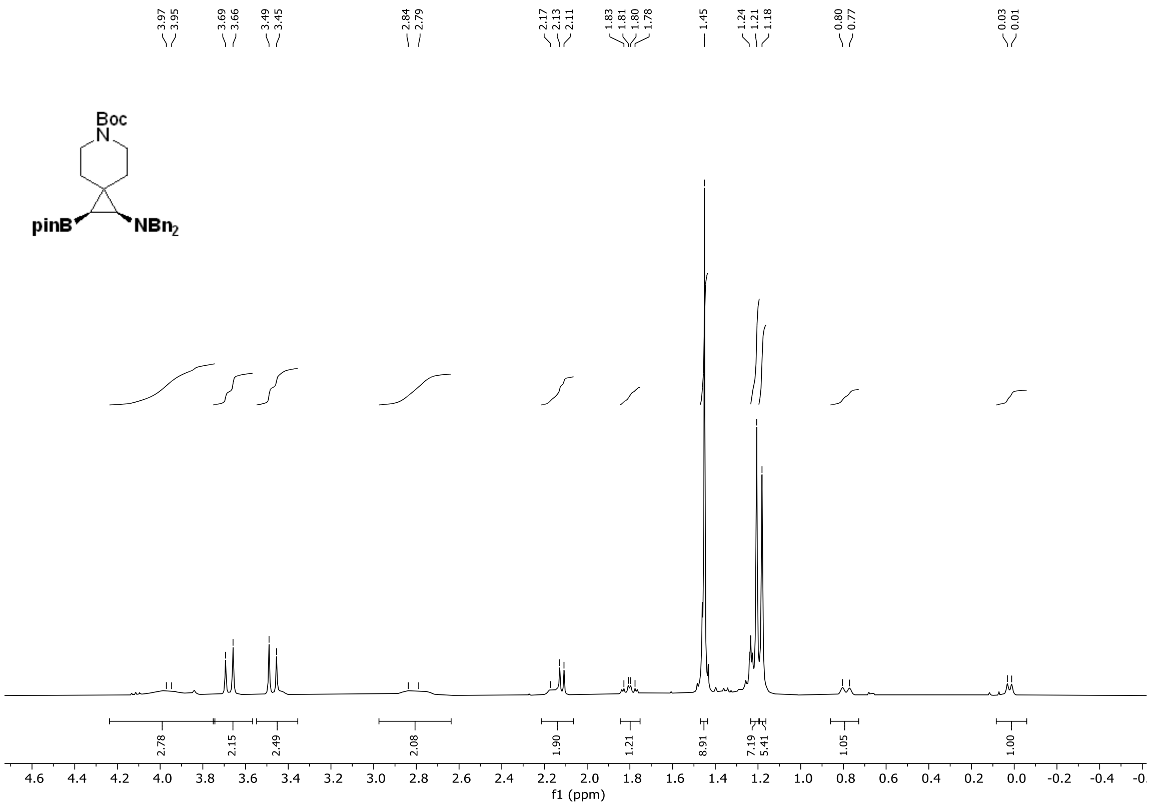

^{13}C NMR (101 MHz, $CDCl_3$)

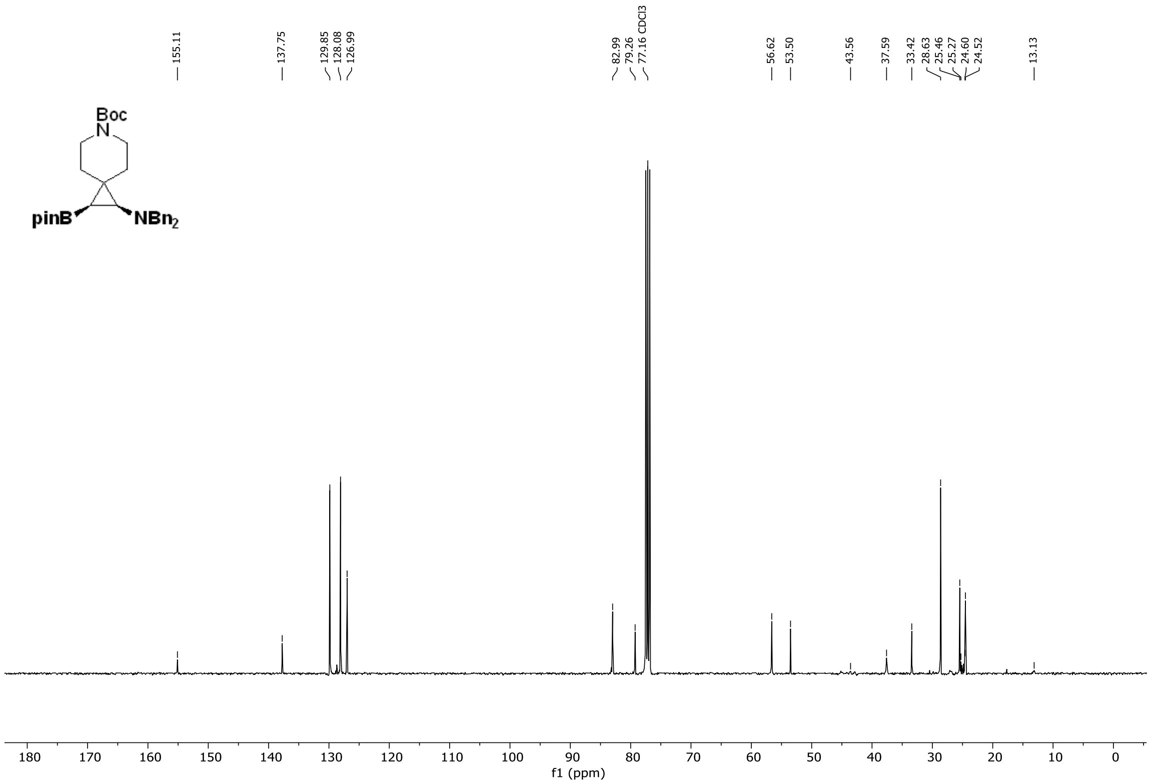

^{11}B NMR (128 MHz, $CDCl_3$)

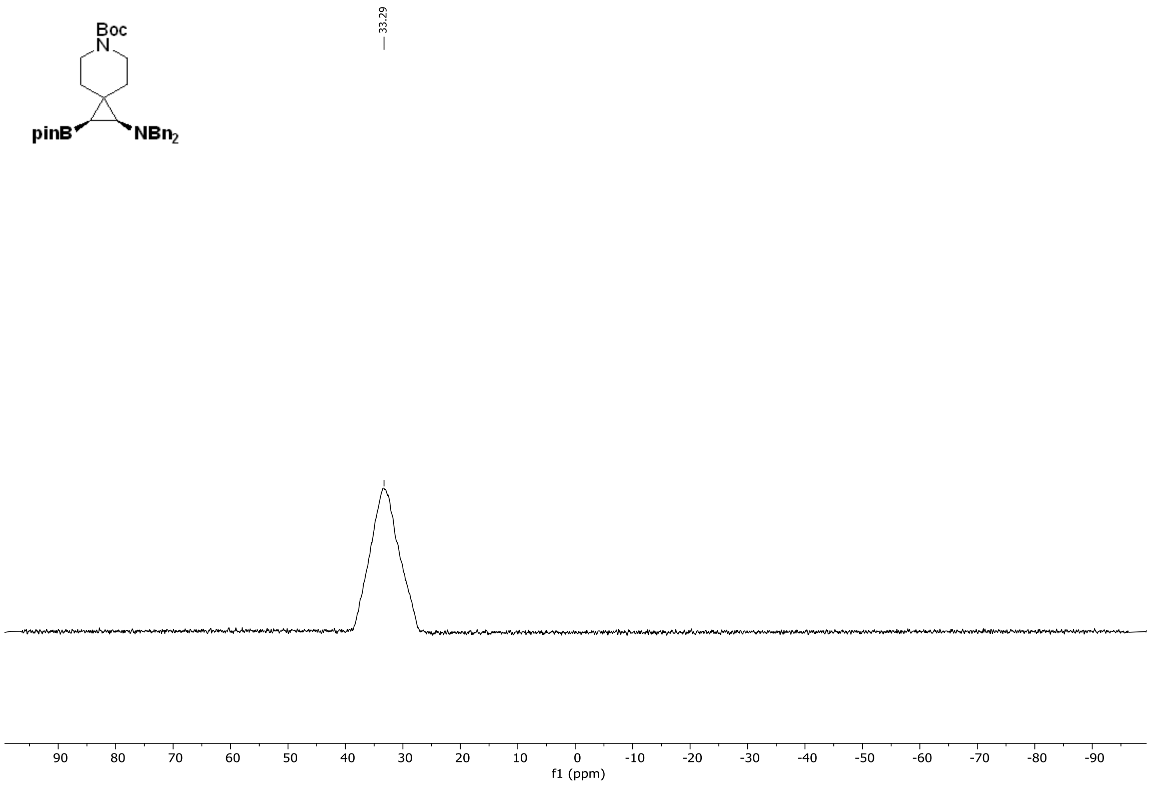

$^1H/^{13}C$ HSQC (400 MHz, $CDCl_3$)

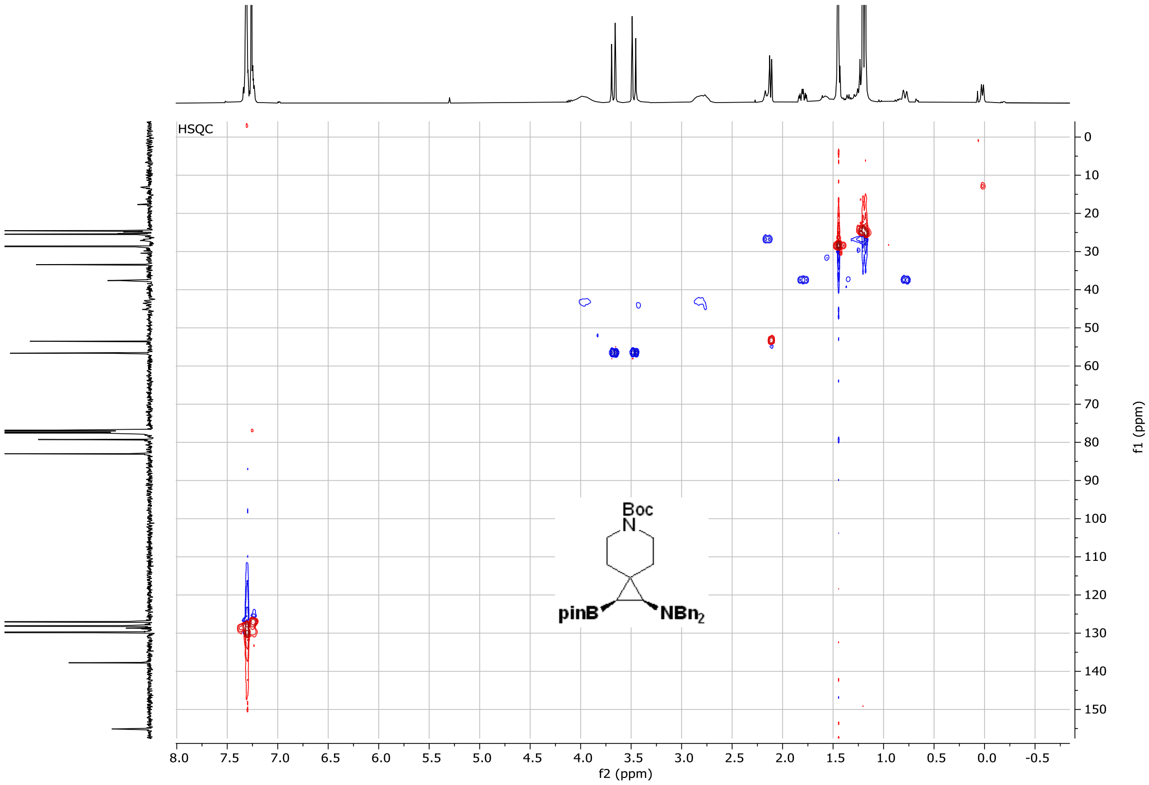

Functionalization of spirocyclobutyl bisboronates

Compound 7a

^{1}H NMR (400 MHz, $CDCl_3$)

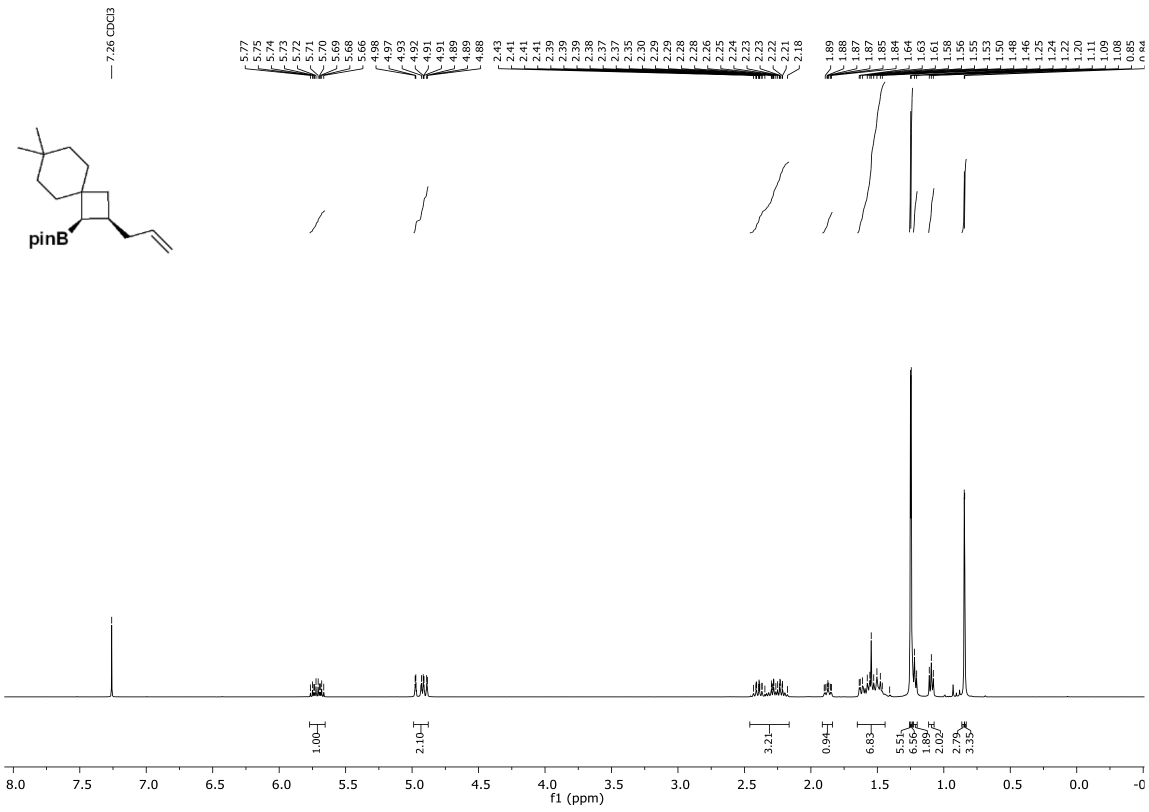

^{13}C NMR (101 MHz, $CDCl_3$)

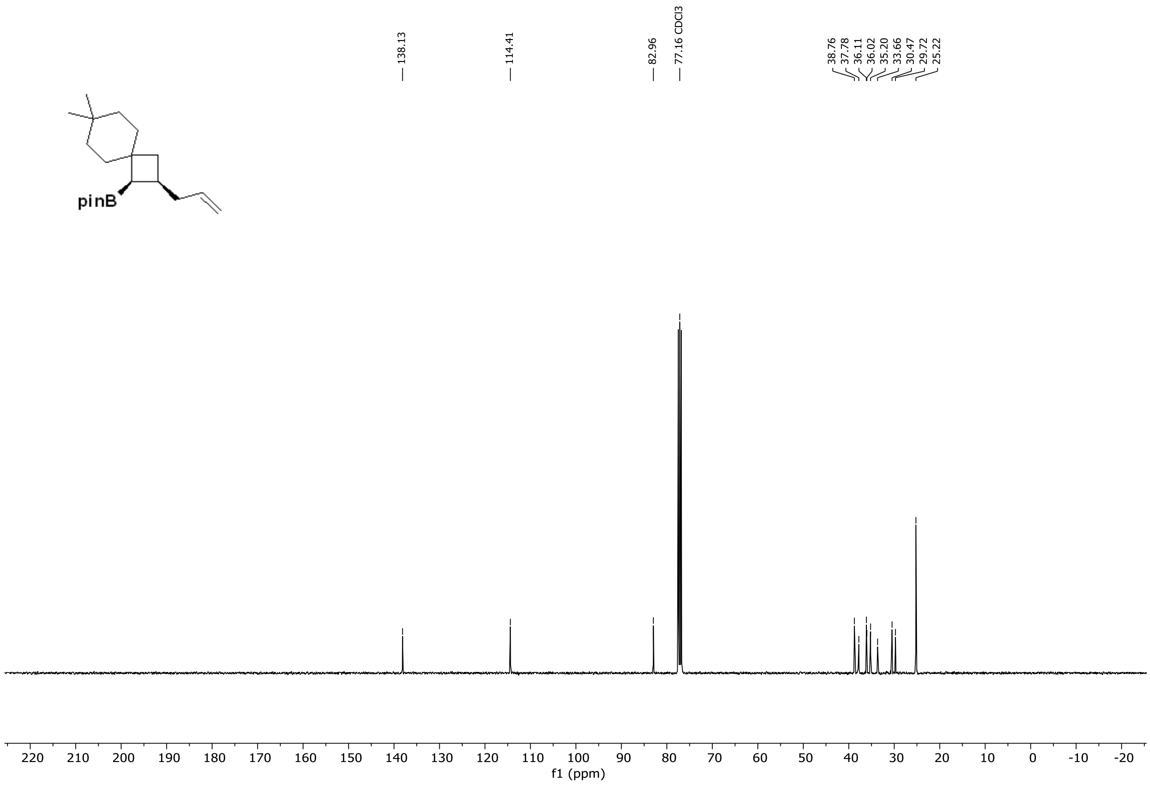

^{11}B NMR (128 MHz, $CDCl_3$)

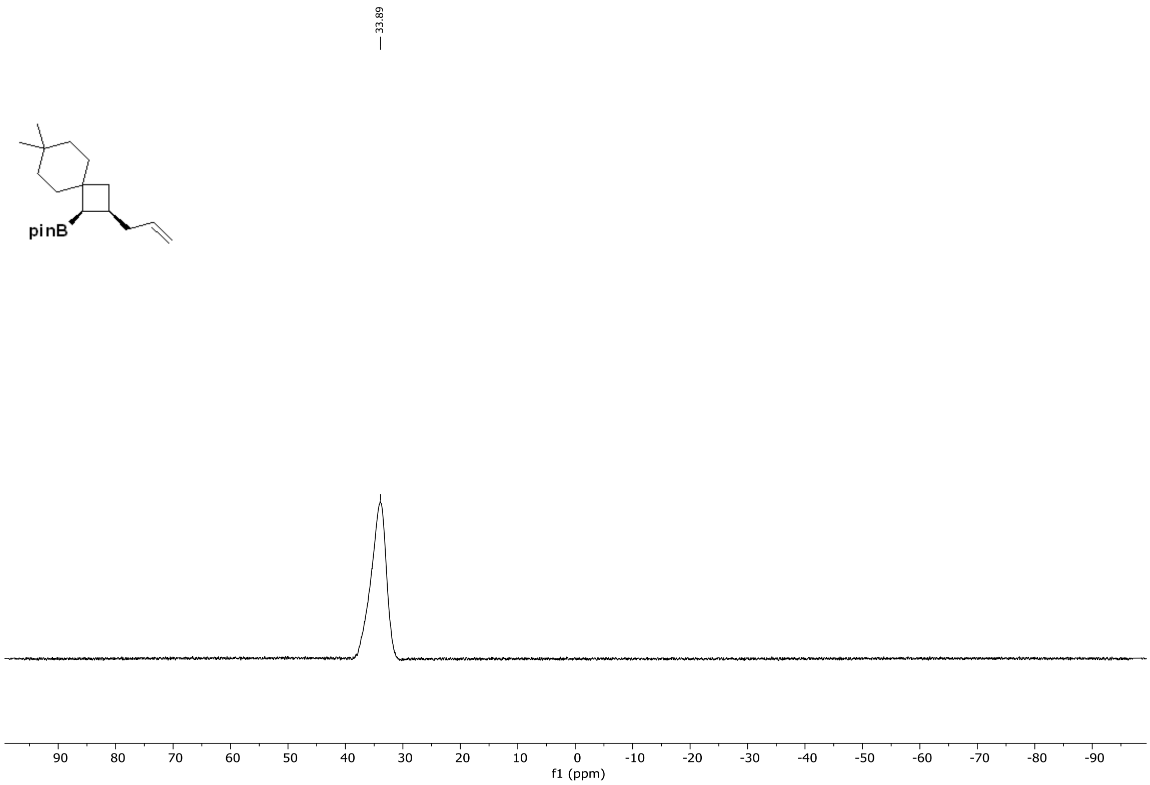

HSQC (400 MHz, $CDCl_3$)

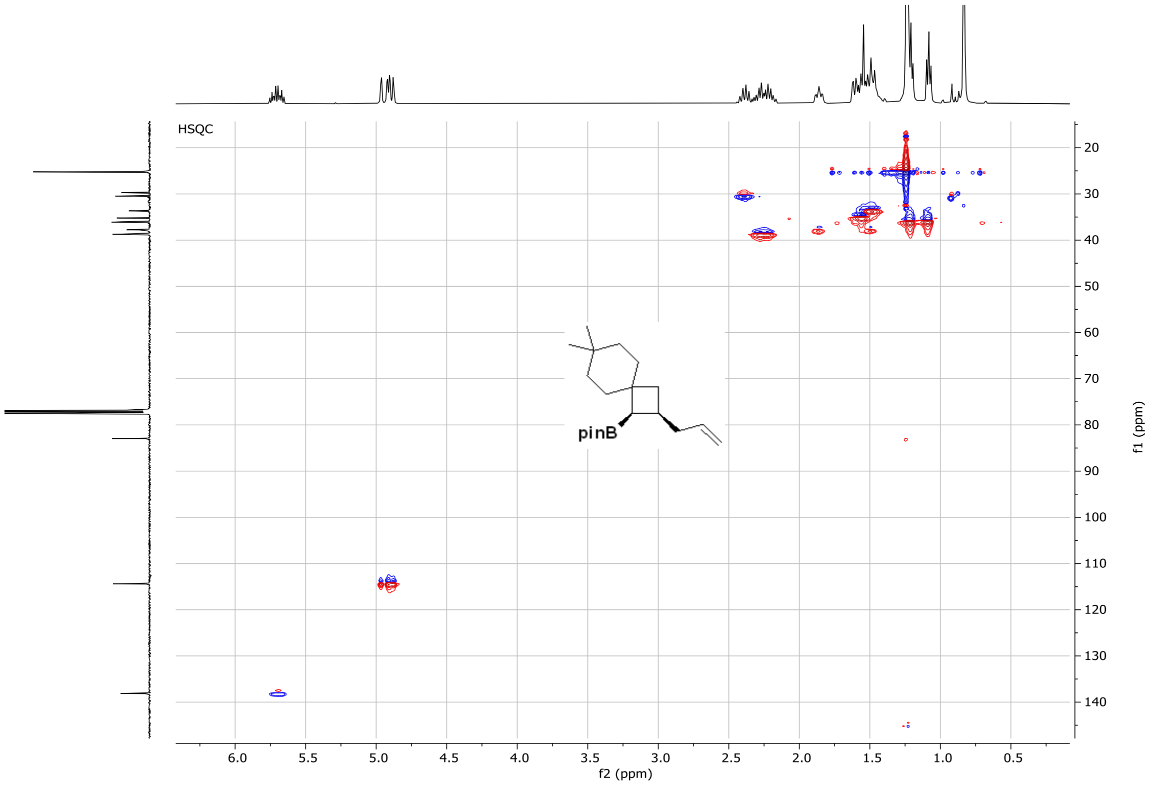

Compound 7b

^{1}H NMR (400 MHz, $CDCl_3$)

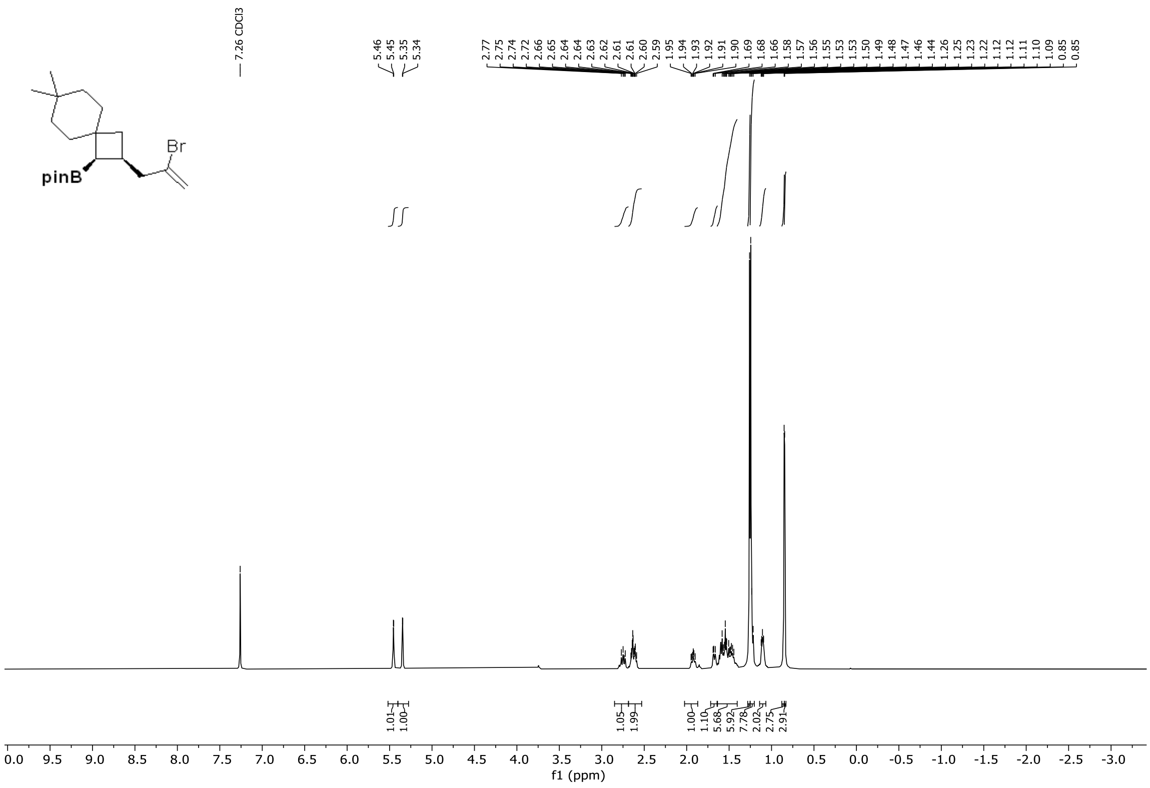

^{13}C NMR (101 MHz, $CDCl_3$)

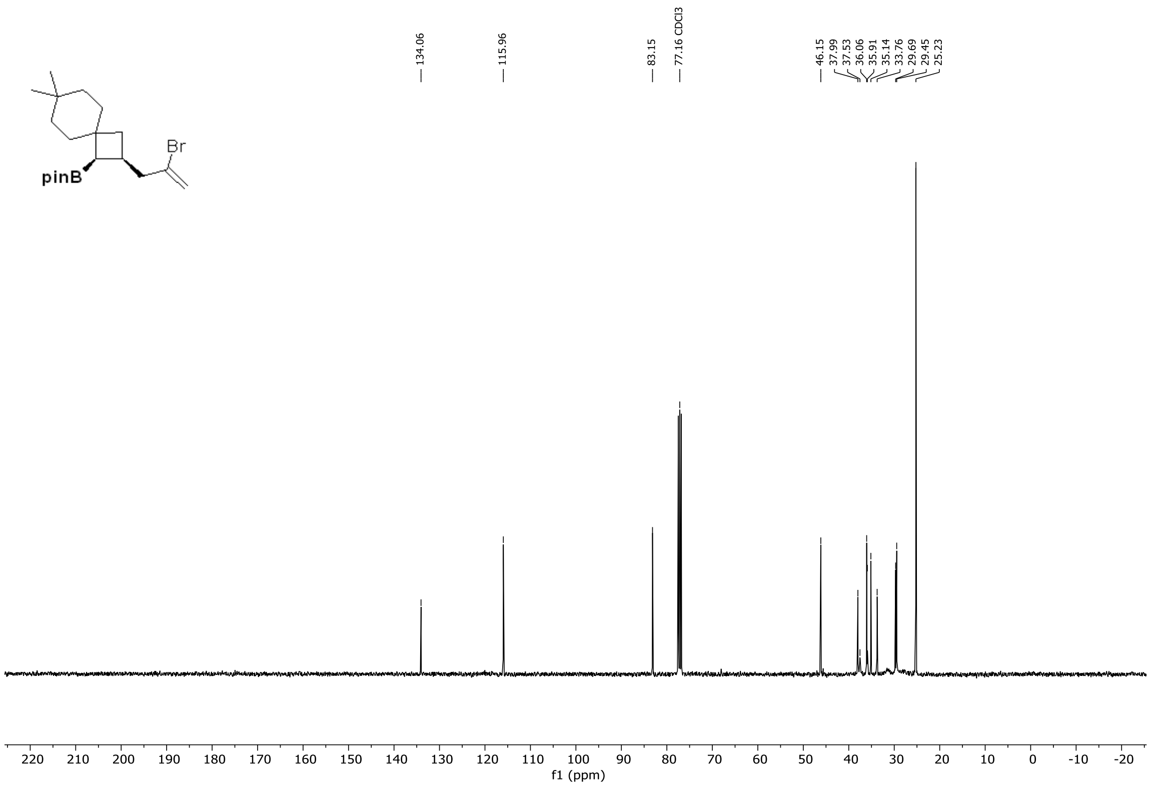

^{11}B NMR (128 MHz, $CDCl_3$)

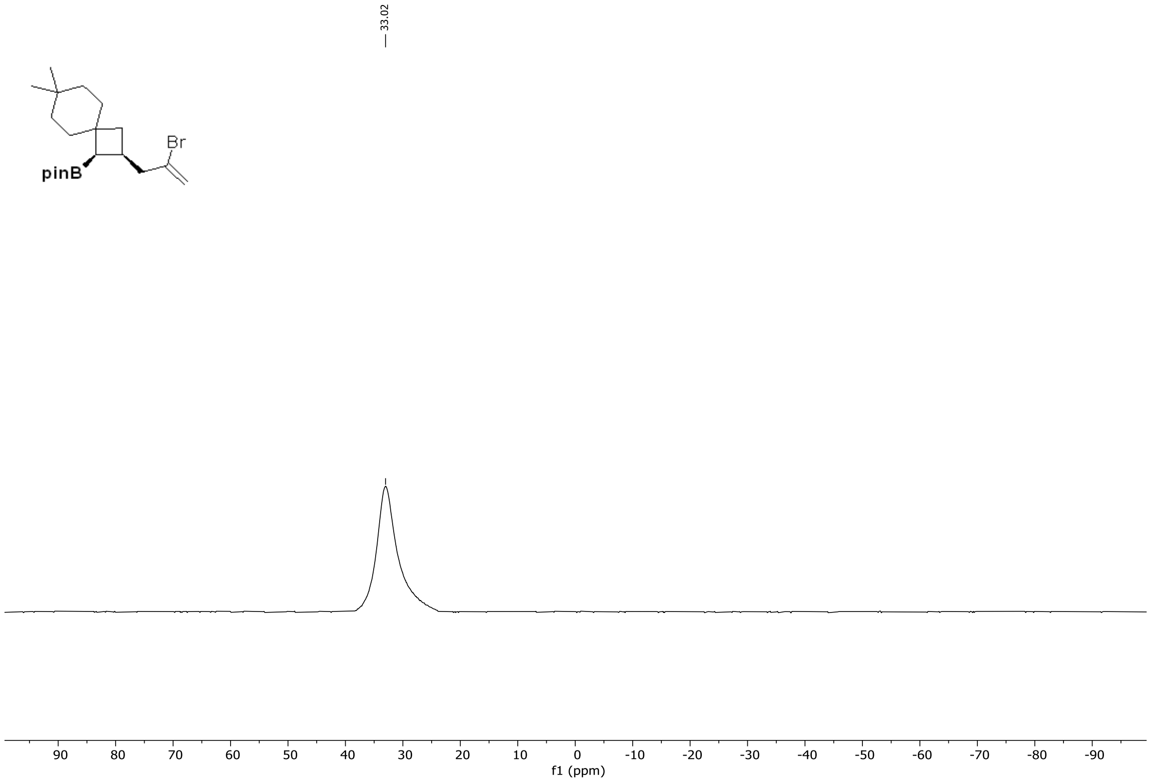

Compound 7c

^{1}H NMR (400 MHz, $CDCl_3$)

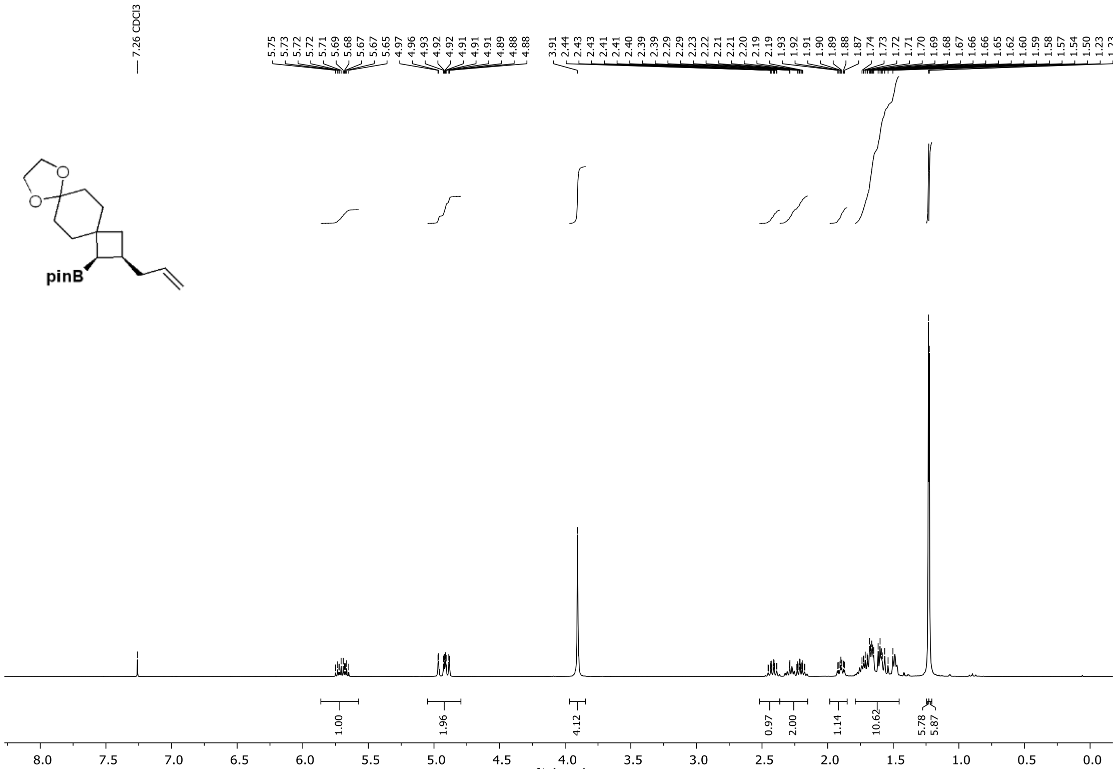

^{13}C NMR (101 MHz, $CDCl_3$)

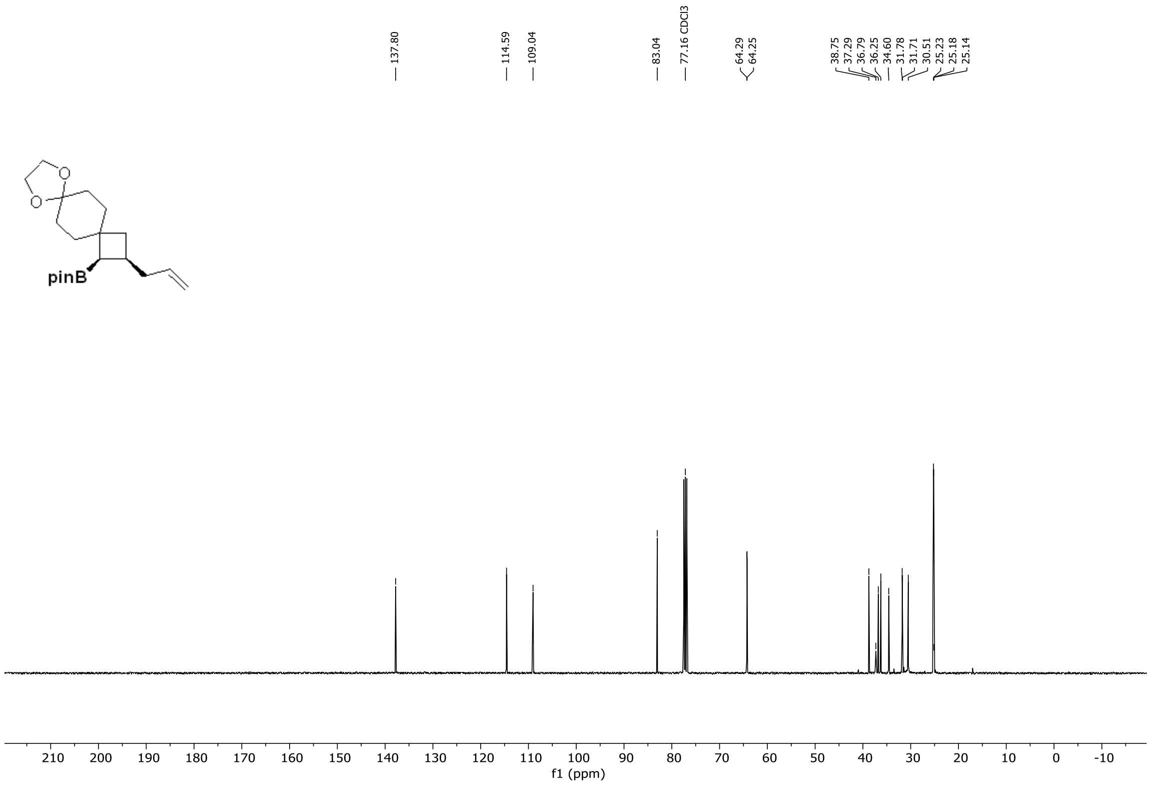

^{11}B NMR (128 MHz, $CDCl_3$)

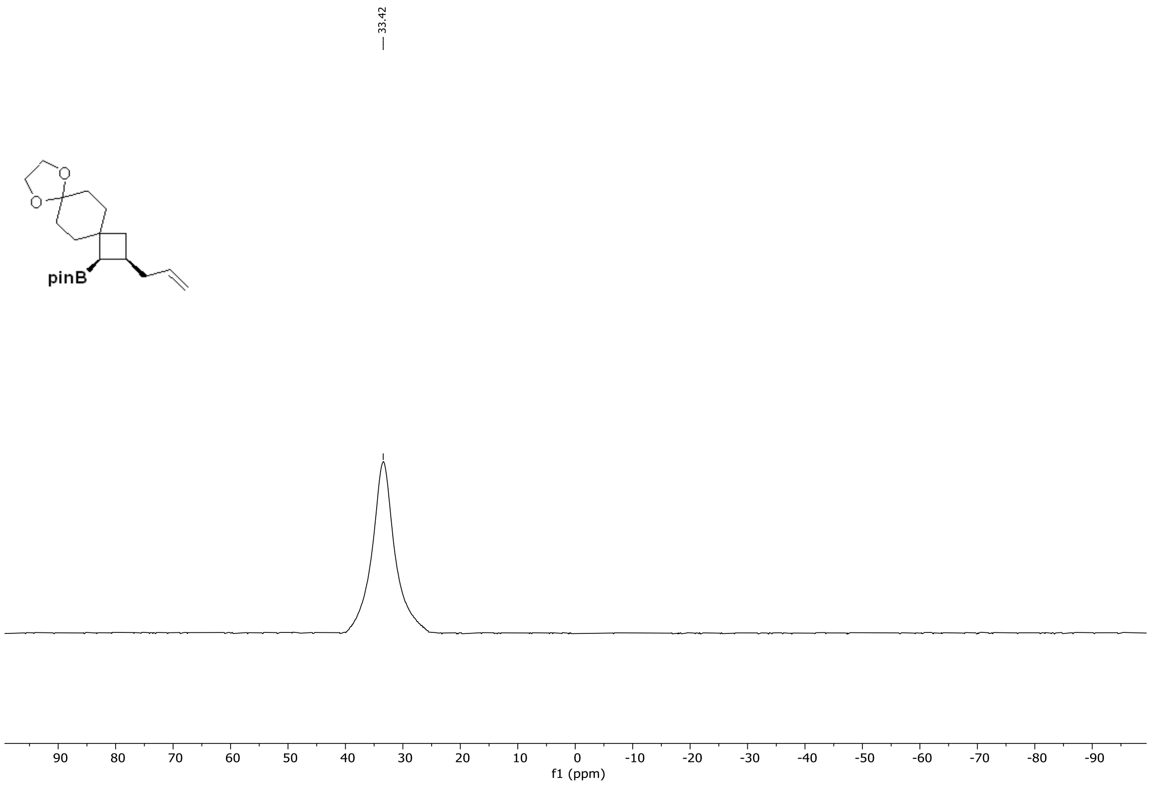

Compound 7d

^{1}H NMR (400 MHz, $CDCl_3$)

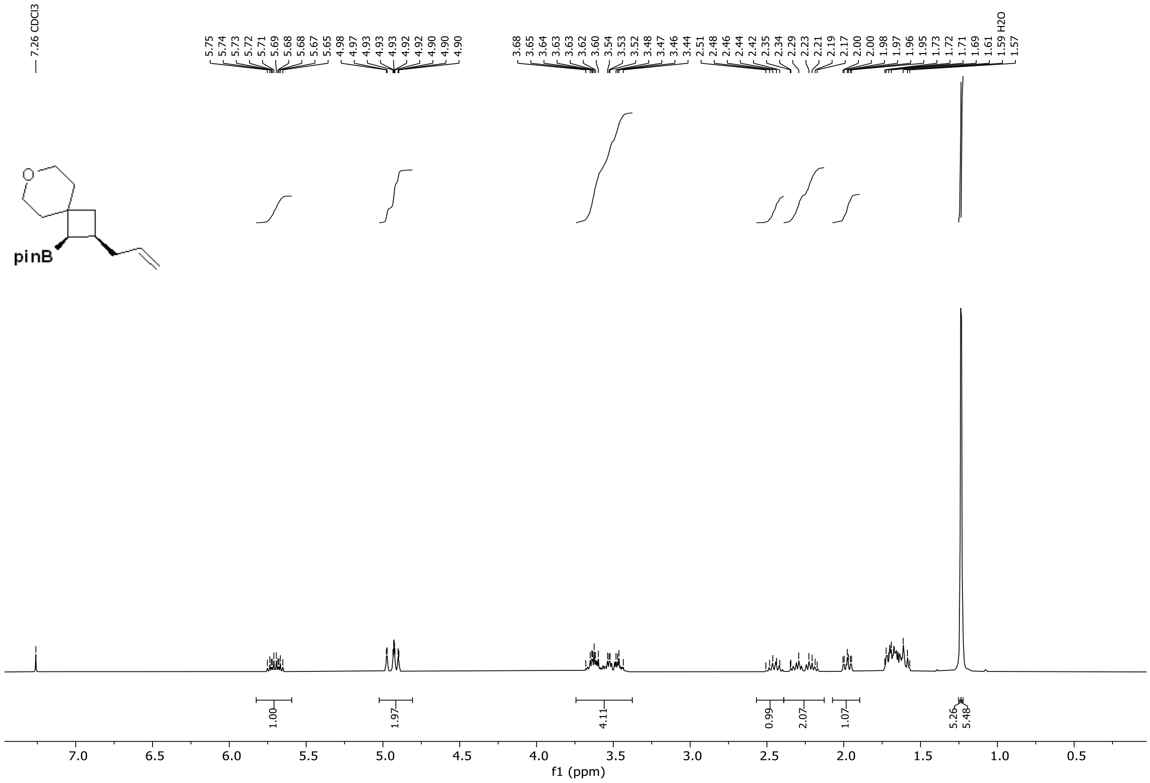

^{13}C NMR (101 MHz, $CDCl_3$)

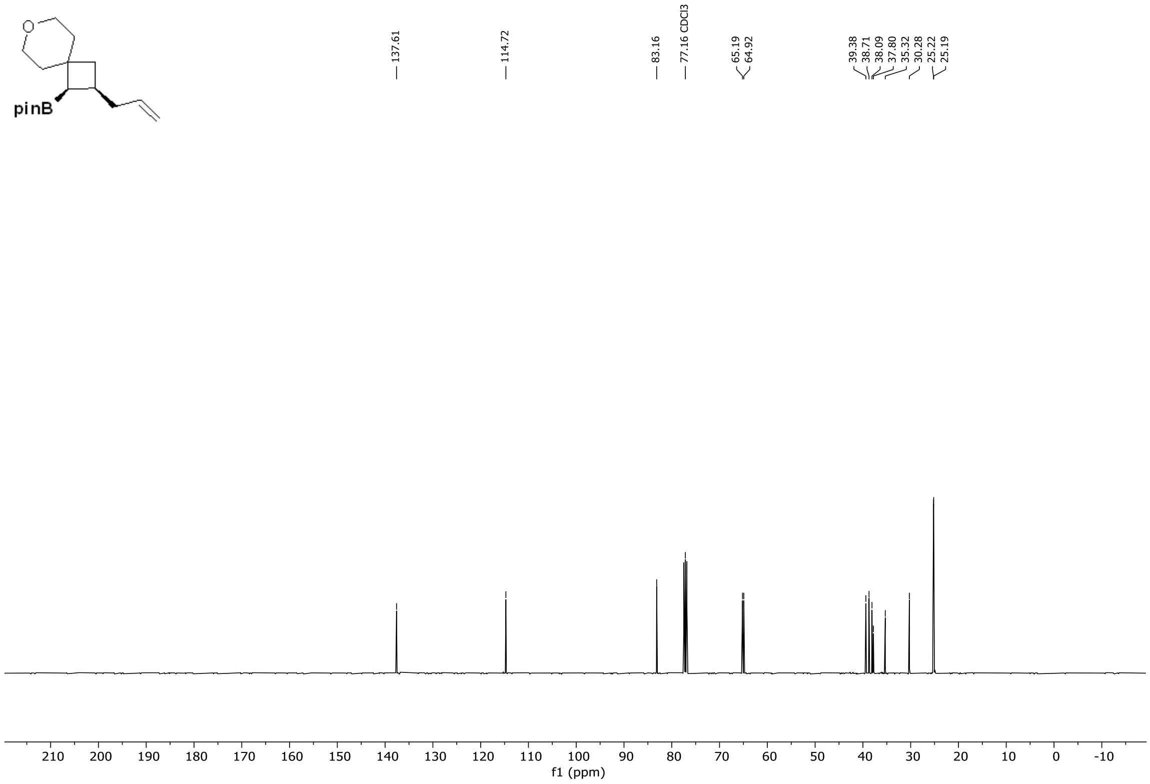

^{11}B NMR (128 MHz, $CDCl_3$)

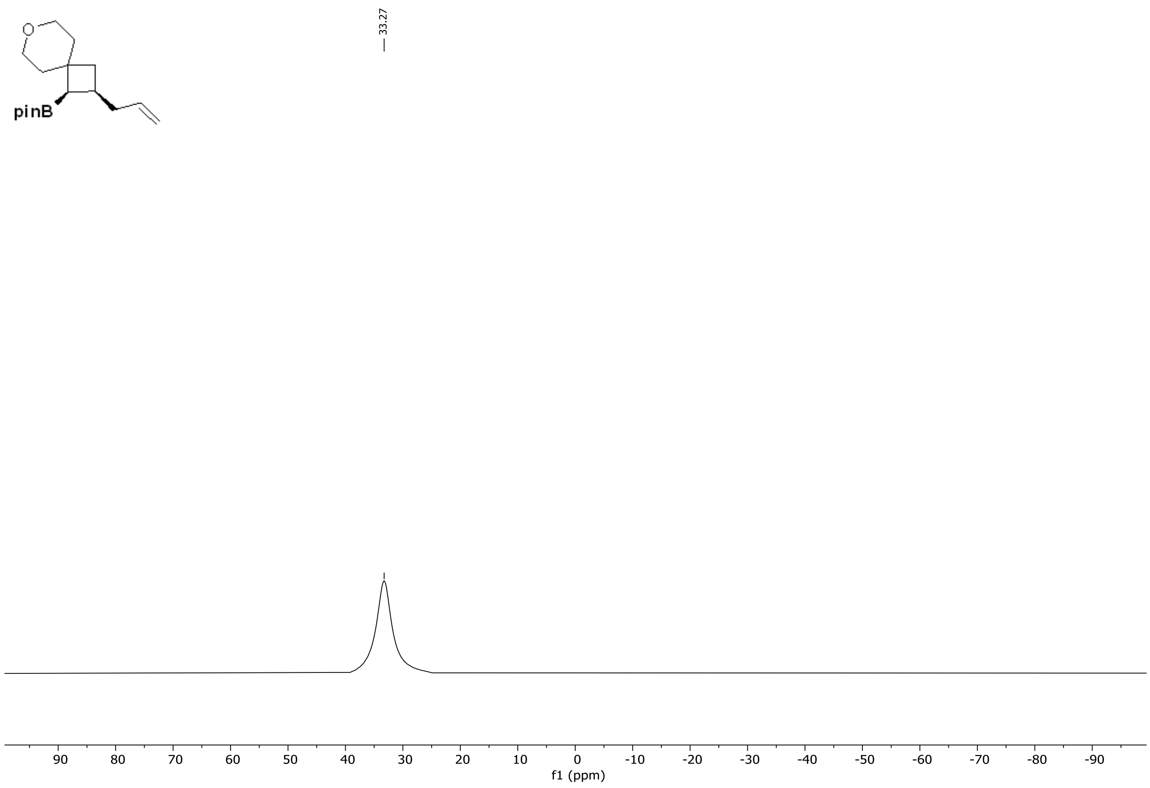

Compound 7e

^{1}H NMR (400 MHz, $CDCl_3$)

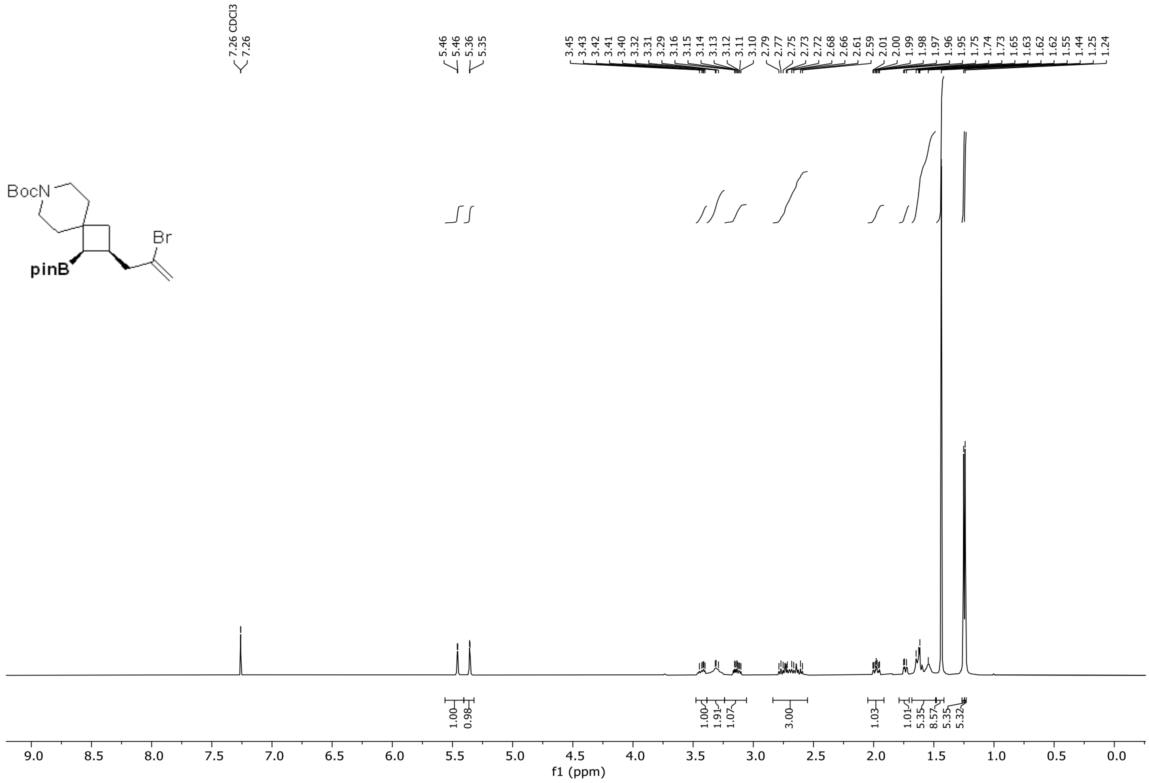

^{13}C NMR (101 MHz, $CDCl_3$)

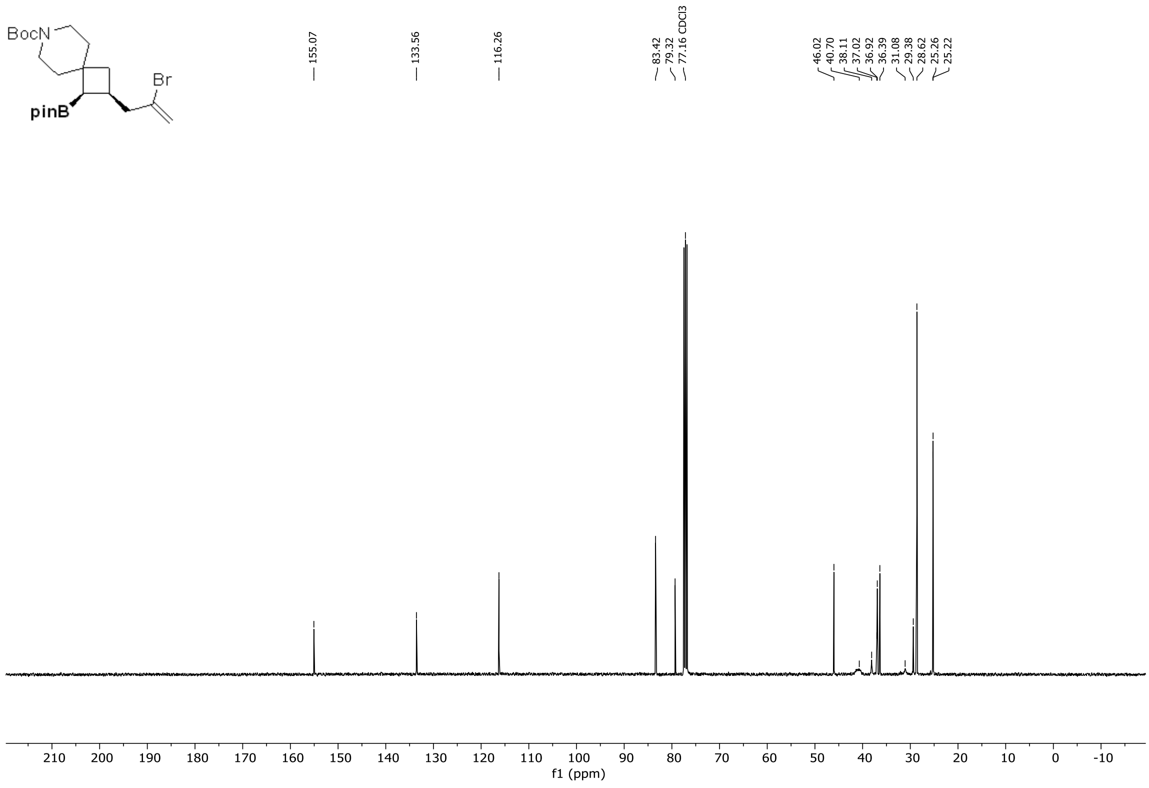

^{11}B NMR (128 MHz, $CDCl_3$)

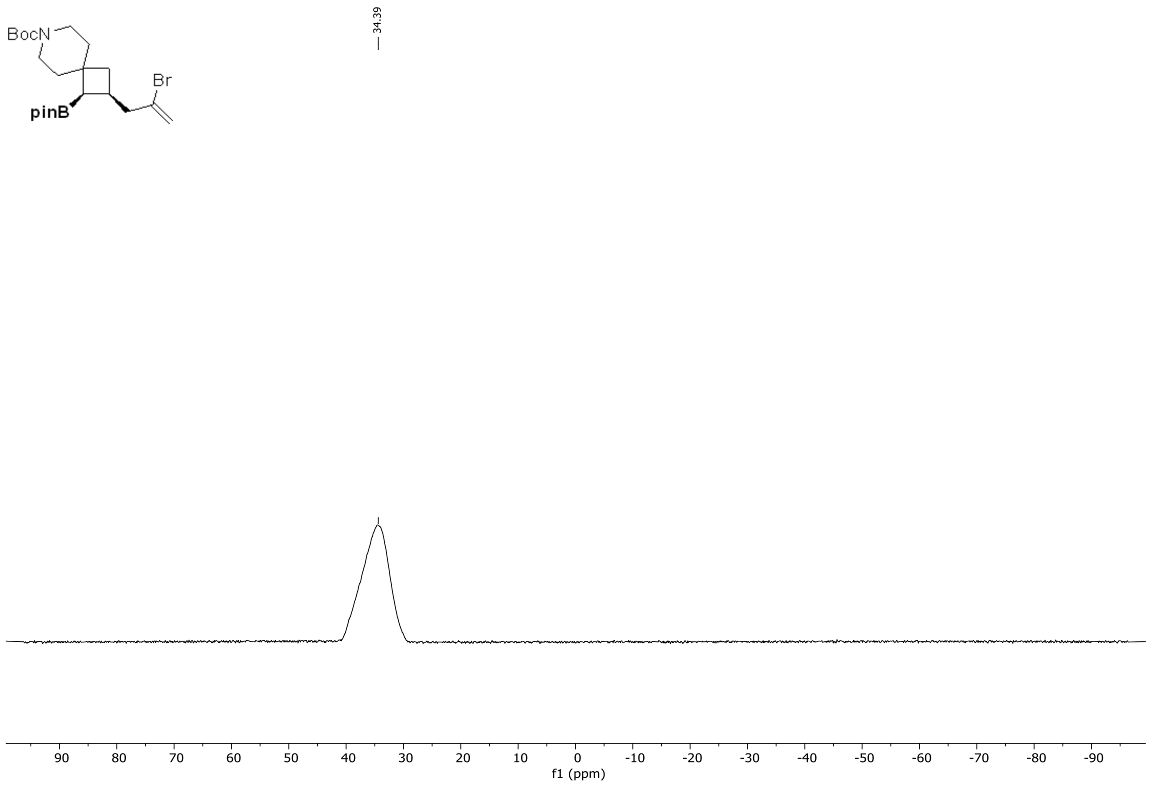

Compound 7f

^1H NMR (400 MHz, $CDCl_3$)

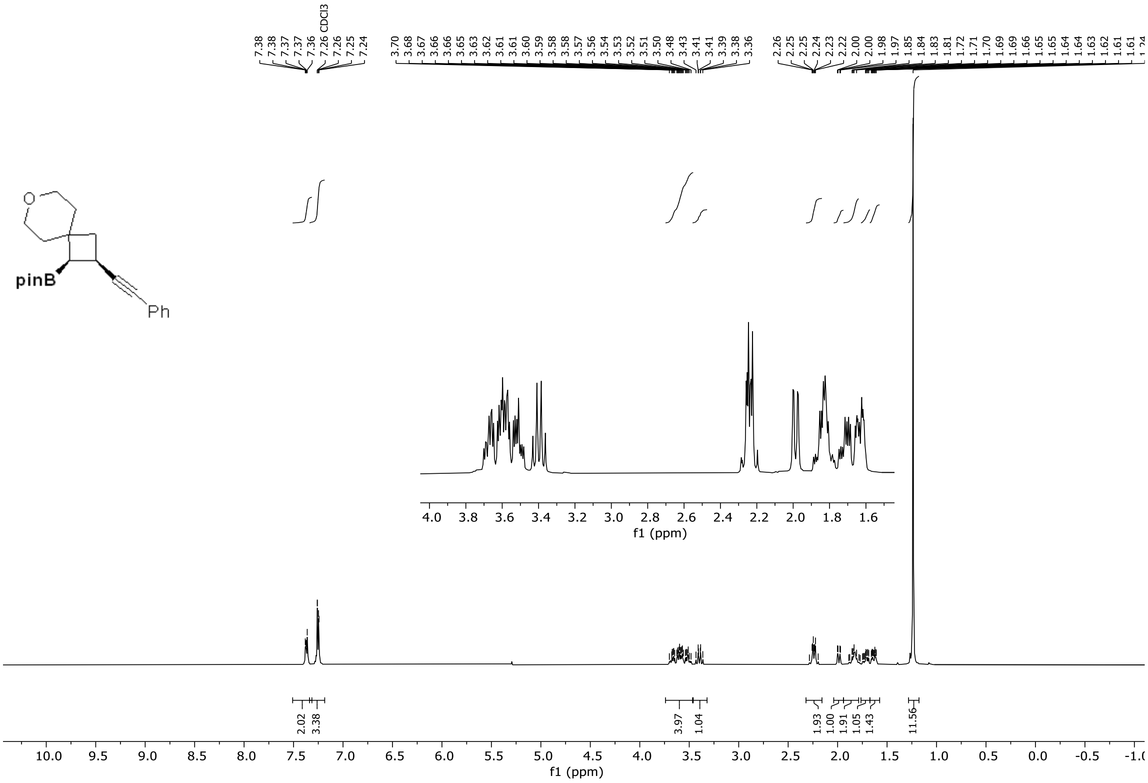

^{13}C NMR (101 MHz, $CDCl_3$)

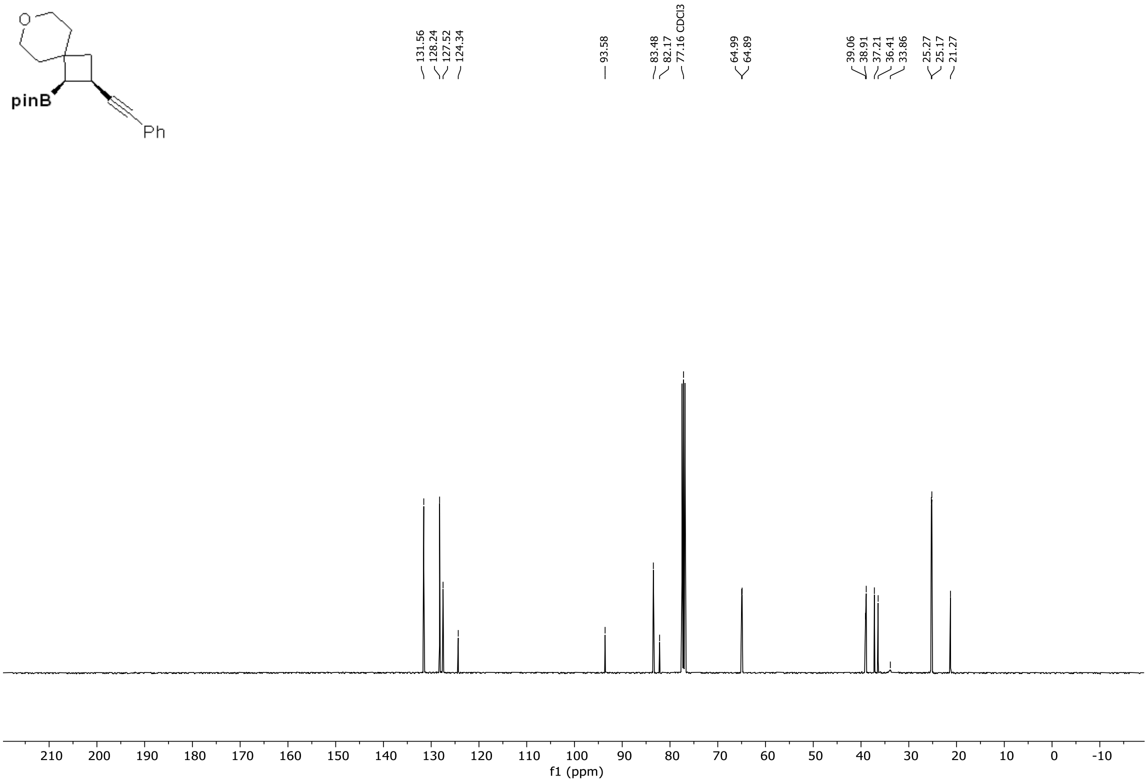

^{11}B NMR (128 MHz, CDCl$_3$)

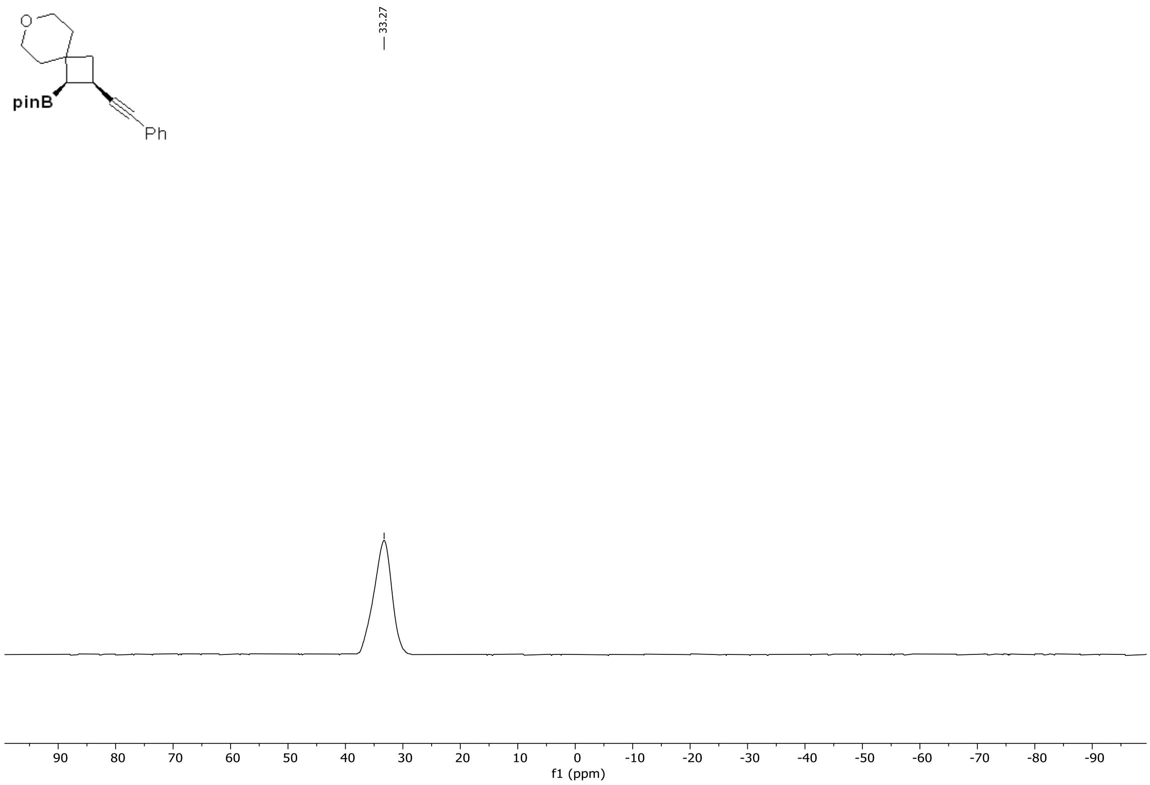

Compound 7g

^{1}H NMR (500 MHz, CDCl$_3$)

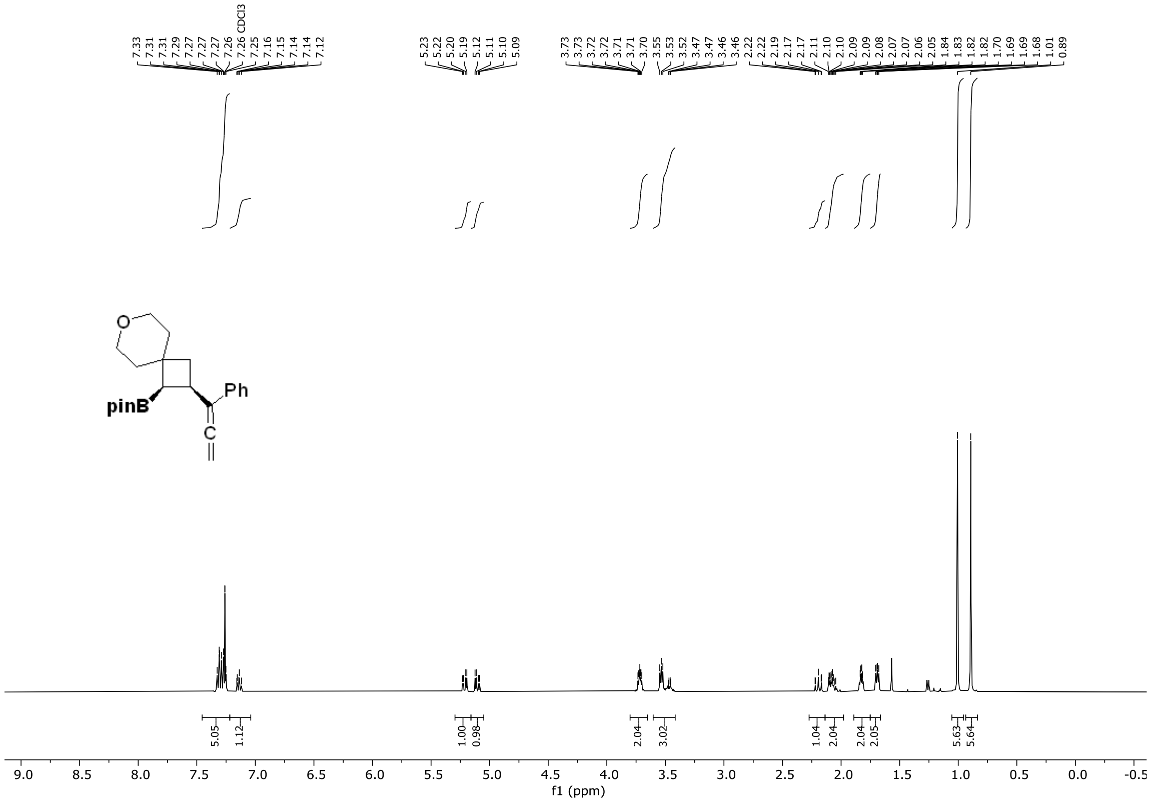

^{13}C NMR (126 MHz, $CDCl_3$)

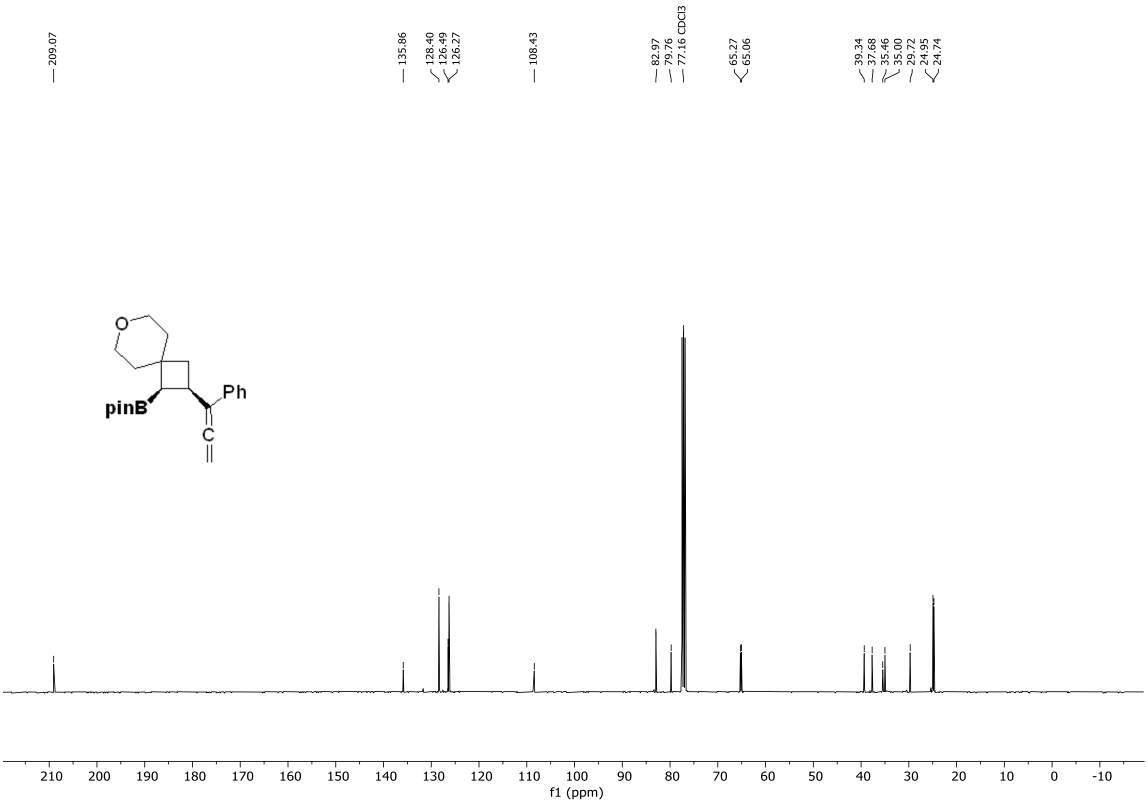

^{11}B NMR (128 MHz, $CDCl_3$)

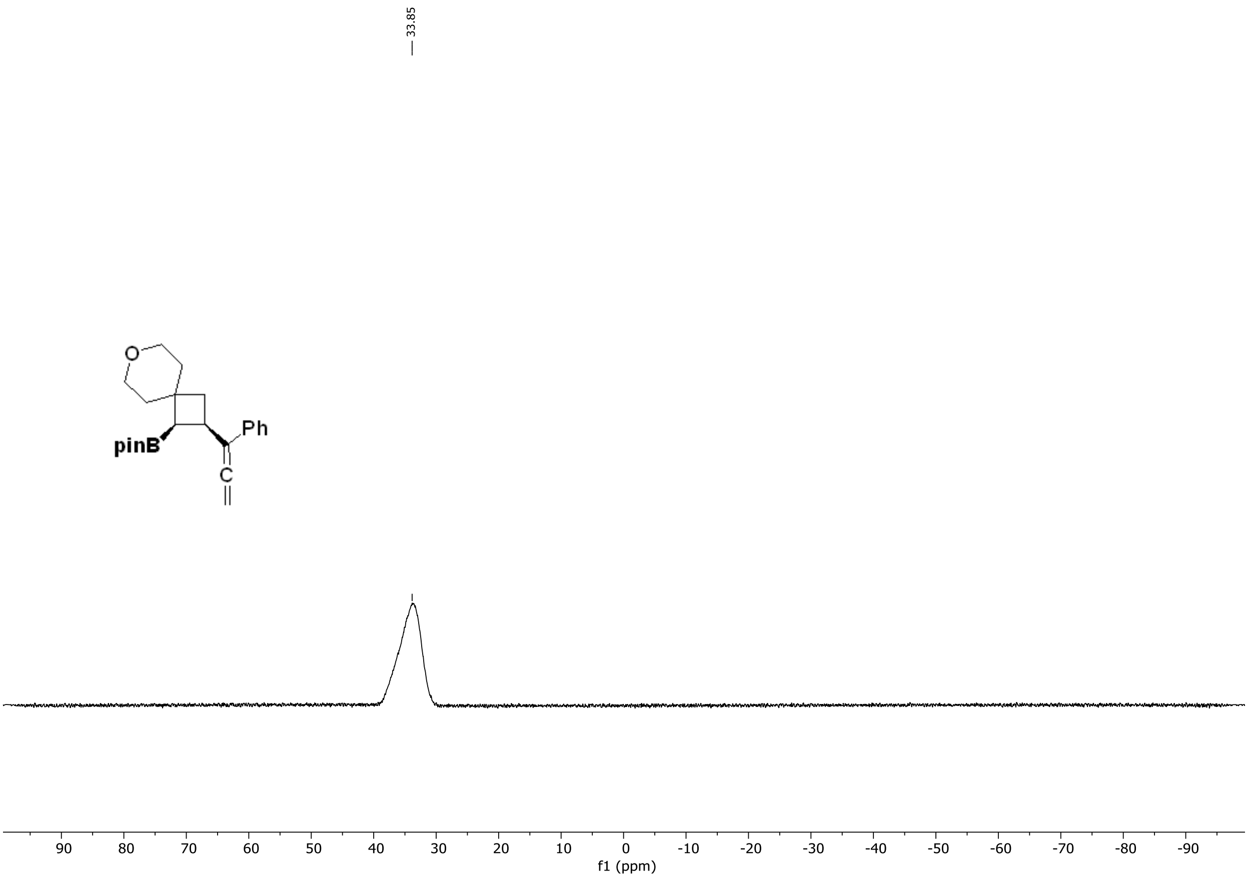

Compound 7h

^{1}H NMR (500 MHz, $CDCl_3$)

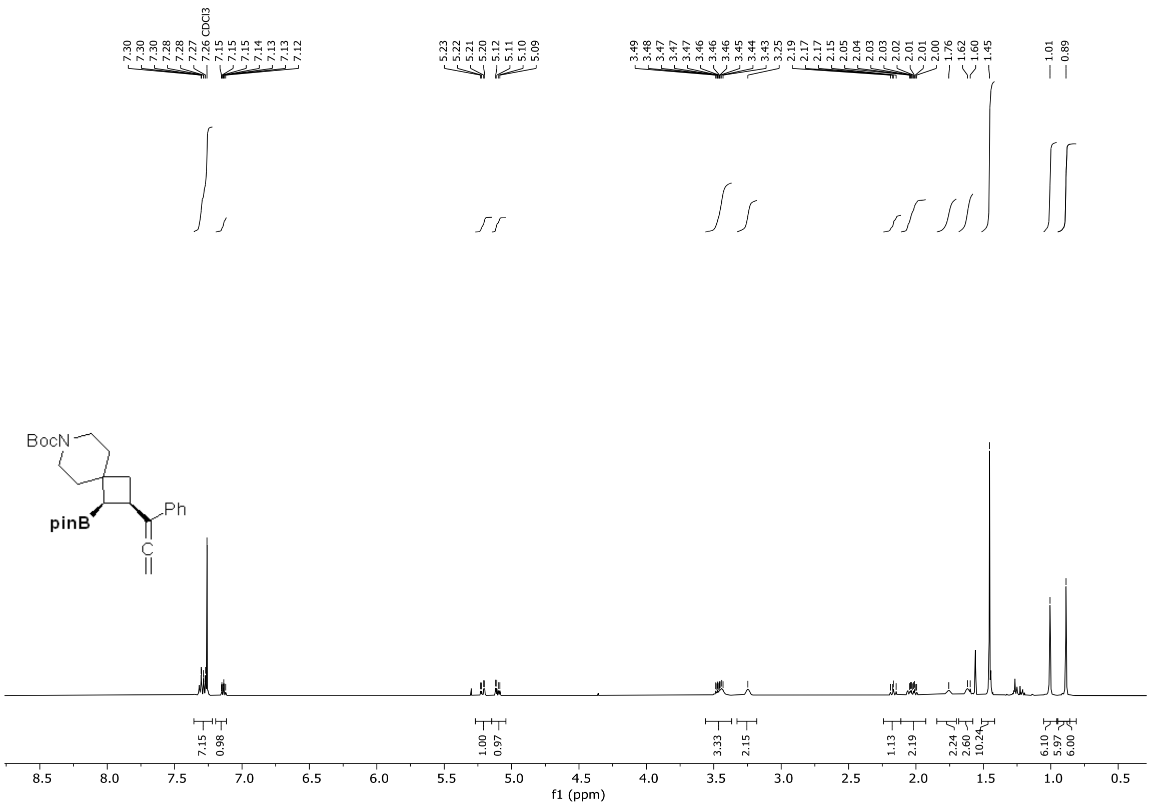

^{13}C NMR (101 MHz, $CDCl_3$)

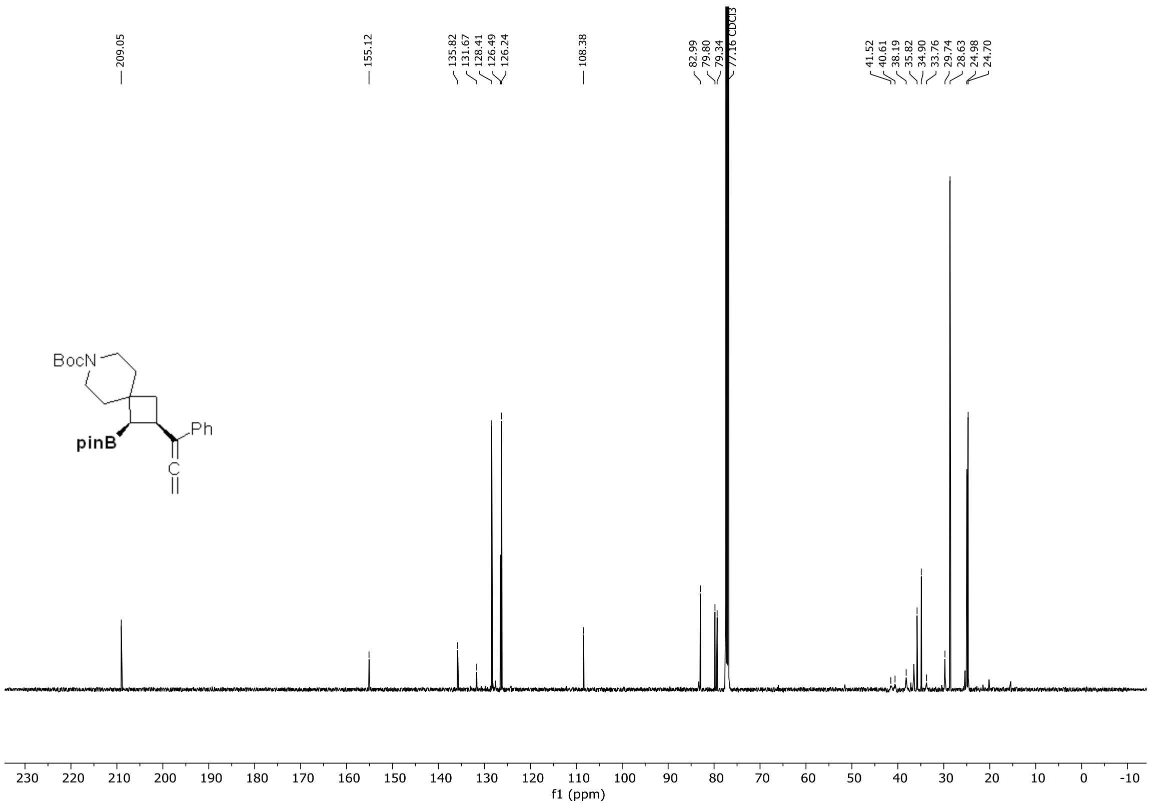

^{11}B NMR (128 MHz, $CDCl_3$)

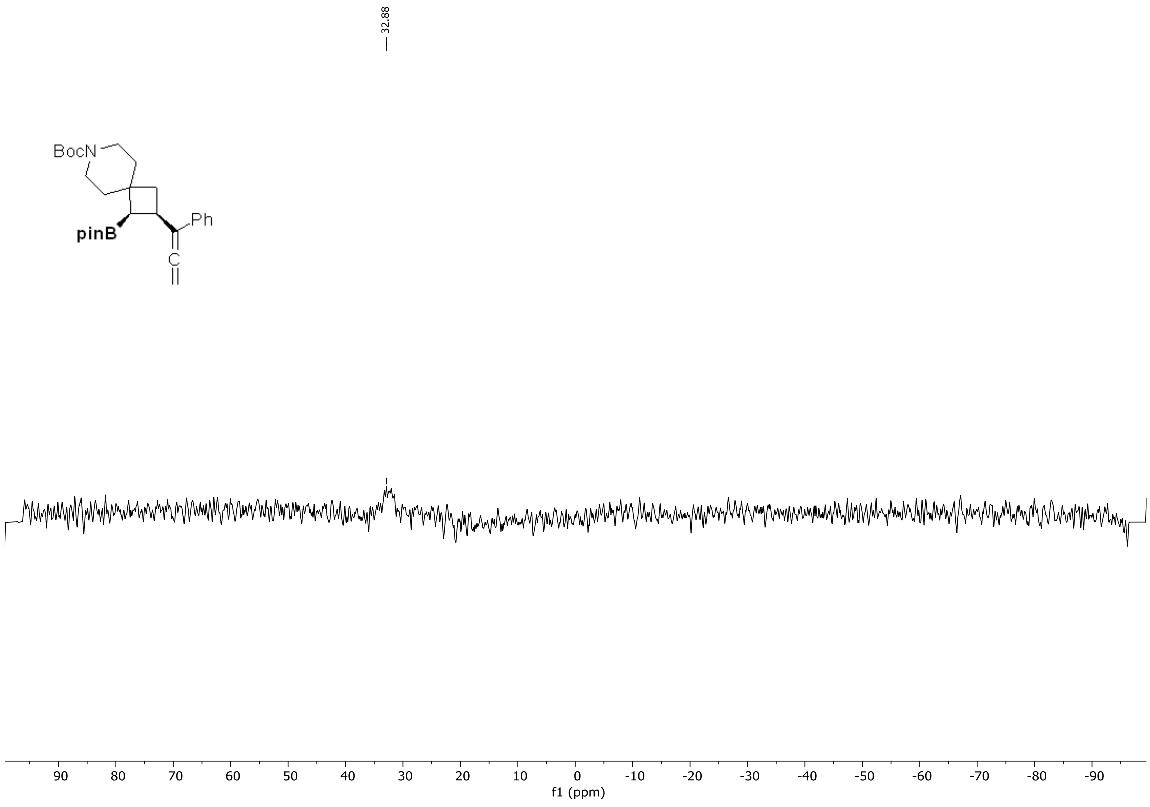

C-B bond functionalization

Compound 8

^{1}H NMR (400 MHz, $CDCl_3$)

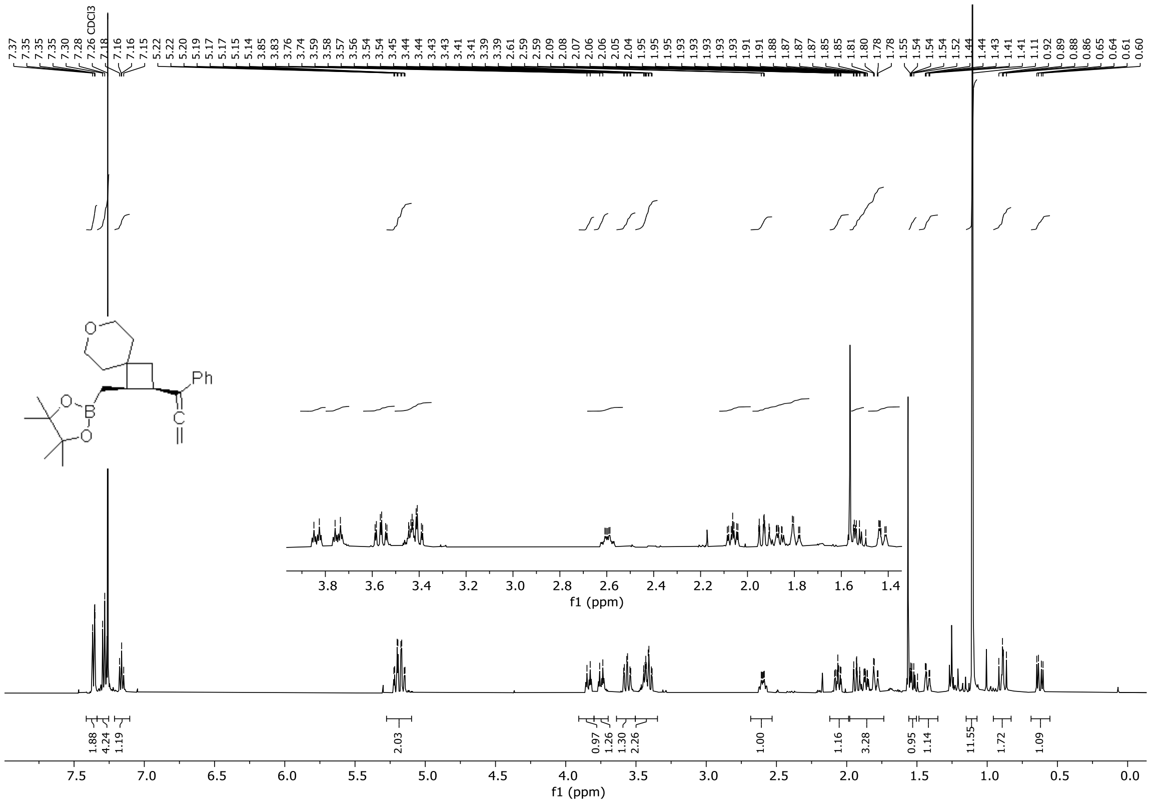

^{13}C NMR (101 MHz, $CDCl_3$)

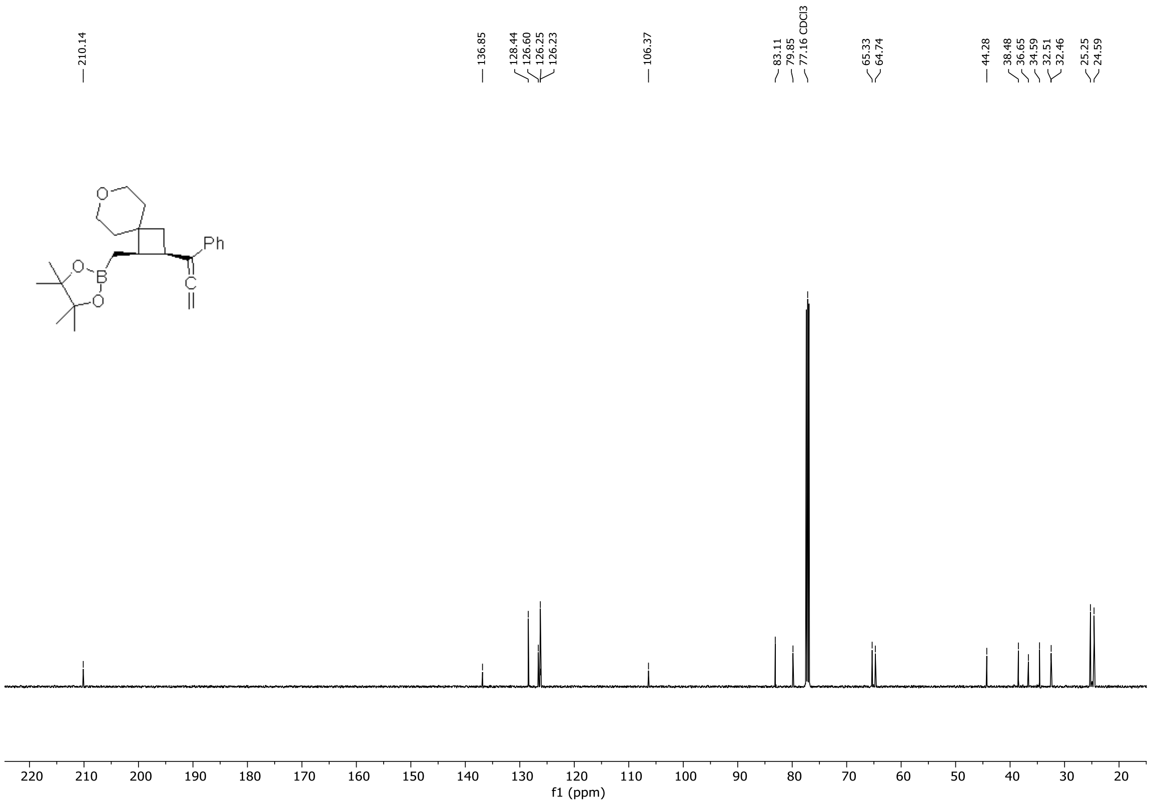

^{11}B NMR (128 MHz, $CDCl_3$)

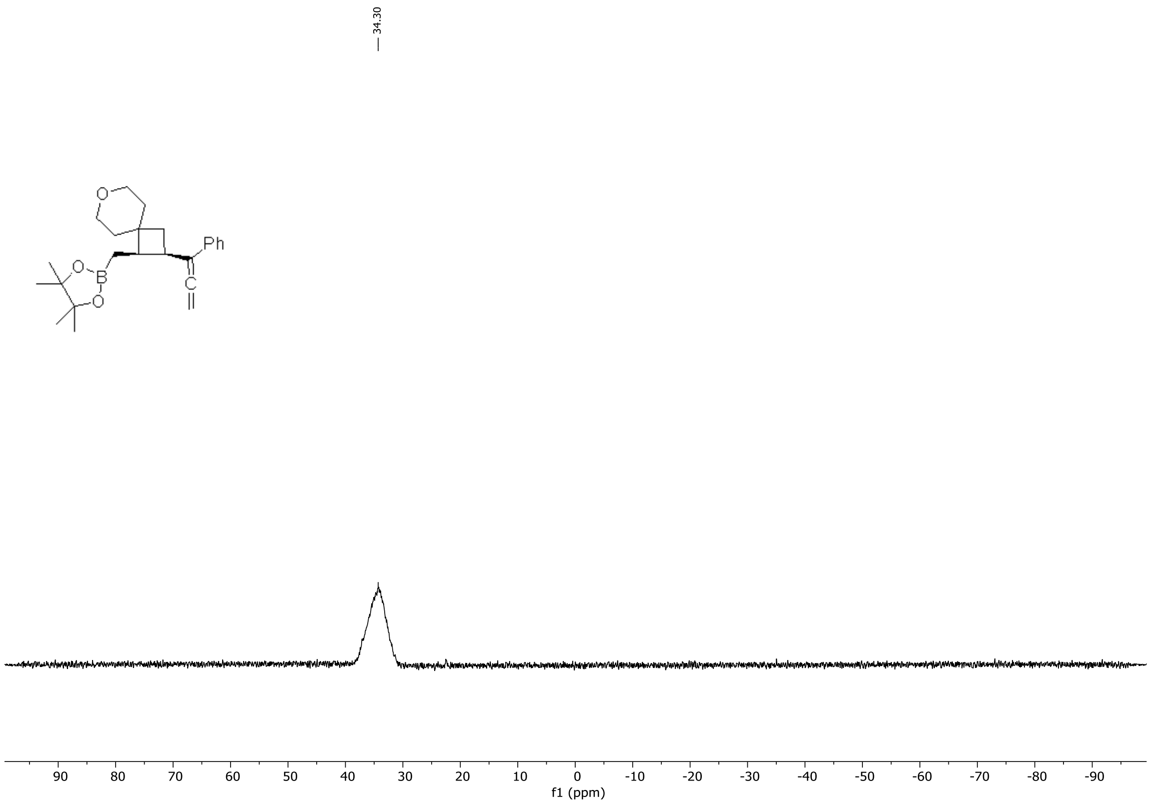

Compound 9

^1H NMR (400 MHz, $CDCl_3$)

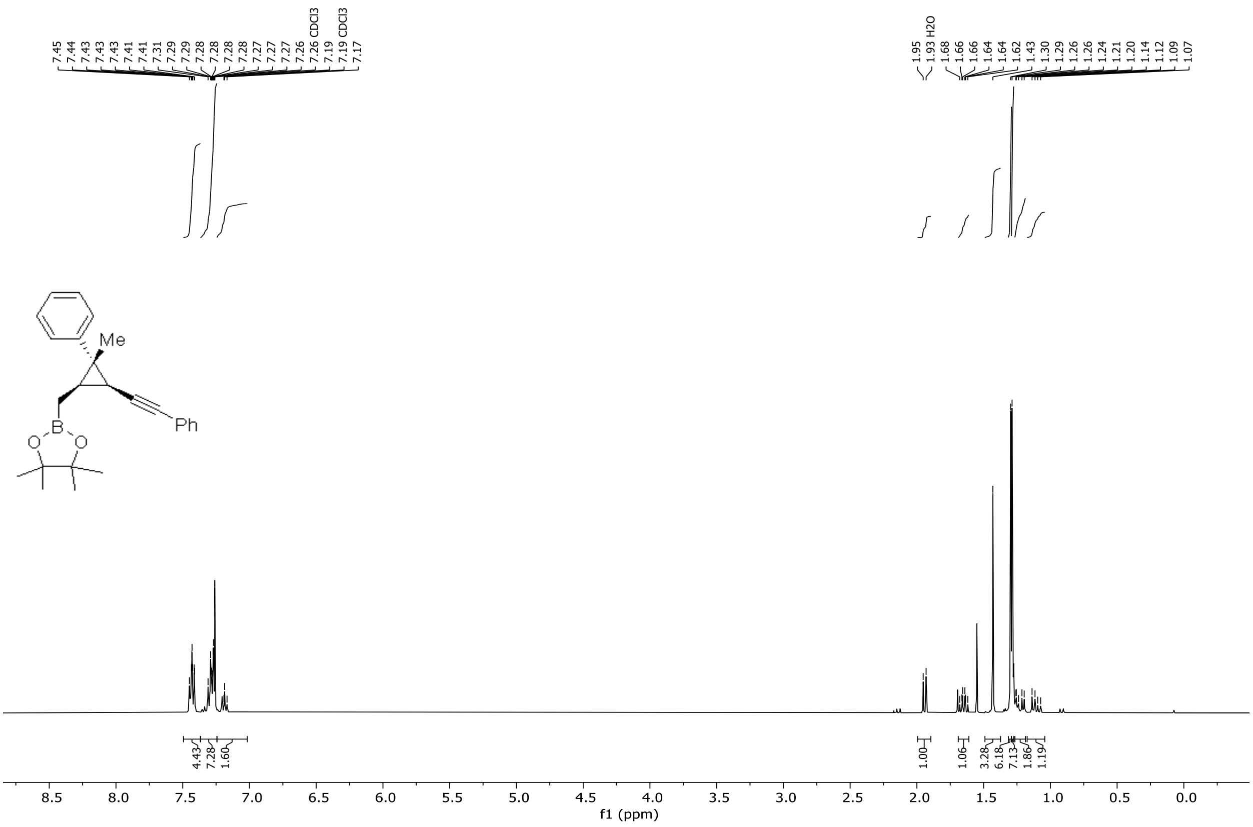

^{13}C NMR (101 MHz, $CDCl_3$)

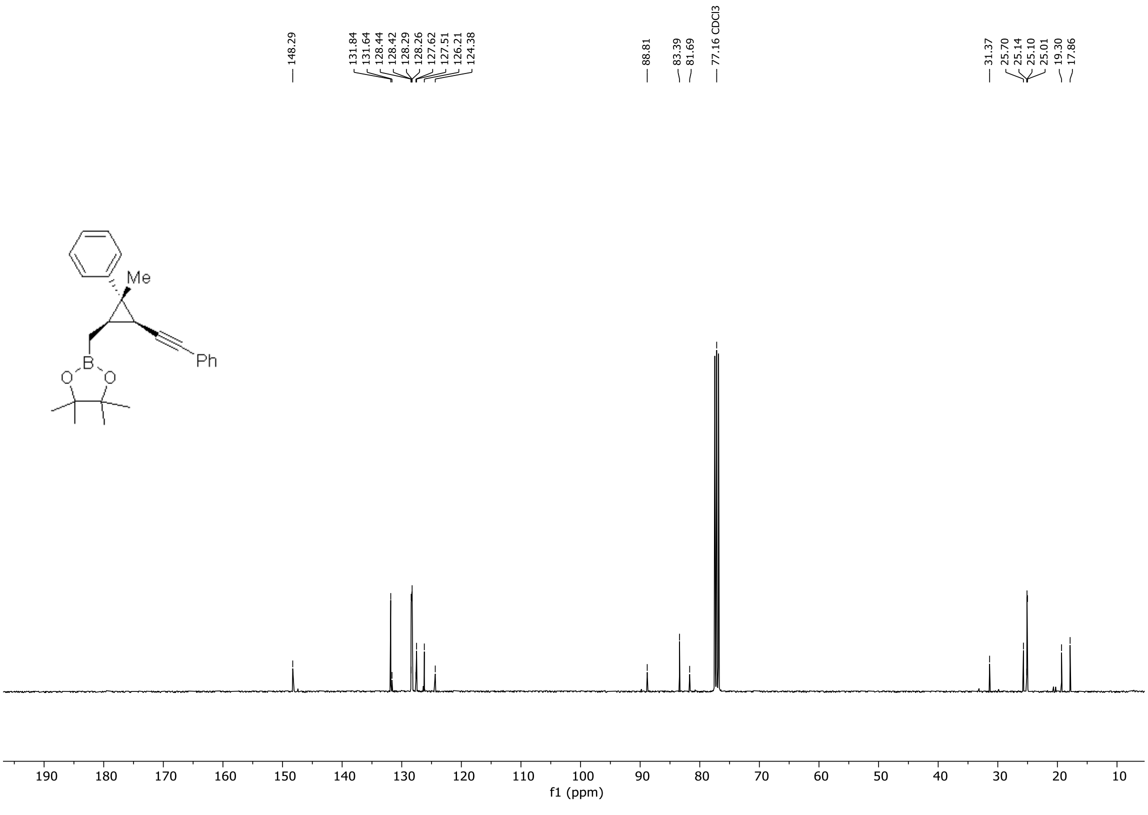

^{11}B NMR (128 MHz, $CDCl_3$)

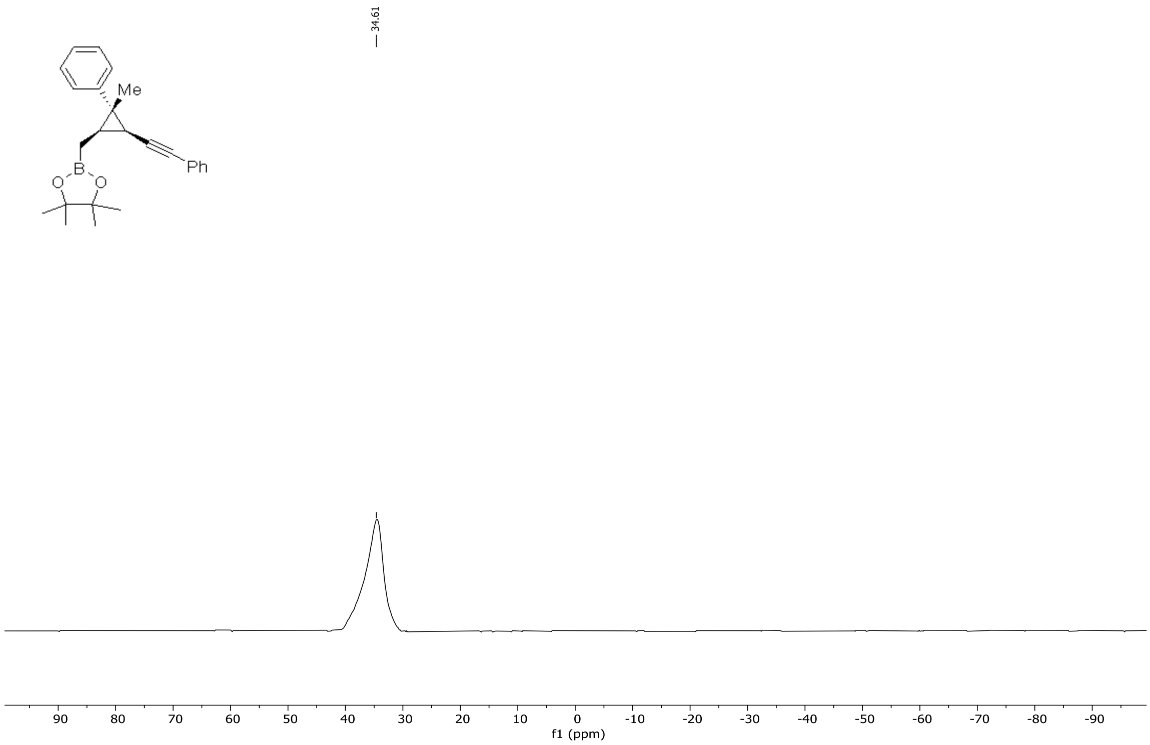

Compound 10

^{1}H NMR (400 MHz, $CDCl_3$)

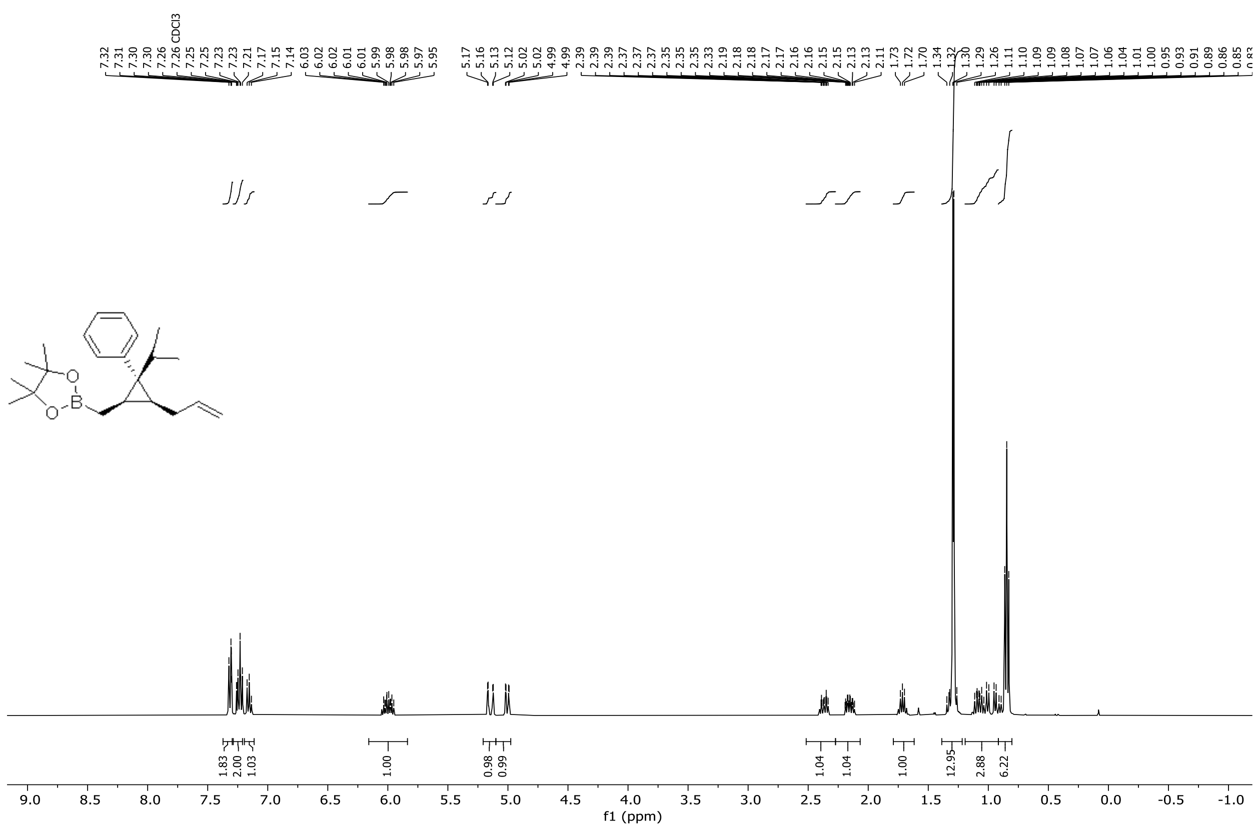

^{13}C NMR (101 MHz, $CDCl_3$)

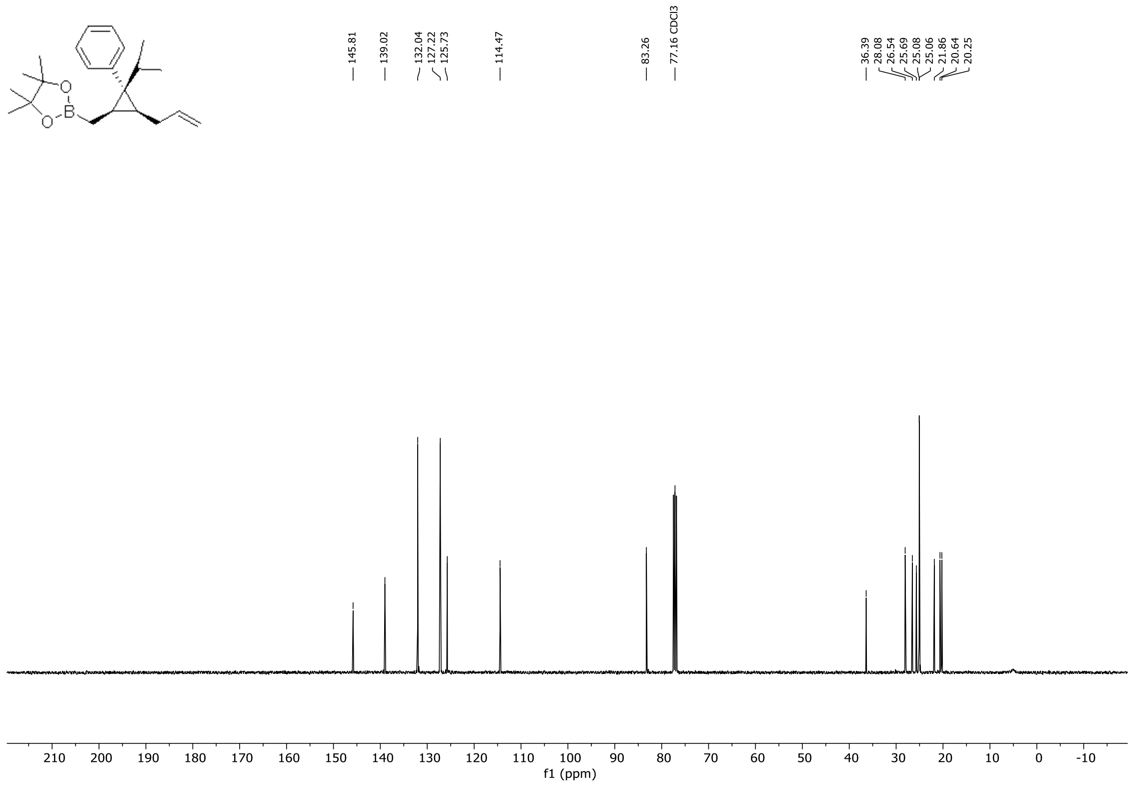

^{11}B NMR (128 MHz, $CDCl_3$)

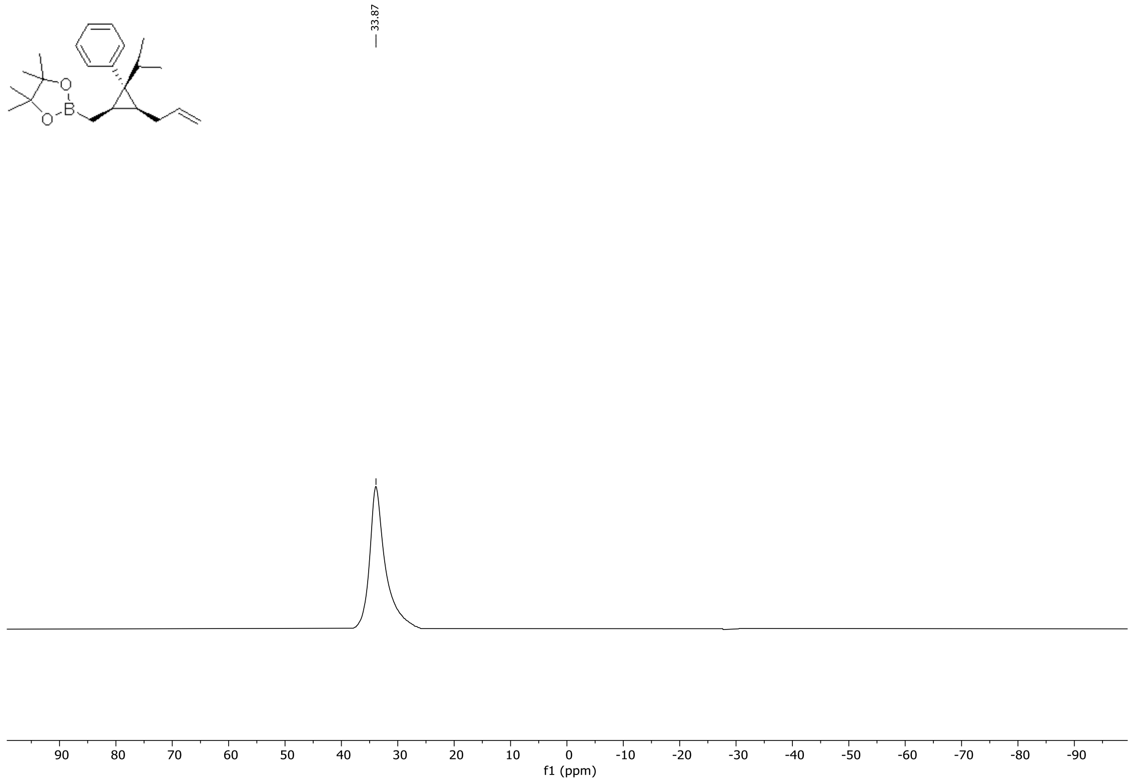

Compound 11

^{1}H NMR (400 MHz, $CDCl_3$)

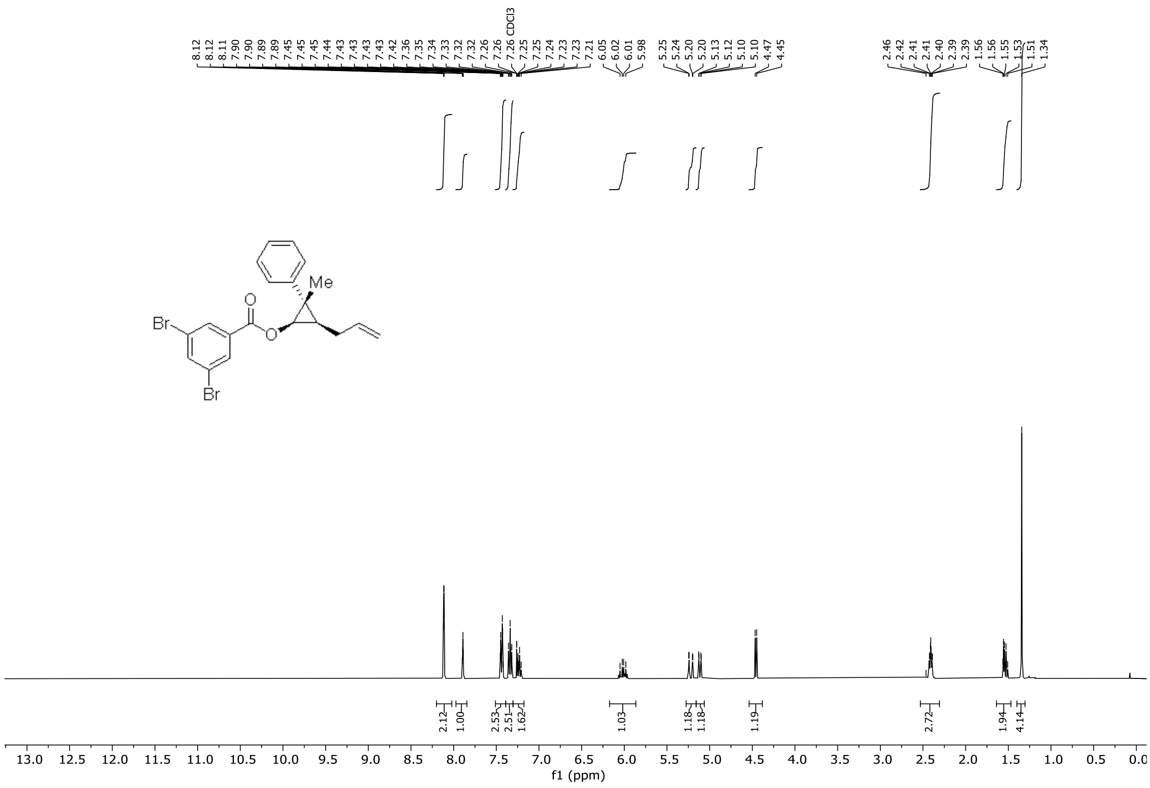

^{13}C NMR (101 MHz, $CDCl_3$)

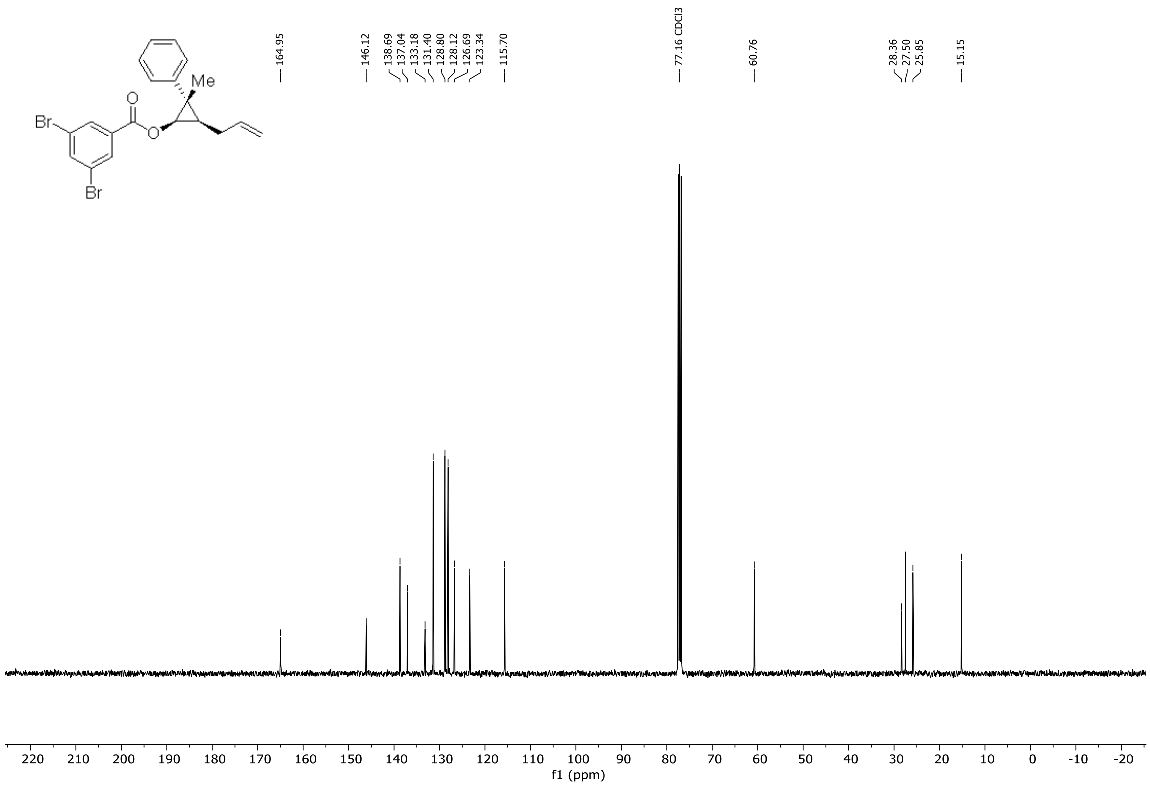

Compound 12

^{1}H NMR (300 MHz, $CDCl_3$)

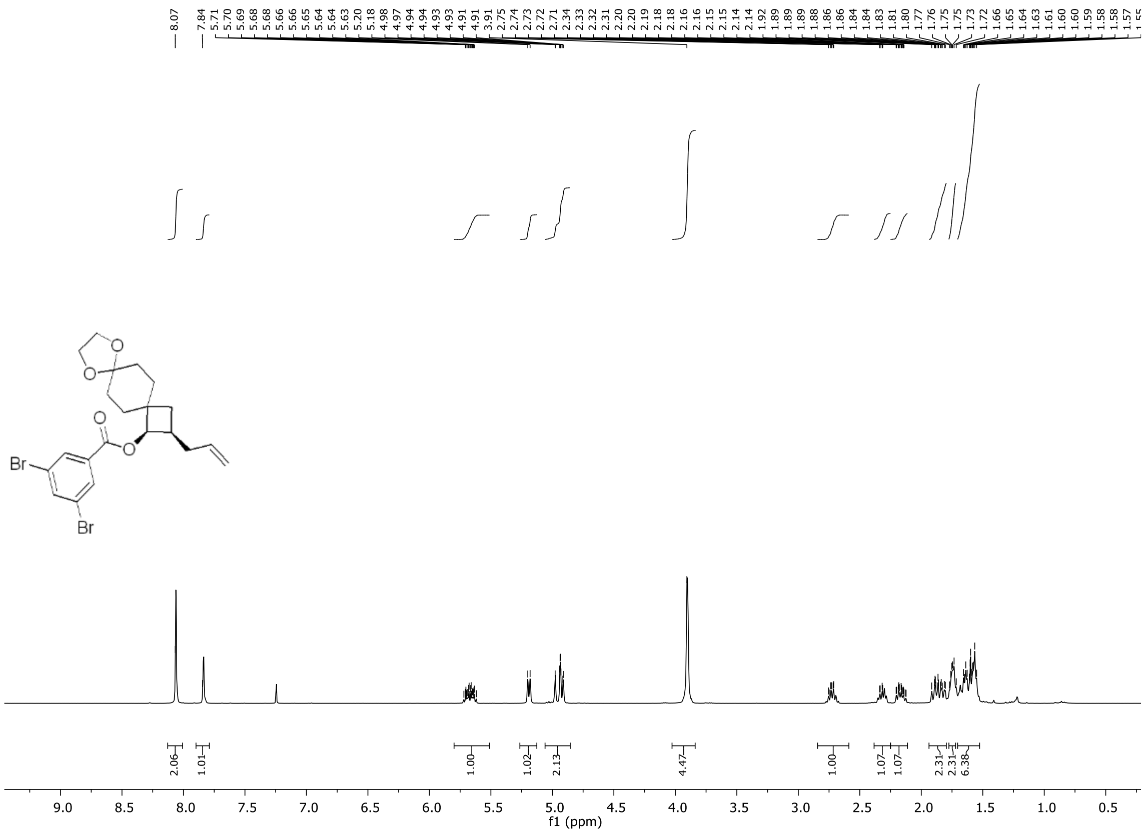

^{13}C NMR (101 MHz, $CDCl_3$)

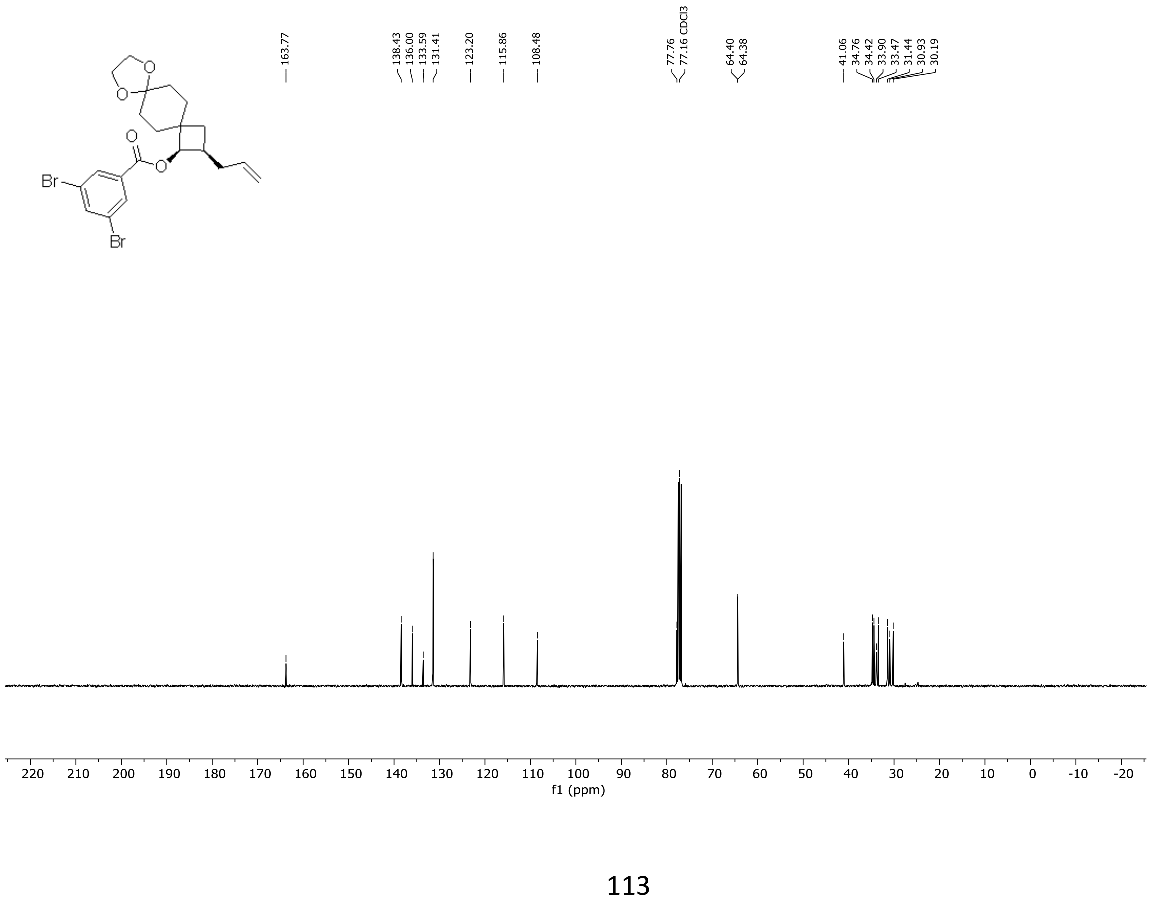

NOESY (400 MHz, $CDCl_3$)

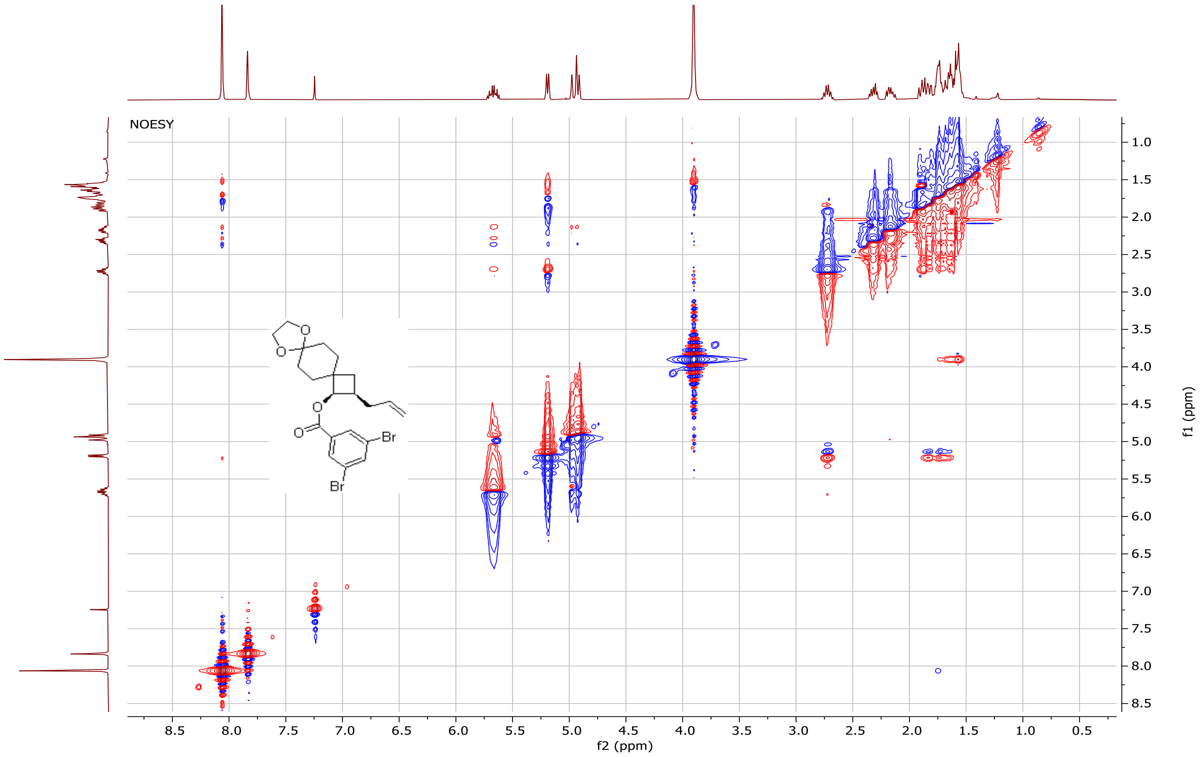

Compound 13

^1H NMR (400 MHz, $CDCl_3$)

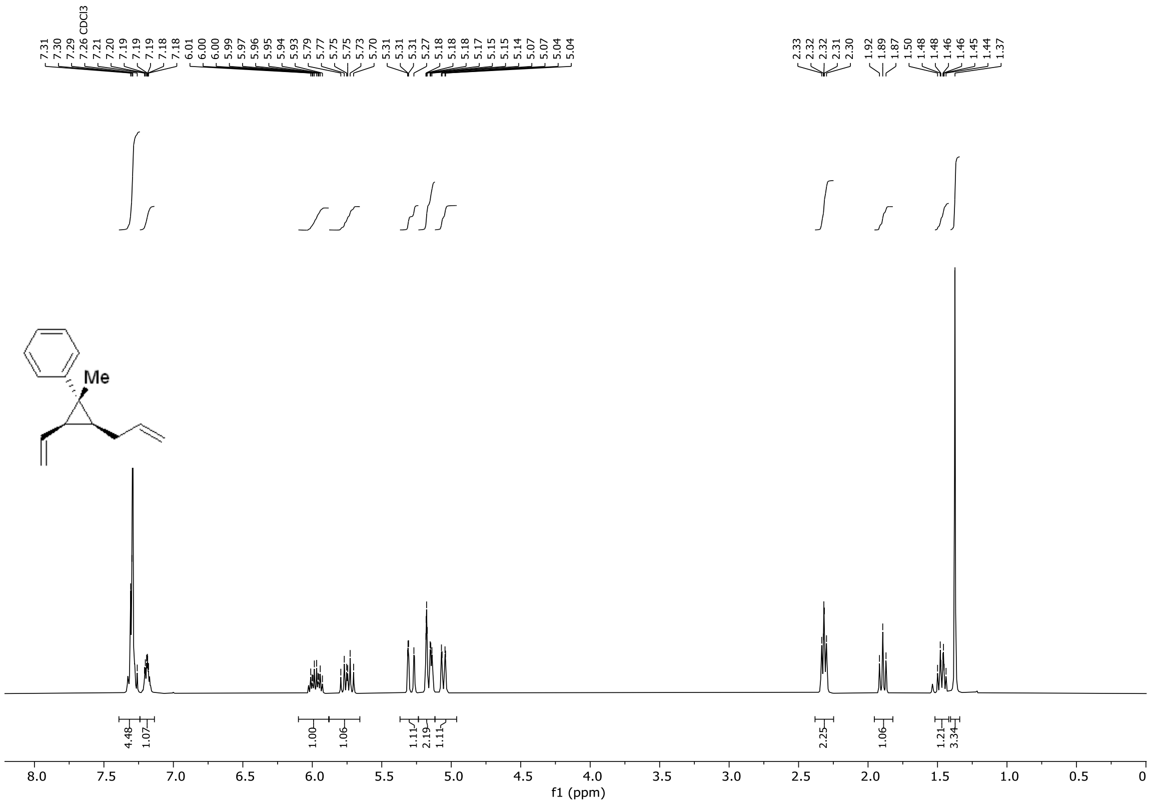

^{13}C NMR (101 MHz, $CDCl_3$)

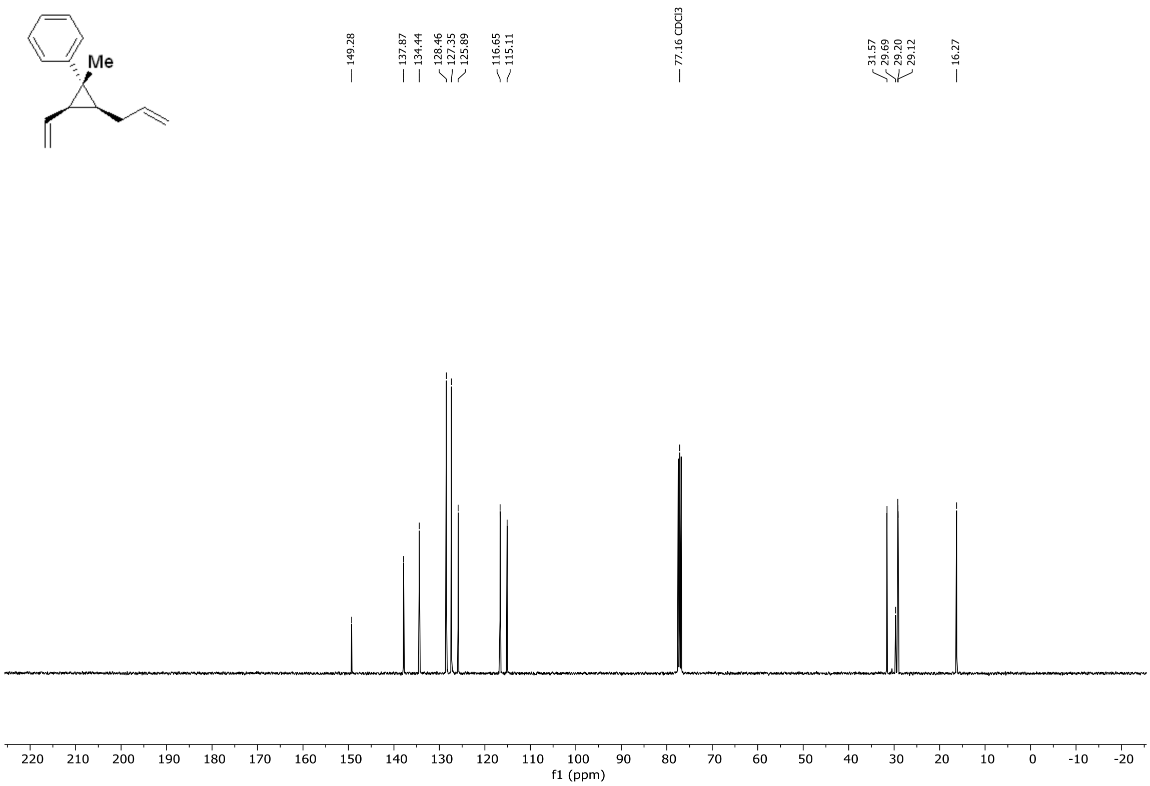

Compound 14

^{1}H NMR (400 MHz, $CDCl_3$)

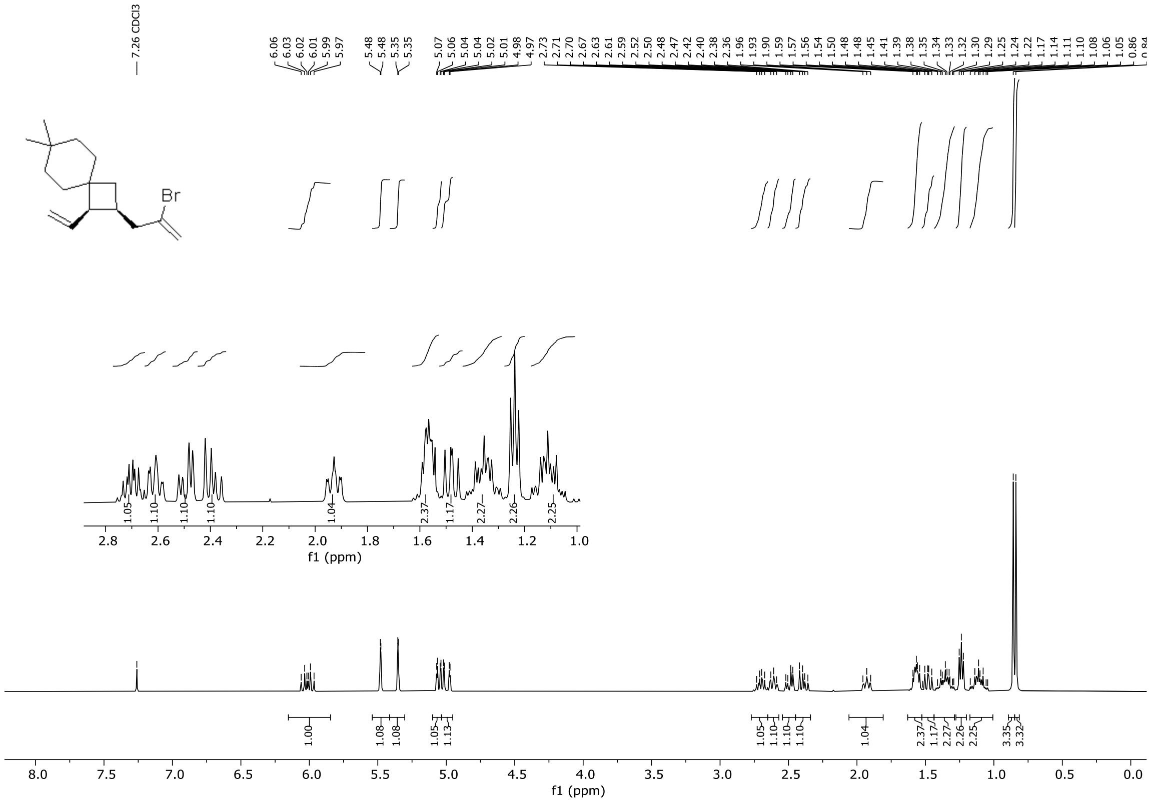

^{13}C NMR (101 MHz, $CDCl_3$)

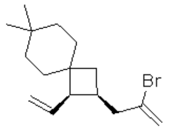

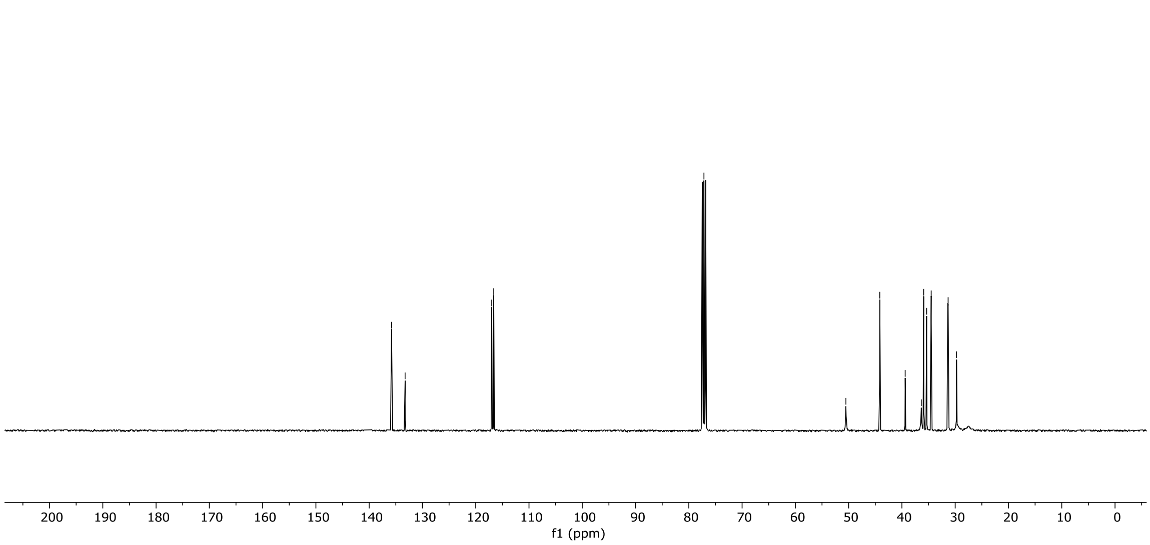

Supplement: Supplementary file 1 [file ol6c01560_si_001.pdf]
